# Csn5 inhibits autophagy by regulating the ubiquitination of Atg6 and Tor to mediate the pathogenicity of *Magnaporthe oryzae*

**DOI:** 10.1186/s12964-024-01598-7

**Published:** 2024-04-09

**Authors:** Zi-Fang Shen, Lin Li, Jing-Yi Wang, Jian Liao, Yun-Ran Zhang, Xue-Ming Zhu, Zi-He Wang, Jian-Ping Lu, Xiao-Hong Liu, Fu-Cheng Lin

**Affiliations:** 1 State Key Laboratory for Managing Biotic and Chemical Treats to the Quality and Safety of Agro-Products, Zhejiang Provincial Key Laboratory of Agricultural Microbiomics, Key Laboratory of Agricultural Microbiome (MARA), Institute of Plant Protection and Microbiology, Zhejiang Academy of Agricultural Sciences, Hangzhou, 310021 China; 2 State Key Laboratory for Managing Biotic and Chemical Treats to the Quality and Safety of Agro-Products, Zhejiang Provincial Key Laboratory of Agricultural Microbiomics, Key Laboratory of Agricultural Microbiome (MARA), Institute of Biotechnology, Zhejiang University, Hangzhou, 310058 China; 3 College of Life Sciences, Zhejiang University, Hangzhou, 310058 China

**Keywords:** COP9 signalosome, Csn5, Autophagy, Ubiquitination, Pathogenicity, Rice blast fungus

## Abstract

Csn5 is subunit 5 of the COP9 signalosome (CSN), but the mechanism by which it strictly controls the pathogenicity of pathogenic fungi through autophagy remains unclear. Here, we found that Csn5 deficiency attenuated pathogenicity and enhanced autophagy in *Magnaporthe oryzae. MoCSN5* knockout led to overubiquitination and overdegradation of MoTor (the core protein of the TORC1 complex [target of rapamycin]) thereby promoted autophagy. In addition, we identified MoCsn5 as a new interactor of MoAtg6. Atg6 was found to be ubiquitinated through linkage with lysine 48 (K48) in cells, which is necessary for infection-associated autophagy in pathogenic fungi. K48-ubiquitination of Atg6 enhanced its degradation and thereby inhibited autophagic activity. Our experimental results indicated that MoCsn5 promoted K48-ubiquitination of MoAtg6, which reduced the MoAtg6 protein content and thus inhibited autophagy. Aberrant ubiquitination and autophagy in Δ*Mocsn5* led to pleiotropic defects in the growth, development, stress resistance, and pathogenicity of *M. oryzae*. In summary, our study revealed a novel mechanism by which Csn5 regulates autophagy and pathogenicity in rice blast fungus through ubiquitination.

**Supplementary Information:**

The online version contains supplementary material available at 10.1186/s12964-024-01598-7.

## Introduction

Rice blast, caused by *Magnaporthe oryzae*, is a worldwide fungal disease [1, 2]. The conidium is the infection weapon of the rice blast fungus and germinates to form a germ tube under suitable environmental conditions; then, the tip of the germ tube expands to form an appressorium [3, 4]. The mature appressorium generates a mechanical force of up to 8.0 MPa and penetrates the plant epidermis through infection nails to form infection hypha [5]. *M. oryzae* has high genetic operability and exhibits the typical growth, development, and infection mechanism characteristics and is a model organism for studying the interaction between pathogens and host plants. The entire infection cycle of rice blast fungus, from surface recognition, adhesion, and germ tube germination to appressorium formation, infectious growth, and pathogenicity, is closely related to the protein degradation process and signal transduction pathways [6, 7]. Protein degradation systems include the autophagy‒lysosome pathway (ALP) and the ubiquitin‒proteasome system (UPS) [8, 9]. The typical signal transduction pathways include the mitogen-activated protein kinase signaling pathway (MAPK), calcium signaling pathway, G protein signaling pathway, and TOR signaling pathway, which are critical to the pathogenicity of pathogenic fungi [10–14].

The COP9 (constitutive morphogenesis number 9) signalosome (usually called the CSN) is a highly conserved protein complex found in almost all multicellular eukaryotes [15, 16]. The CSN consists of eight subunits, Csn1-Csn8, and the recently discovered ninth subunit, CSNAP; among these subunits, Csn5 and Csn6 contain the feature domain MPN (Mpr1 and Pad1 N-terminal) domain, and Csn1-4 and Csn7-8 contain the feature domain PCI (proteasome lid-CSN-initiation factor 3) domain [17, 18]. The most studied function of the CSN is to coordinate the activity of cullin-RING E3 ubiquitin ligases (CRLs) [19]. CRLs are an E3 ubiquitin-ligating enzyme family that has a significant impact on cellular regulation by binding ubiquitin to target proteins [20]. Specifically, CSN negatively regulates the E3 ubiquitin-ligating enzyme activity of CRLs by uncoupling the ubiquitin-like protein Nedd8 (neural precursor cell expressed, developmentally downregulated 8) from the cullin subunit (deneddylation) or combining with deneddylated CRLs, thus relieving the interaction between CRLs and E2 ubiquitin-conjugating enzymes and ubiquitin substrates [21]. Recent studies have shown that the CSN plays a key role in the development and cell processes of eukaryotes. The mammalian CSN plays a complex regulatory role in a series of different biological processes, such as signal transduction, autophagy, circadian rhythm, and cell development [22–24]. In pathogenic fungi, CSNs play indispensable roles in autophagy, ubiquitin protein degradation, aerial hyphal growth, and pathogenicity [25]. The absence of any CSN subunit is fatal for higher eukaryotes but not for fungi; thus, fungi are good models for studying the molecular mechanism of the CSN. Previous studies have shown that the absence of Csn5 in *Arabidopsis thaliana* or *Beauveria bassiana*, the absence of Csn6 in *M. oryzae*, and the absence of any CSN in *Fusarium graminearum* are correlated with the growth, development, and pathogenicity of pathogenic fungi [26–29]. To our knowledge, apart from the interaction between Csn6 and Atg6 in rice blast fungus, which regulates autophagy and virulence [26], little is known about the biological function of the CSN in regulation of autophagy and pathogenicity in pathogenic fungi.

Autophagy is a catabolic membrane transport process that is highly conserved in eukaryotes. Damaged or excess cell components are encapsulated in double-membrane autophagy vesicles and sent to vacuoles (fungi and plants) or lysosomes (mammals) for degradation and recycling, which helps maintain cell homeostasis and organism survival under various stresses [7, 30, 31]. Moderate autophagy is necessary for the complete virulence of pathogenic fungi [32]. Five main systems are involved in the autophagy process [33]. Inhibition of TOR activity by rapamycin (TORC1 inhibitor) leads to dephosphorylation of Atg13 (autophagy-related protein 13), which then binds to Atg17 to form the Atg1-Atg13-Atg17 complex localized at the phagophore assembly site (PAS), marking the initiation of autophagy [34]. After the induction of autophagy, the autophagy membrane extend to the PAS to form vesicles, which eventually form mature autophagosomes that fuse with vacuoles or lysosomes [31]. The ubiquitin-like Atg8/Atg12 conjugation system [35], the phosphatidylinositol 3-phosphate kinase complex 3 (PI3KC3) [36], and the Atg9 recycling complex are involved in this process [37]. Atg6, a homolog of the vacuole protein sorting (Vps) 30 in yeast and Beclin1 in mammals, participates in the assembly of the PI3KC3 complex by interacting with Atg14 and Vps34, which is crucial for the activity of the PI3KC3 complex and the localization of autophagy-related proteins to the PAS [38]. Although the biological functions of autophagy have gradually been revealed, the molecular and regulatory mechanisms of autophagy in phytopathogenic fungi remain poorly understood.

Although the importance of Csn5 has been confirmed in different species, to our knowledge, its relationship with autophagy in filamentous fungi has not been reported. In this study, we found that MoCsn5 is a novel autophagy inhibitor in *M. oryzae* that inhibits autophagy by regulating ubiquitination, thereby mediating pathogenicity. Further analyses revealed the following mechanism: MoCsn5 promoted the K48-ubiquitination of MoAtg6 by interacting with MoAtg6, which was found to be more susceptible to degradation by the ubiquitin‒proteasome pathway and thereby hindered the formation of autophagosomes. Moreover, MoCsn5 inhibited the K48-ubiquitination and UPS-mediated degradation of MoTor (the core protein of the TORC1 complex [target of rapamycin]), thereby maintaining the inhibitory effect of the TORC1 complex on autophagy. In addition, abnormal ubiquitination and autophagy induced by MoCsn5 deficiency led to pleiotropic defects in growth, development, stress resistance, and pathogenicity in *M. oryzae*. In summary, we characterized the novel function of Csn5 in autophagy and provided a new perspective on the mechanism by which ubiquitination regulates autophagy.

## Results

### Targeted gene deletion of the *MoCSN* subunit reduces the growth, sporulation, and pathogenicity of *M. oryzae*

Previously, we identified the CSN complex in *M. oryzae* and found that its component Csn6 is involved in fungal development, autophagy, ubiquitination, and pathogenicity [26]. To further investigate the biological function of the CSN complex in *M. oryzae*, high-throughput target gene deletion strategies were used to knock out *MoCSN1*, *MoCSN2*, *MoCSN3*, *MoCSN4*, *MoCSN5* and *MoCSN7a* in *M. oryzae* 70-15 (wild type) (Figure S1A). At least four positive knockout transformants with similar phenotypes were obtained for each *CSN* gene, and single-copy validation was performed (Figure S1B and C). To determine the roles of various CSN subunits in the growth of rice blast fungus, the obtained CSN mutants and 70-15 were inoculated on complete medium (CM) or minimum medium (MM) for 9 days. As shown in Figure S2A and B, compared with 70-15, the destruction of any CSN subunit led to a sharp decrease in mycelial growth. After the corresponding *CSN* gene was replenished, the mycelial growth rates of all replenished strains (*Mocsn1-C*, *Mocsn2-C*, *Mocsn3-C*, *Mocsn4-C*, *Mocsn5-C* and *Mocsn7a-C*) were restored to those of the wild type (WT) strain (Figure S2A and B). Further phenotypic analysis indicated that all subunits of the CSN are necessary for spore production and complete pathogenicity (Figure S2C and D), indicating that the CSN complex is a key regulator of nutritional growth, spore production, and pathogenicity in rice blast fungus.

Although previous studies have shown that the CSN complex is closely related to the pathogenicity of phytopathogenic fungi, the specific regulatory mechanisms involved are still unclear. MoCsn6 is involved in the regulation of autophagy in *M. oryzae* [26]. Therefore, we used yeast two-hybrid methods to preliminarily identify interactions between CSN subunits and autophagy-related proteins. Yeast two-hybrid experiments showed that MoCsn1, MoCsn3, and MoCsn4 all interacted with MoAtg5, MoAtg6, MoAtg14, MoAtg16, and MoAtg17, that MoCsn4 interacted with MoAtg5, MoAtg12, and MoAtg18, that MoCsn5 interacted with MoAtg6 and MoAtg14, and that MoCsn7a interacted with MoAtg5 and MoAtg6 (Figure S3A). These results suggest that the CSN complex may mediate the pathogenic ability of *M. oryzae* by regulating autophagy, but the exact mechanism still needs to be explored.

### Identification of the Csn5 protein in rice blast fungus

In this study, we focused on MoCsn5, the key subunit of the CSN complex, to explore its function in *M. oryzae*. The amino acid sequence (344 aa) encoded by *MGG_05274* was compared in the NCBI database. The results showed that this protein had 53.31% homology with *Homo sapiens* Csn5 and 66.08% homology with *F. graminearum* Csn5 (Figure S4A); therefore, we named this protein MoCsn5. A phylogenetic tree was constructed based on the Csn5 amino acid sequences of *M. oryzae* (XP_003712833.1), *F. graminearum* (XP_011318525.1), *Mus musculus* (NP_038743.1), *Caenorhabditis elegans* (NP_500841.1), *Aspergillus fumigatus* (XP_755961.2), *H. sapiens* (NP_006828.2), and *Neurospora crassa* (XP_956786.1) (Figure S4B). The phylogenetic tree showed that the Csn5 of *M. oryzae* had greater homology with the Csn5 of *F. graminearum* than with those of *C. elegans*, *M. musculus*, and *H. sapiens* (Figure S4B). Csn5 is the fifth subunit of the CSN, which is highly conserved in eukaryotes and contains a conserved MPN domain (Figure S4C).

### Δ*Mocsn5* is deficient in vegetative growth and sporulation

**Fig. 1:**
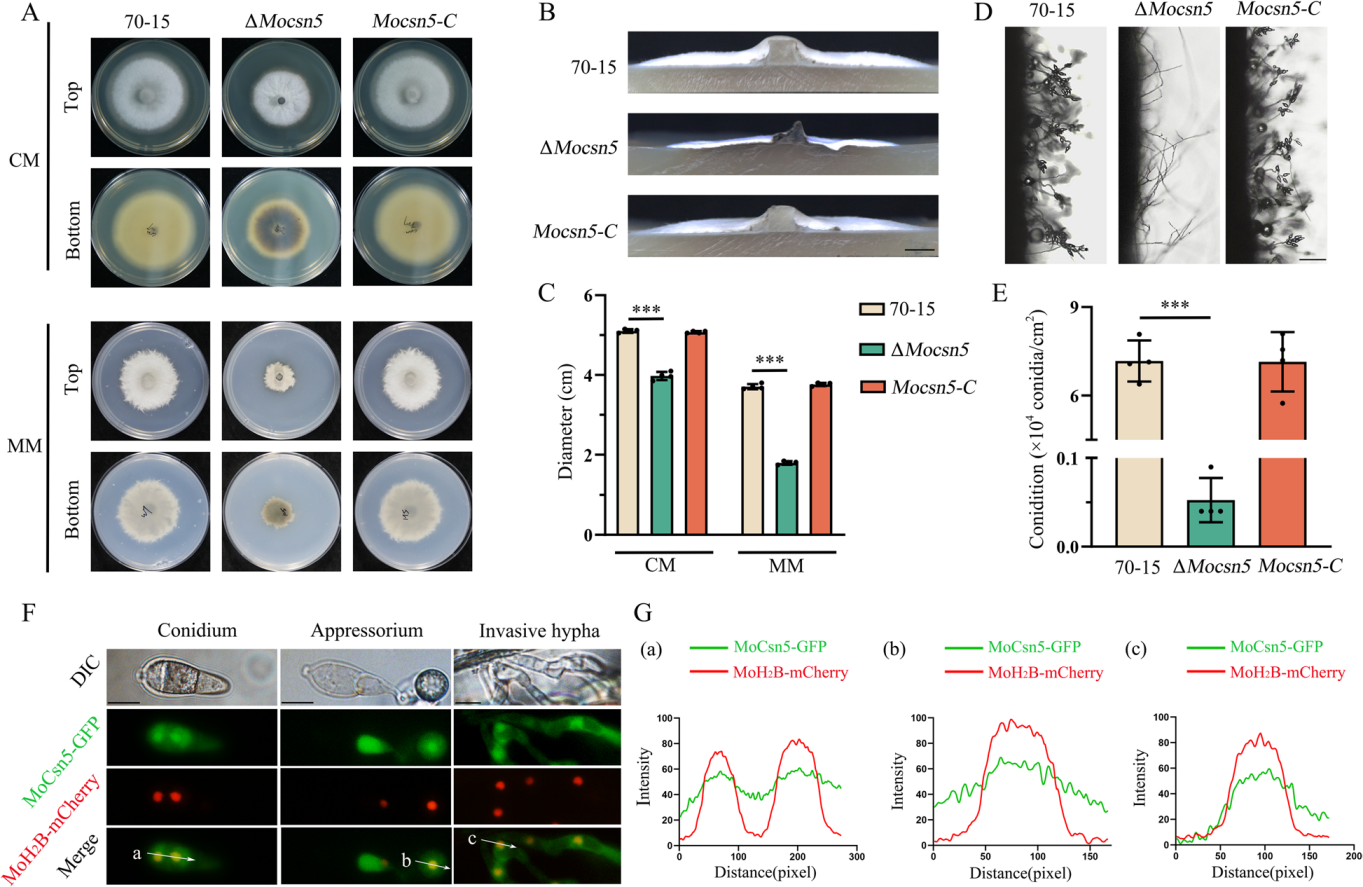
MoCsn5 is required for vegetative growth and conidiation. **A** Colony morphology of 70-15, Δ*Mocsn5* and *Mocsn5-C*. The strains were grown on CM and MM plates for 9 days. **B** Hyphal growth of 70-15, Δ*Mocsn5* and *Mocsn5-C* on solid CM. **C** Statistical analysis of the colony growth diameter. The data were analyzed using GraphPad Prism 8.0 software. The error bars represent the standard deviations. ***P < 0.001. **D** Conidiophores of 70-15, Δ*Mocsn5* and *Mocsn5-C*. The strains were cultivated in an incubator at 25 ℃ for 9 days and observed under an optical microscope. **E** Statistical analysis of conidia production. The data were analyzed with GraphPad Prism 8.0 software. ***P < 0.001. **F** Localization of MoCsn5 in the conidium, appressorium and invasive hypha. The MoH_2_B-mCherry vector was transformed into the complementation strain with the MoCsn5-GFP label. Conidia were harvested from colonies that were cultivated in solid CM for 9 days. The conidial suspension (5 × 10^4^ ml^−1^) was dripped onto a hydrophobic film and incubated in a humid chamber at 22 °C for 24 h. The red fluorescence and green fluorescence in the spores and appressoria were observed under a fluorescence microscope. Bar: 10 μm. **G** The fluorescence densities of MoH_2_B-mCherry and MoCsn5-GFP were analyzed using ImageJ software

To further explore the basic biological functions of MoCsn5, growth and spore production assays were performed. The Δ*Mocsn5* strain showed a significantly slower growth rate on both CM and MM (Fig. 1A). As shown in Fig. 1B, the aerial hyphae of Δ*Mocsn5* were thinner than those of 70-15 and *Mocsn5-C*. Compared with those of 70-15 and *Mocsn5-C*, the colony diameter of Δ*Mocsn5* on CM was decreased by 22.10%, and that on MM was decreased by 51.38% (Fig. 1C). The conidium pedicels of 70-15 and *Mocsn5-C* show typical branching structures and bear more conidium, whereas Δ*Mocsn5* has few fascicular conidial pedicels, and most of them are single-branching structures with few spores (Fig. 1D). Statistical analysis revealed that the spore production of Δ*Mocsn5* was significantly decreased to only 0.73% of that of 70-15 (Fig. 1E). In summary, MoCsn5 was essential for the vegetative growth and sporulation of rice blast fungus.

Then, we investigated the subcellular localization of MoCsn5. The MoCsn5-GFP (green fluorescent protein) vector was constructed and transformed into the Δ*Mocsn5* mutant. As shown in Fig. 1F, the green fluorescence of MoCsn5-GFP was observed in the conidium, appressorium and invasive hypha. To confirm whether MoCsn5-GFP is localized in the nucleus and cytoplasm, the nuclear location marker protein MoH_2_B-mCherry was transformed into the complemented strain with the MoCsn5-GFP label through an *Agrobacterium tumefaciens*-mediated transformation (ATMT) strategy. The green fluorescence of MoCsn5-GFP overlapped with that of the nucleus labeled with MoH_2_B-mCherry (Fig. 1G). These data indicate that MoCsn5 is localized in the cytoplasm and nucleus of *M. oryzae*.

### MoCsn5 is involved in conidial germination, appressorium formation, and pathogenicity in *M. oryzae*

**Fig. 2:**
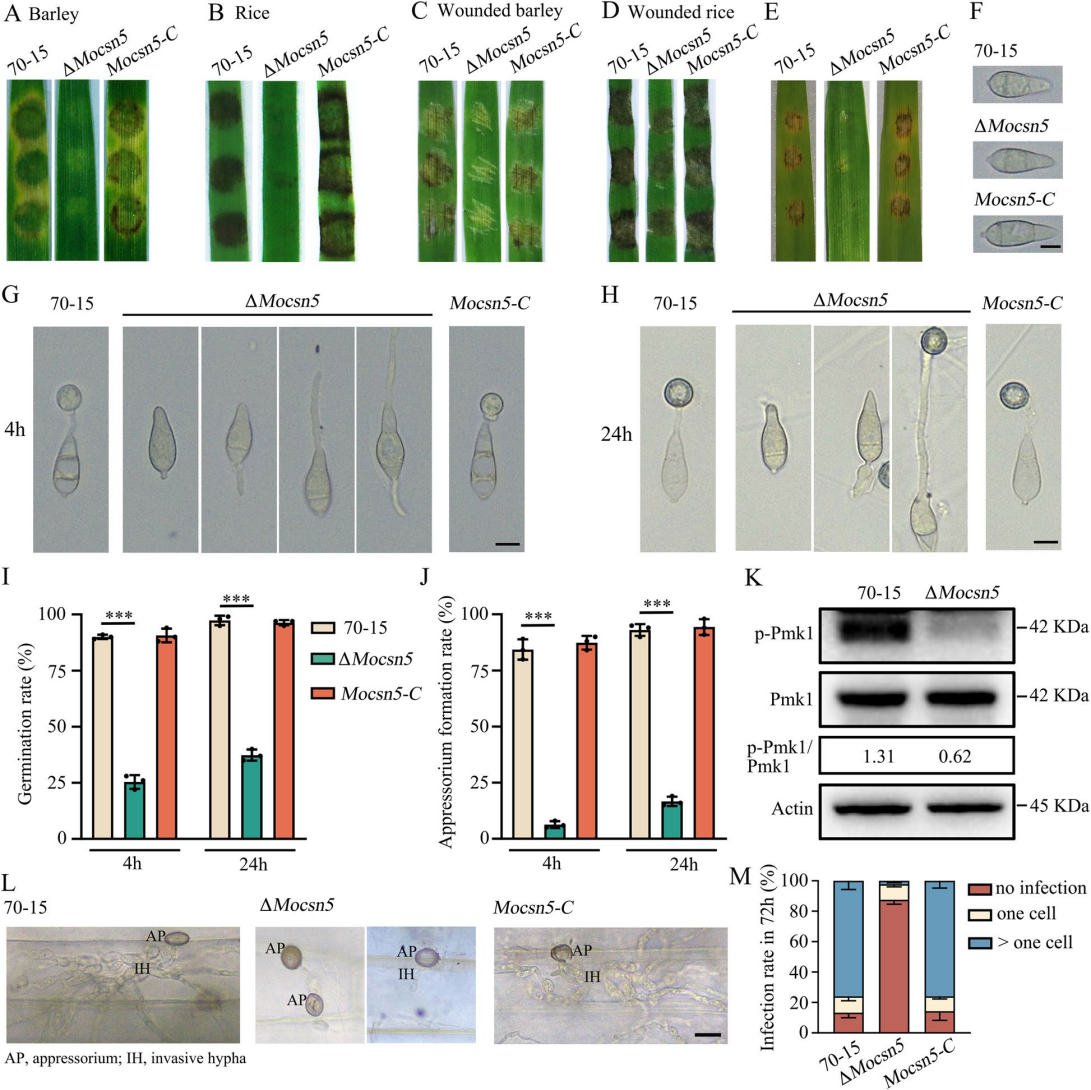
MoCsn5 is involved in conidial germination, appressorium formation, and pathogenicity in *M. oryzae.* **A** Disease spots of detached barley leaves inoculated with mycelial plugs from the 70-15, Δ*Mocsn5* and *Mocsn5-C* strains. Leaves were cultured at 25 ℃ for 4 days after inoculation. **B** Mycelial plugs from the 70-15, Δ*Mocsn5* and *Mocsn5-C* strains were inoculated on detached rice leaves. **C** Disease symptoms on wounded leaves of rice inoculated with mycelial plugs of the 70-15, Δ*Mocsn5* mutant and *Mocsn5-C* strains*.* **D** Disease symptoms on wounded leaves of barley inoculated with mycelial plugs of 70-15, Δ*Mocsn5* mutant and *Mocsn5-C* strains*.* **E** Disease spots of detached barley leaves inoculated with conidial suspensions of the 70-15, Δ*Mocsn5* and *Mocsn5-C* strains. Leaves were cultured at 25 ℃ for 4 days after inoculation. **F** Conidial morphology of 70-15, Δ*Mocsn5* and *Mocsn5-C.* Bar: 10 μm. **G** Germ tube germination assays and appressorium formation assays on hydrophobic surfaces at 4 hpi. Bar: 10 μm. **H** Germ tube germination assays and appressorium formation assays on hydrophobic surfaces at 24 hpi. Bar: 10 μm. **I** Statistical analysis of germ tube germination rates of 70-15, Δ*Mocsn5* and *Mocsn5-C* when conidia were dropped onto hydrophobic surfaces at 4 hpi and 24 hpi. The data were analyzed with GraphPad Prism 8.0. ***P < 0.001. **J** Statistical analysis of the appressorium formation rates of 70-15, Δ*Mocsn5* and *Mocsn5-C* when conidia were dropped onto hydrophobic surfaces at 4 hpi and 24 hpi. The data were analyzed using GraphPad Prism 8.0. ***P < 0.001. **K** Phosphorylation analysis of Pmk1 in 70-15 and Δ*Mocsn5.* Pmk1 phosphorylation and Pmk1 levels were detected with phospho-Pmk1 and Pmk1 antibodies, respectively. The protein actin was used as a loading control. **L** The conidial suspension (5 × 10^4^ ml^−1^) was dripped onto isolated leaves and incubated in a humid chamber at 25 °C. After 3 days, the leaves were decolorized with methanol and observed under an optical microscope. Bar: 10 μm. **M** The infection rate was quantified and statistically analyzed using GraphPad Prism 8.0 software

We then performed mycelial plug pathogenicity experiments on two susceptible hosts (barley and rice). As expected, the disease spots were smaller in the Δ*Mocsn5* mutant than in the 70-15 and complemented strains (Fig. 2A and B). In addition, we inoculated wounded rice and barley leaves with mycelial plugs of 70-15, Δ*Mocsn5* and *Mocsn5-C* for 3 days. Moreover, 70-15 and *Mocsn5-C* caused severe lesions, whereas Δ*Mocsn5* caused smaller disease lesions than 70-15 and *Mocsn5-C* (Fig. 2C and D). As the infectious weapon of rice blast fungus, conidia germinate to form appressoria with sufficient penetration turgor to infect the host and thus play a vital role in virulence. To investigate whether MoCsn5 regulates the development of appressoria in *M. oryzae*, we induced appressoria on an artificially hydrophobic surface and observed their morphology under a microscope. As shown in Fig. 2F, the conidial morphology was not affected by *MoCSN5* deletion, and typical three-celled spores remained. Compared with those of 70-15 and *Mocsn5-C*, the germ tube germination rate and appressorium formation rate of Δ*Mocsn5* were significantly decreased at 4 h postinoculation (hpi) and 24 hpi (Fig. 2G-J). At 4 hpi, the germination rate of Δ*Mocsn5* was only 25.3%, and the appressorium formation rate was only 6.3%, whereas the germination rate of 70-15 was 90.0%, and its appressorium formation rate was 84.3% (F ig. 2I and J). Although the observation time was extended to 24 h, the tube germination rate (37.3%) and appressorium formation rate (16.7%) of Δ*Mocsn5* were still significantly lower than those of 70-15 (97.3%/93%) and *Mocsn5-C* (96.3%/94.3%) (Fig. 2I and J). Pmk1-MAPK pathways have been reported to regulate appressorium formation in *M. oryzae*. The decreased phosphorylation levels of MoPmk1 in Δ*Mocsn5* indicate that MoCsn5 is involved in appressorium formation through the MAPK pathway (Fig. 2K).

Then, we tested the pathogenicity of Δ*Mocsn5* on barley leaves using conidia. As expected, only some nonexpanding small necrotic lesions were observed on the leaves inoculated with Δ*Mocsn5*, while the leaves inoculated with 70-15 and *Mocsn5-C* produced a large number of connected brown necrotic lesions (Fig. 2E). Finally, we carried out infection experiments with isolated barley leaves to further explore the progression of infection in the Δ*Mocsn5* mutant. Seventy-two hours after spore infection, the leaves were observed under a microscope. The main growth morphology of infected hyphae in leaves infected with 70-15, Δ*Mocsn5* and *Mocsn5-C* is shown in Fig. 2L. The infected hyphae of 70-15 and *Mocsn5-C* expand into multiple adjacent plant cells, whereas the appressoria of Δ*Mocsn5* are not infected, or the infected hyphae are limited to a single plant cell (Fig. 2L). At 72 hpi, nearly 86% of the infection structures were detected in the 70-15 strains, and 76% had expanded to neighboring cells. However, with Δ*Mocsn5*, only approximately 12% of the infected structures formed, and 9% were still limited to the initially infected cells (Fig. 2M). These results indicate that MoCsn5 is essential for germ tube germination, appressorium development, and pathogenicity in *M. oryzae*.

**Fig. 3:**
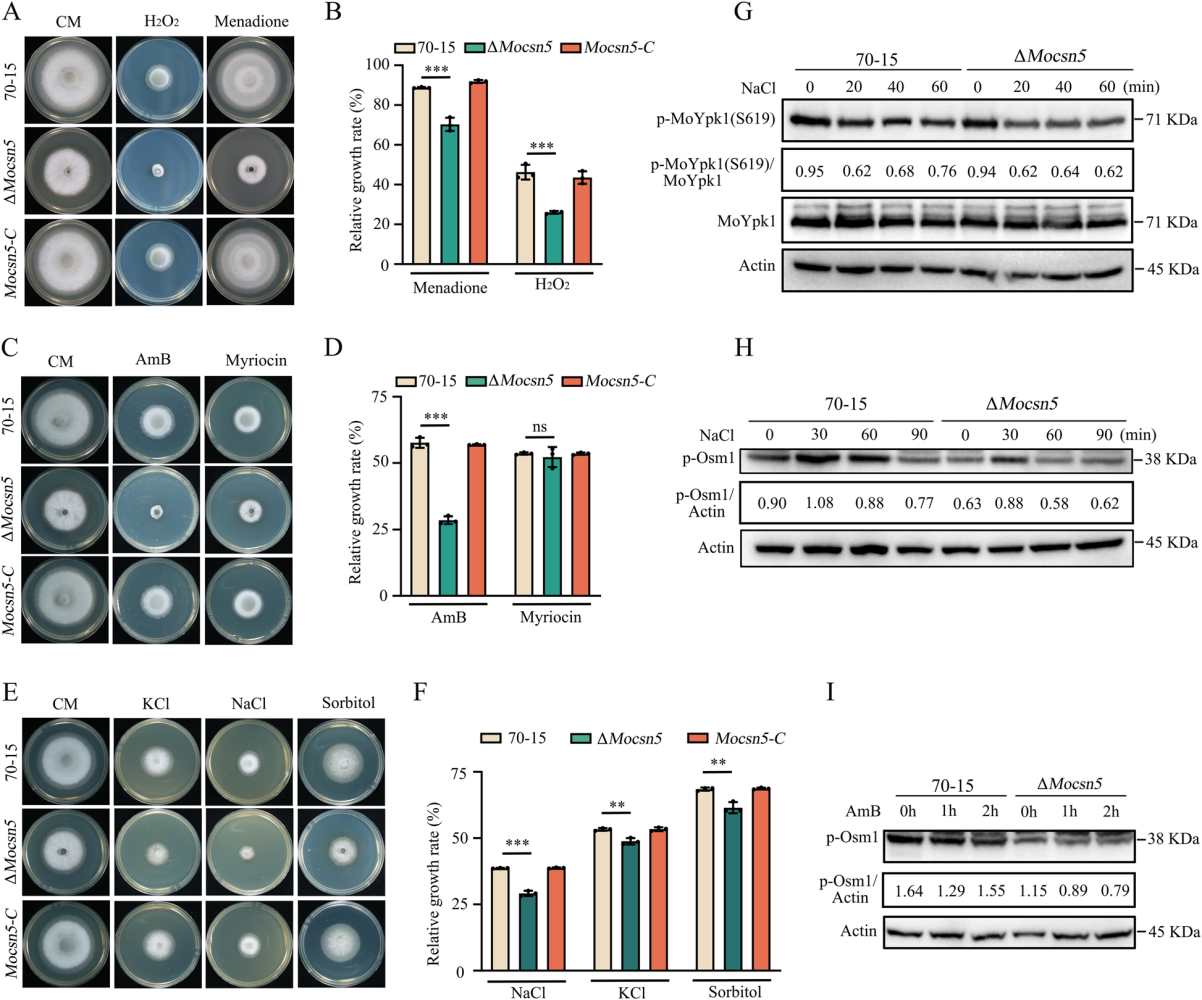
Δ*Mocsn5* is defective in the maintenance of external stresses. **A** and **B** Colony morphology and relative growth rate of 70-15, Δ*Mocsn5* and *Mocsn5-C* on CM supplemented with 2 mM hydrogen peroxide (H_2_O_2_) and 100 μM menadione (VK3). ***P < 0.001. **C** and **D** Colony morphology and relative growth rate of 70-15, Δ*Mocsn5* and *Mocsn5-C* on CM supplemented with 2 μM amphotericin B (AmB) and 1.5 μM myriocin. ***P < 0.001. **E** and **F** Colony morphology and relative growth rate of 70-15, Δ*Mocsn5* and *Mocsn5-C* on CM supplemented with 0.4 M KCl, 0.6 M NaCl, and 0.8 M sorbitol. Pictures were taken at 9 days. The data were analyzed with GraphPad Prism 8.0 software. ***P < 0.001, **P < 0.01. **(G)** Phosphorylation level of MoYpk1 in 70-15 and Δ*Mocsn5*. The strains were cultured in CM supplemented with 0.6 M NaCl before analysis. **H** Phosphorylation level of MoOsm1 in 70-15 and Δ*Mocsn5* strains. The strains were cultured in CM supplemented with 0.6 M NaCl before analysis. **I** Phosphorylation level of MoOsm1 in 70-15 and Δ*Mocsn5* strains. The strains were cultured in CM or treated with 2 μM amphotericin B before analysis

Tolerance to external stresses is critical for the survival, appressorium formation, and invasion of rice blast fungus [39–41]. We found that Δ*Mocsn5* was more sensitive to hyperosmotic stress (KCl, NaCl, and sorbitol), oxidative stress (menadione and H_2_O_2_), and amphotericin B (an antifungal agent that disrupts membrane permeability by binding to sterols on the fungal cell membrane) than 70-15 and was not sensitive to myriocin (a sphingolipid synthesis inhibitor) (Fig. 3A-F). Fungi have been reported to respond to hyperosmotic stress through two mechanisms: the Osm1-MAPK signaling pathway and the TORC2-Ypk1 signaling pathway [42–44]. We further determined the phosphorylation levels of Osm1 and Ypk1 under 0.6 M NaCl treatment. The Δ*Mocsn5* mutant and 70-15 strains showed a consistent trend in Ypk1 phosphorylation (Fig. 3G). However, the level of Osm1 phosphorylation was lower in the Δ*Mocsn5* mutant than in the 70-15 strain (Fig. 3H). The phosphorylation level of Osm1 in the Δ*Mocsn5* mutant was also significantly lower than 70-15 after treatment with 2 μM amphotericin B (Fig. 3I), which disrupts cell membrane permeability. The above-described experimental results indicate that MoCsn5 participates in the response to hyperosmotic stress by participating in the Osm1-MAPK signaling pathway.

### MoCsn5 regulates the ubiquitin‒proteasome pathway in rice blast fungus

**Fig. 4:**
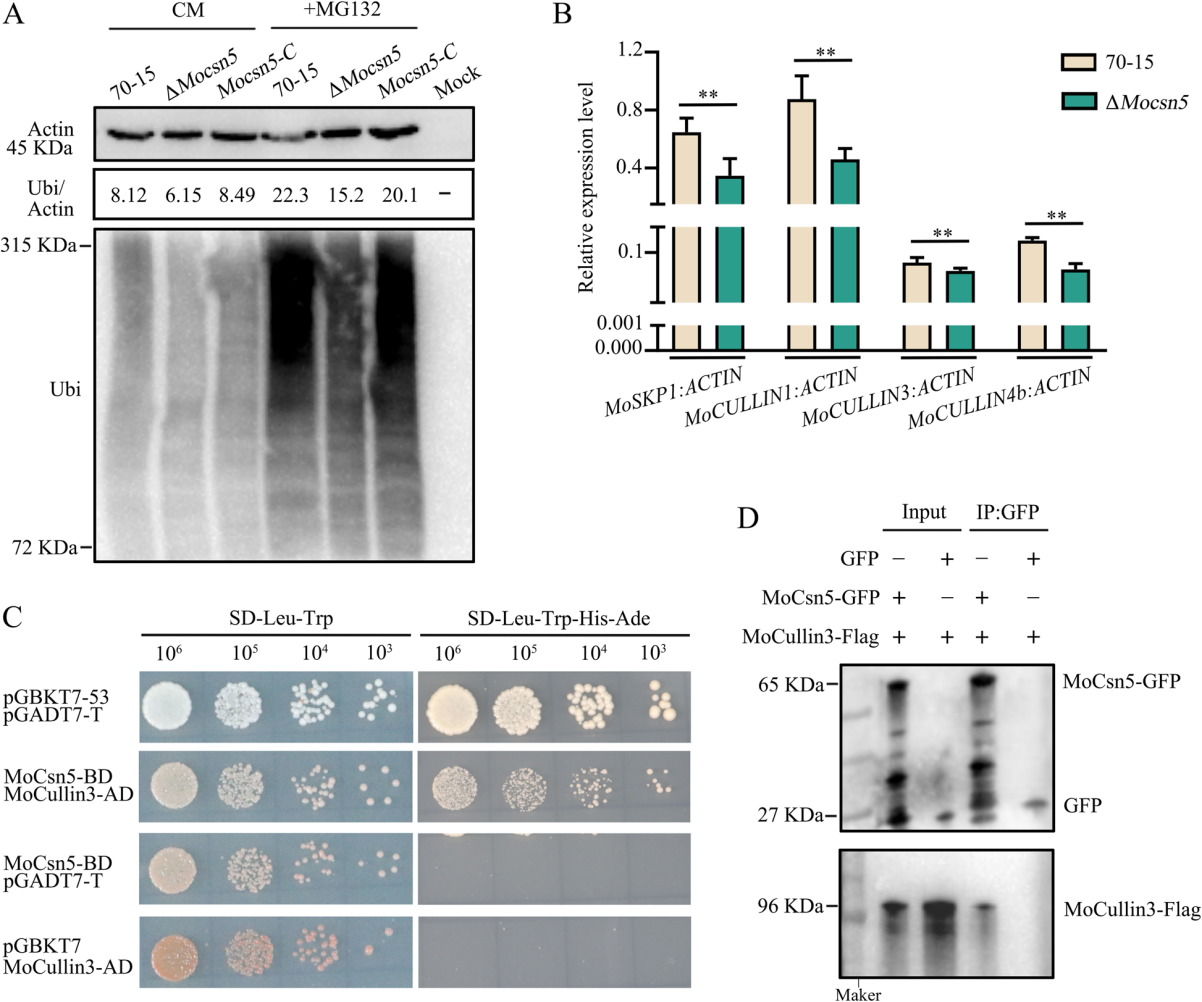
MoCsn5 regulates the ubiquitin‒proteasome pathway in rice blast fungus. **A** Ubiquitination levels of 70-15, Δ*Mocsn5* and *Mocsn5-C* at 0 h and 3 h after MG132 induction. Hyphae were grown in liquid CM for 36 h and then shifted to CM supplemented with 20 μM MG132 (a proteasome inhibitor, a tool for studying cellular degradation of the ubiquitin‒proteasome pathway) for 0 h and 3 h. **B** Transcript levels of *MoSKP1* (MGG_04978), *MoCULLIN1* (MGG_07145), *MoCULLIN3* (MGG_07731) and *MoCULLIN4b* (MGG_14763) in 70-15 and Δ*Mocsn5*. The error bars represent the standard deviations. Tukey’s test was used to determine significance. **P < 0.01. **C** The interaction between MoCsn5 and MoCullin3 was detected by yeast two-hybrid assays. pGADT7-T and pGBKT7-53 were used as positive controls. pGADT7-T and MoCsn5-BD and pGBKT7 and MoCullin3-AD served as two pairs of negative controls. **D** The relationship between MoCsn5 and MoCullin3 in vivo was examined by coimmunoprecipitation assays. The MoCullin3-Flag bands were detected following MoCsn5-GFP immunoprecipitation

Our previous experimental results showed that MoCsn5 is a subunit of the CSN complex and participates in the assembly of the CSN complex through interaction with other CSN subunits, mediating the ubiquitin‒proteasome pathway. To further investigate the role of MoCsn5 in ubiquitination, we detected the ubiquitination levels of 70-15 and ∆*Mocsn5* using an anti-ubiquitin antibody. The lower level of ubiquitination in ∆*Mocsn5* suggests that MoCsn5 promotes ubiquitination (Fig. 4A). This result was further confirmed by the recovery of the ubiquitination levels in the complemented strain *Mocsn5-C* (Fig. 4A). MG132 is a proteasome inhibitor that inhibits the degradation of ubiquitinated proteins. After induction with MG132 for 4 h, the levels of ubiquitination of 70-15 and ∆*Mocsn5* significantly increased due to the accumulation of ubiquitinated proteins (Fig. 4A). However, regardless of MG132 induction, the ubiquitination protein level of ∆*Mocsn5* was significantly lower than that of 70-15 and *Mocsn5-C*. This finding indicates that the ability of MoCsn5 to promote ubiquitination in rice blast fungus was sustained.

To explore the mechanism by which MoCsn5 promotes ubiquitination, we examined the mRNA levels of ubiquitin-associated proteins in *M. oryzae*. The qPCR results showed that the expression levels of *MoCULLIN1*, *MoCULLIN3*, *MoCULLIN4b,* and *MoSKP1* were significantly decreased by the elimination of MoCsn5 (Fig. 4B). This finding explains the reduced level of ubiquitination in ∆*Mocsn5*. In addition, the results of the interaction experiment indicated that MoCsn5 directly interacts with MoCullin3 in vivo and in vitro to regulate CRLs (Fig. 4C and D). In summary, we conclude that MoCsn5 promotes the ubiquitination of *M. oryzae* by regulating the components of CRLs.

### MoCsn5 negatively regulates autophagy

**Fig. 5:**
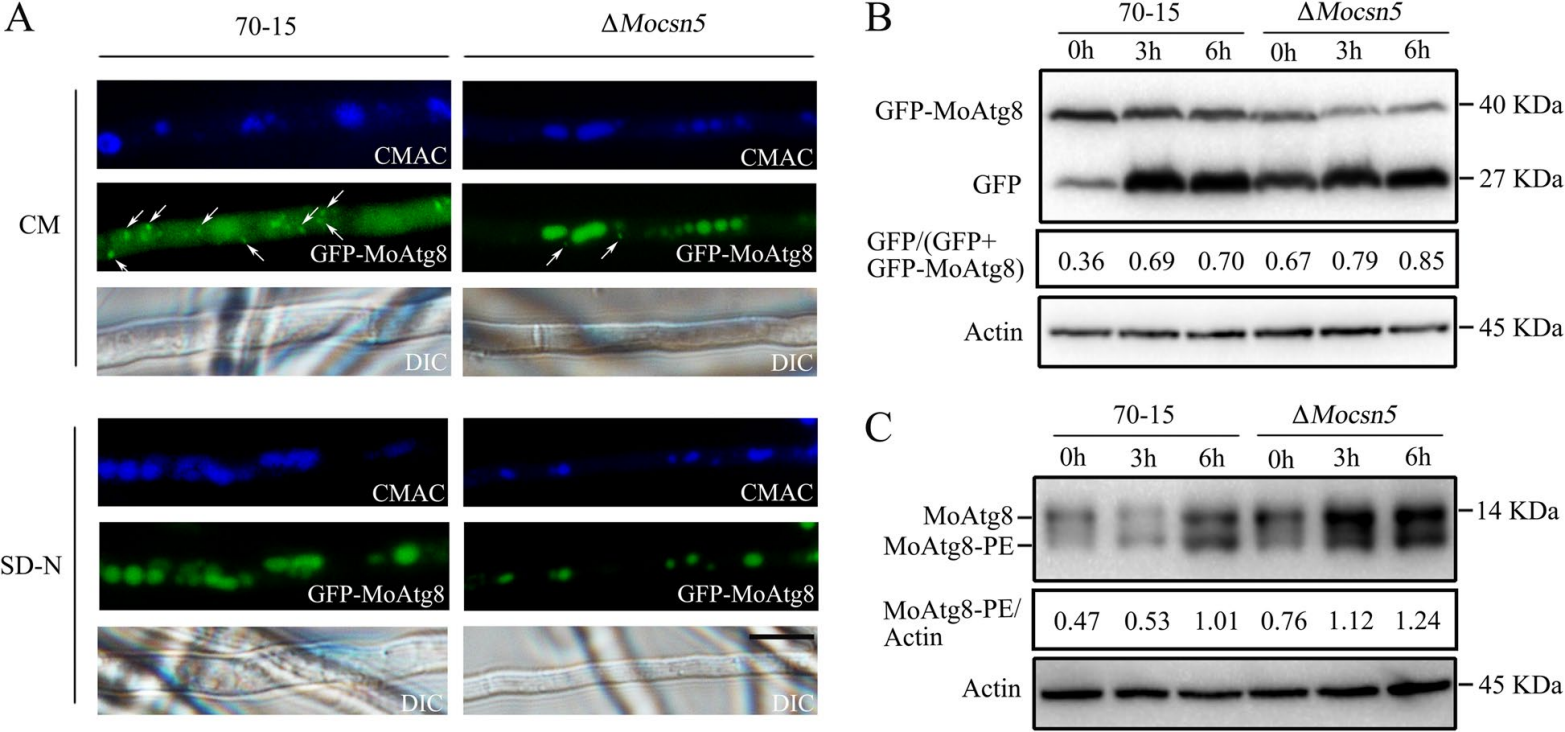
MoCsn5 negatively regulates autophagy. **A** Subcellular localization of GFP-MoAtg8 in 70-15 and Δ*Mocsn5* under nutrient and starvation conditions. Bar: 10 μm. Mycelia were stained with CMAC to label the vacuoles. **B** Autophagic flux analysis of GFP-MoAtg8 in 70-15 and Δ*Mocsn5*. Total GFP-MoAtg8 and free GFP were detected by western blot. The gray values of the protein bands were analyzed with ImageJ software. The degradation rate was expressed as [GFP/(GFP + GFP-MoAtg8)]. The protein content of actin was used as a control. **C** Analysis of MoAtg8/MoAtg8-PE turnover in 70-15 and Δ*Mocsn5*. The ratio of MoAtg8-PE to actin was calculated to assess the rate of MoAtg8 lipidation to yield MoAtg8-PE

The ubiquitin‒proteasome system and autophagy‒lysosome pathway, which are responsible for the degradation of cellular proteins, are crucial for various cellular processes, such as the growth and development of organisms [8]. Due to the important function of CSN in the ubiquitin‒proteasome system and the interaction between MoCsn5 and autophagy-related proteins, we examined the autophagy levels in 70-15 and Δ*Mocsn5*. The GFP-MoAtg8 fusion protein was used to determine whether there was a connection between MoCsn5 and autophagy. During the fusion of the autophagosome with the lysosome, MoAtg8 on the inner membrane of the autophagosome is delivered to the vacuole for degradation, where it produces free GFP (in the vacuole); therefore, the [(GFP)/(GFP + GFP-MoAtg8)] ratio is widely used to indicate the level of autophagy. First, the subcellular localization of GFP-MoAtg8 in 70-15 and Δ*Mocsn5* was observed. As shown in Fig. 5A, under nutritional conditions, GFP-MoAtg8 in 70-15 was positioned around the vacuole in a spot-like manner, whereas the vacuoles in Δ*Mocsn5* appeared bright and uniform green without obvious dot-like localization. After 3 h of starvation induction, the vacuoles in the 70-15 strain were uniformly green overall, and the same trend was observed in the Δ*Mocsn5* strain (Fig. 5A). For further confirmation, a western blot analysis was conducted to detect full-length GFP-MoAtg8 and free GFP in 70–15 and Δ*Mocsn5*. As shown in Fig. 5B, the autophagic flux in the Δ*Mocsn5* mutant was greater than that in the wild type.

Next, we evaluated the turnover rate of endogenous MoAtg8/MoAtg8-phosphatidylethanolamine (MoAtg8-PE). The conversion of microtubule-associated protein 1 light chain 3 (LC3-I, Atg8 homology) to a lapidated form (LC3-II, Atg8-PE) is a committed step in autophagosome formation. Accordingly, the LC3-II protein level is commonly used as a marker of the autophagosome number [45]. As shown in Fig. 5C, under nutritional conditions, the MoAtg8 band was stronger, whereas the MoAtg8-PE band obtained with both 70-15 and Δ*Mocsn5* was weaker. After 3 and 6 h of starvation induction, the MoAtg8-PE and MoAtg8 band strengths were enhanced in both the 70-15 and Δ*Mocsn5* strains, indicating that starvation stimulated the formation of autophagosomes (Fig. 5C). However, it is worth noting that compared to 70-15, Δ*Mocsn5* has stronger MoAtg8-PE and MoAtg8 bands. These results indicated that the total conversion of MoAtg8 to MoAtg8-PE was increased in the Δ*Mocsn5* strain, and the number of autophagosomes and autophagic flux were greater in the Δ*Mocsn*5 strain than in the 70-15 strain.

Because MoCsn5 and MoCsn6 are involved in the regulation of autophagy and other subunits also interact with autophagy-related proteins, we are very interested in whether other subunits of the CSN complex generally have regulatory functions in autophagy. Therefore, we transfected GFP-MoAtg8 into the corresponding mutants to detect the autophagy levels. Several transfection experiments were performed, and GFP-MoAtg8 was not transfected into the Δ*Mocsn2* and Δ*Mocsn3* mutants. Compared with 70-15, the Δ*Mocsn1*, Δ*Mocsn4*, and Δ*Mocsn7a* strains all showed increased levels of autophagy (Figure S3B and C). Thus, other subunits of the CSN complex also regulate autophagy in *M. oryzae*.

### MoCsn5 inhibits autophagy by promoting K48-ubiquitination of MoAtg6

**Fig. 6:**
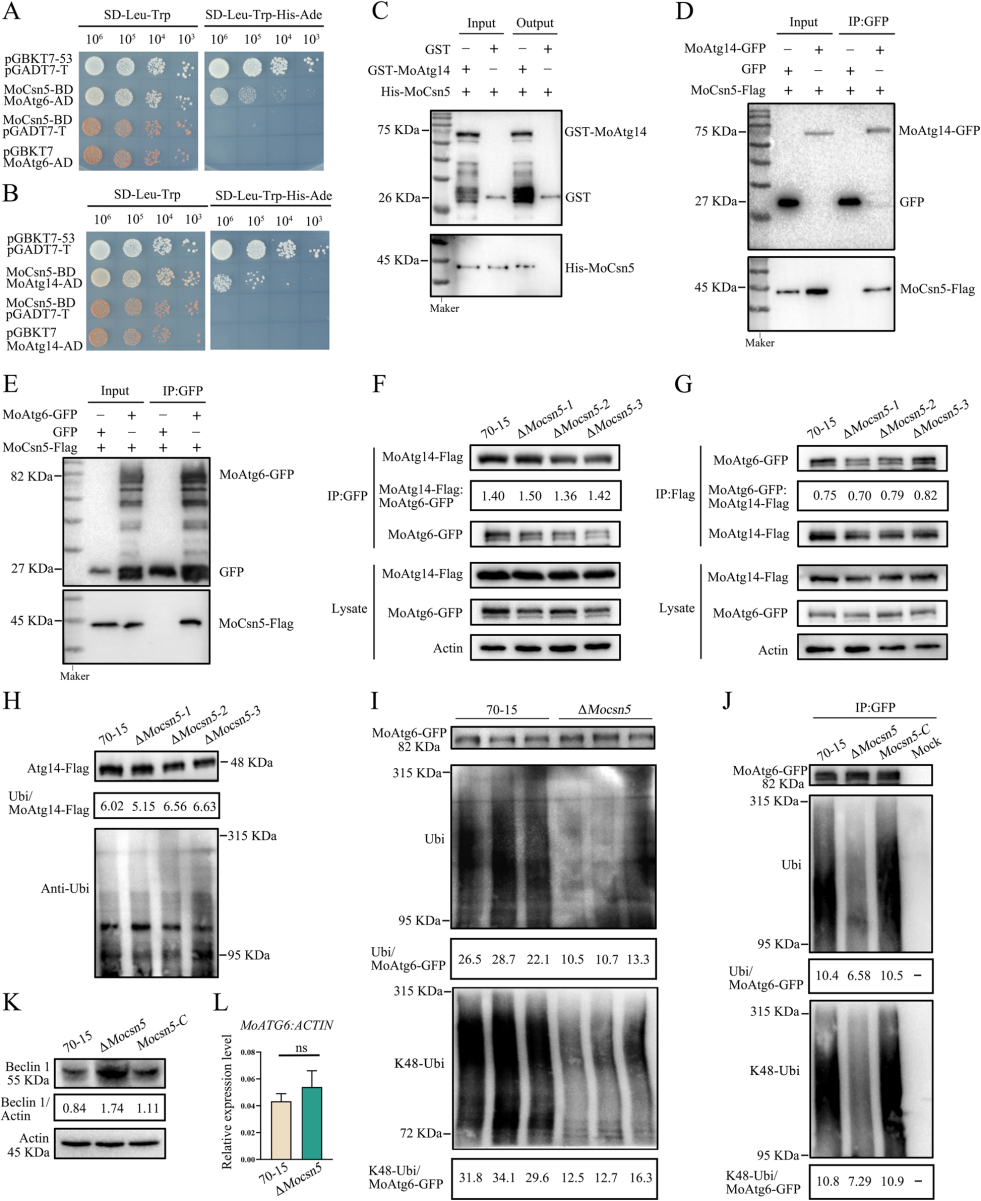
MoCsn5 inhibits autophagy by promoting the K48-ubiquitination of MoAtg6. **A** The interaction between MoCsn5 and MoAtg6 was detected by yeast two-hybrid assays. pGADT7-T and pGBKT7-53 were used as positive controls. pGADT7-T and MoCsn5-BD and pGBKT7 and MoAtg6-AD served as two pairs of negative controls. **B** The interaction between MoCsn5 and MoAtg14 was detected by yeast two-hybrid assays. pGADT7-T and pGBKT7-53 were used as positive controls, and pGADT7-T and MoCsn5-BD and pGBKT7 and MoAtg14-AD were used as two pairs of negative controls. **C** Pulldown assays to detect the interaction between MoCsn5 and MoAtg14 in vitro. GST-MoAtg14 and His-MoCn5, empty GST, and His-MoCsn5 were incubated sequentially with glutathione agarose gel beads with GST labels for 2 h. The final eluents were detected by western blot assays. **D** The relationship between MoCsn5 and MoAtg14 was examined by in vivo coimmunoprecipitation assays. The MoCsn5-Flag bands were detected following MoAtg14-GFP immunoprecipitation. **E** The relationship between MoCsn5 and MoAtg6 was examined by in vivo coimmunoprecipitation assays. The MoCsn5-Flag bands were detected following MoAtg6-GFP immunoprecipitation. **F** Interaction intensity of MoAtg6 and MoAtg14 in 70-15 and Δ*Mocsn5.* MoCsn5 knockout was performed in 70-15 strain expressing MoAtg6-GFP and MoAtg14-Flag to ensure consistent MoAtg6 and MoAtg14 contents. The strains were lysed and immunoprecipitated with anti-GFP beads and then subjected to immunoblotting with anti-GFP and anti-Flag antibodies. The formula MoAtg14-Flag/MoAtg6-GFP was used to calculate the interaction intensity of MoAtg6 and MoAtg14 in the different strains. **G** Interaction intensity of MoAtg6 and MoAtg14 in 70-15 and Δ*Mocsn5.* MoCsn5 knockout was performed in 70-15 strain expressing MoAtg6-GFP and MoAtg14-Flag to ensure consistent MoAtg6 and MoAtg14 contents. The strains were lysed and immunoprecipitated with anti-Flag beads and then subjected to immunoblotting with anti-GFP and anti-Flag antibodies. The formula MoAtg6-GFP/MoAtg14-Flag was used to calculate the interaction intensity of MoAtg6 and MoAtg14 in the different strains. **H** Ubiquitination levels of MoAtg14 in 70-15 and Δ*Mocsn5.* Gene knockout of *MoCSN5* was performed in 70-15 expressing MoAtg14-Flag to ensure consistent transcript levels of *MoATG14*. The strain was immunoprecipitated with anti-Flag beads after lysis, and a western blot analysis was then performed with an anti-Flag antibody and an anti-ubiquitin antibody. The formula ubiquitin/MoAtg14-Flag was used to calculate the ubiquitination levels of MoAtg14-Flag. **I** Deletion of *MoCSN5* inhibited the ubiquitination and K48-ubiquitination of MoAtg6. Gene knockout of *MoCSN5* was performed in 70-15 expressing MoAtg6-GFP to ensure consistent transcript levels of *MoATG6*. The strain was immunoprecipitated with anti-GFP beads after lysis, and a western blot analysis was then performed with anti-GFP antibody, anti-ubiquitin antibody, and anti-K48 antibody. The ubiquitination and K48-ubiquitination levels of MoAtg6-GFP were calculated as ubiquitin/MoAtg6-GFP and K48-ubiquitin/MoAtg6-GFP, respectively. **J** Ubiquitination levels and K48-ubiquitination levels of MoAtg6 in 70-15, Δ*Mocsn5* and *Mocsn5-C*. **K** The protein levels of MoAtg6 in the 70-15, Δ*Mocsn5* and *Mocsn5-C* strains were measured with an anti-Beclin1 antibody. The strains were cultured in liquid CM at 25 ℃ for 36 h. The protein content of actin was used as a control. **L** Transcript levels of *MoATG6* in 70-15 and Δ*Mocsn5*. The error bars represent the standard deviations

Figure 5 shows increased autophagosome formation and accelerated autophagosome transport in Δ*Mocsn5*. Yeast two-hybrid and pull-down assays revealed that MoCsn5 interacts with the autophagy-related proteins MoAtg6 and MoAtg14 in vitro (Fig. 6A-C). Co-IP experiments also confirmed that MoCsn5 interacts with MoAtg6 and MoAtg14 in vivo (Fig. 6D and E). These results indicate that Csn5 may participate in the autophagy regulation pathway in *M. oryzae* by regulating autophagy-related proteins.

The PI3KC3-I complex is formed by Atg6/Vps30/Beclin1, Vps34, Vps15, and Atg14 at the phagophore assembly site (PAS) and is essential for proper PAS targeting of other autophagy-related proteins, such as the Atg8, Atg18, and Atg12–Atg5-Atg16 complex [46, 47]. The localization of PI3KC3-I to the PAS is largely dependent on the binding of Atg6 to Atg14 [48]. To investigate the effect of ubiquitination on the PI3KC3-I complex, we examined the ability of Atg14 to target Atg6. Interaction strength tests showed that the amount of MoAtg14 bound to MoAtg6 in Δ*Mocsn5* was comparable to that bound in 70-15. MoCsn5 did not affect the ability of MoAtg14 to target MoAtg6 to the PAS (Fig. 6F and G). Subsequent analyses revealed that the ubiquitination level of MoAtg14 in Δ*Mocsn5* was unaffected but that the ubiquitination level of MoAtg6 was lower in Δ*Mocsn5* than in 70-15, suggesting that deficient ubiquitination modulates the level of MoAtg6 (Fig. 6H and I).

Atg6 is a core protein in the autophagy pathway, and previous studies have shown a positive correlation between its protein expression and autophagic flux [49]. Subsequent experiments showed that the increased MoAtg6 content in Δ*Mocsn5* resulted in increased autophagy activity (Fig. 6K). However, the mRNA levels of *MoATG6* were not significantly different between Δ*Mocsn5* and 70-15 (Fig. 6L). These results suggested that the increased MoAtg6 content in Δ*Mocsn5* was caused by decreased degradation rather than increased expression. Different types of anti-ubiquitin antibodies were used in western blotting assays, and we ultimately detected a reduced K48-ubiquitination level of MoAtg6 in Δ*Mocsn5* (Fig. 6I). Measurements of the ubiquitination and K48-ubiquitination levels of MoAtg6 in 70-15, Δ*Mocsn5*, and *Mocsn5-C* reconfirmed that the knockout of *MoCSN5* decreased the K48-ubiquitination level of MoAtg6 (Fig. 6J), which means that MoCsn5 promotes the K48-ubiquitination of MoAtg6 in *M. oryzae*.

Previous studies have shown that the K48-ubiquitination of Beclin1 (a homologous protein of Atg6) is a negative pathway for autophagy regulation [50, 51]. K48-ubiquitination promotes proteasome-dependent Beclin1 degradation, thereby effectively downregulating autophagy. Our research indicated that MoCsn5 promotes the K48-ubiquitination of MoAtg6, which promotes MoAtg6 degradation and thus downregulates autophagy in rice blast fungus.

### MoCsn5 inhibits autophagy by suppressing the K48-ubiquitination of MoTor

**Fig. 7:**
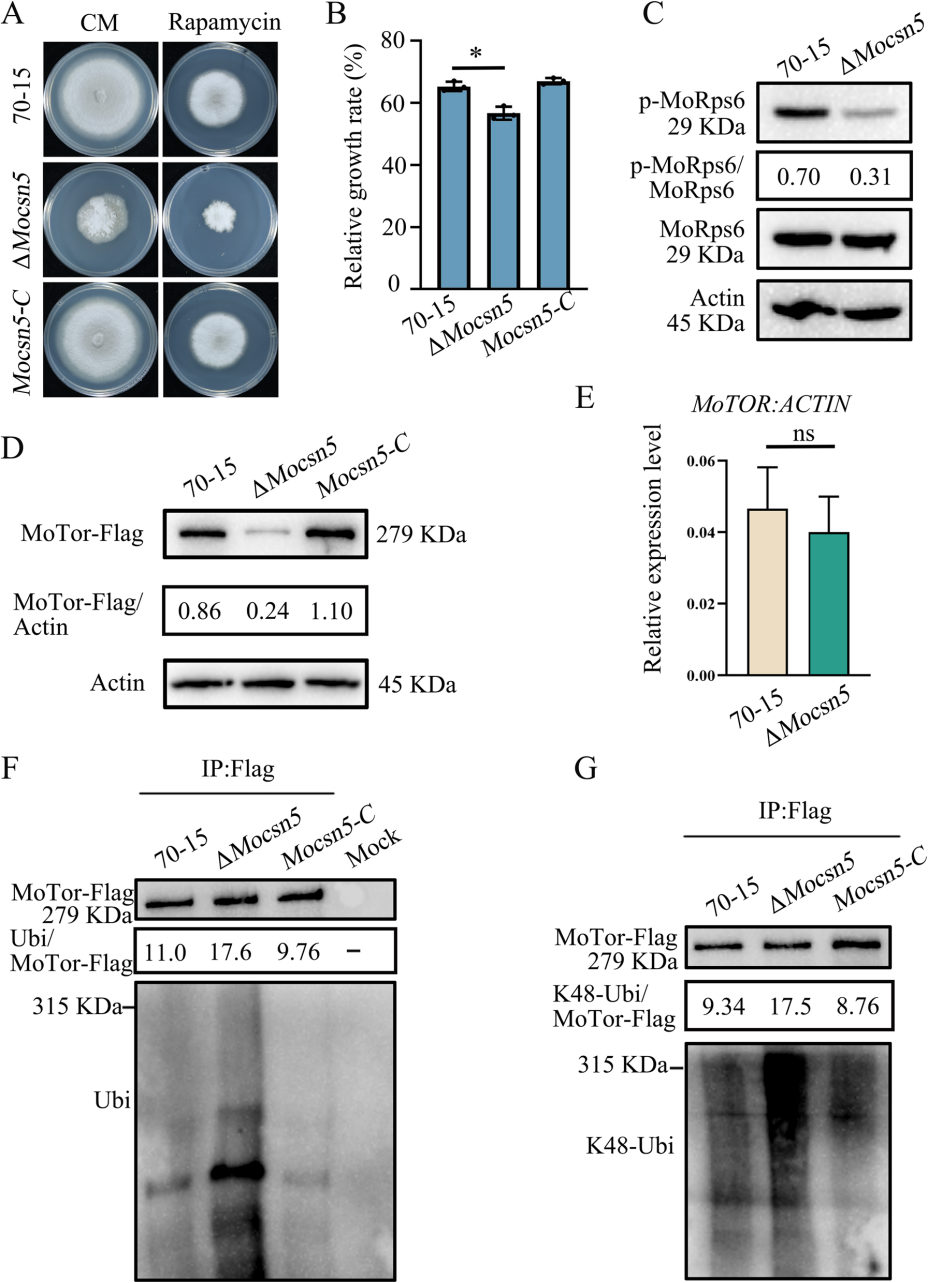
MoCsn5 inhibits autophagy by suppressing the K48-ubiquitination of MoTor. **A** and **B** Colony morphology and relative growth rate of 70-15, Δ*Mocsn5* and *Mocsn5-C* on CM supplemented with 100 ng/mL rapamycin for 9 days. *p < 0.05. **C** The phosphorylation levels of MoRps6 in the 70-15 and Δ*Mocsn5* strains were detected by anti-phospho-Rps6 and anti-Rps6 antibodies. The phosphorylation levels of MoRps6 were calculated as phospho-MoRps6/MoRps6. The protein content of actin was used as a control. **D** Protein levels of MoTor in 70-15, Δ*Mocsn5* and *Mocsn5-C*. The strains were cultured in liquid CM at 25 ℃ for 36 h. The protein content of actin was used as a control. Gene knockout and complementation of *MoCSN5* were performed in 70-15 expressing MoTor-Flag to ensure consistent transcript levels of *MoTor*. **E** Transcript levels of *MoTor* in 70-15 and Δ*Mocsn5*. The error bars represent the standard deviations. **F** Ubiquitination levels of MoTor in 70-15, Δ*Mocsn5* and *Mocsn5-C*. After lysis, the strains were immunoprecipitated with anti-Flag beads, and a western blot analysis was subsequently performed with an anti-Flag antibody and an anti-ubiquitin antibody. The level of ubiquitinated MoTor was calculated as ubiquitin/MoTor-Flag. **G** K48-ubiquitination levels of MoTor in 70-15, Δ*Mocsn5* and *Mocsn5-C*. After lysis, the strains were immunoprecipitated with anti-Flag beads, and a western blot analysis was subsequently performed with an anti-Flag antibody and an anti-K48-ubiquitin antibody. The K48-ubiquitination levels of MoTor were calculated as K48-ubiquitin/MoTor-Flag

Autophagosome formation is regulated by the TORC1 complex, and active TOR signaling inhibits autophagy through the phosphorylation of Atg proteins [52, 53]. To test whether MoCsn5 regulates MoTor activity, we used rapamycin to validate the relationship between MoCsn5 and MoTor kinase. Compared with 70-15 and the complemented strain *Mocsn5-C*, the Δ*Mocsn5* mutant was more sensitive to rapamycin on CM at 25 °C for 9 days (Fig. 7A and B). This finding suggested that the activity of the TORC1 complex in Δ*Mocsn5* was altered. To verify the regulatory effect of MoCsn5 on MoTor activity, we examined the phosphorylation of the TORC1 activity marker MoRps6. Compared with those in the 70-15 strain, the phosphorylation levels of MoRps6 in the Δ*Mocsn5* mutant strain were significantly lower after treatment with rapamycin, indicating reduced TORC1 complex activity (Fig. 7C).

Our results showed that the reduction in the MoTor content in the Δ*Mocsn5* mutant induced a decrease in TORC1 complex activity (Fig. 7D), which in turn led to increased autophagy. However, no significant difference in the mRNA levels of *MoTOR* were found between the Δ*Mocsn5* and 70-15 strains (Fig. 7E). These results suggest that the decrease in the MoTor content in Δ*Mocsn5* is due to enhanced degradation rather than decreased expression.

Previous studies have shown that the protein content of Tor is regulated by ubiquitination [54]. The ubiquitination level of MoTor in Δ*Mocsn5* was significantly greater than that in the WT 70-15 (Fig. 7F), suggesting that MoCsn5-mediated ubiquitination regulates the protein level of MoTor. Measurements of the ubiquitination and K48-ubiquitination levels of MoTor in 70-15, Δ*Mocsn5*, and the complemented strain *Mocsn5-C* also confirmed that the knockout of *MoCSN5* increased the K48-ubiquitination level of MoTor (Fig. 7G). These results showed that MoCsn5 stabilized the MoTor content through specific K48-ubiquitination, thereby inhibiting autophagy.

## Discussion

Autophagy is an ancient biological process that maintains cell homeostasis and is facilitated by numerous biological stimuli and environmental pressures [33, 55]. Although autophagy is closely related to the pathogenicity of phytopathogenic fungi [7], the specific regulatory mechanisms involved remain to be explored. During this process, numerous autophagy-related proteins that signal the autophagy machinery have been identified. Among them, Atg6/Beclin1/Vps30 is arguably the most studied molecule and is crucial for processes such as the autophagy, inflammation, tumors, and pathogenicity of pathogenic fungi [56–58]. As one of the key initiators of autophagy, Atg6 binds to PtdIns3K (class III phosphatidylinositol 3-kinase), thereby mediating the biogenesis and dynamics of subcellular membranes involved in autophagy [58, 59]. In this study, we identified a novel regulator of MoAtg6, MoCsn5, which is also a new autophagy inhibitor. Our data indicated that to inhibit autophagy, Csn5 inhibits K48-ubiquitination by interacting with Atg6, which is crucial for Atg6 degradation, thereby reducing Atg6 protein expression and inhibiting autophagy.

The COP9 signalosome is an evolutionarily highly conserved multifunctional complex that controls the cell cycle, signal transduction, circadian rhythm, embryonic development, autophagy, and other biological functions in mammals, plants, and fungi [60–62]. In previous studies, the regulatory function of CSN subunits in autophagy has been controversial. The impaired fusion between autophagosomes and lysosomes in CR-CSN8^KO^ mice (in which Csn8 was knocked out) is the first line of evidence showing that CSN regulates autophagy [63, 64]. Subsequent studies showed that Csn8 plays an important role in autophagosome maturation. In mammals, Csn3 not only is necessary for autophagosome formation but also promotes autophagosome maturation. Niu et al. showed that Csn3 deficiency inhibits the LC3B-I-to-LC3B-II transformation and promotes the mTOR pathway, which inhibits autophagy in mammalian osteosarcoma [22]. In rice blast fungus, Csn6 deletion leads to enhanced autophagy, which is manifested by an increased number of autophagosomes and an accelerated autophagy rate [26]. In this study, we demonstrated that Csn5 inhibits autophagy in *M. oryzae* by promoting ubiquitin-dependent Atg6 protein degradation and stabilizing the TOR pathway in autophagosome formation. This difference may be due to the fact that different CSN subunits do not play the same roles in autophagy or to species differences. At present, the role of CSN in autophagy regulation is emerging, but the specific functions of other subunits in autophagy regulation still need to be discovered.

The expression and degradation of proteins are crucial for various aspects of cell development. In eukaryotes, the autophagy‒lysosomal pathway and the ubiquitin‒proteasome system are two highly conserved protein clearance pathways [8, 9, 65, 66]. Although these two pathways are often discussed as separate functional pathways, in recent years, there has been increasing evidence of crosstalk between the ubiquitin‒proteasome system and the autophagy‒lysosomal pathway. Zhang et al. demonstrated that Cand2-regulated ubiquitination and autophagy are essential for pathogenic fungal pathogenicity [67]. We found that MoCsn5 not only regulates the overall ubiquitination level of organisms but also mediates autophagy by directly targeting the ubiquitination of Atg6 and Tor. Using the Csn5 subunit of the key regulatory complex CSN in ubiquitin as an entry point, we revealed a novel mechanism by which ubiquitination regulates autophagy and thus affects pathogenicity in rice blast fungus.

The pathogenicity of pathogenic fungi is not regulated by a single pathway but by the joint regulation of multiple pathways. The Osm1-MAPK pathway modulates the response of pathogenic fungi to hyperosmotic stress [44]. The Pmk1-MAPK pathway is necessary for appressorium-mediated infection of *M. oryzae* [68, 69]*.* Although starvation induces autophagy, autophagy is maintained at a basic level even under normal growth conditions, thus controlling the metabolism, development and proliferation of eukaryotes through a comprehensive regulatory pathway [7]. We found that the absence of MoCsn5 downregulated the phosphorylation of MoOsm1 and MoPmk1, reducing the resistance to hyperosmotic stress and the invasion ability of the appressorium in *M. oryzae*. Studies in mammals have shown that the MAPK signaling pathways (ERK1/2, ERK5, p38, and JNK1/2/3 signaling pathways) are regulated by E3 ubiquitin ligases in cancer [70]. In rectal cancer, Usp14 (ubiquitin-specific peptidase 14) deubiquitinates and stabilizes JNK, thereby promoting MAPK/JNK signaling cascade activation [71]. In fission yeast, the autophagy-related protein Atg1 acts in the Pmk1-MAPK pathway. Pmk1 signaling is suppressed in the Δ*atg1* strain, and the growth defects caused by overexpression of Pck2, an upstream activator of Pmk1-MAPK, are mitigated by the deletion of *ATG1* [72]. We hypothesize that the pleiotropic defects in the growth, development, and pathogenicity of Δ*Mocsn5* are the result of the dysregulation of different signaling pathways. MoCsn5 may not only regulate autophagy through ubiquitination but also mediate signaling pathways associated with rice blast fungus infection, such as the MoOsm1-MAPK and MoPmk1-MAPK pathways, via ubiquitination and/or autophagy, but the specific regulatory mechanisms involved remain to be investigated, and we believe that these mechanisms will be a very interesting subject.

**Fig. 8:**
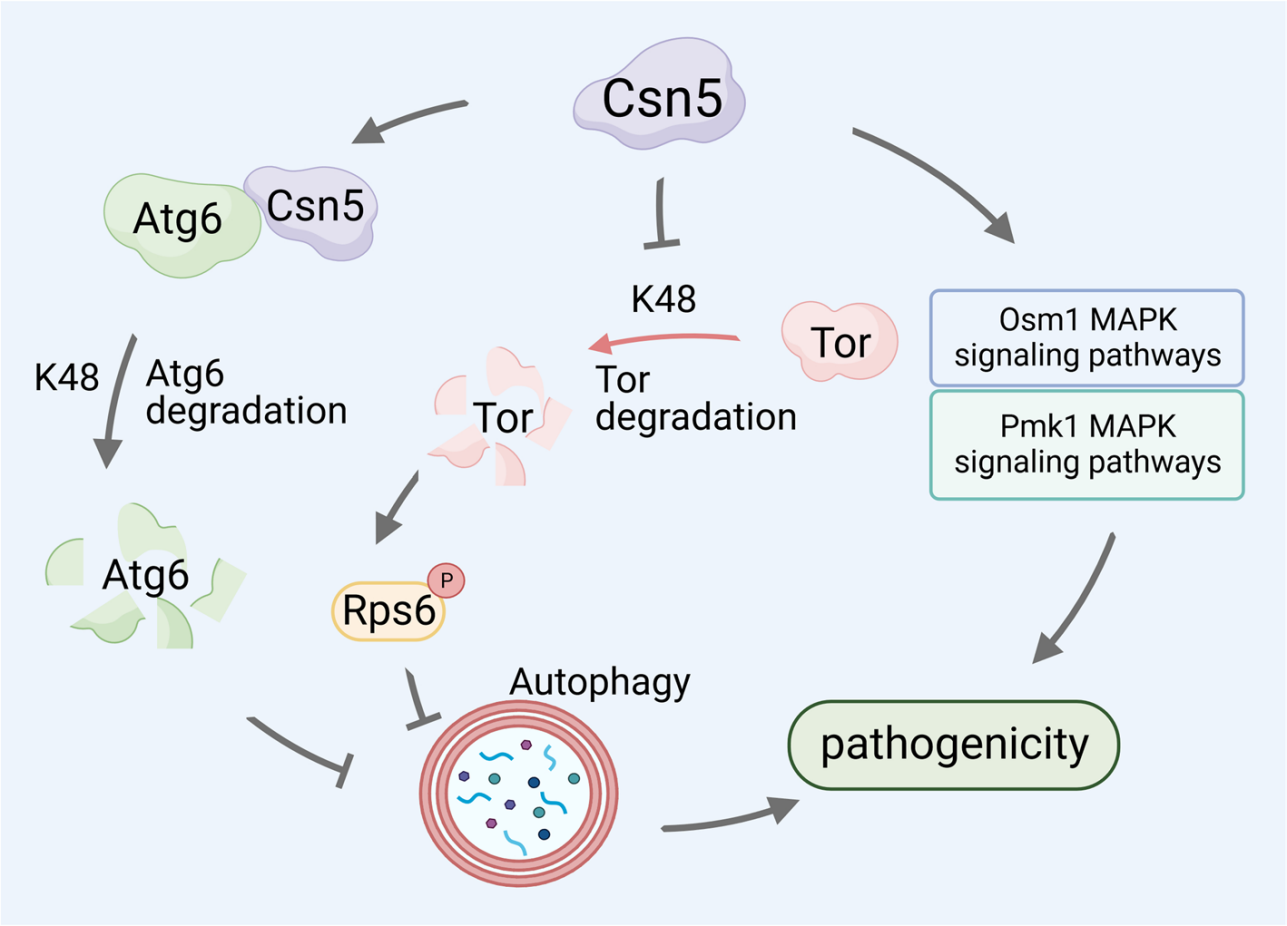
A proposed regulatory model for the function of MoCsn5 in *M. oryzae.* As a novel autophagy inhibitor, MoCsn5 participates in the regulation of the pathogenicity of *M. oryzae*. MoCsn5 inhibits autophagy by regulating the ubiquitination of the autophagy-related proteins MoAtg6 and MoTor, inhibiting the degradation of MoTor and promoting the degradation of MoAtg6. In addition, MoCsn5 affects the phosphorylation of MoPmk1 and MoOsm1, thereby regulating the infection and hypertonic stress response of *M. oryzae*

Taken together, our findings revealed new associations between Csn5 and autophagy, ubiquitination, and pathogenicity in rice blast fungus (Fig. 8). MoCsn5 regulates the protein expression levels of MoAtg6 and MoTor through ubiquitination, thereby inhibiting autophagy. Our results also indicate that MoCsn5 is essential for fungal development, germ tube germination, and appressorium formation in *M. oryzae*. Although meaningful progress has been made, there are still many questions waiting to be answered. First, are the effects of different CSN subunits on autophagy in different species due to the species or functional specificity of the subunits? Second, does Csn5 regulate the MoOsm1-MAPK and MoPmk1-MAPK pathways through ubiquitination or independently of ubiquitination? Third, as a multifunctional complex, does the CSN complex or its subunits regulate autophagy through other pathways? The regulatory function of the CSN is much more complex than expected. The resolution of these issues could deepen our understanding of CSN-regulated autophagy, which is important for the development of new therapeutic strategies for pathogenic fungi.

## Materials and methods

### Strains, growth conditions, and quantitative RT‒PCR

The WT strain of *M. oryzae*, 70-15, was used in this study. All fungal strains were cultured on CM at 25 °C under a 16–18 h light-dark cycle for 8–10 days. For different stress tests, 100 µΜ menadione (VK3), 2 mM hydrogen peroxide (H_2_O_2_), 2 µΜ amphotericin B, 1.5 µΜ myriocin, 0.4 M KCl, 0.6 M NaCl and 0.8 M sorbitol were added to solid CM agar plates. The relative growth rates were calculated using the following formula: (the diameter of the strain treated with chemicals)/(the diameter of the untreated strain). Data analysis was performed using ImageJ 1.51j8 and GraphPad Prism 8.0 software. All assays were repeated three times.

To detect the expression of target genes, the 70-15 and Δ*Mocsn5* strains were cultured in liquid CM for 40 h at 150 rpm at 25 °C. Total RNA was extracted by the TRIzol method and reverse transcribed into cDNA using a reverse transcription kit (TaKaRa, Japan). qRT-PCR was performed using a SYBR Premix Ex Taq (Tli RNaseH Plus) kit (TaKaRa, Japan). All assays were repeated three times. Data analysis was performed using GraphPad Prism 8.0 software. All primers were designed online by Integrated DNA Technologies (https://sg.idtdna.com/pages) and are listed in Table S1.

### Gene knockout and complementation assays

In this study, a homologous recombination strategy based on the *Agrobacterium tumefaciens-mediated* transformation (ATMT) strategy was used for gene deletion [73]. The knockout vector was designed using pKO3A by Lu et al. as described previously [74]. The 1.5-kb upstream fragment (*UF*) and downstream fragment (*DF*) of the targeted gene were amplified by PCR with the specific primers *CSN*-up/down (F) and *CSN*-up/down (R). The pKO3A vector was digested with *Xba*I and *Hin*dIII. Then, the hygromycin resistance gene (*HPH*) fragment, *UF*, and *DF* were fused to the linearized vector by ligase (Vazyme, P505-d1). The recombination cassette was transferred into 70-15 by ATMT, and the positive transformants with hygromycin resistance were selected using 200 μg/mL hygromycin B (Sangon Biotech, A600230-0001). The long fragment (*LF*) and short fragment (*SF*) in mutants were further validated using the primers *CSN*-Long-F/Long-HPH-R and *CSN*-SF/SR. The copy number of *HPH* was verified by quantitative real-time PCR, and the *TUBULIN* gene was used as a control. Strains complemented with the pKD5-GFP/pKD7-Flag plasmid vector harboring the sulfonylurea resistance gene (*SUR*)/genetic resistance gene (*G418*) were used. Western blotting and fluorescence microscopy were used to screen for positive transformants. The complemented strain with a GFP label was also used for subcellular localization analysis. All abovementioned primers are listed in Table S1.

### Phenotypic characterization

For phenotypic assays, strains were grown on CM for 9 days, after which the colony diameter and conidia production were measured. For the conidia germination and appressorium formation assays, the conidia were collected and diluted to 5 × 10^4^ conidia/mL and then inoculated on hydrophobic plastic films. After incubation at 22 °C for 4 h and 24 h, the germination and appressorium formation rates were observed, and counted under an optical microscope. For the pathogenicity assays, mycelial plugs and 5 × 10^4^ mL^−1^ conidial suspensions were incubated on cut leaves of rice and barley. Diseased leaves were imaged 4 days after inoculation. For the appressorial penetration assays, 5 × 10^4^ mL^−1^ conidial suspensions were added to the isolated barley leaves for 72 h. All the leaves were decolorized in methanol, and appressorium penetration was analyzed under a light microscope. All assays were repeated three times.

### Fluorescence observation

pKD8-MoH_2_B-mCherry was constructed by inserting a fragment of *MoH*_*2*_*B* genomic DNA without a terminator codon (TAA) into the linearized vector pKD8 digested with *Bam*HI and *Sam*I. The constructed vector was transferred into the complemented strains with MoCsn5-GFP labeled by ATMT. To determine the cellular localization, conidia were collected from 10-day-old CM agar plates and observed under a fluorescence microscope. Conidial suspensions (5 × 10^4^ mL^−1^) were inoculated on hydrophobic plastic coverslips to induce appressorium formation and observed at 24 hpi. All primers mentioned above are listed in Table S1.

### Ubiquitin assays

To assay the ubiquitination level of total proteins, mycelia were grown in liquid CM at 25 °C for 40 h and then transferred to CM supplemented with 20 µΜ MG132 for 0 and 3 h to induce ubiquitinated protein accumulation. At different time points, mycelial proteins were extracted with protein extraction buffer (150 mM NaCl, 1 mM EDTA, 50 mM Tris-HCl, 1% Triton X-100, and 1% protease inhibitor mixture).

To assay the ubiquitination level of a specific protein, using MoAtg6 as an example, pKD3 with a GFP tag was linearized at the *Eco*RI/*Sam*I sites and fused with *MoATG6* and the native promoter of *MoATG6*. Recombined plasmids were transferred into 70-15. Knockout of *MoCSN5* was performed in 70-15 expressing MoAtg6-GFP, and complementation of *MoCSN5* was performed in the resulting strain to ensure consistent transcript levels of *MoATG6.* These three strains were grown in liquid CM at 25 °C for 40 h. Then, the three strains were extracted and immunoprecipitated with anti-GFP beads and immunoblotted with anti-GFP, anti-ubiquitin, and anti-K48-ubiquitin antibodies. The formula ubiquitin/MoAtg6-GFP was used to calculate the ubiquitination levels of MoAtg6 in the different strains.

Data analysis was performed using ImageJ and GraphPad Prism 8.0 software. All assays were repeated three times. The agents and antibodies used in the abovementioned assays were MG132 (Cell Signaling Technology, 2194S), the anti-GFP affinity beads 4FF (Smart-lifesciences, SA070001), the anti-Flag affinity beads 4FF (Smart-lifesciences, SA042001), the anti-ubiquitin antibody (Cell Signaling Technology, 3936S, 1:1000), the anti-K63-ubiquitin antibody (Cell Signaling Technology, 12930S, 1:1000), the anti-K48-ubiquitin antibody (Cell Signaling Technology, 8081S, 1:1000) and the anti-β-actin antibody (ABclonal, China, AC004, 1:2000). All abovementioned primers are listed in Table S1.

### Autophagy assays

To detect the endogenous lipidation of MoAtg8, 70–15 and Δ*Mocsn5* were grown in liquid CM for 38 h and then transferred to synthetic defined media without amino acids and ammonium sulfate (SD-N) for 3 h and 6 h. The TCA-acetone method was used to extract mycelial protein, and anti-Atg8 and anti-Actin antibodies were used for protein detection by western blotting. Atg8 and Atg8-PE, which have similar molecular weights, were separated by 13.5% SDS‒PAGE with 6 M urea. ImageJ software was used to calculate the gray values of the protein bands.

To monitor autophagic flux, the GFP-MoAtg8 vector constructed in previous studies was transformed into 70-15, Δ*Mocsn1*, Δ*Mocsn4*, Δ*Mocsn5*, or Δ*Mocsn7a* by ATMT [75]. The strains were grown in liquid CM for 38 h before being transferred to liquid SD-N medium for 3 h and 6 h to induce autophagy. At different time points, mycelial proteins were extracted with protein extraction buffer, and after centrifugation at 12000 rpm for 20 min, the supernatant was used for western blotting. SDS-PAGE without urea (12.5%) was used to detect the exogenous insertion protein GFP-MoAtg8/GFP. To observe the localization of GFP-MoAtg8, 7-amino-4-chloromethylcoumarin (CMAC) was used to stain the abovementioned mycelia at 0 h and 3 h, and blue and green fluorescence was observed under a fluorescence microscope. ImageJ software was used to calculate the gray values of the protein bands.

Data analysis was performed using ImageJ and GraphPad Prism 8.0 software. All assays were repeated three times. The agents and antibodies used in the abovementioned assays were anti-β-actin antibody (ABclonal, China, AC004, 1:2000), anti-GFP antibody (Abcam, AB32146, 1:5000), anti-Atg8 antibody (MBL, PM090,1:2000), goat anti-rabbit HRP (FDbio, China, FDR007, 1:8000), goat anti-mouse HRP (FDbio, China, FDM007, 1:8000), protease inhibitor mixture (FDbio, China, FD1001), CMAC (Invitrogen, C2110), urea (Sangon Biotech, A600148-0500), TEMED (Sangon Biotech, A10076-0100), ammonium persulfate (Sangon Biotech, A100486-0025), Acryl/Bis 30% solution (Sangon Biotech, B546017-0500), 1.4 M Tris-HCl (FDbio, FD2084), and 1.5 M Tris-HCl (FDbio, FD2080).

### Phosphorylation level assay and western blotting

To assay phosphorylated Pmk1, mycelia were grown in liquid yeast extract-glucose (YEG) media at 25 °C for 40 h. The TCA-acetone method was used for the total protein extraction [76]. To assay phosphorylated Rps6, mycelia were grown in liquid CM at 25 °C for 40 h, and the TCA-acetone method was used for the extraction of total proteins. To assay phosphorylated Osm1 and Ypk1, mycelia were grown in liquid CM at 25 °C for 40 h and then transferred to CM supplemented with 0.6 M NaCl for 0, 30, 60, or 90 min or to CM supplemented with 2 µΜ amphotericin B for 0, 1, or 2 h. The TCA-acetone method was then used for total protein extraction. To detect the protein content of Atg6, mycelia were grown in liquid CM at 25 °C for 40 h. Mycelial proteins were subsequently extracted with protein extraction buffer (150 mM NaCl, 1 mM EDTA, 50 mM Tris-HCl, 1% Triton X-100, and 1% protease inhibitor mixture).

Data analysis was performed using ImageJ and GraphPad Prism 8.0 software. All assays were repeated three times. The agents and antibodies used in the abovementioned assays were anti-phospho-p44/42 MAPK antibody (Cell Signal Technology, 4370S, 1:500), anti-ERK1/2 MAPK antibody (Santa Cruz Biotechnology, sc-514302, 1:500), anti-phospho-MoYpk1 (S619) antibody and anti-MoYpk1 antibody (prepared by ABclonal Biotechnology Co., Ltd.), anti-phospho-MoRps6 antibody and anti-MoRps6 antibody (prepared by ABclonal Biotechnology Co., Ltd.), anti-Beclin1 antibody (ABclonal, China, A21191, 1:1000), and anti-β-actin antibody (ABclonal, China, AC004, 1:2000).

### Yeast two-hybrid assays, pulldown assays, and coimmunoprecipitation assays

In this study, the yeast strain Y2HGold was used, and pGADT7-T and pGBKT7-53 were used as positive controls. The coding DNA sequences (CDSs) of *MoCSN5* and *MoATG14* were amplified and fused with the bait vector pGBKT7 and the prey vector pGADT7, respectively. After sequencing, a pair of bait and prey vectors were cotransformed into the yeast Y2HGold strain according to the Matchmaker Gold Yeast Two-Hybrid System (Clontech, Mountain View, CA, USA), and the cell growth on SD-Leu-Trp-His-Ade media and SD-Leu-Trp media was observed. All abovementioned primers are listed in Table S1.

For pull-down assays, the full-length cDNA of MoAtg14 was amplified and inserted into the vector pGEX4T-1 to obtain the GST-MoAtg14 plasmid. The full-length cDNA of MoCsn5 was amplified and inserted into the vector pET35a to obtain the His-MoCsn5 plasmid. These plasmids were subsequently transformed into *E. coli* strain BL21(DE3) for expression. The bacterial cells were lysed to obtain bacterial lysates according to a previously described method [77, 78]. The extracted protein was subjected to SDS-PAGE and Coomassie brilliant blue staining to ensure protein expression. The soluble GST or GST fusion protein was incubated with 55 µl of glutathione agarose beads with GST tags (Smart-Lifesciences Biotech, SA042001) at 4 °C for 2 h. Then, the agarose beads were washed five times with washing buffer and incubated with the supernatant containing His-MoCsn5 protein for 2 h. The proteins were eluted with elution buffer and detected by immunoblotting with an anti-His antibody (Huabio, R1207-2, 1:2000) and an anti-GST antibody (Huabio, EM80701, 1:2000). All abovementioned primers are listed in Table S1.

For coimmunoprecipitation assays, taking MoAtg14 and MoCsn5 as examples, the pKD5-MoAtg14-GFP vector was constructed by inserting a fragment of *MoATG14* genomic DNA without a terminator codon (TAA) into the linearized vector pKD5 digested with *Bam*HI and *Sam*I. Then the constructed empty GFP and MoAtg14-GFP vectors with *SUR* resistance were transferred into the complemented strains with the MoCsn5-3 × Flag label (*G418* resistance). The positive transformants were screened by PCR, western blot, and fluorescence microscopy. Total proteins were extracted from the mycelia of the positive transformants coexpressing GFP and MoCsn5-3 × Flag, MoAtg14-GFP, and MoCsn5-3 × Flag with protein extraction buffer. After centrifugation at 12,000 rpm for 20 min, the supernatant of the lysates was incubated with GFP beads at 4 °C for 4 h. Moreover, anti-GFP antibody and anti-Flag antibody were used to detect the whole protein and the eluent of affinity beads eluted with acidic amino acids, respectively. All abovementioned primers are listed in Table S1.

### Supplementary Information

**Additional file 1: Figure S1.** Verification of deletion mutants by PCR and quantitative real-time PCR. (A) Rice blast fungus knockout model. (B) PCR detection was performed on the null mutants, and the recombinant fragment (LF) was cloned from the positive strain but not from the WT strain. The 500-bp characteristic fragment (SF) of the target gene was cloned in the WT strain but not in the mutants. *TUBULIN *was used as a positive control. (C) The copy number of the resistance gene *HPH* in the deletion mutants was verified by quantitative real-time PCR.** Figure S2.** The targeted gene destruction of CSN subunits decreased the growth, sporulation, and pathogenicity of *M. oryzae*. (A) Colony morphology of 70-15, Δ*Mocsn1*,* Mocsn1-C*, Δ*Mocsn2*,* Mocsn2-C*, Δ*Mocsn3*,* Mocsn3-C*, Δ*Mocsn4*,* Mocsn4-C*, Δ*Mocsn7a*, and* Mocsn7a-C*. The strains were grown on CM and MM plates for 9 days. (B) Statistical analysis of the colony growth diameter. The data were analyzed using GraphPad Prism 8.0 software. The error bars represent the standard deviations. ***P < 0.001. (C) Conidiophores of 70-15, Δ*Mocsn1*,* Mocsn1-C*, Δ*Mocsn2*,* Mocsn2-C*, Δ*Mocsn3*,* Mocsn3-C*, Δ*Mocsn4*,* Mocsn4-C*, Δ*Mocsn7a*, and* Mocsn7a-C*. The strains were cultivated in an incubator at 25 ℃ for 9 days and observed under an optical microscope. (D) Disease spots of detached barley leaves inoculated with mycelial plugs from 70-15, Δ*Mocsn1*,* Mocsn1-C*, Δ*Mocsn2*,* Mocsn2-C*, Δ*Mocsn3*,* Mocsn3-C*, Δ*Mocsn4*,* Mocsn4-C*, Δ*Mocsn7a*, and* Mocsn7a-C*. Leaves were cultured at 25 ℃ for 4 days after inoculation.** Figure S3.** CSN subunits are involved in the regulation of autophagy in *M. oryzae*. (A) Yeast two-hybrid assays were used to detect the interactions between CSN subunits and Atg-related proteins. Pairs of pGBKT7-53 and PGADT7-T were used as positive controls. Yeast transformants carrying the indicated constructs were plated onto selective plates supplemented without Leu/Trp or Leu/Trp/His/Ade for growth assays. (B) Autophagic flux analysis of GFP-MoAtg8 in 70-15 and *ΔMocsn1*.Total GFP-MoAtg8 and free GFP were detected by western blotting. (C) Autophagic flux analysis of GFP-MoAtg8 in 70-15, Δ*Mocsn4 *andΔ*Mocsn7a*.** Figure S4.** Identification of the Csn5 protein in rice blast fungus. (A) Comparison of Csn5 amino acid sequences in different eukaryotes via DNAMAN 8 software. The amino acid sequences used for comparison were from *Magnaporthe oryzae* (XP_003712833.1), *Fusarium graminearum* (XP_011318525.1), *Mus musculus* (NP_038743.1), *Caenorhabditis elegans* (NP_500841.1), *Aspergillus fumigatus *(XP_755961.2), *Homo sapiens* (NP_006828.2) and *Neurospora crassa *(XP_956786.1). (B) Phylogenetic trees of the Csn5 proteins constructed using MEGA 11. (C) Conserved MPN domain of Csn5 in different eukaryotes was identified using IBS 1.0.1 software.

**Additional file 2: Table S1.** Primers used in this study.

## Data Availability

Dataset used or analyzed during the current study are available from the corresponding author on reasonable request.
