# Supplementary figures and images for: Csn5 inhibits autophagy by regulating the ubiquitination of Atg6 and Tor to mediate the pathogenicity of Magnaporthe oryzae
Source: Cell Commun Signal. 2024 Apr 9;22:222. doi: 10.1186/s12964-024-01598-7 (PMC11003145; doi:10.1186/s12964-024-01598-7)

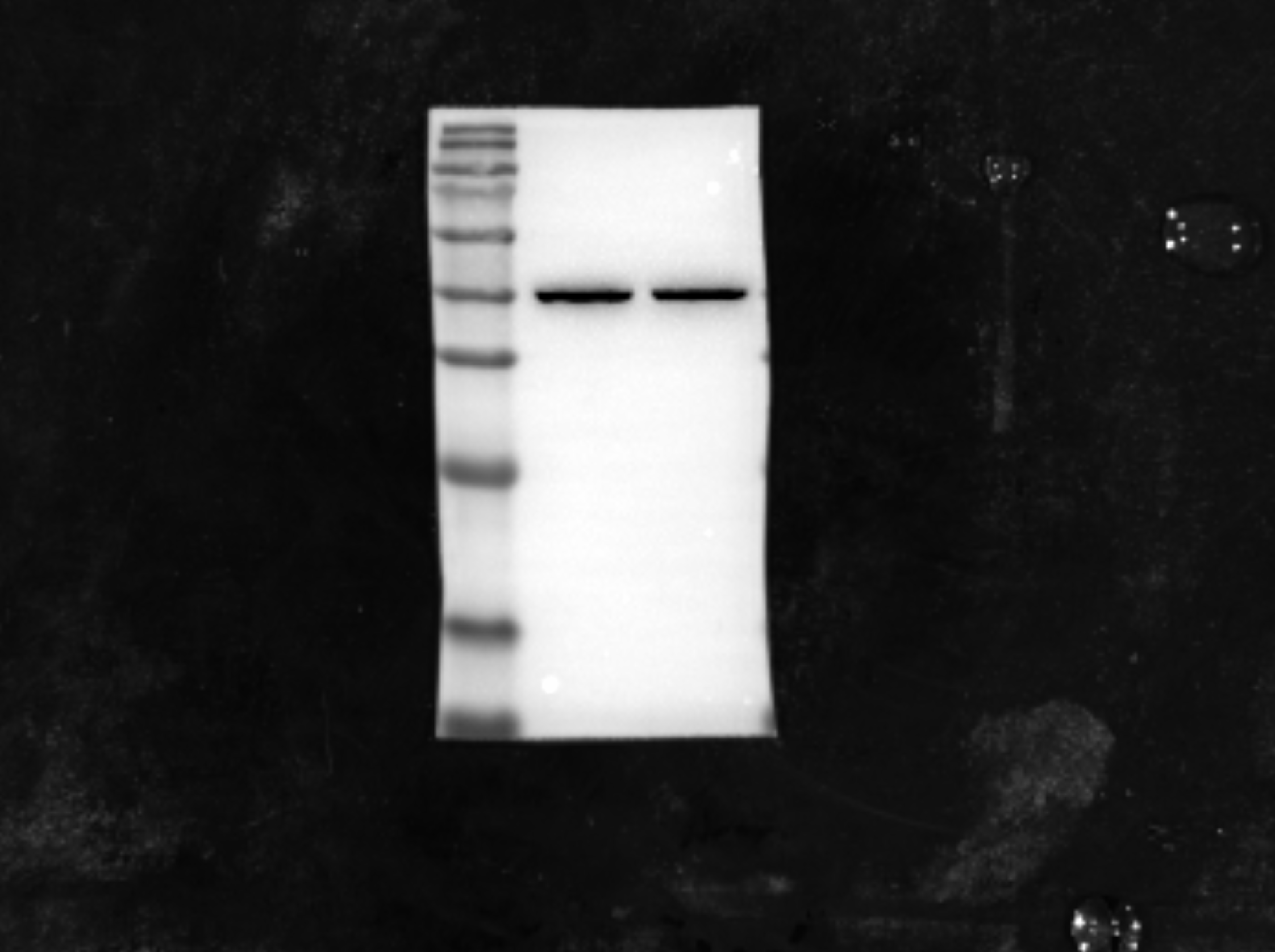

Supplement: Supplementary file 1 — Additional file 1: Figure S1. Verification of deletion mutants by PCR and quantitative real-time PCR. (A) Rice blast fungus knockout model. (B) PCR detection was performed on the null mutants, and the recombinant fragment (LF) was cloned from the positive strain but not from the WT strain. The 500-bp characteristic fragment (SF) of the target gene was cloned in the WT strain but not in the mutants. TUBULIN was used as a positive control. (C) The copy number of the resistance gene HPH in the deletion mutants was verified by quantitative real-time PCR. Figure S2. The targeted gene destruction of CSN subunits decreased the growth, sporulation, and pathogenicity of M. oryzae. (A) Colony morphology of 70-15, ΔMocsn1, Mocsn1-C, ΔMocsn2, Mocsn2-C, ΔMocsn3, Mocsn3-C, ΔMocsn4, Mocsn4-C, ΔMocsn7a, and Mocsn7a-C. The strains were grown on CM and MM plates for 9 days. (B) Statistical analysis of the colony growth diameter. The data were analyzed using GraphPad Prism 8.0 software. The error bars represent the standard deviations. ***P < 0.001. (C) Conidiophores of 70-15, ΔMocsn1, Mocsn1-C, ΔMocsn2, Mocsn2-C, ΔMocsn3, Mocsn3-C, ΔMocsn4, Mocsn4-C, ΔMocsn7a, and Mocsn7a-C. The strains were cultivated in an incubator at 25 ℃ for 9 days and observed under an optical microscope. (D) Disease spots of detached barley leaves inoculated with mycelial plugs from 70-15, ΔMocsn1, Mocsn1-C, ΔMocsn2, Mocsn2-C, ΔMocsn3, Mocsn3-C, ΔMocsn4, Mocsn4-C, ΔMocsn7a, and Mocsn7a-C. Leaves were cultured at 25 ℃ for 4 days after inoculation. Figure S3. CSN subunits are involved in the regulation of autophagy in M. oryzae. (A) Yeast two-hybrid assays were used to detect the interactions between CSN subunits and Atg-related proteins. Pairs of pGBKT7-53 and PGADT7-T were used as positive controls. Yeast transformants carrying the indicated constructs were plated onto selective plates supplemented without Leu/Trp or Leu/Trp/His/Ade for growth assays. (B) Autophagic flux analysis of GFP-MoAtg8 in 70-15 and ΔMo [file 12964_2024_1598_MOESM1_ESM.zip › Supplementary files/Figure2I-Actin.tif]

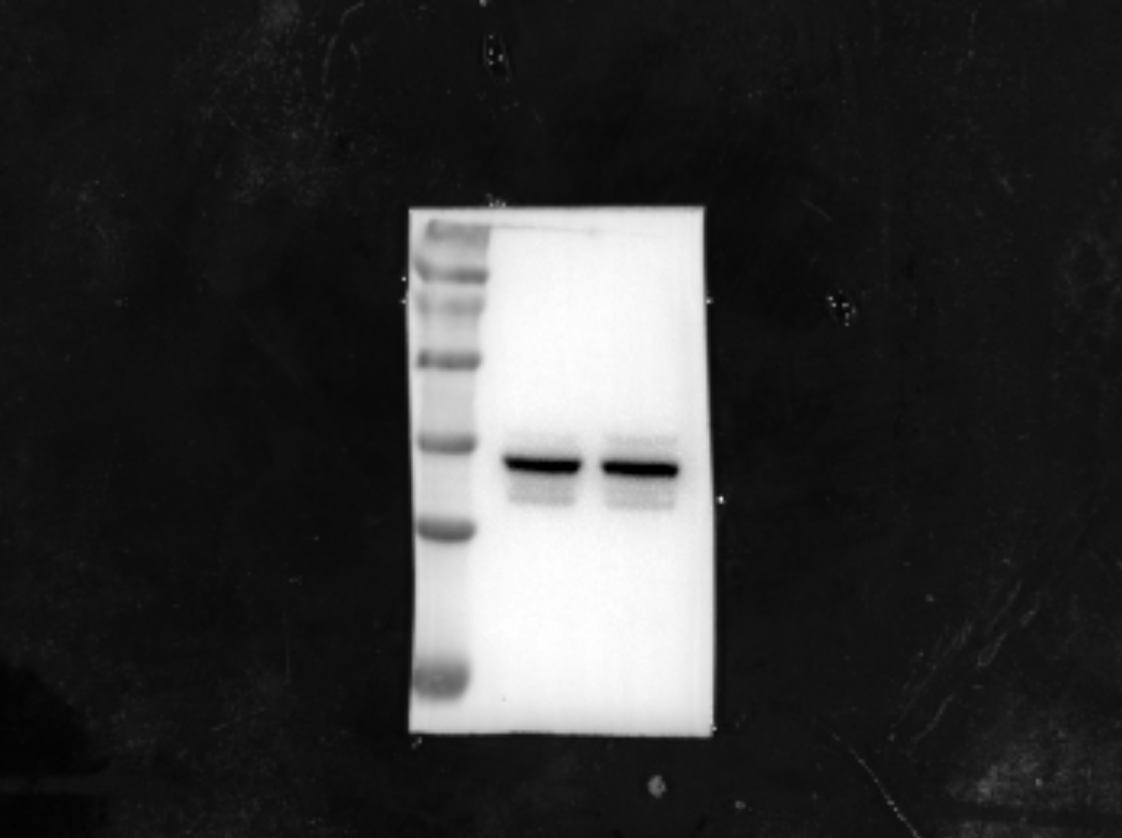

Supplement: Supplementary file 1 — Additional file 1: Figure S1. Verification of deletion mutants by PCR and quantitative real-time PCR. (A) Rice blast fungus knockout model. (B) PCR detection was performed on the null mutants, and the recombinant fragment (LF) was cloned from the positive strain but not from the WT strain. The 500-bp characteristic fragment (SF) of the target gene was cloned in the WT strain but not in the mutants. TUBULIN was used as a positive control. (C) The copy number of the resistance gene HPH in the deletion mutants was verified by quantitative real-time PCR. Figure S2. The targeted gene destruction of CSN subunits decreased the growth, sporulation, and pathogenicity of M. oryzae. (A) Colony morphology of 70-15, ΔMocsn1, Mocsn1-C, ΔMocsn2, Mocsn2-C, ΔMocsn3, Mocsn3-C, ΔMocsn4, Mocsn4-C, ΔMocsn7a, and Mocsn7a-C. The strains were grown on CM and MM plates for 9 days. (B) Statistical analysis of the colony growth diameter. The data were analyzed using GraphPad Prism 8.0 software. The error bars represent the standard deviations. ***P < 0.001. (C) Conidiophores of 70-15, ΔMocsn1, Mocsn1-C, ΔMocsn2, Mocsn2-C, ΔMocsn3, Mocsn3-C, ΔMocsn4, Mocsn4-C, ΔMocsn7a, and Mocsn7a-C. The strains were cultivated in an incubator at 25 ℃ for 9 days and observed under an optical microscope. (D) Disease spots of detached barley leaves inoculated with mycelial plugs from 70-15, ΔMocsn1, Mocsn1-C, ΔMocsn2, Mocsn2-C, ΔMocsn3, Mocsn3-C, ΔMocsn4, Mocsn4-C, ΔMocsn7a, and Mocsn7a-C. Leaves were cultured at 25 ℃ for 4 days after inoculation. Figure S3. CSN subunits are involved in the regulation of autophagy in M. oryzae. (A) Yeast two-hybrid assays were used to detect the interactions between CSN subunits and Atg-related proteins. Pairs of pGBKT7-53 and PGADT7-T were used as positive controls. Yeast transformants carrying the indicated constructs were plated onto selective plates supplemented without Leu/Trp or Leu/Trp/His/Ade for growth assays. (B) Autophagic flux analysis of GFP-MoAtg8 in 70-15 and ΔMo [file 12964_2024_1598_MOESM1_ESM.zip › Supplementary files/Figure2I-Pmk1.tif]

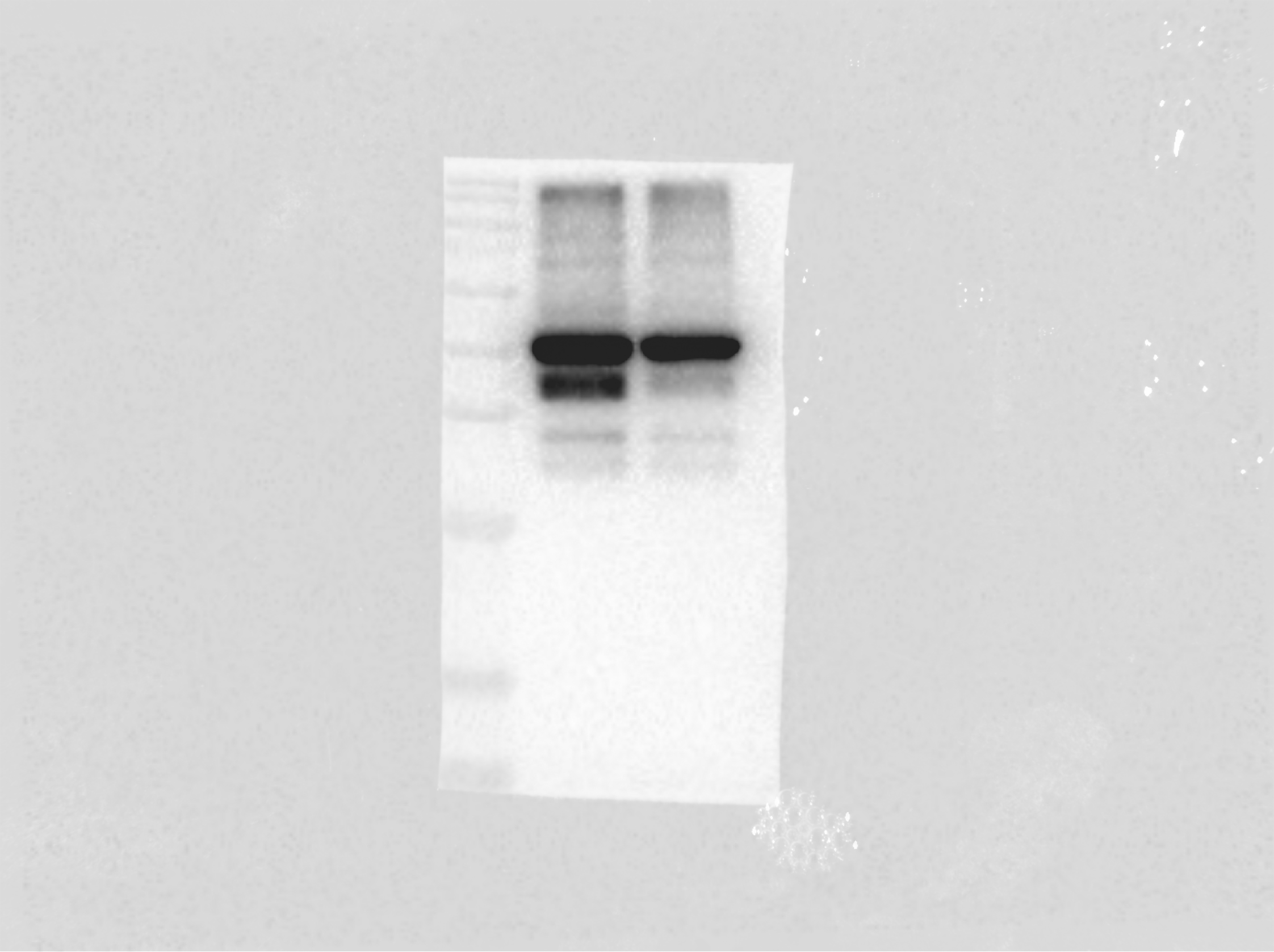

Supplement: Supplementary file 1 — Additional file 1: Figure S1. Verification of deletion mutants by PCR and quantitative real-time PCR. (A) Rice blast fungus knockout model. (B) PCR detection was performed on the null mutants, and the recombinant fragment (LF) was cloned from the positive strain but not from the WT strain. The 500-bp characteristic fragment (SF) of the target gene was cloned in the WT strain but not in the mutants. TUBULIN was used as a positive control. (C) The copy number of the resistance gene HPH in the deletion mutants was verified by quantitative real-time PCR. Figure S2. The targeted gene destruction of CSN subunits decreased the growth, sporulation, and pathogenicity of M. oryzae. (A) Colony morphology of 70-15, ΔMocsn1, Mocsn1-C, ΔMocsn2, Mocsn2-C, ΔMocsn3, Mocsn3-C, ΔMocsn4, Mocsn4-C, ΔMocsn7a, and Mocsn7a-C. The strains were grown on CM and MM plates for 9 days. (B) Statistical analysis of the colony growth diameter. The data were analyzed using GraphPad Prism 8.0 software. The error bars represent the standard deviations. ***P < 0.001. (C) Conidiophores of 70-15, ΔMocsn1, Mocsn1-C, ΔMocsn2, Mocsn2-C, ΔMocsn3, Mocsn3-C, ΔMocsn4, Mocsn4-C, ΔMocsn7a, and Mocsn7a-C. The strains were cultivated in an incubator at 25 ℃ for 9 days and observed under an optical microscope. (D) Disease spots of detached barley leaves inoculated with mycelial plugs from 70-15, ΔMocsn1, Mocsn1-C, ΔMocsn2, Mocsn2-C, ΔMocsn3, Mocsn3-C, ΔMocsn4, Mocsn4-C, ΔMocsn7a, and Mocsn7a-C. Leaves were cultured at 25 ℃ for 4 days after inoculation. Figure S3. CSN subunits are involved in the regulation of autophagy in M. oryzae. (A) Yeast two-hybrid assays were used to detect the interactions between CSN subunits and Atg-related proteins. Pairs of pGBKT7-53 and PGADT7-T were used as positive controls. Yeast transformants carrying the indicated constructs were plated onto selective plates supplemented without Leu/Trp or Leu/Trp/His/Ade for growth assays. (B) Autophagic flux analysis of GFP-MoAtg8 in 70-15 and ΔMo [file 12964_2024_1598_MOESM1_ESM.zip › Supplementary files/Figure2I-p-Pmk1.tif]

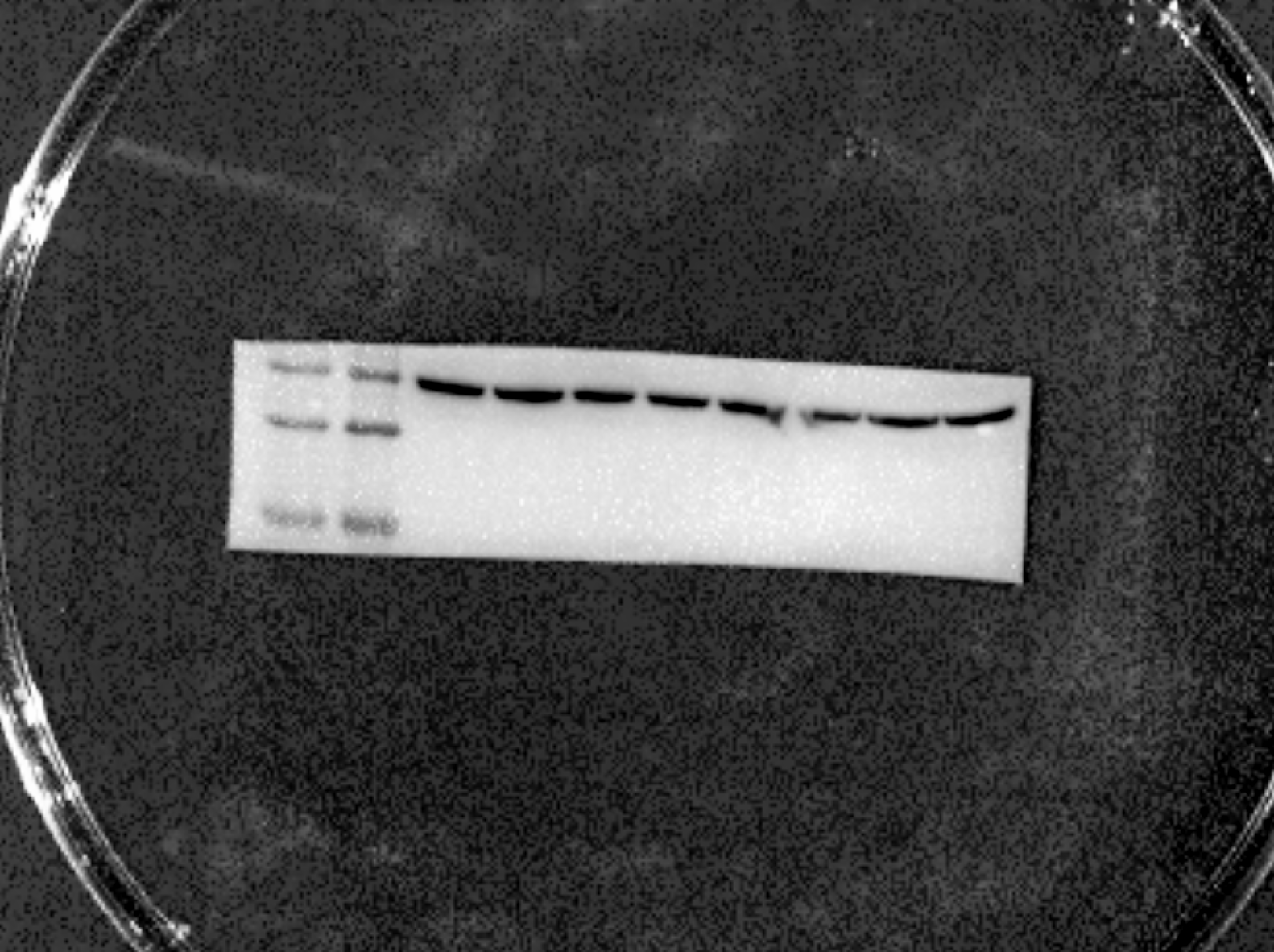

Supplement: Supplementary file 1 — Additional file 1: Figure S1. Verification of deletion mutants by PCR and quantitative real-time PCR. (A) Rice blast fungus knockout model. (B) PCR detection was performed on the null mutants, and the recombinant fragment (LF) was cloned from the positive strain but not from the WT strain. The 500-bp characteristic fragment (SF) of the target gene was cloned in the WT strain but not in the mutants. TUBULIN was used as a positive control. (C) The copy number of the resistance gene HPH in the deletion mutants was verified by quantitative real-time PCR. Figure S2. The targeted gene destruction of CSN subunits decreased the growth, sporulation, and pathogenicity of M. oryzae. (A) Colony morphology of 70-15, ΔMocsn1, Mocsn1-C, ΔMocsn2, Mocsn2-C, ΔMocsn3, Mocsn3-C, ΔMocsn4, Mocsn4-C, ΔMocsn7a, and Mocsn7a-C. The strains were grown on CM and MM plates for 9 days. (B) Statistical analysis of the colony growth diameter. The data were analyzed using GraphPad Prism 8.0 software. The error bars represent the standard deviations. ***P < 0.001. (C) Conidiophores of 70-15, ΔMocsn1, Mocsn1-C, ΔMocsn2, Mocsn2-C, ΔMocsn3, Mocsn3-C, ΔMocsn4, Mocsn4-C, ΔMocsn7a, and Mocsn7a-C. The strains were cultivated in an incubator at 25 ℃ for 9 days and observed under an optical microscope. (D) Disease spots of detached barley leaves inoculated with mycelial plugs from 70-15, ΔMocsn1, Mocsn1-C, ΔMocsn2, Mocsn2-C, ΔMocsn3, Mocsn3-C, ΔMocsn4, Mocsn4-C, ΔMocsn7a, and Mocsn7a-C. Leaves were cultured at 25 ℃ for 4 days after inoculation. Figure S3. CSN subunits are involved in the regulation of autophagy in M. oryzae. (A) Yeast two-hybrid assays were used to detect the interactions between CSN subunits and Atg-related proteins. Pairs of pGBKT7-53 and PGADT7-T were used as positive controls. Yeast transformants carrying the indicated constructs were plated onto selective plates supplemented without Leu/Trp or Leu/Trp/His/Ade for growth assays. (B) Autophagic flux analysis of GFP-MoAtg8 in 70-15 and ΔMo [file 12964_2024_1598_MOESM1_ESM.zip › Supplementary files/Figure3G-Actin.tif]

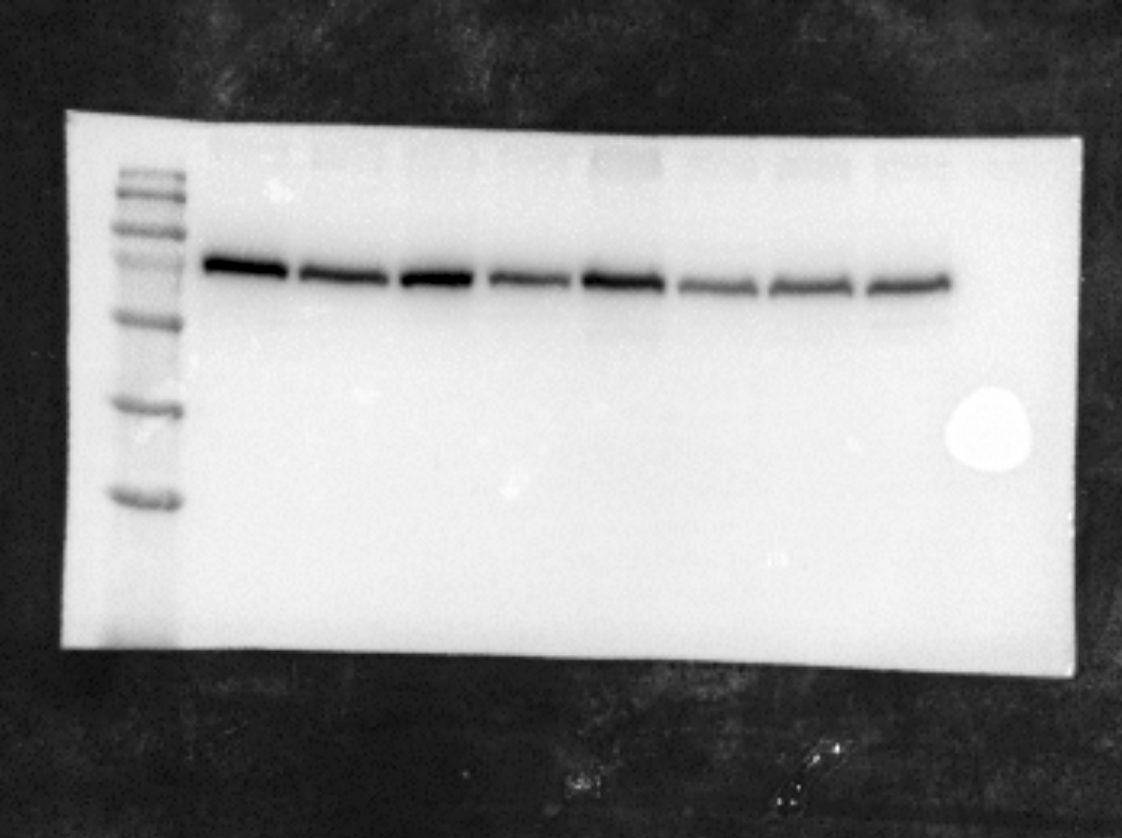

Supplement: Supplementary file 1 — Additional file 1: Figure S1. Verification of deletion mutants by PCR and quantitative real-time PCR. (A) Rice blast fungus knockout model. (B) PCR detection was performed on the null mutants, and the recombinant fragment (LF) was cloned from the positive strain but not from the WT strain. The 500-bp characteristic fragment (SF) of the target gene was cloned in the WT strain but not in the mutants. TUBULIN was used as a positive control. (C) The copy number of the resistance gene HPH in the deletion mutants was verified by quantitative real-time PCR. Figure S2. The targeted gene destruction of CSN subunits decreased the growth, sporulation, and pathogenicity of M. oryzae. (A) Colony morphology of 70-15, ΔMocsn1, Mocsn1-C, ΔMocsn2, Mocsn2-C, ΔMocsn3, Mocsn3-C, ΔMocsn4, Mocsn4-C, ΔMocsn7a, and Mocsn7a-C. The strains were grown on CM and MM plates for 9 days. (B) Statistical analysis of the colony growth diameter. The data were analyzed using GraphPad Prism 8.0 software. The error bars represent the standard deviations. ***P < 0.001. (C) Conidiophores of 70-15, ΔMocsn1, Mocsn1-C, ΔMocsn2, Mocsn2-C, ΔMocsn3, Mocsn3-C, ΔMocsn4, Mocsn4-C, ΔMocsn7a, and Mocsn7a-C. The strains were cultivated in an incubator at 25 ℃ for 9 days and observed under an optical microscope. (D) Disease spots of detached barley leaves inoculated with mycelial plugs from 70-15, ΔMocsn1, Mocsn1-C, ΔMocsn2, Mocsn2-C, ΔMocsn3, Mocsn3-C, ΔMocsn4, Mocsn4-C, ΔMocsn7a, and Mocsn7a-C. Leaves were cultured at 25 ℃ for 4 days after inoculation. Figure S3. CSN subunits are involved in the regulation of autophagy in M. oryzae. (A) Yeast two-hybrid assays were used to detect the interactions between CSN subunits and Atg-related proteins. Pairs of pGBKT7-53 and PGADT7-T were used as positive controls. Yeast transformants carrying the indicated constructs were plated onto selective plates supplemented without Leu/Trp or Leu/Trp/His/Ade for growth assays. (B) Autophagic flux analysis of GFP-MoAtg8 in 70-15 and ΔMo [file 12964_2024_1598_MOESM1_ESM.zip › Supplementary files/Figure3G-p-Ypk1.tif]

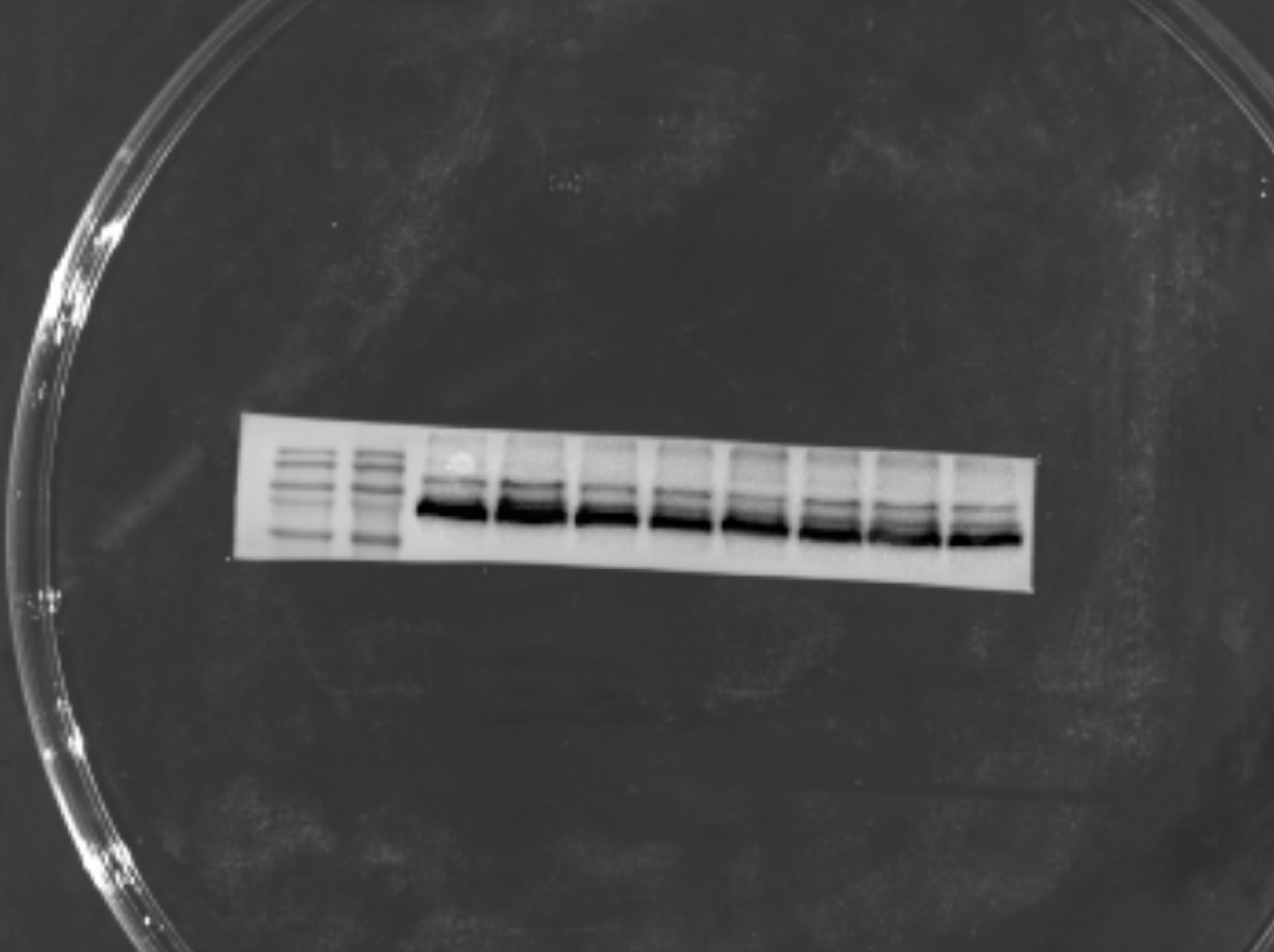

Supplement: Supplementary file 1 — Additional file 1: Figure S1. Verification of deletion mutants by PCR and quantitative real-time PCR. (A) Rice blast fungus knockout model. (B) PCR detection was performed on the null mutants, and the recombinant fragment (LF) was cloned from the positive strain but not from the WT strain. The 500-bp characteristic fragment (SF) of the target gene was cloned in the WT strain but not in the mutants. TUBULIN was used as a positive control. (C) The copy number of the resistance gene HPH in the deletion mutants was verified by quantitative real-time PCR. Figure S2. The targeted gene destruction of CSN subunits decreased the growth, sporulation, and pathogenicity of M. oryzae. (A) Colony morphology of 70-15, ΔMocsn1, Mocsn1-C, ΔMocsn2, Mocsn2-C, ΔMocsn3, Mocsn3-C, ΔMocsn4, Mocsn4-C, ΔMocsn7a, and Mocsn7a-C. The strains were grown on CM and MM plates for 9 days. (B) Statistical analysis of the colony growth diameter. The data were analyzed using GraphPad Prism 8.0 software. The error bars represent the standard deviations. ***P < 0.001. (C) Conidiophores of 70-15, ΔMocsn1, Mocsn1-C, ΔMocsn2, Mocsn2-C, ΔMocsn3, Mocsn3-C, ΔMocsn4, Mocsn4-C, ΔMocsn7a, and Mocsn7a-C. The strains were cultivated in an incubator at 25 ℃ for 9 days and observed under an optical microscope. (D) Disease spots of detached barley leaves inoculated with mycelial plugs from 70-15, ΔMocsn1, Mocsn1-C, ΔMocsn2, Mocsn2-C, ΔMocsn3, Mocsn3-C, ΔMocsn4, Mocsn4-C, ΔMocsn7a, and Mocsn7a-C. Leaves were cultured at 25 ℃ for 4 days after inoculation. Figure S3. CSN subunits are involved in the regulation of autophagy in M. oryzae. (A) Yeast two-hybrid assays were used to detect the interactions between CSN subunits and Atg-related proteins. Pairs of pGBKT7-53 and PGADT7-T were used as positive controls. Yeast transformants carrying the indicated constructs were plated onto selective plates supplemented without Leu/Trp or Leu/Trp/His/Ade for growth assays. (B) Autophagic flux analysis of GFP-MoAtg8 in 70-15 and ΔMo [file 12964_2024_1598_MOESM1_ESM.zip › Supplementary files/Figure3G-Ypk1.tif]

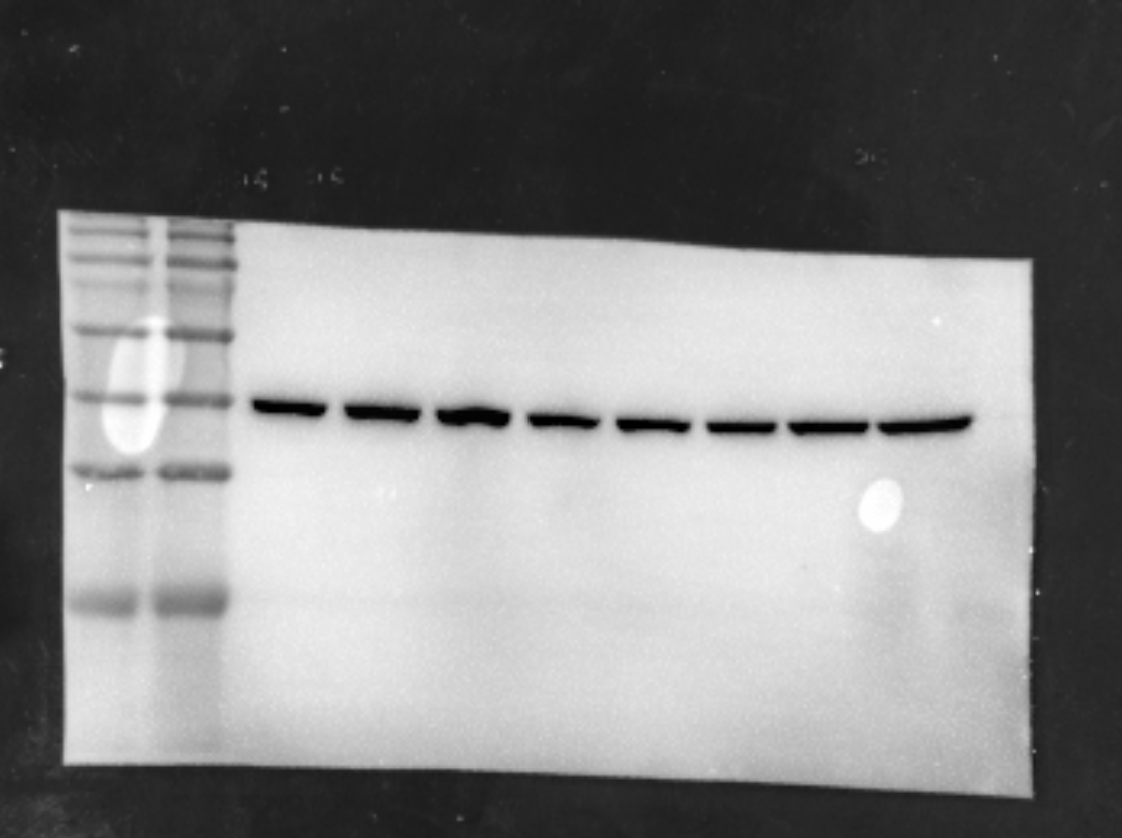

Supplement: Supplementary file 1 — Additional file 1: Figure S1. Verification of deletion mutants by PCR and quantitative real-time PCR. (A) Rice blast fungus knockout model. (B) PCR detection was performed on the null mutants, and the recombinant fragment (LF) was cloned from the positive strain but not from the WT strain. The 500-bp characteristic fragment (SF) of the target gene was cloned in the WT strain but not in the mutants. TUBULIN was used as a positive control. (C) The copy number of the resistance gene HPH in the deletion mutants was verified by quantitative real-time PCR. Figure S2. The targeted gene destruction of CSN subunits decreased the growth, sporulation, and pathogenicity of M. oryzae. (A) Colony morphology of 70-15, ΔMocsn1, Mocsn1-C, ΔMocsn2, Mocsn2-C, ΔMocsn3, Mocsn3-C, ΔMocsn4, Mocsn4-C, ΔMocsn7a, and Mocsn7a-C. The strains were grown on CM and MM plates for 9 days. (B) Statistical analysis of the colony growth diameter. The data were analyzed using GraphPad Prism 8.0 software. The error bars represent the standard deviations. ***P < 0.001. (C) Conidiophores of 70-15, ΔMocsn1, Mocsn1-C, ΔMocsn2, Mocsn2-C, ΔMocsn3, Mocsn3-C, ΔMocsn4, Mocsn4-C, ΔMocsn7a, and Mocsn7a-C. The strains were cultivated in an incubator at 25 ℃ for 9 days and observed under an optical microscope. (D) Disease spots of detached barley leaves inoculated with mycelial plugs from 70-15, ΔMocsn1, Mocsn1-C, ΔMocsn2, Mocsn2-C, ΔMocsn3, Mocsn3-C, ΔMocsn4, Mocsn4-C, ΔMocsn7a, and Mocsn7a-C. Leaves were cultured at 25 ℃ for 4 days after inoculation. Figure S3. CSN subunits are involved in the regulation of autophagy in M. oryzae. (A) Yeast two-hybrid assays were used to detect the interactions between CSN subunits and Atg-related proteins. Pairs of pGBKT7-53 and PGADT7-T were used as positive controls. Yeast transformants carrying the indicated constructs were plated onto selective plates supplemented without Leu/Trp or Leu/Trp/His/Ade for growth assays. (B) Autophagic flux analysis of GFP-MoAtg8 in 70-15 and ΔMo [file 12964_2024_1598_MOESM1_ESM.zip › Supplementary files/Figure3H-Actin.tif]

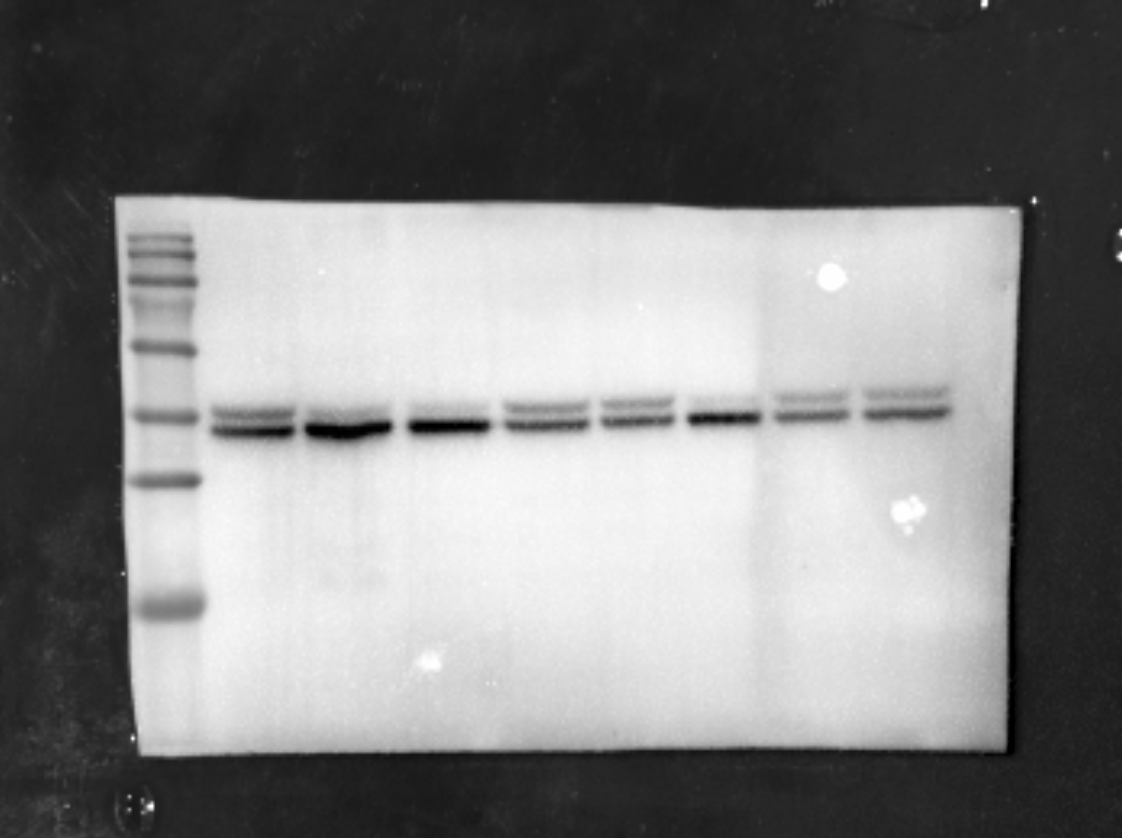

Supplement: Supplementary file 1 — Additional file 1: Figure S1. Verification of deletion mutants by PCR and quantitative real-time PCR. (A) Rice blast fungus knockout model. (B) PCR detection was performed on the null mutants, and the recombinant fragment (LF) was cloned from the positive strain but not from the WT strain. The 500-bp characteristic fragment (SF) of the target gene was cloned in the WT strain but not in the mutants. TUBULIN was used as a positive control. (C) The copy number of the resistance gene HPH in the deletion mutants was verified by quantitative real-time PCR. Figure S2. The targeted gene destruction of CSN subunits decreased the growth, sporulation, and pathogenicity of M. oryzae. (A) Colony morphology of 70-15, ΔMocsn1, Mocsn1-C, ΔMocsn2, Mocsn2-C, ΔMocsn3, Mocsn3-C, ΔMocsn4, Mocsn4-C, ΔMocsn7a, and Mocsn7a-C. The strains were grown on CM and MM plates for 9 days. (B) Statistical analysis of the colony growth diameter. The data were analyzed using GraphPad Prism 8.0 software. The error bars represent the standard deviations. ***P < 0.001. (C) Conidiophores of 70-15, ΔMocsn1, Mocsn1-C, ΔMocsn2, Mocsn2-C, ΔMocsn3, Mocsn3-C, ΔMocsn4, Mocsn4-C, ΔMocsn7a, and Mocsn7a-C. The strains were cultivated in an incubator at 25 ℃ for 9 days and observed under an optical microscope. (D) Disease spots of detached barley leaves inoculated with mycelial plugs from 70-15, ΔMocsn1, Mocsn1-C, ΔMocsn2, Mocsn2-C, ΔMocsn3, Mocsn3-C, ΔMocsn4, Mocsn4-C, ΔMocsn7a, and Mocsn7a-C. Leaves were cultured at 25 ℃ for 4 days after inoculation. Figure S3. CSN subunits are involved in the regulation of autophagy in M. oryzae. (A) Yeast two-hybrid assays were used to detect the interactions between CSN subunits and Atg-related proteins. Pairs of pGBKT7-53 and PGADT7-T were used as positive controls. Yeast transformants carrying the indicated constructs were plated onto selective plates supplemented without Leu/Trp or Leu/Trp/His/Ade for growth assays. (B) Autophagic flux analysis of GFP-MoAtg8 in 70-15 and ΔMo [file 12964_2024_1598_MOESM1_ESM.zip › Supplementary files/Figure3H-p-Osm1.tif]

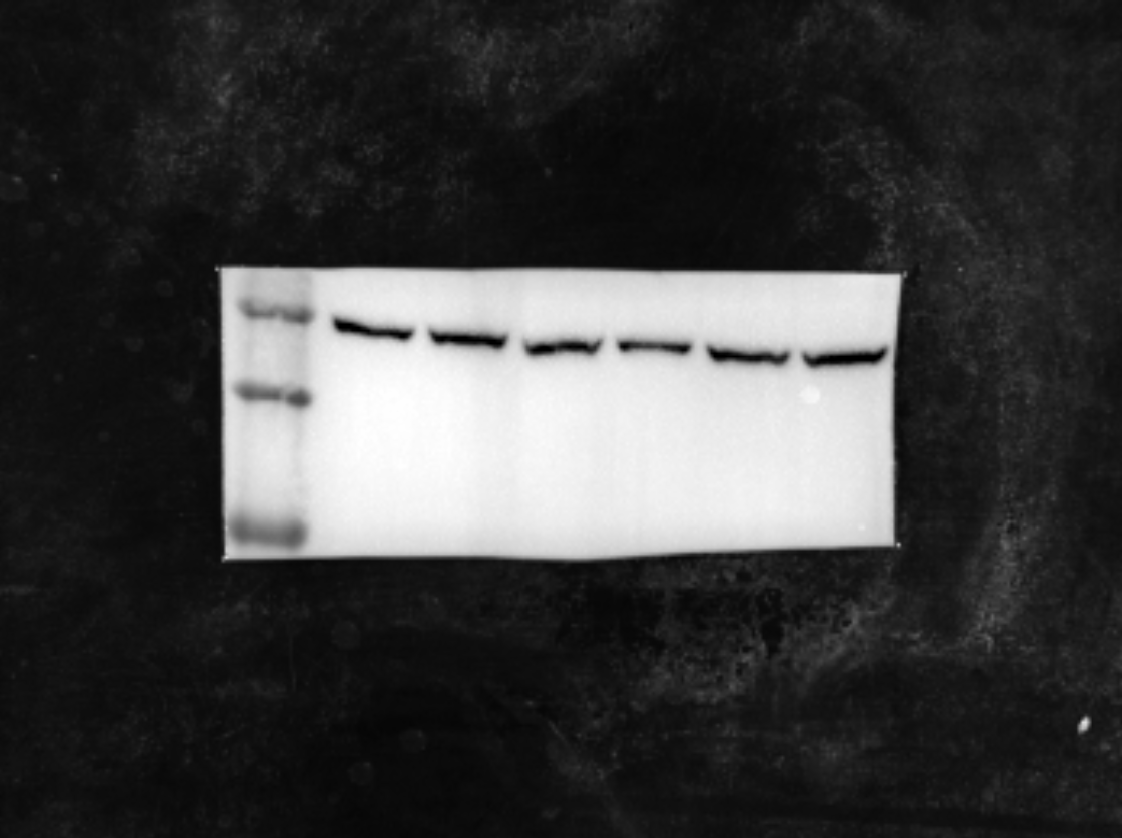

Supplement: Supplementary file 1 — Additional file 1: Figure S1. Verification of deletion mutants by PCR and quantitative real-time PCR. (A) Rice blast fungus knockout model. (B) PCR detection was performed on the null mutants, and the recombinant fragment (LF) was cloned from the positive strain but not from the WT strain. The 500-bp characteristic fragment (SF) of the target gene was cloned in the WT strain but not in the mutants. TUBULIN was used as a positive control. (C) The copy number of the resistance gene HPH in the deletion mutants was verified by quantitative real-time PCR. Figure S2. The targeted gene destruction of CSN subunits decreased the growth, sporulation, and pathogenicity of M. oryzae. (A) Colony morphology of 70-15, ΔMocsn1, Mocsn1-C, ΔMocsn2, Mocsn2-C, ΔMocsn3, Mocsn3-C, ΔMocsn4, Mocsn4-C, ΔMocsn7a, and Mocsn7a-C. The strains were grown on CM and MM plates for 9 days. (B) Statistical analysis of the colony growth diameter. The data were analyzed using GraphPad Prism 8.0 software. The error bars represent the standard deviations. ***P < 0.001. (C) Conidiophores of 70-15, ΔMocsn1, Mocsn1-C, ΔMocsn2, Mocsn2-C, ΔMocsn3, Mocsn3-C, ΔMocsn4, Mocsn4-C, ΔMocsn7a, and Mocsn7a-C. The strains were cultivated in an incubator at 25 ℃ for 9 days and observed under an optical microscope. (D) Disease spots of detached barley leaves inoculated with mycelial plugs from 70-15, ΔMocsn1, Mocsn1-C, ΔMocsn2, Mocsn2-C, ΔMocsn3, Mocsn3-C, ΔMocsn4, Mocsn4-C, ΔMocsn7a, and Mocsn7a-C. Leaves were cultured at 25 ℃ for 4 days after inoculation. Figure S3. CSN subunits are involved in the regulation of autophagy in M. oryzae. (A) Yeast two-hybrid assays were used to detect the interactions between CSN subunits and Atg-related proteins. Pairs of pGBKT7-53 and PGADT7-T were used as positive controls. Yeast transformants carrying the indicated constructs were plated onto selective plates supplemented without Leu/Trp or Leu/Trp/His/Ade for growth assays. (B) Autophagic flux analysis of GFP-MoAtg8 in 70-15 and ΔMo [file 12964_2024_1598_MOESM1_ESM.zip › Supplementary files/Figure3I-Actin.tif]

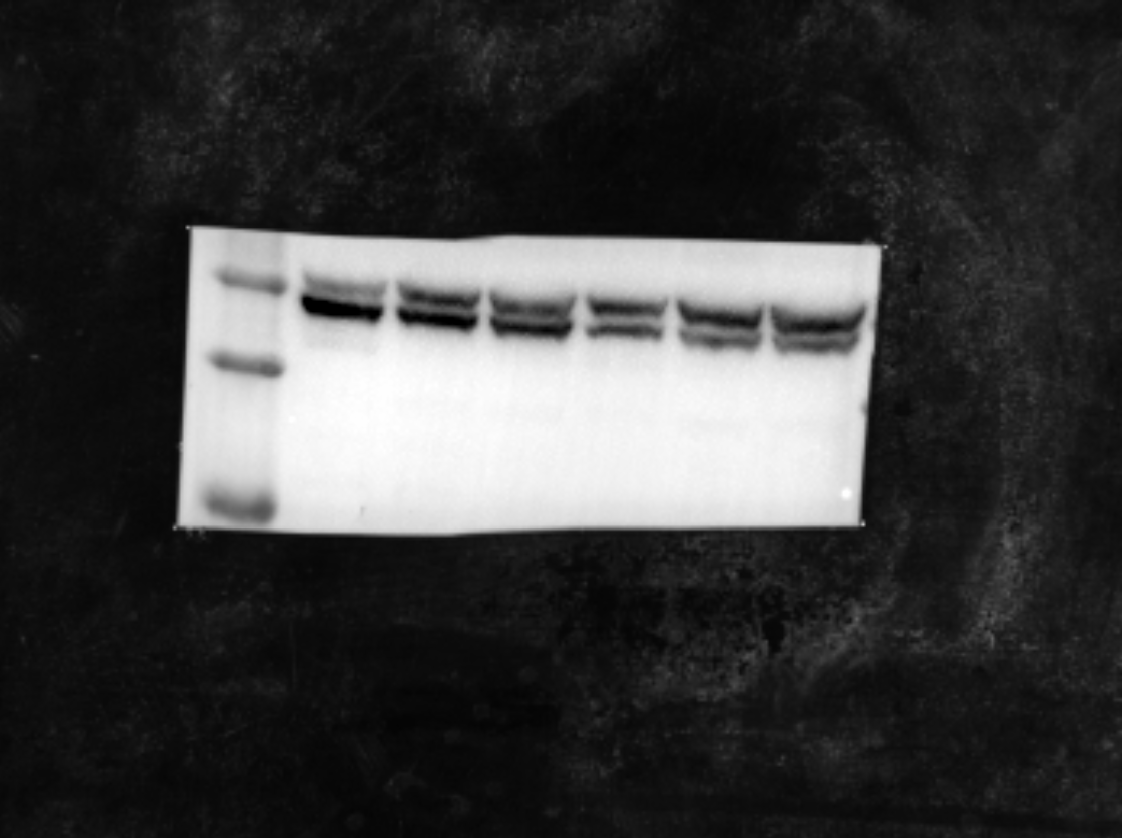

Supplement: Supplementary file 1 — Additional file 1: Figure S1. Verification of deletion mutants by PCR and quantitative real-time PCR. (A) Rice blast fungus knockout model. (B) PCR detection was performed on the null mutants, and the recombinant fragment (LF) was cloned from the positive strain but not from the WT strain. The 500-bp characteristic fragment (SF) of the target gene was cloned in the WT strain but not in the mutants. TUBULIN was used as a positive control. (C) The copy number of the resistance gene HPH in the deletion mutants was verified by quantitative real-time PCR. Figure S2. The targeted gene destruction of CSN subunits decreased the growth, sporulation, and pathogenicity of M. oryzae. (A) Colony morphology of 70-15, ΔMocsn1, Mocsn1-C, ΔMocsn2, Mocsn2-C, ΔMocsn3, Mocsn3-C, ΔMocsn4, Mocsn4-C, ΔMocsn7a, and Mocsn7a-C. The strains were grown on CM and MM plates for 9 days. (B) Statistical analysis of the colony growth diameter. The data were analyzed using GraphPad Prism 8.0 software. The error bars represent the standard deviations. ***P < 0.001. (C) Conidiophores of 70-15, ΔMocsn1, Mocsn1-C, ΔMocsn2, Mocsn2-C, ΔMocsn3, Mocsn3-C, ΔMocsn4, Mocsn4-C, ΔMocsn7a, and Mocsn7a-C. The strains were cultivated in an incubator at 25 ℃ for 9 days and observed under an optical microscope. (D) Disease spots of detached barley leaves inoculated with mycelial plugs from 70-15, ΔMocsn1, Mocsn1-C, ΔMocsn2, Mocsn2-C, ΔMocsn3, Mocsn3-C, ΔMocsn4, Mocsn4-C, ΔMocsn7a, and Mocsn7a-C. Leaves were cultured at 25 ℃ for 4 days after inoculation. Figure S3. CSN subunits are involved in the regulation of autophagy in M. oryzae. (A) Yeast two-hybrid assays were used to detect the interactions between CSN subunits and Atg-related proteins. Pairs of pGBKT7-53 and PGADT7-T were used as positive controls. Yeast transformants carrying the indicated constructs were plated onto selective plates supplemented without Leu/Trp or Leu/Trp/His/Ade for growth assays. (B) Autophagic flux analysis of GFP-MoAtg8 in 70-15 and ΔMo [file 12964_2024_1598_MOESM1_ESM.zip › Supplementary files/Figure3I-p-Osm1.tif]

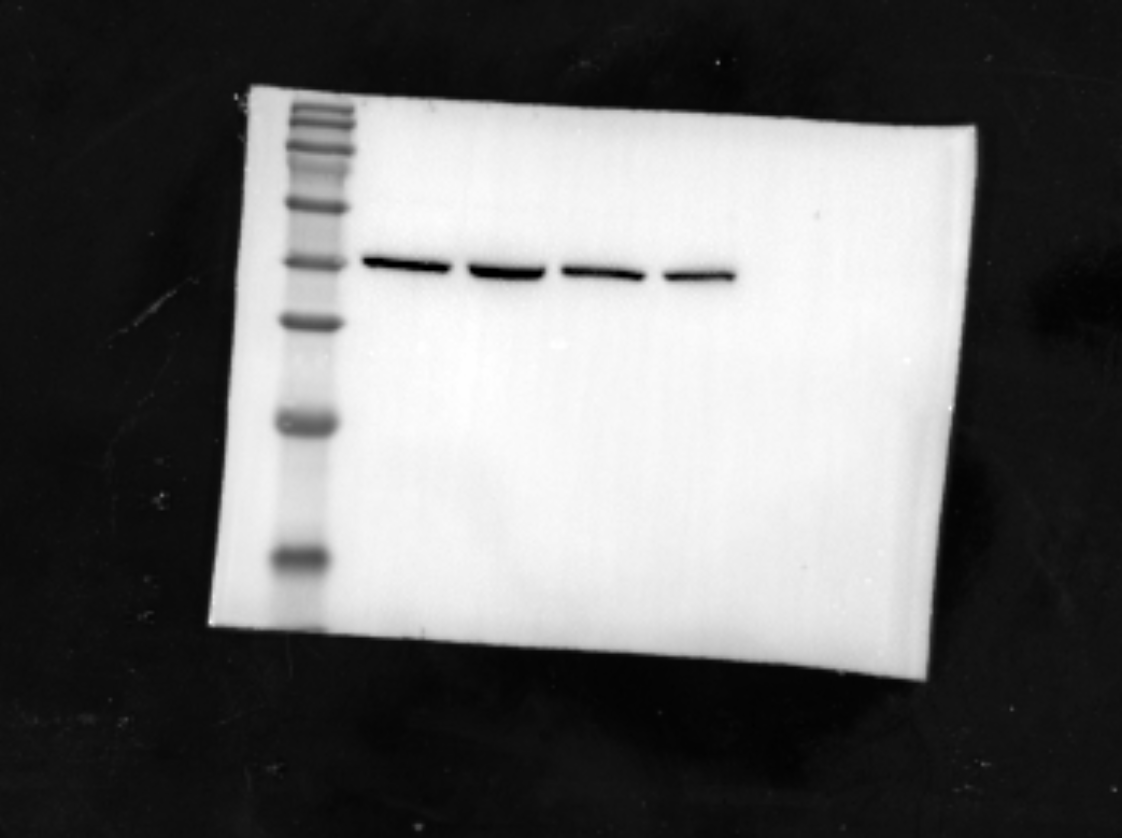

Supplement: Supplementary file 1 — Additional file 1: Figure S1. Verification of deletion mutants by PCR and quantitative real-time PCR. (A) Rice blast fungus knockout model. (B) PCR detection was performed on the null mutants, and the recombinant fragment (LF) was cloned from the positive strain but not from the WT strain. The 500-bp characteristic fragment (SF) of the target gene was cloned in the WT strain but not in the mutants. TUBULIN was used as a positive control. (C) The copy number of the resistance gene HPH in the deletion mutants was verified by quantitative real-time PCR. Figure S2. The targeted gene destruction of CSN subunits decreased the growth, sporulation, and pathogenicity of M. oryzae. (A) Colony morphology of 70-15, ΔMocsn1, Mocsn1-C, ΔMocsn2, Mocsn2-C, ΔMocsn3, Mocsn3-C, ΔMocsn4, Mocsn4-C, ΔMocsn7a, and Mocsn7a-C. The strains were grown on CM and MM plates for 9 days. (B) Statistical analysis of the colony growth diameter. The data were analyzed using GraphPad Prism 8.0 software. The error bars represent the standard deviations. ***P < 0.001. (C) Conidiophores of 70-15, ΔMocsn1, Mocsn1-C, ΔMocsn2, Mocsn2-C, ΔMocsn3, Mocsn3-C, ΔMocsn4, Mocsn4-C, ΔMocsn7a, and Mocsn7a-C. The strains were cultivated in an incubator at 25 ℃ for 9 days and observed under an optical microscope. (D) Disease spots of detached barley leaves inoculated with mycelial plugs from 70-15, ΔMocsn1, Mocsn1-C, ΔMocsn2, Mocsn2-C, ΔMocsn3, Mocsn3-C, ΔMocsn4, Mocsn4-C, ΔMocsn7a, and Mocsn7a-C. Leaves were cultured at 25 ℃ for 4 days after inoculation. Figure S3. CSN subunits are involved in the regulation of autophagy in M. oryzae. (A) Yeast two-hybrid assays were used to detect the interactions between CSN subunits and Atg-related proteins. Pairs of pGBKT7-53 and PGADT7-T were used as positive controls. Yeast transformants carrying the indicated constructs were plated onto selective plates supplemented without Leu/Trp or Leu/Trp/His/Ade for growth assays. (B) Autophagic flux analysis of GFP-MoAtg8 in 70-15 and ΔMo [file 12964_2024_1598_MOESM1_ESM.zip › Supplementary files/Figure4A-Actin.tif]

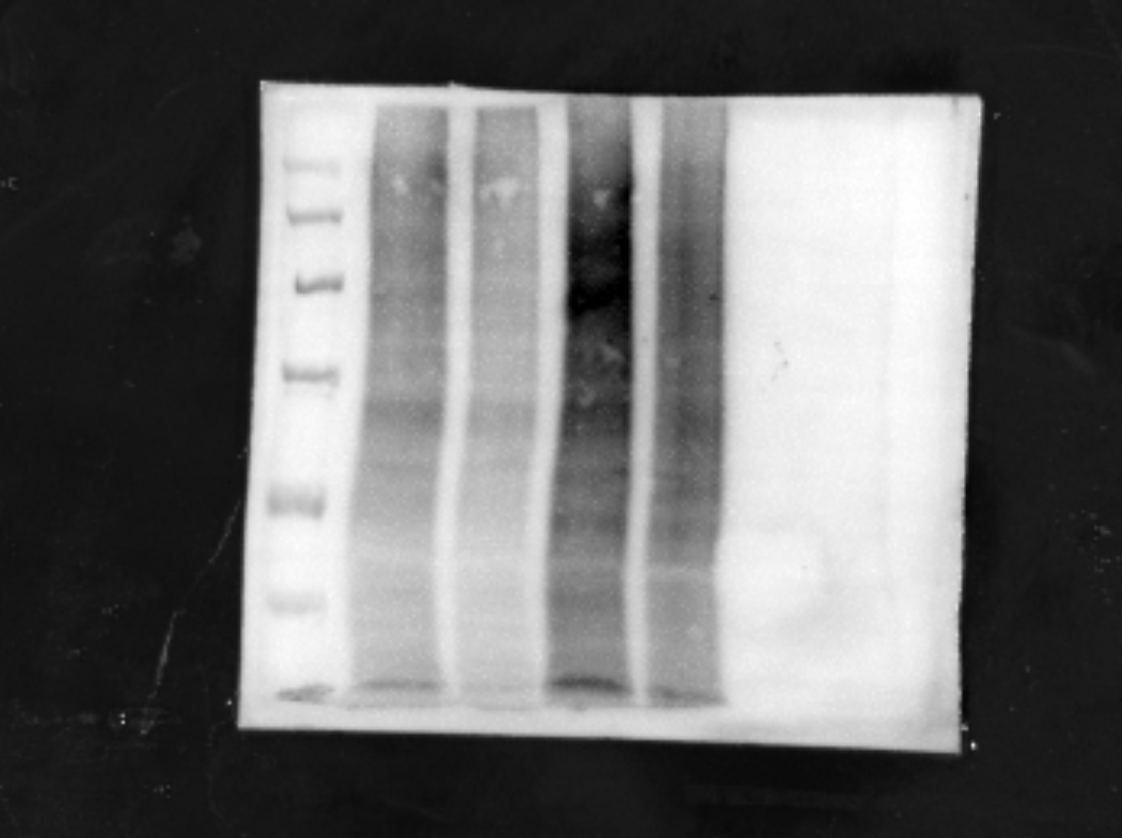

Supplement: Supplementary file 1 — Additional file 1: Figure S1. Verification of deletion mutants by PCR and quantitative real-time PCR. (A) Rice blast fungus knockout model. (B) PCR detection was performed on the null mutants, and the recombinant fragment (LF) was cloned from the positive strain but not from the WT strain. The 500-bp characteristic fragment (SF) of the target gene was cloned in the WT strain but not in the mutants. TUBULIN was used as a positive control. (C) The copy number of the resistance gene HPH in the deletion mutants was verified by quantitative real-time PCR. Figure S2. The targeted gene destruction of CSN subunits decreased the growth, sporulation, and pathogenicity of M. oryzae. (A) Colony morphology of 70-15, ΔMocsn1, Mocsn1-C, ΔMocsn2, Mocsn2-C, ΔMocsn3, Mocsn3-C, ΔMocsn4, Mocsn4-C, ΔMocsn7a, and Mocsn7a-C. The strains were grown on CM and MM plates for 9 days. (B) Statistical analysis of the colony growth diameter. The data were analyzed using GraphPad Prism 8.0 software. The error bars represent the standard deviations. ***P < 0.001. (C) Conidiophores of 70-15, ΔMocsn1, Mocsn1-C, ΔMocsn2, Mocsn2-C, ΔMocsn3, Mocsn3-C, ΔMocsn4, Mocsn4-C, ΔMocsn7a, and Mocsn7a-C. The strains were cultivated in an incubator at 25 ℃ for 9 days and observed under an optical microscope. (D) Disease spots of detached barley leaves inoculated with mycelial plugs from 70-15, ΔMocsn1, Mocsn1-C, ΔMocsn2, Mocsn2-C, ΔMocsn3, Mocsn3-C, ΔMocsn4, Mocsn4-C, ΔMocsn7a, and Mocsn7a-C. Leaves were cultured at 25 ℃ for 4 days after inoculation. Figure S3. CSN subunits are involved in the regulation of autophagy in M. oryzae. (A) Yeast two-hybrid assays were used to detect the interactions between CSN subunits and Atg-related proteins. Pairs of pGBKT7-53 and PGADT7-T were used as positive controls. Yeast transformants carrying the indicated constructs were plated onto selective plates supplemented without Leu/Trp or Leu/Trp/His/Ade for growth assays. (B) Autophagic flux analysis of GFP-MoAtg8 in 70-15 and ΔMo [file 12964_2024_1598_MOESM1_ESM.zip › Supplementary files/Figure4A-Ubi.tif]

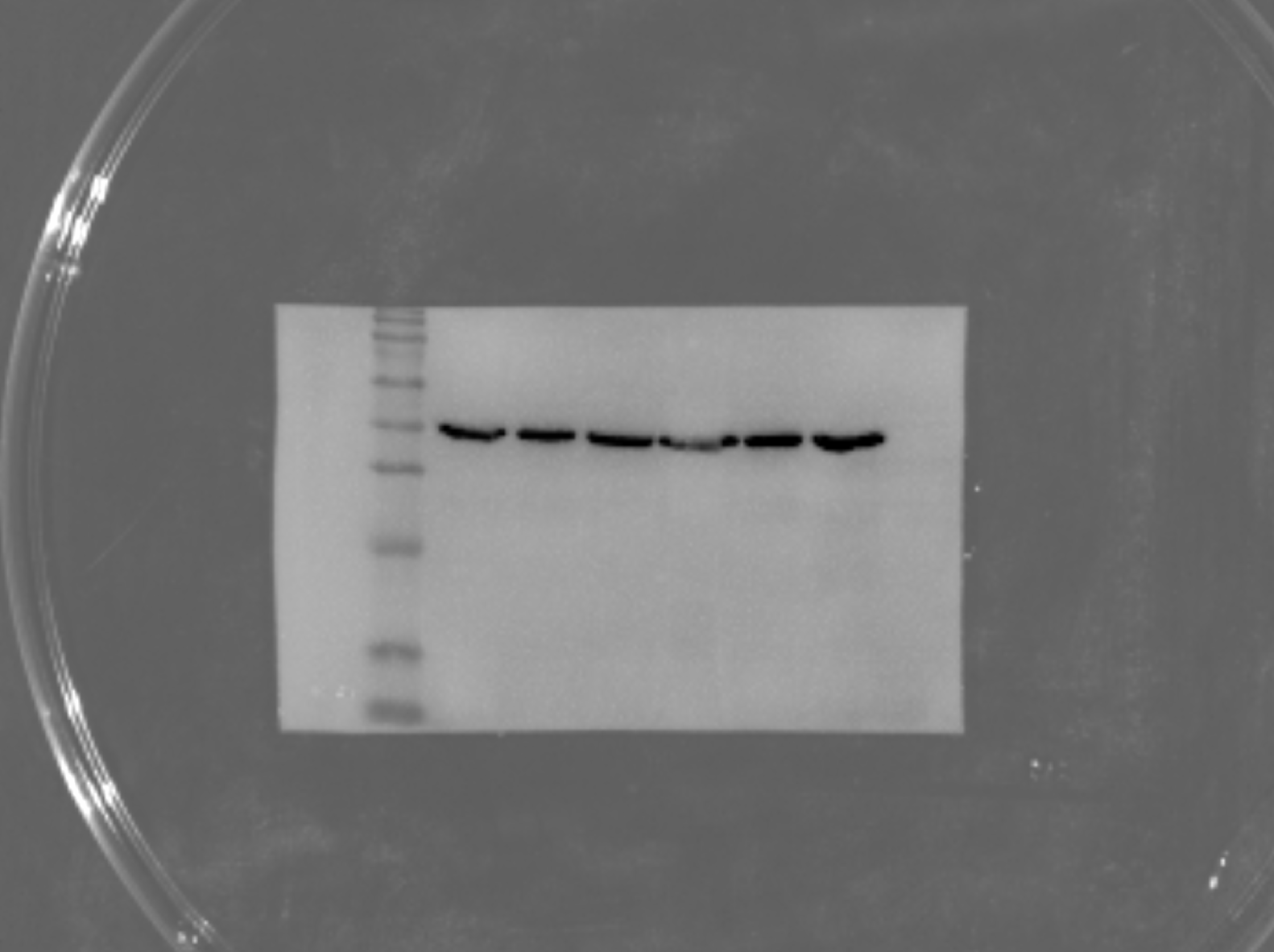

Supplement: Supplementary file 1 — Additional file 1: Figure S1. Verification of deletion mutants by PCR and quantitative real-time PCR. (A) Rice blast fungus knockout model. (B) PCR detection was performed on the null mutants, and the recombinant fragment (LF) was cloned from the positive strain but not from the WT strain. The 500-bp characteristic fragment (SF) of the target gene was cloned in the WT strain but not in the mutants. TUBULIN was used as a positive control. (C) The copy number of the resistance gene HPH in the deletion mutants was verified by quantitative real-time PCR. Figure S2. The targeted gene destruction of CSN subunits decreased the growth, sporulation, and pathogenicity of M. oryzae. (A) Colony morphology of 70-15, ΔMocsn1, Mocsn1-C, ΔMocsn2, Mocsn2-C, ΔMocsn3, Mocsn3-C, ΔMocsn4, Mocsn4-C, ΔMocsn7a, and Mocsn7a-C. The strains were grown on CM and MM plates for 9 days. (B) Statistical analysis of the colony growth diameter. The data were analyzed using GraphPad Prism 8.0 software. The error bars represent the standard deviations. ***P < 0.001. (C) Conidiophores of 70-15, ΔMocsn1, Mocsn1-C, ΔMocsn2, Mocsn2-C, ΔMocsn3, Mocsn3-C, ΔMocsn4, Mocsn4-C, ΔMocsn7a, and Mocsn7a-C. The strains were cultivated in an incubator at 25 ℃ for 9 days and observed under an optical microscope. (D) Disease spots of detached barley leaves inoculated with mycelial plugs from 70-15, ΔMocsn1, Mocsn1-C, ΔMocsn2, Mocsn2-C, ΔMocsn3, Mocsn3-C, ΔMocsn4, Mocsn4-C, ΔMocsn7a, and Mocsn7a-C. Leaves were cultured at 25 ℃ for 4 days after inoculation. Figure S3. CSN subunits are involved in the regulation of autophagy in M. oryzae. (A) Yeast two-hybrid assays were used to detect the interactions between CSN subunits and Atg-related proteins. Pairs of pGBKT7-53 and PGADT7-T were used as positive controls. Yeast transformants carrying the indicated constructs were plated onto selective plates supplemented without Leu/Trp or Leu/Trp/His/Ade for growth assays. (B) Autophagic flux analysis of GFP-MoAtg8 in 70-15 and ΔMo [file 12964_2024_1598_MOESM1_ESM.zip › Supplementary files/Figure4B-Actin.tif]

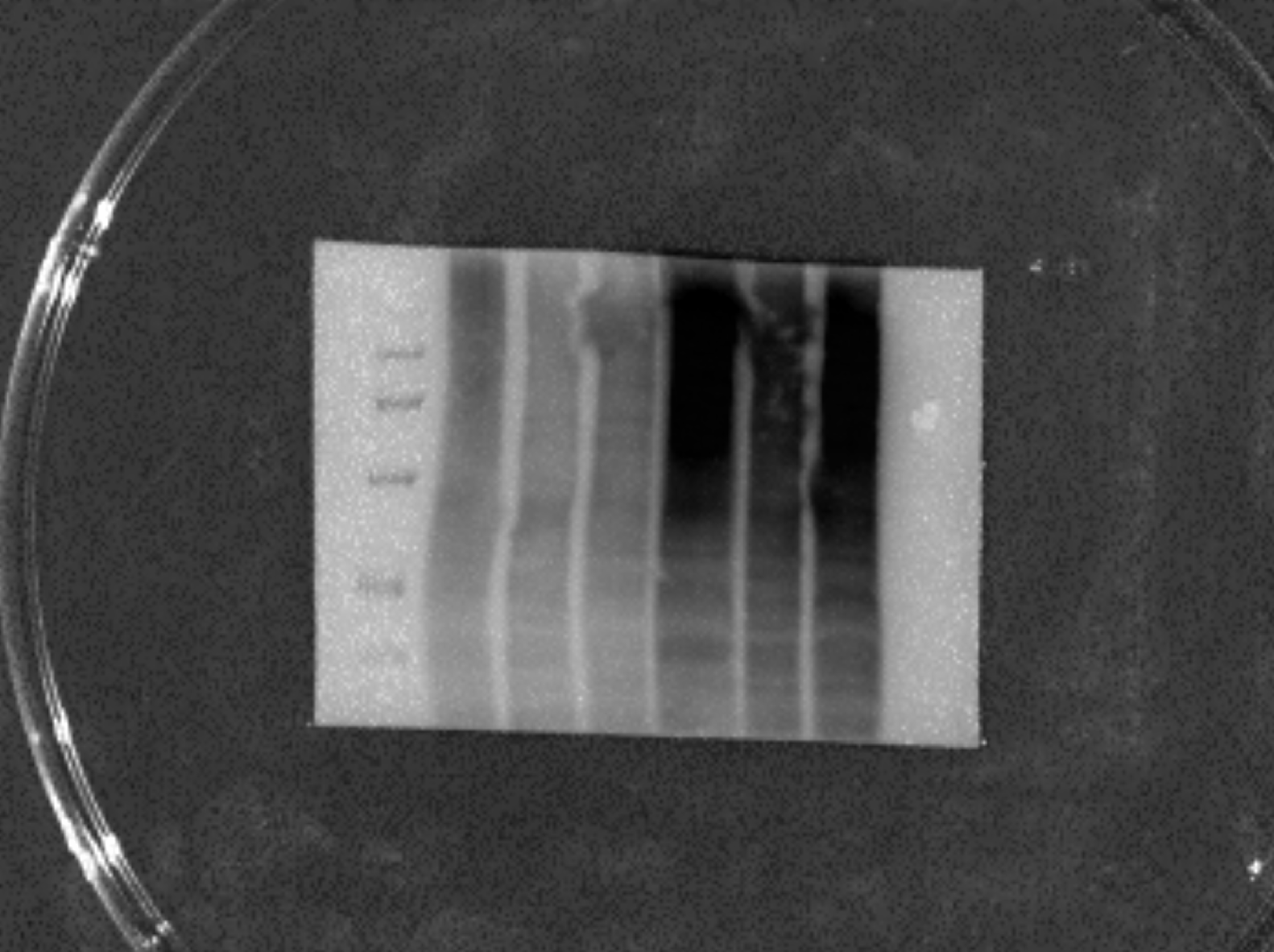

Supplement: Supplementary file 1 — Additional file 1: Figure S1. Verification of deletion mutants by PCR and quantitative real-time PCR. (A) Rice blast fungus knockout model. (B) PCR detection was performed on the null mutants, and the recombinant fragment (LF) was cloned from the positive strain but not from the WT strain. The 500-bp characteristic fragment (SF) of the target gene was cloned in the WT strain but not in the mutants. TUBULIN was used as a positive control. (C) The copy number of the resistance gene HPH in the deletion mutants was verified by quantitative real-time PCR. Figure S2. The targeted gene destruction of CSN subunits decreased the growth, sporulation, and pathogenicity of M. oryzae. (A) Colony morphology of 70-15, ΔMocsn1, Mocsn1-C, ΔMocsn2, Mocsn2-C, ΔMocsn3, Mocsn3-C, ΔMocsn4, Mocsn4-C, ΔMocsn7a, and Mocsn7a-C. The strains were grown on CM and MM plates for 9 days. (B) Statistical analysis of the colony growth diameter. The data were analyzed using GraphPad Prism 8.0 software. The error bars represent the standard deviations. ***P < 0.001. (C) Conidiophores of 70-15, ΔMocsn1, Mocsn1-C, ΔMocsn2, Mocsn2-C, ΔMocsn3, Mocsn3-C, ΔMocsn4, Mocsn4-C, ΔMocsn7a, and Mocsn7a-C. The strains were cultivated in an incubator at 25 ℃ for 9 days and observed under an optical microscope. (D) Disease spots of detached barley leaves inoculated with mycelial plugs from 70-15, ΔMocsn1, Mocsn1-C, ΔMocsn2, Mocsn2-C, ΔMocsn3, Mocsn3-C, ΔMocsn4, Mocsn4-C, ΔMocsn7a, and Mocsn7a-C. Leaves were cultured at 25 ℃ for 4 days after inoculation. Figure S3. CSN subunits are involved in the regulation of autophagy in M. oryzae. (A) Yeast two-hybrid assays were used to detect the interactions between CSN subunits and Atg-related proteins. Pairs of pGBKT7-53 and PGADT7-T were used as positive controls. Yeast transformants carrying the indicated constructs were plated onto selective plates supplemented without Leu/Trp or Leu/Trp/His/Ade for growth assays. (B) Autophagic flux analysis of GFP-MoAtg8 in 70-15 and ΔMo [file 12964_2024_1598_MOESM1_ESM.zip › Supplementary files/Figure4B-Ubi.tif]

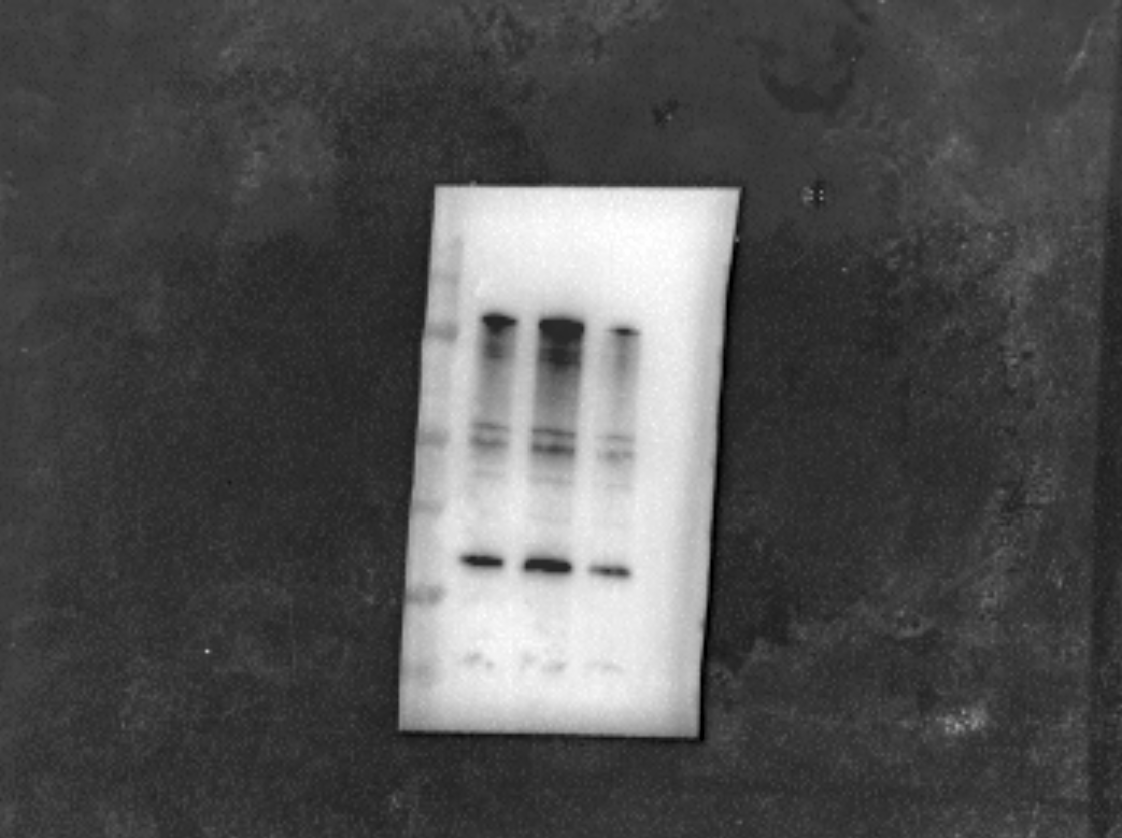

Supplement: Supplementary file 1 — Additional file 1: Figure S1. Verification of deletion mutants by PCR and quantitative real-time PCR. (A) Rice blast fungus knockout model. (B) PCR detection was performed on the null mutants, and the recombinant fragment (LF) was cloned from the positive strain but not from the WT strain. The 500-bp characteristic fragment (SF) of the target gene was cloned in the WT strain but not in the mutants. TUBULIN was used as a positive control. (C) The copy number of the resistance gene HPH in the deletion mutants was verified by quantitative real-time PCR. Figure S2. The targeted gene destruction of CSN subunits decreased the growth, sporulation, and pathogenicity of M. oryzae. (A) Colony morphology of 70-15, ΔMocsn1, Mocsn1-C, ΔMocsn2, Mocsn2-C, ΔMocsn3, Mocsn3-C, ΔMocsn4, Mocsn4-C, ΔMocsn7a, and Mocsn7a-C. The strains were grown on CM and MM plates for 9 days. (B) Statistical analysis of the colony growth diameter. The data were analyzed using GraphPad Prism 8.0 software. The error bars represent the standard deviations. ***P < 0.001. (C) Conidiophores of 70-15, ΔMocsn1, Mocsn1-C, ΔMocsn2, Mocsn2-C, ΔMocsn3, Mocsn3-C, ΔMocsn4, Mocsn4-C, ΔMocsn7a, and Mocsn7a-C. The strains were cultivated in an incubator at 25 ℃ for 9 days and observed under an optical microscope. (D) Disease spots of detached barley leaves inoculated with mycelial plugs from 70-15, ΔMocsn1, Mocsn1-C, ΔMocsn2, Mocsn2-C, ΔMocsn3, Mocsn3-C, ΔMocsn4, Mocsn4-C, ΔMocsn7a, and Mocsn7a-C. Leaves were cultured at 25 ℃ for 4 days after inoculation. Figure S3. CSN subunits are involved in the regulation of autophagy in M. oryzae. (A) Yeast two-hybrid assays were used to detect the interactions between CSN subunits and Atg-related proteins. Pairs of pGBKT7-53 and PGADT7-T were used as positive controls. Yeast transformants carrying the indicated constructs were plated onto selective plates supplemented without Leu/Trp or Leu/Trp/His/Ade for growth assays. (B) Autophagic flux analysis of GFP-MoAtg8 in 70-15 and ΔMo [file 12964_2024_1598_MOESM1_ESM.zip › Supplementary files/Figure4H-Flag.tif]

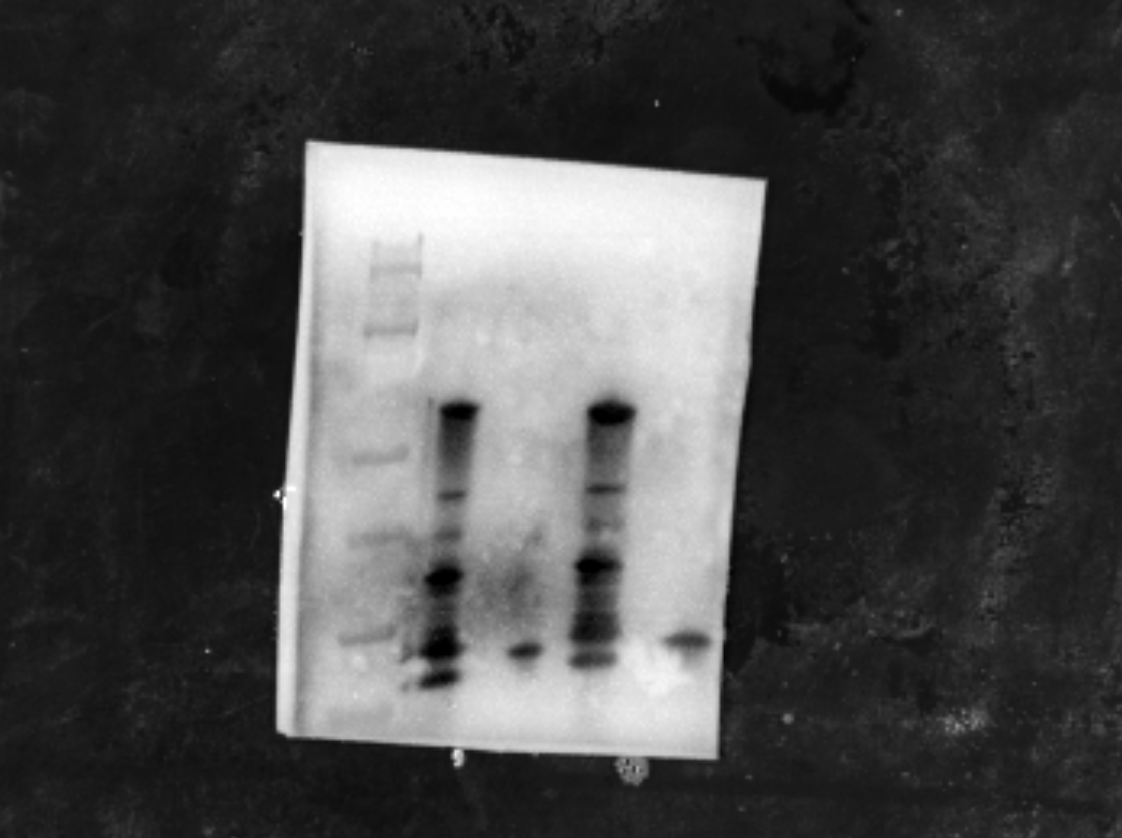

Supplement: Supplementary file 1 — Additional file 1: Figure S1. Verification of deletion mutants by PCR and quantitative real-time PCR. (A) Rice blast fungus knockout model. (B) PCR detection was performed on the null mutants, and the recombinant fragment (LF) was cloned from the positive strain but not from the WT strain. The 500-bp characteristic fragment (SF) of the target gene was cloned in the WT strain but not in the mutants. TUBULIN was used as a positive control. (C) The copy number of the resistance gene HPH in the deletion mutants was verified by quantitative real-time PCR. Figure S2. The targeted gene destruction of CSN subunits decreased the growth, sporulation, and pathogenicity of M. oryzae. (A) Colony morphology of 70-15, ΔMocsn1, Mocsn1-C, ΔMocsn2, Mocsn2-C, ΔMocsn3, Mocsn3-C, ΔMocsn4, Mocsn4-C, ΔMocsn7a, and Mocsn7a-C. The strains were grown on CM and MM plates for 9 days. (B) Statistical analysis of the colony growth diameter. The data were analyzed using GraphPad Prism 8.0 software. The error bars represent the standard deviations. ***P < 0.001. (C) Conidiophores of 70-15, ΔMocsn1, Mocsn1-C, ΔMocsn2, Mocsn2-C, ΔMocsn3, Mocsn3-C, ΔMocsn4, Mocsn4-C, ΔMocsn7a, and Mocsn7a-C. The strains were cultivated in an incubator at 25 ℃ for 9 days and observed under an optical microscope. (D) Disease spots of detached barley leaves inoculated with mycelial plugs from 70-15, ΔMocsn1, Mocsn1-C, ΔMocsn2, Mocsn2-C, ΔMocsn3, Mocsn3-C, ΔMocsn4, Mocsn4-C, ΔMocsn7a, and Mocsn7a-C. Leaves were cultured at 25 ℃ for 4 days after inoculation. Figure S3. CSN subunits are involved in the regulation of autophagy in M. oryzae. (A) Yeast two-hybrid assays were used to detect the interactions between CSN subunits and Atg-related proteins. Pairs of pGBKT7-53 and PGADT7-T were used as positive controls. Yeast transformants carrying the indicated constructs were plated onto selective plates supplemented without Leu/Trp or Leu/Trp/His/Ade for growth assays. (B) Autophagic flux analysis of GFP-MoAtg8 in 70-15 and ΔMo [file 12964_2024_1598_MOESM1_ESM.zip › Supplementary files/Figure4H-GFP.tif]

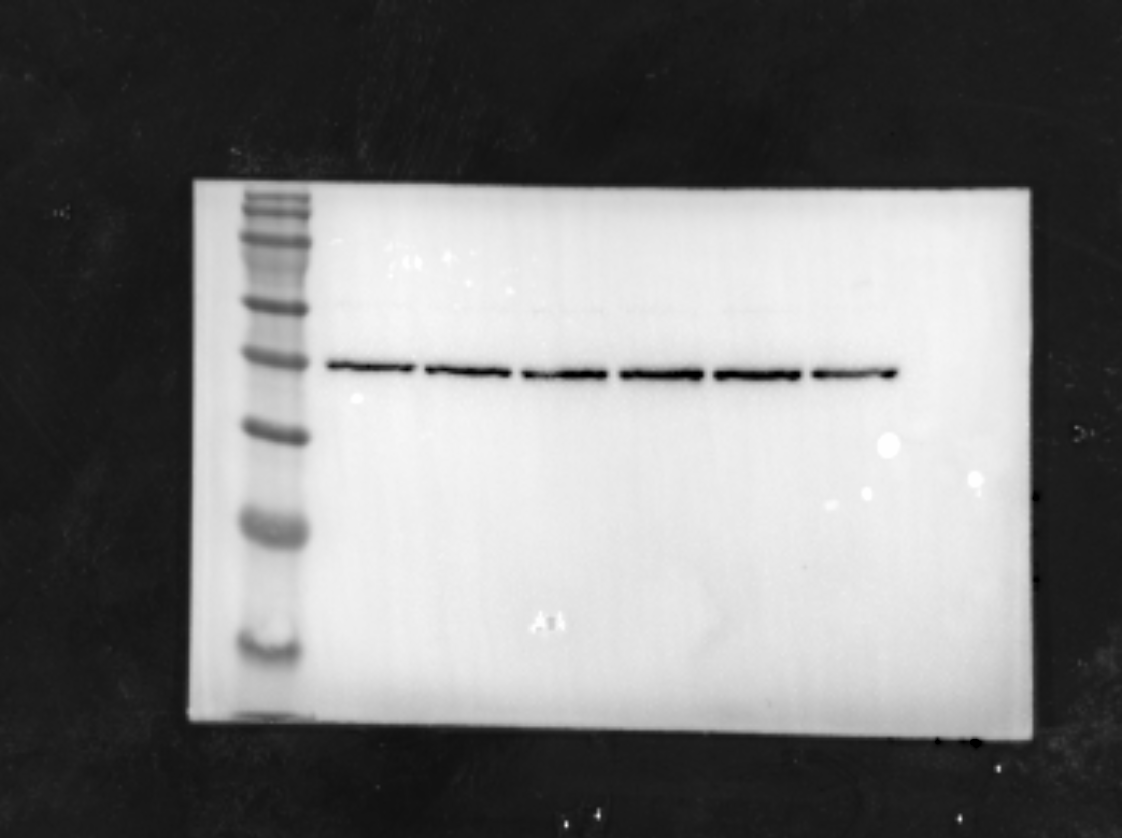

Supplement: Supplementary file 1 — Additional file 1: Figure S1. Verification of deletion mutants by PCR and quantitative real-time PCR. (A) Rice blast fungus knockout model. (B) PCR detection was performed on the null mutants, and the recombinant fragment (LF) was cloned from the positive strain but not from the WT strain. The 500-bp characteristic fragment (SF) of the target gene was cloned in the WT strain but not in the mutants. TUBULIN was used as a positive control. (C) The copy number of the resistance gene HPH in the deletion mutants was verified by quantitative real-time PCR. Figure S2. The targeted gene destruction of CSN subunits decreased the growth, sporulation, and pathogenicity of M. oryzae. (A) Colony morphology of 70-15, ΔMocsn1, Mocsn1-C, ΔMocsn2, Mocsn2-C, ΔMocsn3, Mocsn3-C, ΔMocsn4, Mocsn4-C, ΔMocsn7a, and Mocsn7a-C. The strains were grown on CM and MM plates for 9 days. (B) Statistical analysis of the colony growth diameter. The data were analyzed using GraphPad Prism 8.0 software. The error bars represent the standard deviations. ***P < 0.001. (C) Conidiophores of 70-15, ΔMocsn1, Mocsn1-C, ΔMocsn2, Mocsn2-C, ΔMocsn3, Mocsn3-C, ΔMocsn4, Mocsn4-C, ΔMocsn7a, and Mocsn7a-C. The strains were cultivated in an incubator at 25 ℃ for 9 days and observed under an optical microscope. (D) Disease spots of detached barley leaves inoculated with mycelial plugs from 70-15, ΔMocsn1, Mocsn1-C, ΔMocsn2, Mocsn2-C, ΔMocsn3, Mocsn3-C, ΔMocsn4, Mocsn4-C, ΔMocsn7a, and Mocsn7a-C. Leaves were cultured at 25 ℃ for 4 days after inoculation. Figure S3. CSN subunits are involved in the regulation of autophagy in M. oryzae. (A) Yeast two-hybrid assays were used to detect the interactions between CSN subunits and Atg-related proteins. Pairs of pGBKT7-53 and PGADT7-T were used as positive controls. Yeast transformants carrying the indicated constructs were plated onto selective plates supplemented without Leu/Trp or Leu/Trp/His/Ade for growth assays. (B) Autophagic flux analysis of GFP-MoAtg8 in 70-15 and ΔMo [file 12964_2024_1598_MOESM1_ESM.zip › Supplementary files/Figure5B-Actin.tif]

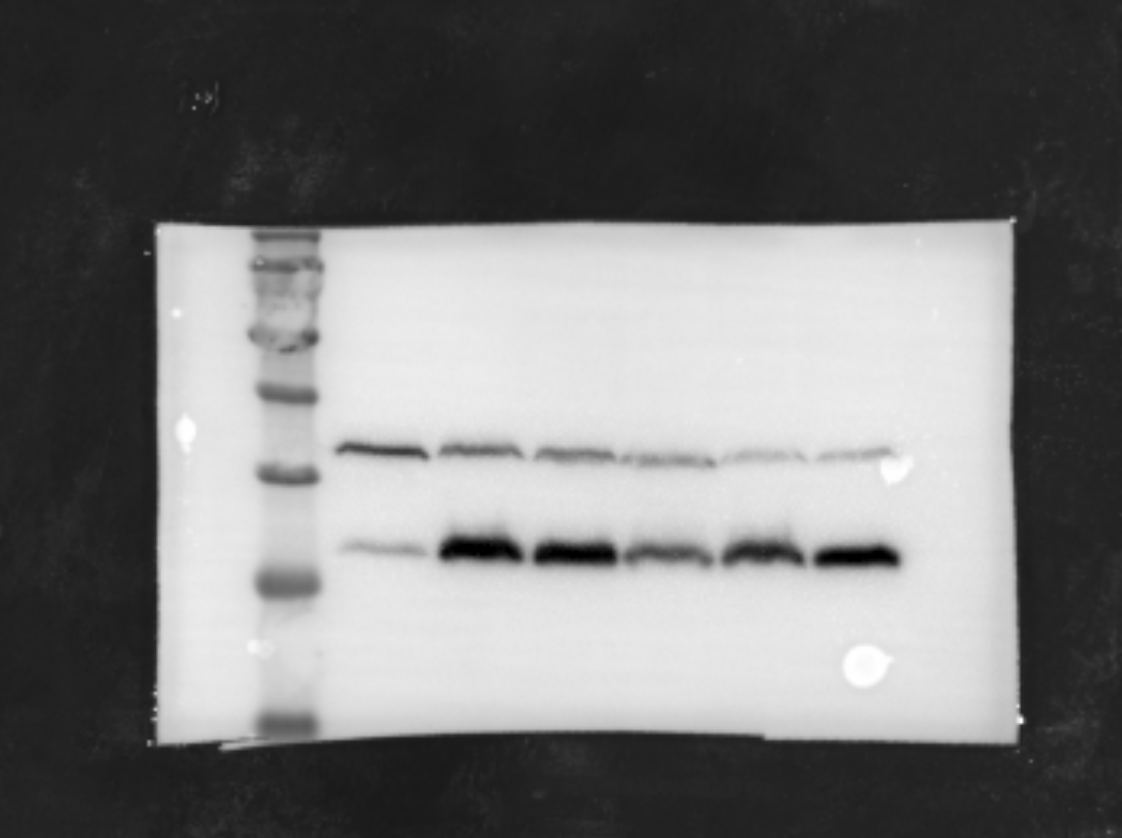

Supplement: Supplementary file 1 — Additional file 1: Figure S1. Verification of deletion mutants by PCR and quantitative real-time PCR. (A) Rice blast fungus knockout model. (B) PCR detection was performed on the null mutants, and the recombinant fragment (LF) was cloned from the positive strain but not from the WT strain. The 500-bp characteristic fragment (SF) of the target gene was cloned in the WT strain but not in the mutants. TUBULIN was used as a positive control. (C) The copy number of the resistance gene HPH in the deletion mutants was verified by quantitative real-time PCR. Figure S2. The targeted gene destruction of CSN subunits decreased the growth, sporulation, and pathogenicity of M. oryzae. (A) Colony morphology of 70-15, ΔMocsn1, Mocsn1-C, ΔMocsn2, Mocsn2-C, ΔMocsn3, Mocsn3-C, ΔMocsn4, Mocsn4-C, ΔMocsn7a, and Mocsn7a-C. The strains were grown on CM and MM plates for 9 days. (B) Statistical analysis of the colony growth diameter. The data were analyzed using GraphPad Prism 8.0 software. The error bars represent the standard deviations. ***P < 0.001. (C) Conidiophores of 70-15, ΔMocsn1, Mocsn1-C, ΔMocsn2, Mocsn2-C, ΔMocsn3, Mocsn3-C, ΔMocsn4, Mocsn4-C, ΔMocsn7a, and Mocsn7a-C. The strains were cultivated in an incubator at 25 ℃ for 9 days and observed under an optical microscope. (D) Disease spots of detached barley leaves inoculated with mycelial plugs from 70-15, ΔMocsn1, Mocsn1-C, ΔMocsn2, Mocsn2-C, ΔMocsn3, Mocsn3-C, ΔMocsn4, Mocsn4-C, ΔMocsn7a, and Mocsn7a-C. Leaves were cultured at 25 ℃ for 4 days after inoculation. Figure S3. CSN subunits are involved in the regulation of autophagy in M. oryzae. (A) Yeast two-hybrid assays were used to detect the interactions between CSN subunits and Atg-related proteins. Pairs of pGBKT7-53 and PGADT7-T were used as positive controls. Yeast transformants carrying the indicated constructs were plated onto selective plates supplemented without Leu/Trp or Leu/Trp/His/Ade for growth assays. (B) Autophagic flux analysis of GFP-MoAtg8 in 70-15 and ΔMo [file 12964_2024_1598_MOESM1_ESM.zip › Supplementary files/Figure5B-GFP.tif]

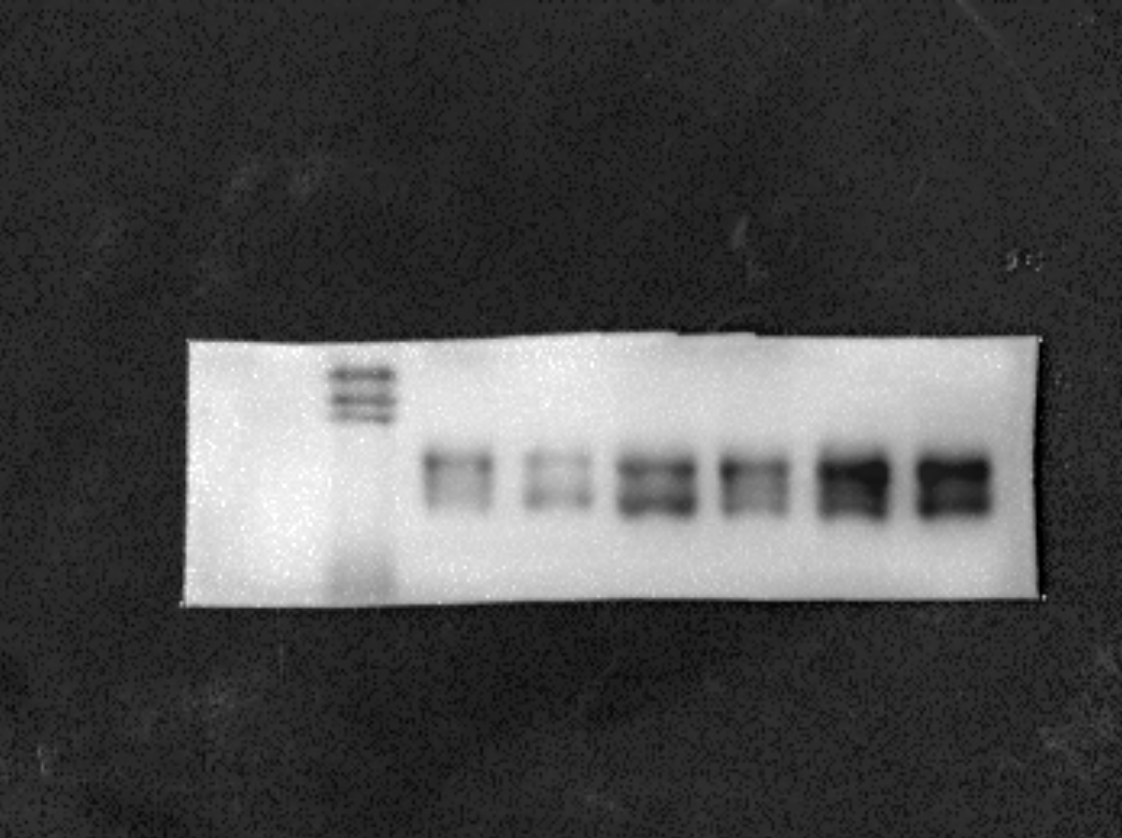

Supplement: Supplementary file 1 — Additional file 1: Figure S1. Verification of deletion mutants by PCR and quantitative real-time PCR. (A) Rice blast fungus knockout model. (B) PCR detection was performed on the null mutants, and the recombinant fragment (LF) was cloned from the positive strain but not from the WT strain. The 500-bp characteristic fragment (SF) of the target gene was cloned in the WT strain but not in the mutants. TUBULIN was used as a positive control. (C) The copy number of the resistance gene HPH in the deletion mutants was verified by quantitative real-time PCR. Figure S2. The targeted gene destruction of CSN subunits decreased the growth, sporulation, and pathogenicity of M. oryzae. (A) Colony morphology of 70-15, ΔMocsn1, Mocsn1-C, ΔMocsn2, Mocsn2-C, ΔMocsn3, Mocsn3-C, ΔMocsn4, Mocsn4-C, ΔMocsn7a, and Mocsn7a-C. The strains were grown on CM and MM plates for 9 days. (B) Statistical analysis of the colony growth diameter. The data were analyzed using GraphPad Prism 8.0 software. The error bars represent the standard deviations. ***P < 0.001. (C) Conidiophores of 70-15, ΔMocsn1, Mocsn1-C, ΔMocsn2, Mocsn2-C, ΔMocsn3, Mocsn3-C, ΔMocsn4, Mocsn4-C, ΔMocsn7a, and Mocsn7a-C. The strains were cultivated in an incubator at 25 ℃ for 9 days and observed under an optical microscope. (D) Disease spots of detached barley leaves inoculated with mycelial plugs from 70-15, ΔMocsn1, Mocsn1-C, ΔMocsn2, Mocsn2-C, ΔMocsn3, Mocsn3-C, ΔMocsn4, Mocsn4-C, ΔMocsn7a, and Mocsn7a-C. Leaves were cultured at 25 ℃ for 4 days after inoculation. Figure S3. CSN subunits are involved in the regulation of autophagy in M. oryzae. (A) Yeast two-hybrid assays were used to detect the interactions between CSN subunits and Atg-related proteins. Pairs of pGBKT7-53 and PGADT7-T were used as positive controls. Yeast transformants carrying the indicated constructs were plated onto selective plates supplemented without Leu/Trp or Leu/Trp/His/Ade for growth assays. (B) Autophagic flux analysis of GFP-MoAtg8 in 70-15 and ΔMo [file 12964_2024_1598_MOESM1_ESM.zip › Supplementary files/Figure5C-A8PE.tif]

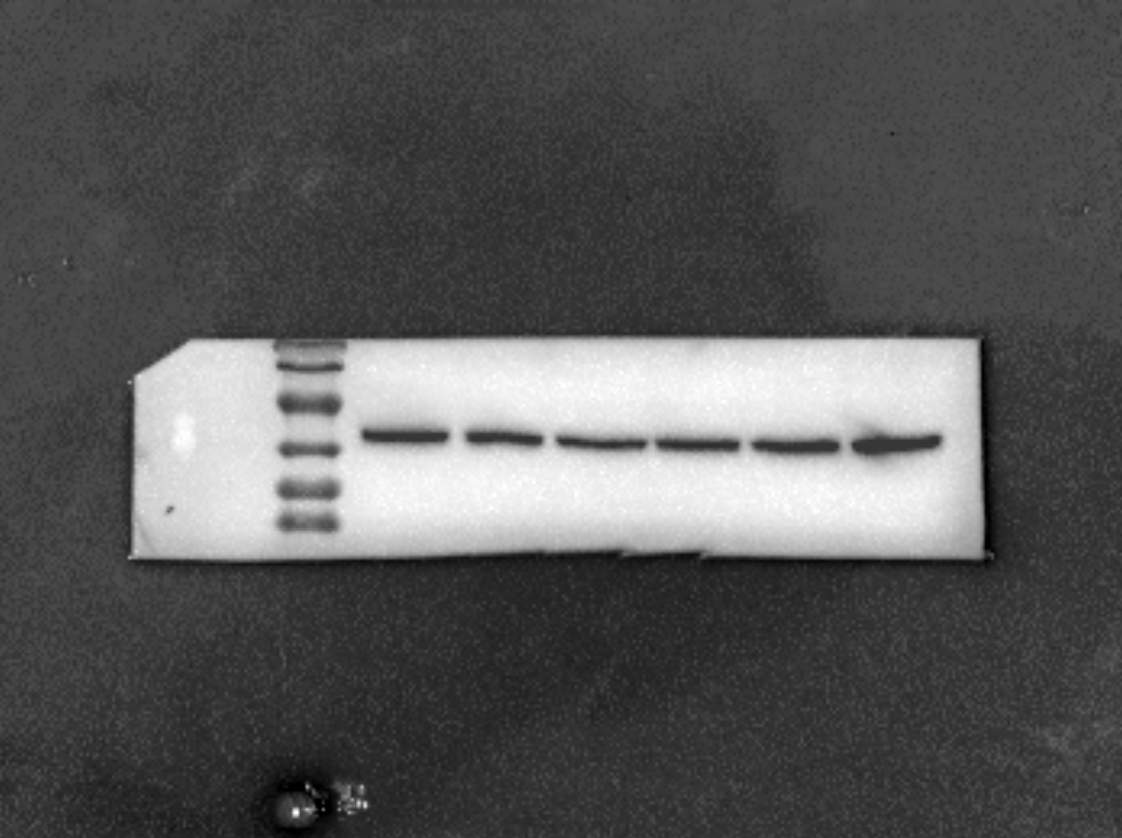

Supplement: Supplementary file 1 — Additional file 1: Figure S1. Verification of deletion mutants by PCR and quantitative real-time PCR. (A) Rice blast fungus knockout model. (B) PCR detection was performed on the null mutants, and the recombinant fragment (LF) was cloned from the positive strain but not from the WT strain. The 500-bp characteristic fragment (SF) of the target gene was cloned in the WT strain but not in the mutants. TUBULIN was used as a positive control. (C) The copy number of the resistance gene HPH in the deletion mutants was verified by quantitative real-time PCR. Figure S2. The targeted gene destruction of CSN subunits decreased the growth, sporulation, and pathogenicity of M. oryzae. (A) Colony morphology of 70-15, ΔMocsn1, Mocsn1-C, ΔMocsn2, Mocsn2-C, ΔMocsn3, Mocsn3-C, ΔMocsn4, Mocsn4-C, ΔMocsn7a, and Mocsn7a-C. The strains were grown on CM and MM plates for 9 days. (B) Statistical analysis of the colony growth diameter. The data were analyzed using GraphPad Prism 8.0 software. The error bars represent the standard deviations. ***P < 0.001. (C) Conidiophores of 70-15, ΔMocsn1, Mocsn1-C, ΔMocsn2, Mocsn2-C, ΔMocsn3, Mocsn3-C, ΔMocsn4, Mocsn4-C, ΔMocsn7a, and Mocsn7a-C. The strains were cultivated in an incubator at 25 ℃ for 9 days and observed under an optical microscope. (D) Disease spots of detached barley leaves inoculated with mycelial plugs from 70-15, ΔMocsn1, Mocsn1-C, ΔMocsn2, Mocsn2-C, ΔMocsn3, Mocsn3-C, ΔMocsn4, Mocsn4-C, ΔMocsn7a, and Mocsn7a-C. Leaves were cultured at 25 ℃ for 4 days after inoculation. Figure S3. CSN subunits are involved in the regulation of autophagy in M. oryzae. (A) Yeast two-hybrid assays were used to detect the interactions between CSN subunits and Atg-related proteins. Pairs of pGBKT7-53 and PGADT7-T were used as positive controls. Yeast transformants carrying the indicated constructs were plated onto selective plates supplemented without Leu/Trp or Leu/Trp/His/Ade for growth assays. (B) Autophagic flux analysis of GFP-MoAtg8 in 70-15 and ΔMo [file 12964_2024_1598_MOESM1_ESM.zip › Supplementary files/Figure5C-Actin.tif]

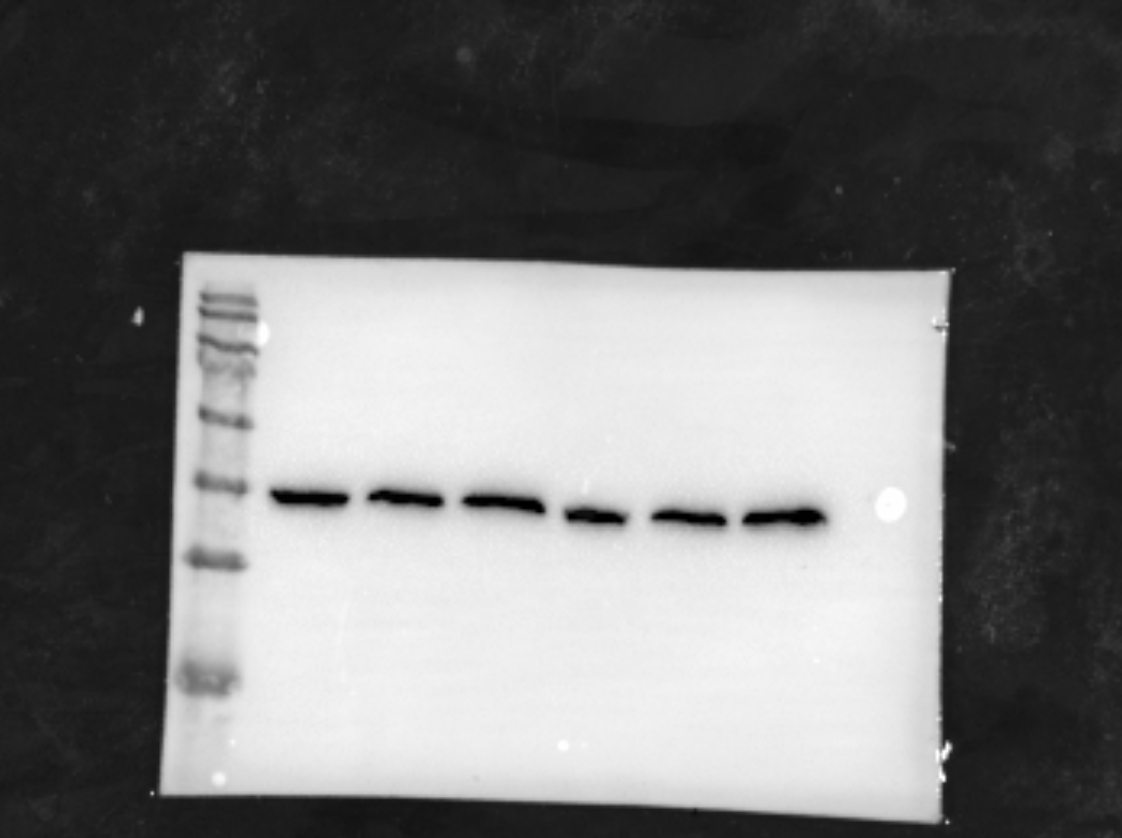

Supplement: Supplementary file 1 — Additional file 1: Figure S1. Verification of deletion mutants by PCR and quantitative real-time PCR. (A) Rice blast fungus knockout model. (B) PCR detection was performed on the null mutants, and the recombinant fragment (LF) was cloned from the positive strain but not from the WT strain. The 500-bp characteristic fragment (SF) of the target gene was cloned in the WT strain but not in the mutants. TUBULIN was used as a positive control. (C) The copy number of the resistance gene HPH in the deletion mutants was verified by quantitative real-time PCR. Figure S2. The targeted gene destruction of CSN subunits decreased the growth, sporulation, and pathogenicity of M. oryzae. (A) Colony morphology of 70-15, ΔMocsn1, Mocsn1-C, ΔMocsn2, Mocsn2-C, ΔMocsn3, Mocsn3-C, ΔMocsn4, Mocsn4-C, ΔMocsn7a, and Mocsn7a-C. The strains were grown on CM and MM plates for 9 days. (B) Statistical analysis of the colony growth diameter. The data were analyzed using GraphPad Prism 8.0 software. The error bars represent the standard deviations. ***P < 0.001. (C) Conidiophores of 70-15, ΔMocsn1, Mocsn1-C, ΔMocsn2, Mocsn2-C, ΔMocsn3, Mocsn3-C, ΔMocsn4, Mocsn4-C, ΔMocsn7a, and Mocsn7a-C. The strains were cultivated in an incubator at 25 ℃ for 9 days and observed under an optical microscope. (D) Disease spots of detached barley leaves inoculated with mycelial plugs from 70-15, ΔMocsn1, Mocsn1-C, ΔMocsn2, Mocsn2-C, ΔMocsn3, Mocsn3-C, ΔMocsn4, Mocsn4-C, ΔMocsn7a, and Mocsn7a-C. Leaves were cultured at 25 ℃ for 4 days after inoculation. Figure S3. CSN subunits are involved in the regulation of autophagy in M. oryzae. (A) Yeast two-hybrid assays were used to detect the interactions between CSN subunits and Atg-related proteins. Pairs of pGBKT7-53 and PGADT7-T were used as positive controls. Yeast transformants carrying the indicated constructs were plated onto selective plates supplemented without Leu/Trp or Leu/Trp/His/Ade for growth assays. (B) Autophagic flux analysis of GFP-MoAtg8 in 70-15 and ΔMo [file 12964_2024_1598_MOESM1_ESM.zip › Supplementary files/Figure5D-Actin.tif]

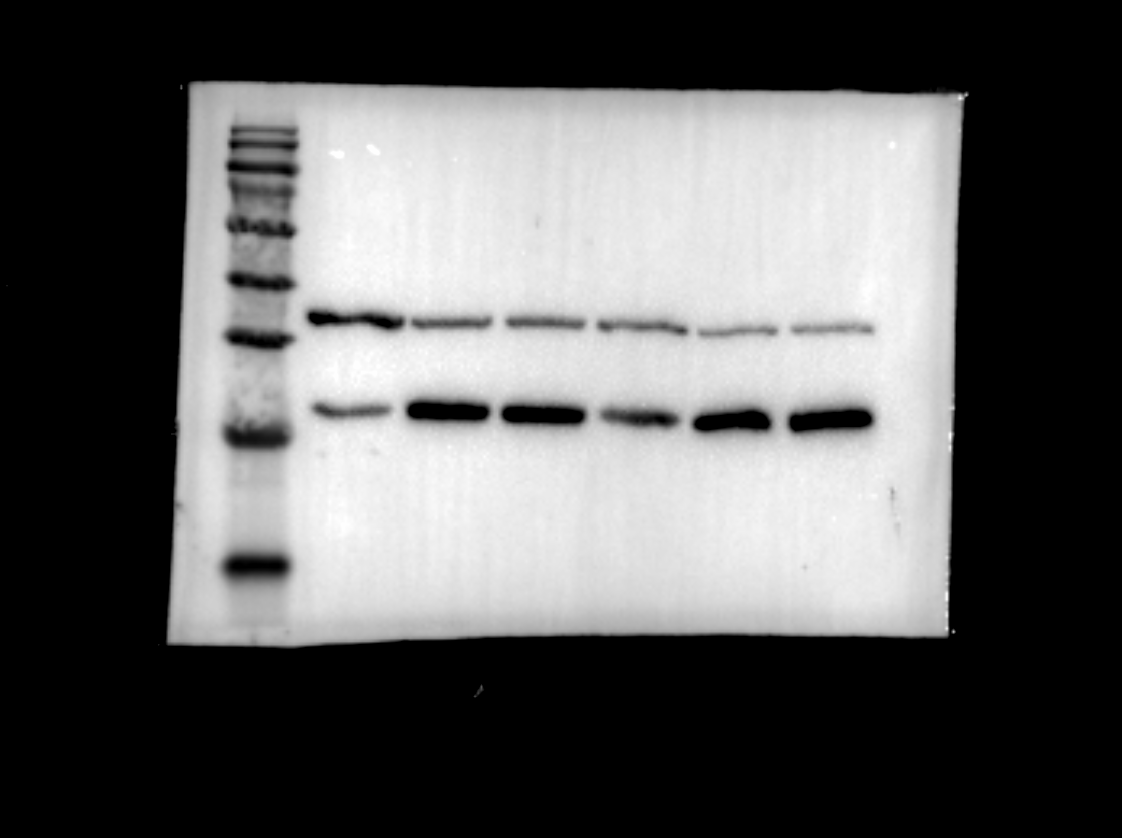

Supplement: Supplementary file 1 — Additional file 1: Figure S1. Verification of deletion mutants by PCR and quantitative real-time PCR. (A) Rice blast fungus knockout model. (B) PCR detection was performed on the null mutants, and the recombinant fragment (LF) was cloned from the positive strain but not from the WT strain. The 500-bp characteristic fragment (SF) of the target gene was cloned in the WT strain but not in the mutants. TUBULIN was used as a positive control. (C) The copy number of the resistance gene HPH in the deletion mutants was verified by quantitative real-time PCR. Figure S2. The targeted gene destruction of CSN subunits decreased the growth, sporulation, and pathogenicity of M. oryzae. (A) Colony morphology of 70-15, ΔMocsn1, Mocsn1-C, ΔMocsn2, Mocsn2-C, ΔMocsn3, Mocsn3-C, ΔMocsn4, Mocsn4-C, ΔMocsn7a, and Mocsn7a-C. The strains were grown on CM and MM plates for 9 days. (B) Statistical analysis of the colony growth diameter. The data were analyzed using GraphPad Prism 8.0 software. The error bars represent the standard deviations. ***P < 0.001. (C) Conidiophores of 70-15, ΔMocsn1, Mocsn1-C, ΔMocsn2, Mocsn2-C, ΔMocsn3, Mocsn3-C, ΔMocsn4, Mocsn4-C, ΔMocsn7a, and Mocsn7a-C. The strains were cultivated in an incubator at 25 ℃ for 9 days and observed under an optical microscope. (D) Disease spots of detached barley leaves inoculated with mycelial plugs from 70-15, ΔMocsn1, Mocsn1-C, ΔMocsn2, Mocsn2-C, ΔMocsn3, Mocsn3-C, ΔMocsn4, Mocsn4-C, ΔMocsn7a, and Mocsn7a-C. Leaves were cultured at 25 ℃ for 4 days after inoculation. Figure S3. CSN subunits are involved in the regulation of autophagy in M. oryzae. (A) Yeast two-hybrid assays were used to detect the interactions between CSN subunits and Atg-related proteins. Pairs of pGBKT7-53 and PGADT7-T were used as positive controls. Yeast transformants carrying the indicated constructs were plated onto selective plates supplemented without Leu/Trp or Leu/Trp/His/Ade for growth assays. (B) Autophagic flux analysis of GFP-MoAtg8 in 70-15 and ΔMo [file 12964_2024_1598_MOESM1_ESM.zip › Supplementary files/Figure5D-GFP.tif]

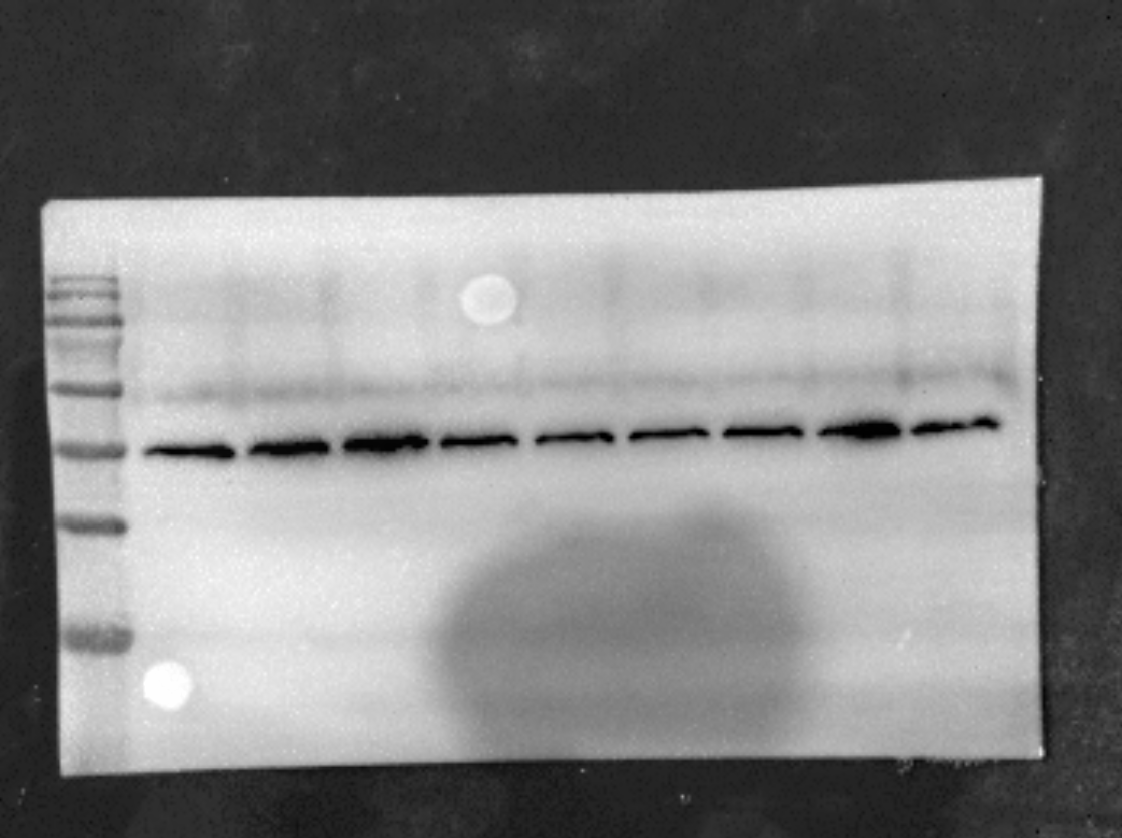

Supplement: Supplementary file 1 — Additional file 1: Figure S1. Verification of deletion mutants by PCR and quantitative real-time PCR. (A) Rice blast fungus knockout model. (B) PCR detection was performed on the null mutants, and the recombinant fragment (LF) was cloned from the positive strain but not from the WT strain. The 500-bp characteristic fragment (SF) of the target gene was cloned in the WT strain but not in the mutants. TUBULIN was used as a positive control. (C) The copy number of the resistance gene HPH in the deletion mutants was verified by quantitative real-time PCR. Figure S2. The targeted gene destruction of CSN subunits decreased the growth, sporulation, and pathogenicity of M. oryzae. (A) Colony morphology of 70-15, ΔMocsn1, Mocsn1-C, ΔMocsn2, Mocsn2-C, ΔMocsn3, Mocsn3-C, ΔMocsn4, Mocsn4-C, ΔMocsn7a, and Mocsn7a-C. The strains were grown on CM and MM plates for 9 days. (B) Statistical analysis of the colony growth diameter. The data were analyzed using GraphPad Prism 8.0 software. The error bars represent the standard deviations. ***P < 0.001. (C) Conidiophores of 70-15, ΔMocsn1, Mocsn1-C, ΔMocsn2, Mocsn2-C, ΔMocsn3, Mocsn3-C, ΔMocsn4, Mocsn4-C, ΔMocsn7a, and Mocsn7a-C. The strains were cultivated in an incubator at 25 ℃ for 9 days and observed under an optical microscope. (D) Disease spots of detached barley leaves inoculated with mycelial plugs from 70-15, ΔMocsn1, Mocsn1-C, ΔMocsn2, Mocsn2-C, ΔMocsn3, Mocsn3-C, ΔMocsn4, Mocsn4-C, ΔMocsn7a, and Mocsn7a-C. Leaves were cultured at 25 ℃ for 4 days after inoculation. Figure S3. CSN subunits are involved in the regulation of autophagy in M. oryzae. (A) Yeast two-hybrid assays were used to detect the interactions between CSN subunits and Atg-related proteins. Pairs of pGBKT7-53 and PGADT7-T were used as positive controls. Yeast transformants carrying the indicated constructs were plated onto selective plates supplemented without Leu/Trp or Leu/Trp/His/Ade for growth assays. (B) Autophagic flux analysis of GFP-MoAtg8 in 70-15 and ΔMo [file 12964_2024_1598_MOESM1_ESM.zip › Supplementary files/Figure5E-Actin.tif]

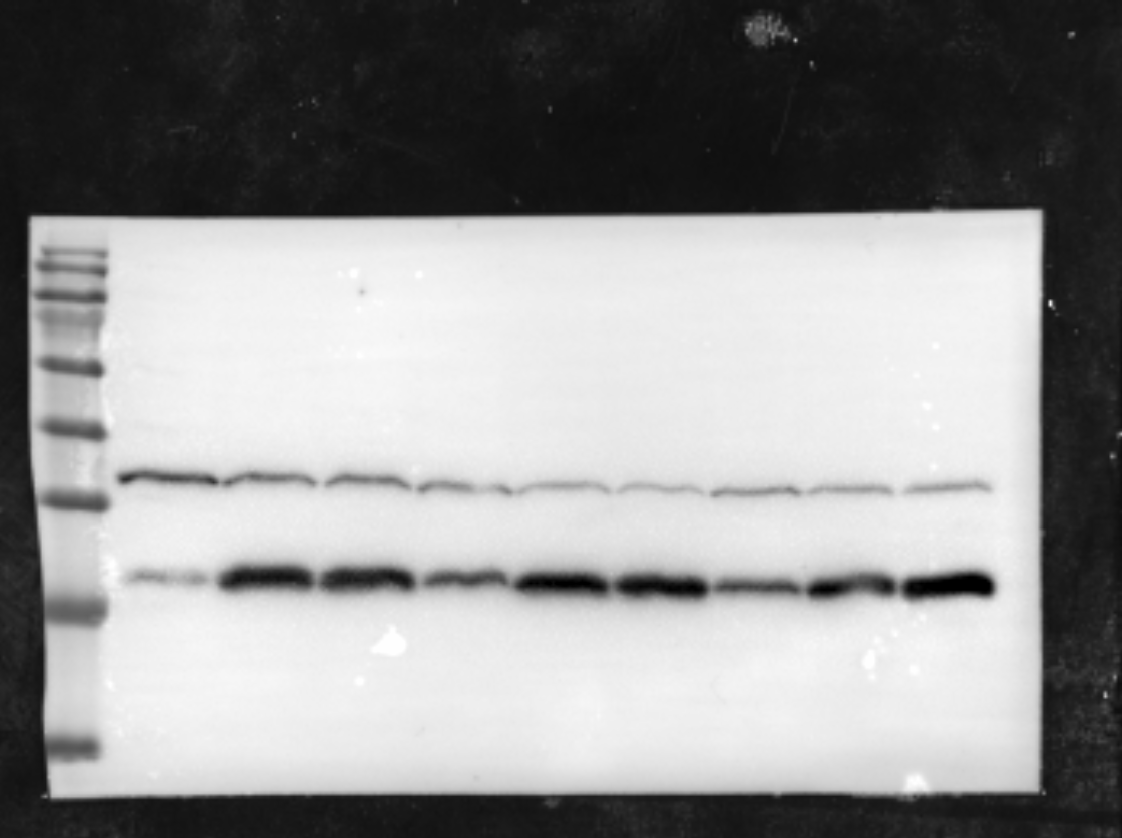

Supplement: Supplementary file 1 — Additional file 1: Figure S1. Verification of deletion mutants by PCR and quantitative real-time PCR. (A) Rice blast fungus knockout model. (B) PCR detection was performed on the null mutants, and the recombinant fragment (LF) was cloned from the positive strain but not from the WT strain. The 500-bp characteristic fragment (SF) of the target gene was cloned in the WT strain but not in the mutants. TUBULIN was used as a positive control. (C) The copy number of the resistance gene HPH in the deletion mutants was verified by quantitative real-time PCR. Figure S2. The targeted gene destruction of CSN subunits decreased the growth, sporulation, and pathogenicity of M. oryzae. (A) Colony morphology of 70-15, ΔMocsn1, Mocsn1-C, ΔMocsn2, Mocsn2-C, ΔMocsn3, Mocsn3-C, ΔMocsn4, Mocsn4-C, ΔMocsn7a, and Mocsn7a-C. The strains were grown on CM and MM plates for 9 days. (B) Statistical analysis of the colony growth diameter. The data were analyzed using GraphPad Prism 8.0 software. The error bars represent the standard deviations. ***P < 0.001. (C) Conidiophores of 70-15, ΔMocsn1, Mocsn1-C, ΔMocsn2, Mocsn2-C, ΔMocsn3, Mocsn3-C, ΔMocsn4, Mocsn4-C, ΔMocsn7a, and Mocsn7a-C. The strains were cultivated in an incubator at 25 ℃ for 9 days and observed under an optical microscope. (D) Disease spots of detached barley leaves inoculated with mycelial plugs from 70-15, ΔMocsn1, Mocsn1-C, ΔMocsn2, Mocsn2-C, ΔMocsn3, Mocsn3-C, ΔMocsn4, Mocsn4-C, ΔMocsn7a, and Mocsn7a-C. Leaves were cultured at 25 ℃ for 4 days after inoculation. Figure S3. CSN subunits are involved in the regulation of autophagy in M. oryzae. (A) Yeast two-hybrid assays were used to detect the interactions between CSN subunits and Atg-related proteins. Pairs of pGBKT7-53 and PGADT7-T were used as positive controls. Yeast transformants carrying the indicated constructs were plated onto selective plates supplemented without Leu/Trp or Leu/Trp/His/Ade for growth assays. (B) Autophagic flux analysis of GFP-MoAtg8 in 70-15 and ΔMo [file 12964_2024_1598_MOESM1_ESM.zip › Supplementary files/Figure5E-GFP.tif]

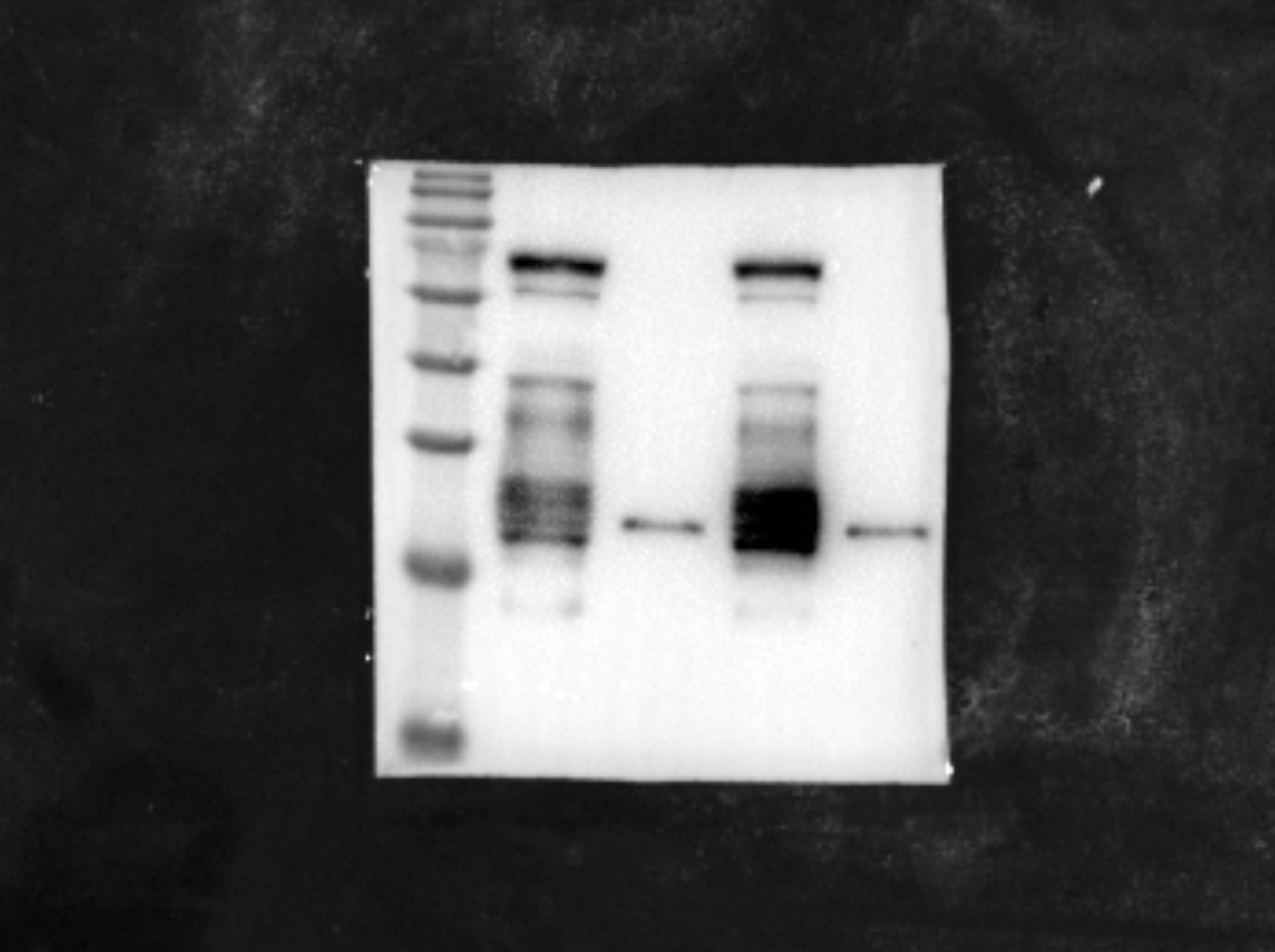

Supplement: Supplementary file 1 — Additional file 1: Figure S1. Verification of deletion mutants by PCR and quantitative real-time PCR. (A) Rice blast fungus knockout model. (B) PCR detection was performed on the null mutants, and the recombinant fragment (LF) was cloned from the positive strain but not from the WT strain. The 500-bp characteristic fragment (SF) of the target gene was cloned in the WT strain but not in the mutants. TUBULIN was used as a positive control. (C) The copy number of the resistance gene HPH in the deletion mutants was verified by quantitative real-time PCR. Figure S2. The targeted gene destruction of CSN subunits decreased the growth, sporulation, and pathogenicity of M. oryzae. (A) Colony morphology of 70-15, ΔMocsn1, Mocsn1-C, ΔMocsn2, Mocsn2-C, ΔMocsn3, Mocsn3-C, ΔMocsn4, Mocsn4-C, ΔMocsn7a, and Mocsn7a-C. The strains were grown on CM and MM plates for 9 days. (B) Statistical analysis of the colony growth diameter. The data were analyzed using GraphPad Prism 8.0 software. The error bars represent the standard deviations. ***P < 0.001. (C) Conidiophores of 70-15, ΔMocsn1, Mocsn1-C, ΔMocsn2, Mocsn2-C, ΔMocsn3, Mocsn3-C, ΔMocsn4, Mocsn4-C, ΔMocsn7a, and Mocsn7a-C. The strains were cultivated in an incubator at 25 ℃ for 9 days and observed under an optical microscope. (D) Disease spots of detached barley leaves inoculated with mycelial plugs from 70-15, ΔMocsn1, Mocsn1-C, ΔMocsn2, Mocsn2-C, ΔMocsn3, Mocsn3-C, ΔMocsn4, Mocsn4-C, ΔMocsn7a, and Mocsn7a-C. Leaves were cultured at 25 ℃ for 4 days after inoculation. Figure S3. CSN subunits are involved in the regulation of autophagy in M. oryzae. (A) Yeast two-hybrid assays were used to detect the interactions between CSN subunits and Atg-related proteins. Pairs of pGBKT7-53 and PGADT7-T were used as positive controls. Yeast transformants carrying the indicated constructs were plated onto selective plates supplemented without Leu/Trp or Leu/Trp/His/Ade for growth assays. (B) Autophagic flux analysis of GFP-MoAtg8 in 70-15 and ΔMo [file 12964_2024_1598_MOESM1_ESM.zip › Supplementary files/Figure6C-GST.tif]

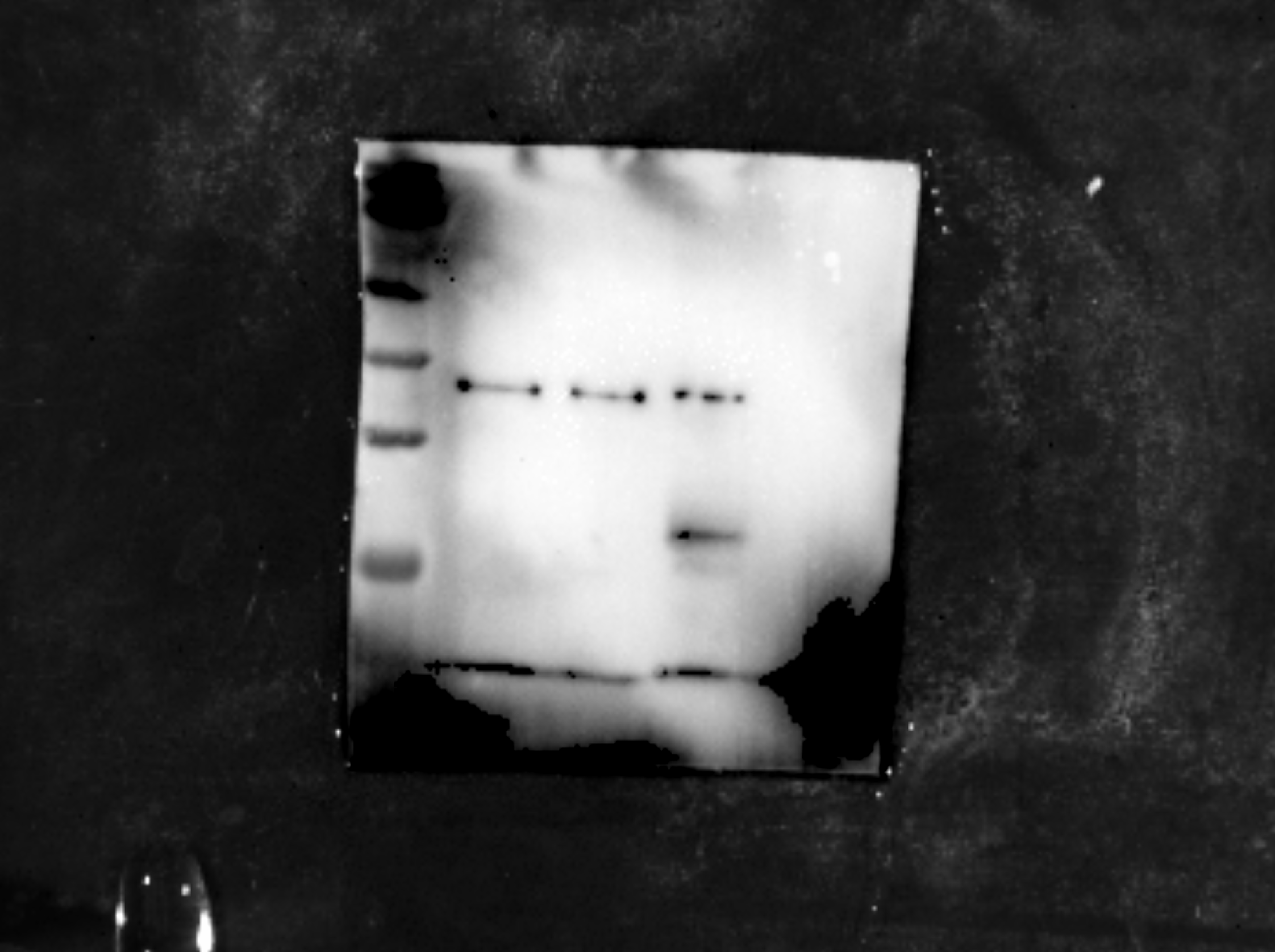

Supplement: Supplementary file 1 — Additional file 1: Figure S1. Verification of deletion mutants by PCR and quantitative real-time PCR. (A) Rice blast fungus knockout model. (B) PCR detection was performed on the null mutants, and the recombinant fragment (LF) was cloned from the positive strain but not from the WT strain. The 500-bp characteristic fragment (SF) of the target gene was cloned in the WT strain but not in the mutants. TUBULIN was used as a positive control. (C) The copy number of the resistance gene HPH in the deletion mutants was verified by quantitative real-time PCR. Figure S2. The targeted gene destruction of CSN subunits decreased the growth, sporulation, and pathogenicity of M. oryzae. (A) Colony morphology of 70-15, ΔMocsn1, Mocsn1-C, ΔMocsn2, Mocsn2-C, ΔMocsn3, Mocsn3-C, ΔMocsn4, Mocsn4-C, ΔMocsn7a, and Mocsn7a-C. The strains were grown on CM and MM plates for 9 days. (B) Statistical analysis of the colony growth diameter. The data were analyzed using GraphPad Prism 8.0 software. The error bars represent the standard deviations. ***P < 0.001. (C) Conidiophores of 70-15, ΔMocsn1, Mocsn1-C, ΔMocsn2, Mocsn2-C, ΔMocsn3, Mocsn3-C, ΔMocsn4, Mocsn4-C, ΔMocsn7a, and Mocsn7a-C. The strains were cultivated in an incubator at 25 ℃ for 9 days and observed under an optical microscope. (D) Disease spots of detached barley leaves inoculated with mycelial plugs from 70-15, ΔMocsn1, Mocsn1-C, ΔMocsn2, Mocsn2-C, ΔMocsn3, Mocsn3-C, ΔMocsn4, Mocsn4-C, ΔMocsn7a, and Mocsn7a-C. Leaves were cultured at 25 ℃ for 4 days after inoculation. Figure S3. CSN subunits are involved in the regulation of autophagy in M. oryzae. (A) Yeast two-hybrid assays were used to detect the interactions between CSN subunits and Atg-related proteins. Pairs of pGBKT7-53 and PGADT7-T were used as positive controls. Yeast transformants carrying the indicated constructs were plated onto selective plates supplemented without Leu/Trp or Leu/Trp/His/Ade for growth assays. (B) Autophagic flux analysis of GFP-MoAtg8 in 70-15 and ΔMo [file 12964_2024_1598_MOESM1_ESM.zip › Supplementary files/Figure6C-His.tif]

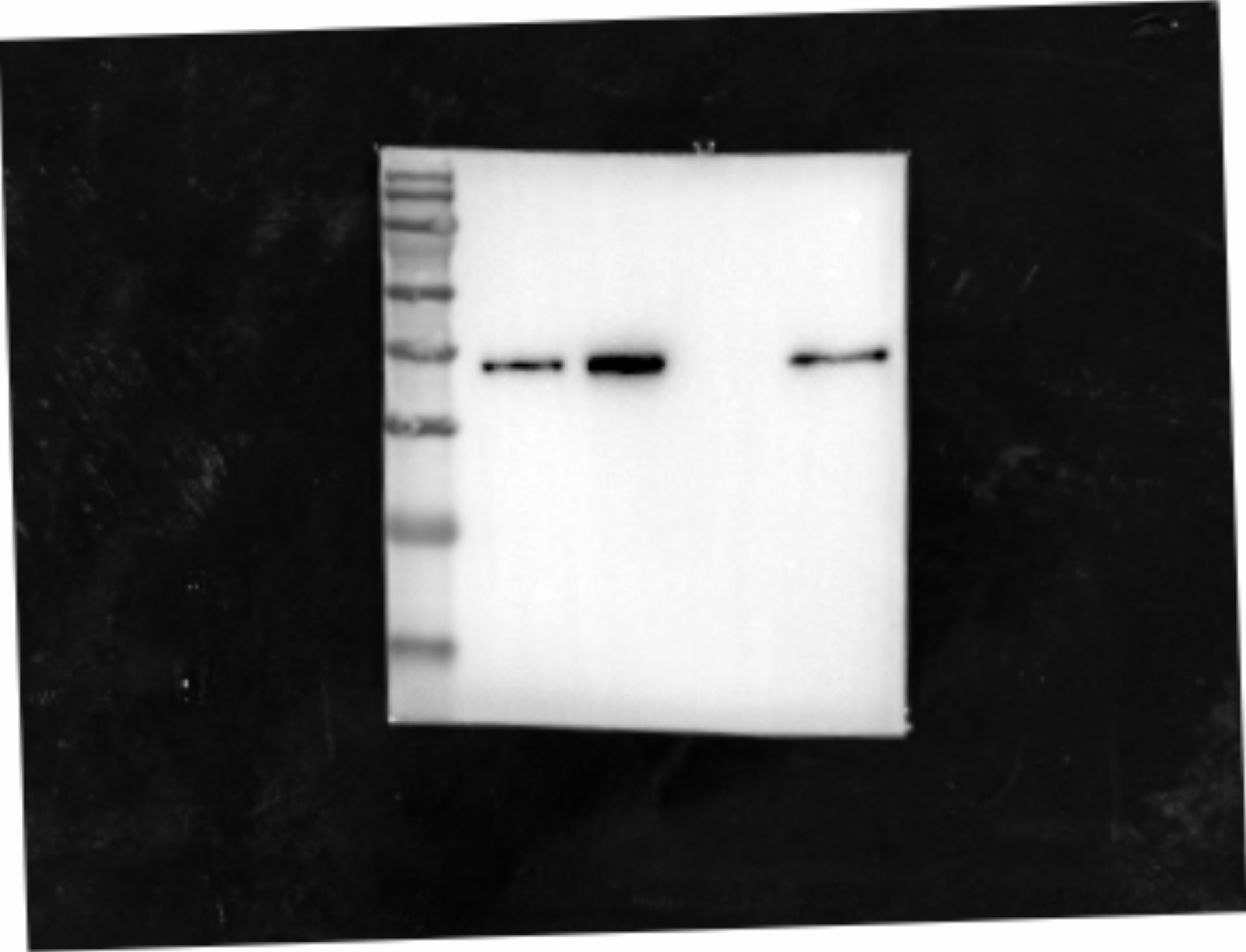

Supplement: Supplementary file 1 — Additional file 1: Figure S1. Verification of deletion mutants by PCR and quantitative real-time PCR. (A) Rice blast fungus knockout model. (B) PCR detection was performed on the null mutants, and the recombinant fragment (LF) was cloned from the positive strain but not from the WT strain. The 500-bp characteristic fragment (SF) of the target gene was cloned in the WT strain but not in the mutants. TUBULIN was used as a positive control. (C) The copy number of the resistance gene HPH in the deletion mutants was verified by quantitative real-time PCR. Figure S2. The targeted gene destruction of CSN subunits decreased the growth, sporulation, and pathogenicity of M. oryzae. (A) Colony morphology of 70-15, ΔMocsn1, Mocsn1-C, ΔMocsn2, Mocsn2-C, ΔMocsn3, Mocsn3-C, ΔMocsn4, Mocsn4-C, ΔMocsn7a, and Mocsn7a-C. The strains were grown on CM and MM plates for 9 days. (B) Statistical analysis of the colony growth diameter. The data were analyzed using GraphPad Prism 8.0 software. The error bars represent the standard deviations. ***P < 0.001. (C) Conidiophores of 70-15, ΔMocsn1, Mocsn1-C, ΔMocsn2, Mocsn2-C, ΔMocsn3, Mocsn3-C, ΔMocsn4, Mocsn4-C, ΔMocsn7a, and Mocsn7a-C. The strains were cultivated in an incubator at 25 ℃ for 9 days and observed under an optical microscope. (D) Disease spots of detached barley leaves inoculated with mycelial plugs from 70-15, ΔMocsn1, Mocsn1-C, ΔMocsn2, Mocsn2-C, ΔMocsn3, Mocsn3-C, ΔMocsn4, Mocsn4-C, ΔMocsn7a, and Mocsn7a-C. Leaves were cultured at 25 ℃ for 4 days after inoculation. Figure S3. CSN subunits are involved in the regulation of autophagy in M. oryzae. (A) Yeast two-hybrid assays were used to detect the interactions between CSN subunits and Atg-related proteins. Pairs of pGBKT7-53 and PGADT7-T were used as positive controls. Yeast transformants carrying the indicated constructs were plated onto selective plates supplemented without Leu/Trp or Leu/Trp/His/Ade for growth assays. (B) Autophagic flux analysis of GFP-MoAtg8 in 70-15 and ΔMo [file 12964_2024_1598_MOESM1_ESM.zip › Supplementary files/Figure6D-Flag.tif]

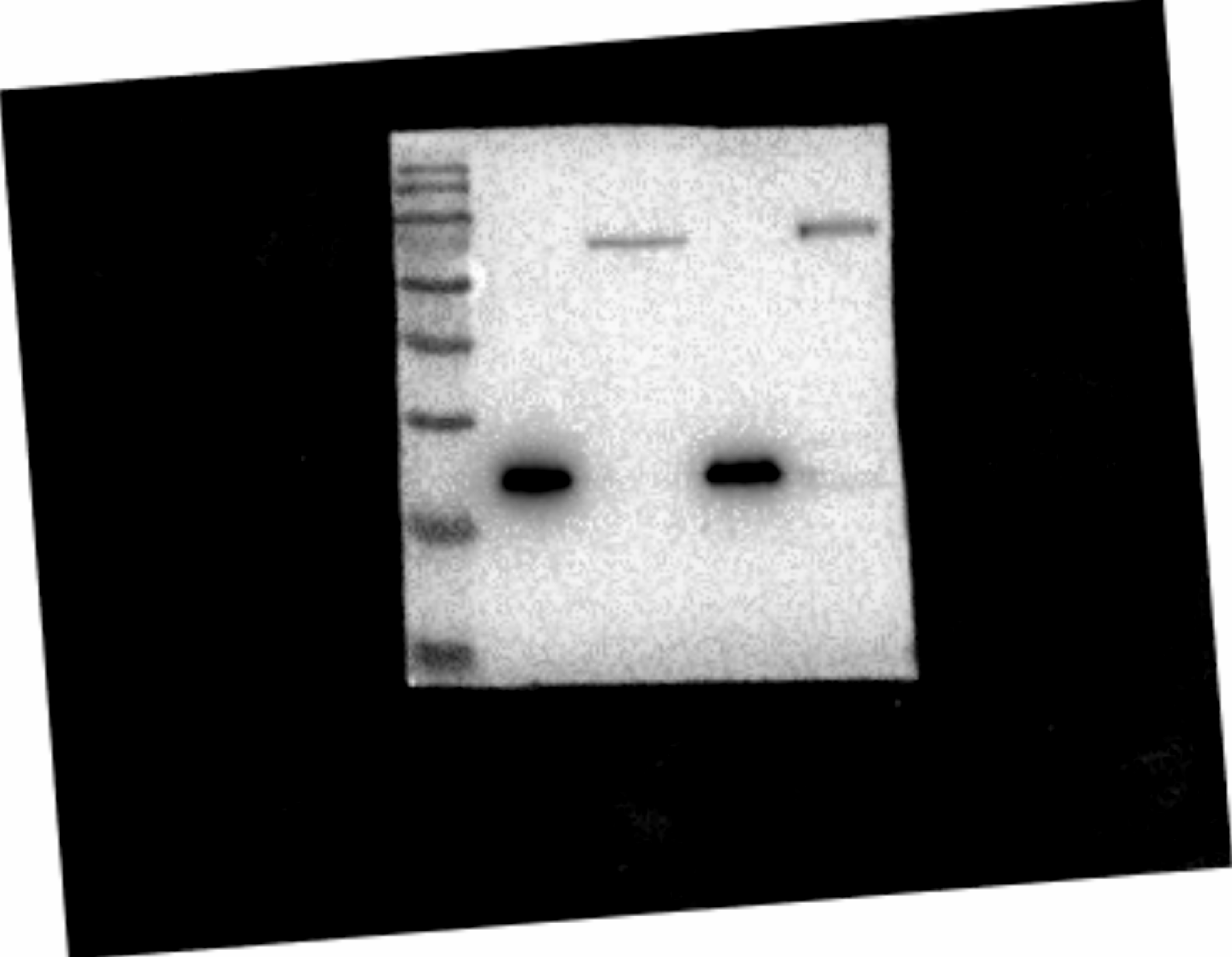

Supplement: Supplementary file 1 — Additional file 1: Figure S1. Verification of deletion mutants by PCR and quantitative real-time PCR. (A) Rice blast fungus knockout model. (B) PCR detection was performed on the null mutants, and the recombinant fragment (LF) was cloned from the positive strain but not from the WT strain. The 500-bp characteristic fragment (SF) of the target gene was cloned in the WT strain but not in the mutants. TUBULIN was used as a positive control. (C) The copy number of the resistance gene HPH in the deletion mutants was verified by quantitative real-time PCR. Figure S2. The targeted gene destruction of CSN subunits decreased the growth, sporulation, and pathogenicity of M. oryzae. (A) Colony morphology of 70-15, ΔMocsn1, Mocsn1-C, ΔMocsn2, Mocsn2-C, ΔMocsn3, Mocsn3-C, ΔMocsn4, Mocsn4-C, ΔMocsn7a, and Mocsn7a-C. The strains were grown on CM and MM plates for 9 days. (B) Statistical analysis of the colony growth diameter. The data were analyzed using GraphPad Prism 8.0 software. The error bars represent the standard deviations. ***P < 0.001. (C) Conidiophores of 70-15, ΔMocsn1, Mocsn1-C, ΔMocsn2, Mocsn2-C, ΔMocsn3, Mocsn3-C, ΔMocsn4, Mocsn4-C, ΔMocsn7a, and Mocsn7a-C. The strains were cultivated in an incubator at 25 ℃ for 9 days and observed under an optical microscope. (D) Disease spots of detached barley leaves inoculated with mycelial plugs from 70-15, ΔMocsn1, Mocsn1-C, ΔMocsn2, Mocsn2-C, ΔMocsn3, Mocsn3-C, ΔMocsn4, Mocsn4-C, ΔMocsn7a, and Mocsn7a-C. Leaves were cultured at 25 ℃ for 4 days after inoculation. Figure S3. CSN subunits are involved in the regulation of autophagy in M. oryzae. (A) Yeast two-hybrid assays were used to detect the interactions between CSN subunits and Atg-related proteins. Pairs of pGBKT7-53 and PGADT7-T were used as positive controls. Yeast transformants carrying the indicated constructs were plated onto selective plates supplemented without Leu/Trp or Leu/Trp/His/Ade for growth assays. (B) Autophagic flux analysis of GFP-MoAtg8 in 70-15 and ΔMo [file 12964_2024_1598_MOESM1_ESM.zip › Supplementary files/Figure6D-GFP.tif]

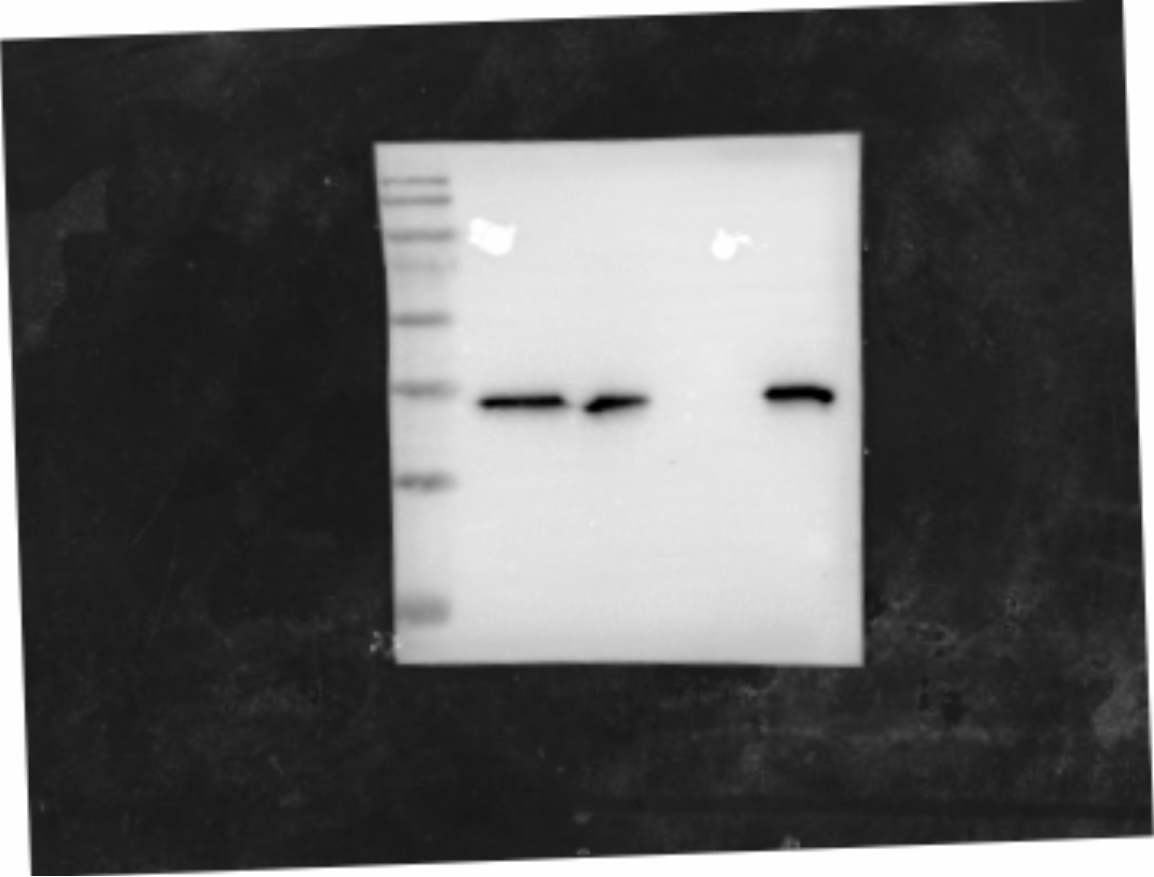

Supplement: Supplementary file 1 — Additional file 1: Figure S1. Verification of deletion mutants by PCR and quantitative real-time PCR. (A) Rice blast fungus knockout model. (B) PCR detection was performed on the null mutants, and the recombinant fragment (LF) was cloned from the positive strain but not from the WT strain. The 500-bp characteristic fragment (SF) of the target gene was cloned in the WT strain but not in the mutants. TUBULIN was used as a positive control. (C) The copy number of the resistance gene HPH in the deletion mutants was verified by quantitative real-time PCR. Figure S2. The targeted gene destruction of CSN subunits decreased the growth, sporulation, and pathogenicity of M. oryzae. (A) Colony morphology of 70-15, ΔMocsn1, Mocsn1-C, ΔMocsn2, Mocsn2-C, ΔMocsn3, Mocsn3-C, ΔMocsn4, Mocsn4-C, ΔMocsn7a, and Mocsn7a-C. The strains were grown on CM and MM plates for 9 days. (B) Statistical analysis of the colony growth diameter. The data were analyzed using GraphPad Prism 8.0 software. The error bars represent the standard deviations. ***P < 0.001. (C) Conidiophores of 70-15, ΔMocsn1, Mocsn1-C, ΔMocsn2, Mocsn2-C, ΔMocsn3, Mocsn3-C, ΔMocsn4, Mocsn4-C, ΔMocsn7a, and Mocsn7a-C. The strains were cultivated in an incubator at 25 ℃ for 9 days and observed under an optical microscope. (D) Disease spots of detached barley leaves inoculated with mycelial plugs from 70-15, ΔMocsn1, Mocsn1-C, ΔMocsn2, Mocsn2-C, ΔMocsn3, Mocsn3-C, ΔMocsn4, Mocsn4-C, ΔMocsn7a, and Mocsn7a-C. Leaves were cultured at 25 ℃ for 4 days after inoculation. Figure S3. CSN subunits are involved in the regulation of autophagy in M. oryzae. (A) Yeast two-hybrid assays were used to detect the interactions between CSN subunits and Atg-related proteins. Pairs of pGBKT7-53 and PGADT7-T were used as positive controls. Yeast transformants carrying the indicated constructs were plated onto selective plates supplemented without Leu/Trp or Leu/Trp/His/Ade for growth assays. (B) Autophagic flux analysis of GFP-MoAtg8 in 70-15 and ΔMo [file 12964_2024_1598_MOESM1_ESM.zip › Supplementary files/Figure6E-Flag.tif]

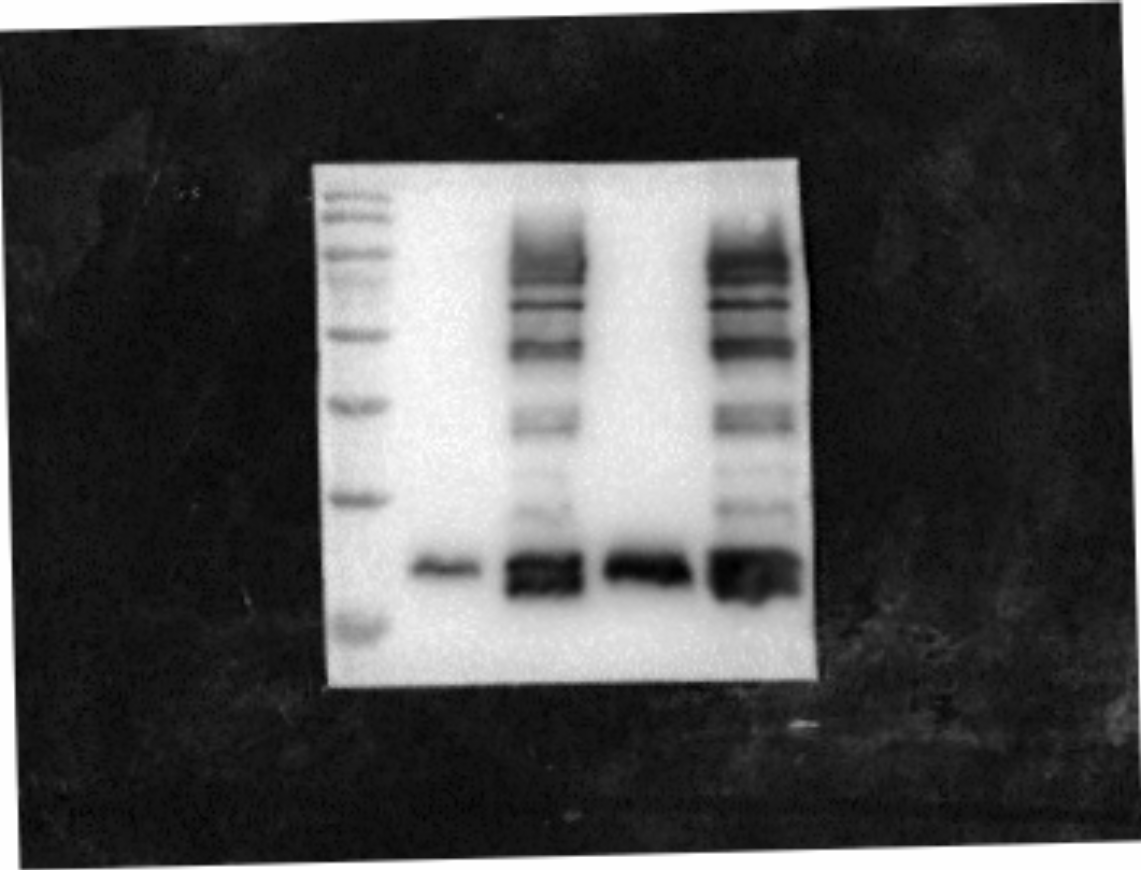

Supplement: Supplementary file 1 — Additional file 1: Figure S1. Verification of deletion mutants by PCR and quantitative real-time PCR. (A) Rice blast fungus knockout model. (B) PCR detection was performed on the null mutants, and the recombinant fragment (LF) was cloned from the positive strain but not from the WT strain. The 500-bp characteristic fragment (SF) of the target gene was cloned in the WT strain but not in the mutants. TUBULIN was used as a positive control. (C) The copy number of the resistance gene HPH in the deletion mutants was verified by quantitative real-time PCR. Figure S2. The targeted gene destruction of CSN subunits decreased the growth, sporulation, and pathogenicity of M. oryzae. (A) Colony morphology of 70-15, ΔMocsn1, Mocsn1-C, ΔMocsn2, Mocsn2-C, ΔMocsn3, Mocsn3-C, ΔMocsn4, Mocsn4-C, ΔMocsn7a, and Mocsn7a-C. The strains were grown on CM and MM plates for 9 days. (B) Statistical analysis of the colony growth diameter. The data were analyzed using GraphPad Prism 8.0 software. The error bars represent the standard deviations. ***P < 0.001. (C) Conidiophores of 70-15, ΔMocsn1, Mocsn1-C, ΔMocsn2, Mocsn2-C, ΔMocsn3, Mocsn3-C, ΔMocsn4, Mocsn4-C, ΔMocsn7a, and Mocsn7a-C. The strains were cultivated in an incubator at 25 ℃ for 9 days and observed under an optical microscope. (D) Disease spots of detached barley leaves inoculated with mycelial plugs from 70-15, ΔMocsn1, Mocsn1-C, ΔMocsn2, Mocsn2-C, ΔMocsn3, Mocsn3-C, ΔMocsn4, Mocsn4-C, ΔMocsn7a, and Mocsn7a-C. Leaves were cultured at 25 ℃ for 4 days after inoculation. Figure S3. CSN subunits are involved in the regulation of autophagy in M. oryzae. (A) Yeast two-hybrid assays were used to detect the interactions between CSN subunits and Atg-related proteins. Pairs of pGBKT7-53 and PGADT7-T were used as positive controls. Yeast transformants carrying the indicated constructs were plated onto selective plates supplemented without Leu/Trp or Leu/Trp/His/Ade for growth assays. (B) Autophagic flux analysis of GFP-MoAtg8 in 70-15 and ΔMo [file 12964_2024_1598_MOESM1_ESM.zip › Supplementary files/Figure6E-GFP.tif]

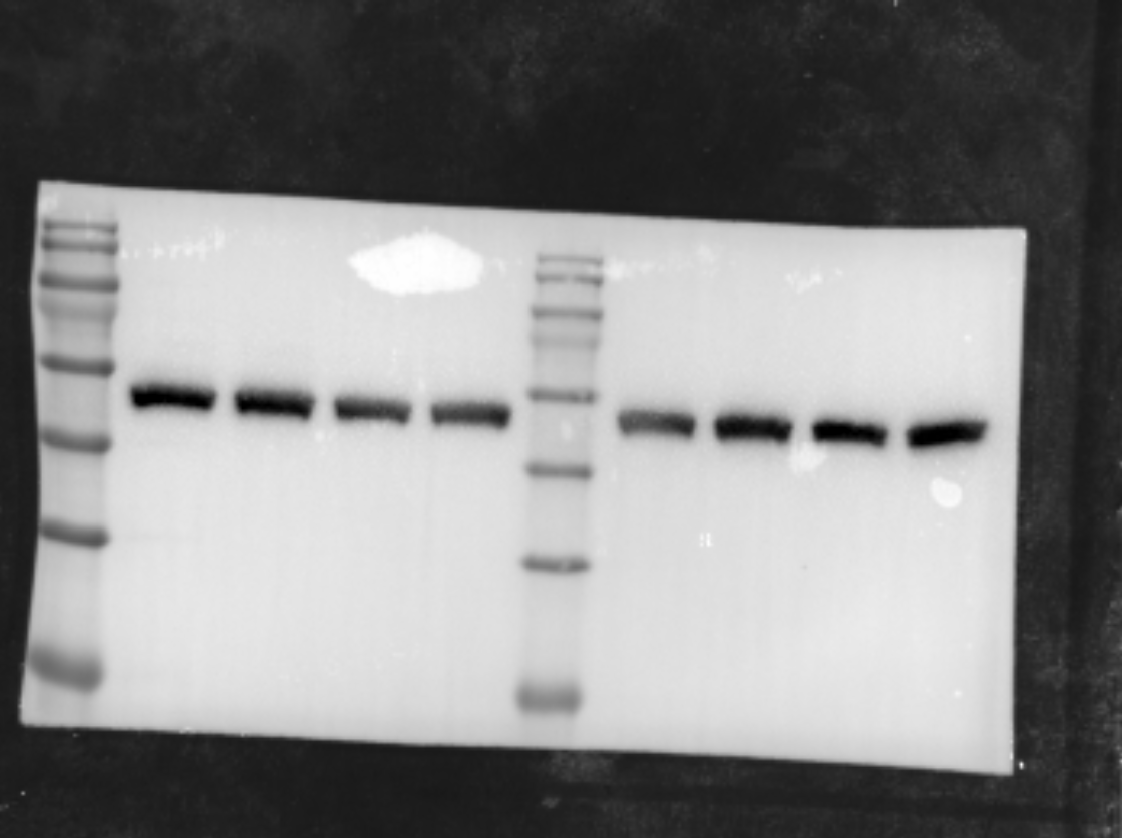

Supplement: Supplementary file 1 — Additional file 1: Figure S1. Verification of deletion mutants by PCR and quantitative real-time PCR. (A) Rice blast fungus knockout model. (B) PCR detection was performed on the null mutants, and the recombinant fragment (LF) was cloned from the positive strain but not from the WT strain. The 500-bp characteristic fragment (SF) of the target gene was cloned in the WT strain but not in the mutants. TUBULIN was used as a positive control. (C) The copy number of the resistance gene HPH in the deletion mutants was verified by quantitative real-time PCR. Figure S2. The targeted gene destruction of CSN subunits decreased the growth, sporulation, and pathogenicity of M. oryzae. (A) Colony morphology of 70-15, ΔMocsn1, Mocsn1-C, ΔMocsn2, Mocsn2-C, ΔMocsn3, Mocsn3-C, ΔMocsn4, Mocsn4-C, ΔMocsn7a, and Mocsn7a-C. The strains were grown on CM and MM plates for 9 days. (B) Statistical analysis of the colony growth diameter. The data were analyzed using GraphPad Prism 8.0 software. The error bars represent the standard deviations. ***P < 0.001. (C) Conidiophores of 70-15, ΔMocsn1, Mocsn1-C, ΔMocsn2, Mocsn2-C, ΔMocsn3, Mocsn3-C, ΔMocsn4, Mocsn4-C, ΔMocsn7a, and Mocsn7a-C. The strains were cultivated in an incubator at 25 ℃ for 9 days and observed under an optical microscope. (D) Disease spots of detached barley leaves inoculated with mycelial plugs from 70-15, ΔMocsn1, Mocsn1-C, ΔMocsn2, Mocsn2-C, ΔMocsn3, Mocsn3-C, ΔMocsn4, Mocsn4-C, ΔMocsn7a, and Mocsn7a-C. Leaves were cultured at 25 ℃ for 4 days after inoculation. Figure S3. CSN subunits are involved in the regulation of autophagy in M. oryzae. (A) Yeast two-hybrid assays were used to detect the interactions between CSN subunits and Atg-related proteins. Pairs of pGBKT7-53 and PGADT7-T were used as positive controls. Yeast transformants carrying the indicated constructs were plated onto selective plates supplemented without Leu/Trp or Leu/Trp/His/Ade for growth assays. (B) Autophagic flux analysis of GFP-MoAtg8 in 70-15 and ΔMo [file 12964_2024_1598_MOESM1_ESM.zip › Supplementary files/Figure6F-IP-A14Flag(left).tif]

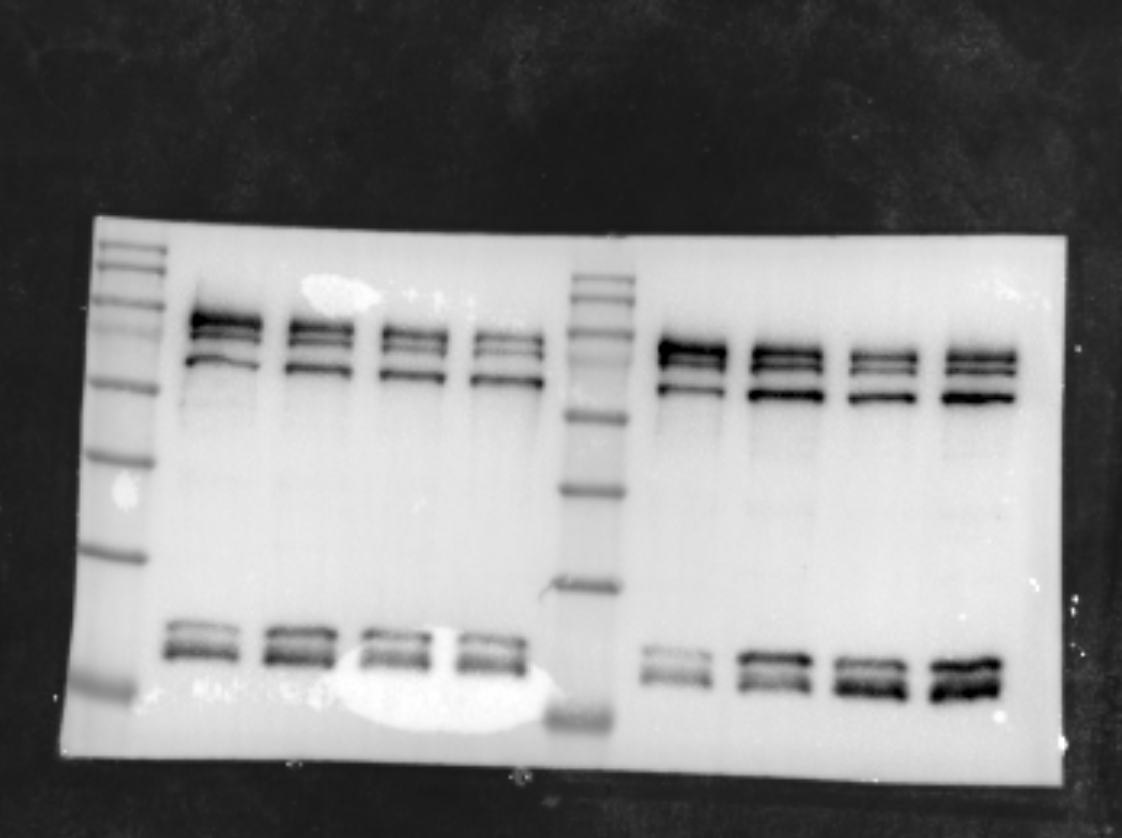

Supplement: Supplementary file 1 — Additional file 1: Figure S1. Verification of deletion mutants by PCR and quantitative real-time PCR. (A) Rice blast fungus knockout model. (B) PCR detection was performed on the null mutants, and the recombinant fragment (LF) was cloned from the positive strain but not from the WT strain. The 500-bp characteristic fragment (SF) of the target gene was cloned in the WT strain but not in the mutants. TUBULIN was used as a positive control. (C) The copy number of the resistance gene HPH in the deletion mutants was verified by quantitative real-time PCR. Figure S2. The targeted gene destruction of CSN subunits decreased the growth, sporulation, and pathogenicity of M. oryzae. (A) Colony morphology of 70-15, ΔMocsn1, Mocsn1-C, ΔMocsn2, Mocsn2-C, ΔMocsn3, Mocsn3-C, ΔMocsn4, Mocsn4-C, ΔMocsn7a, and Mocsn7a-C. The strains were grown on CM and MM plates for 9 days. (B) Statistical analysis of the colony growth diameter. The data were analyzed using GraphPad Prism 8.0 software. The error bars represent the standard deviations. ***P < 0.001. (C) Conidiophores of 70-15, ΔMocsn1, Mocsn1-C, ΔMocsn2, Mocsn2-C, ΔMocsn3, Mocsn3-C, ΔMocsn4, Mocsn4-C, ΔMocsn7a, and Mocsn7a-C. The strains were cultivated in an incubator at 25 ℃ for 9 days and observed under an optical microscope. (D) Disease spots of detached barley leaves inoculated with mycelial plugs from 70-15, ΔMocsn1, Mocsn1-C, ΔMocsn2, Mocsn2-C, ΔMocsn3, Mocsn3-C, ΔMocsn4, Mocsn4-C, ΔMocsn7a, and Mocsn7a-C. Leaves were cultured at 25 ℃ for 4 days after inoculation. Figure S3. CSN subunits are involved in the regulation of autophagy in M. oryzae. (A) Yeast two-hybrid assays were used to detect the interactions between CSN subunits and Atg-related proteins. Pairs of pGBKT7-53 and PGADT7-T were used as positive controls. Yeast transformants carrying the indicated constructs were plated onto selective plates supplemented without Leu/Trp or Leu/Trp/His/Ade for growth assays. (B) Autophagic flux analysis of GFP-MoAtg8 in 70-15 and ΔMo [file 12964_2024_1598_MOESM1_ESM.zip › Supplementary files/Figure6F-IP-A6GFP(left).tif]

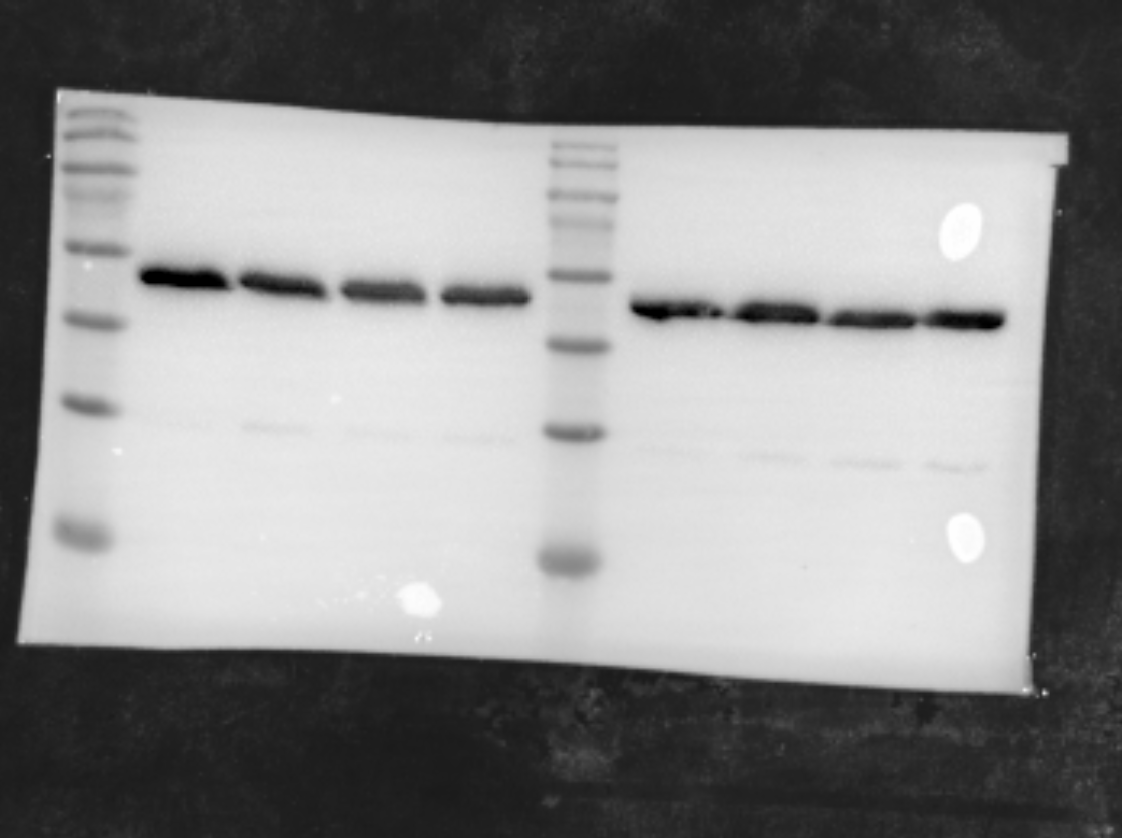

Supplement: Supplementary file 1 — Additional file 1: Figure S1. Verification of deletion mutants by PCR and quantitative real-time PCR. (A) Rice blast fungus knockout model. (B) PCR detection was performed on the null mutants, and the recombinant fragment (LF) was cloned from the positive strain but not from the WT strain. The 500-bp characteristic fragment (SF) of the target gene was cloned in the WT strain but not in the mutants. TUBULIN was used as a positive control. (C) The copy number of the resistance gene HPH in the deletion mutants was verified by quantitative real-time PCR. Figure S2. The targeted gene destruction of CSN subunits decreased the growth, sporulation, and pathogenicity of M. oryzae. (A) Colony morphology of 70-15, ΔMocsn1, Mocsn1-C, ΔMocsn2, Mocsn2-C, ΔMocsn3, Mocsn3-C, ΔMocsn4, Mocsn4-C, ΔMocsn7a, and Mocsn7a-C. The strains were grown on CM and MM plates for 9 days. (B) Statistical analysis of the colony growth diameter. The data were analyzed using GraphPad Prism 8.0 software. The error bars represent the standard deviations. ***P < 0.001. (C) Conidiophores of 70-15, ΔMocsn1, Mocsn1-C, ΔMocsn2, Mocsn2-C, ΔMocsn3, Mocsn3-C, ΔMocsn4, Mocsn4-C, ΔMocsn7a, and Mocsn7a-C. The strains were cultivated in an incubator at 25 ℃ for 9 days and observed under an optical microscope. (D) Disease spots of detached barley leaves inoculated with mycelial plugs from 70-15, ΔMocsn1, Mocsn1-C, ΔMocsn2, Mocsn2-C, ΔMocsn3, Mocsn3-C, ΔMocsn4, Mocsn4-C, ΔMocsn7a, and Mocsn7a-C. Leaves were cultured at 25 ℃ for 4 days after inoculation. Figure S3. CSN subunits are involved in the regulation of autophagy in M. oryzae. (A) Yeast two-hybrid assays were used to detect the interactions between CSN subunits and Atg-related proteins. Pairs of pGBKT7-53 and PGADT7-T were used as positive controls. Yeast transformants carrying the indicated constructs were plated onto selective plates supplemented without Leu/Trp or Leu/Trp/His/Ade for growth assays. (B) Autophagic flux analysis of GFP-MoAtg8 in 70-15 and ΔMo [file 12964_2024_1598_MOESM1_ESM.zip › Supplementary files/Figure6F-Lysate-A14Flag(left).tif]

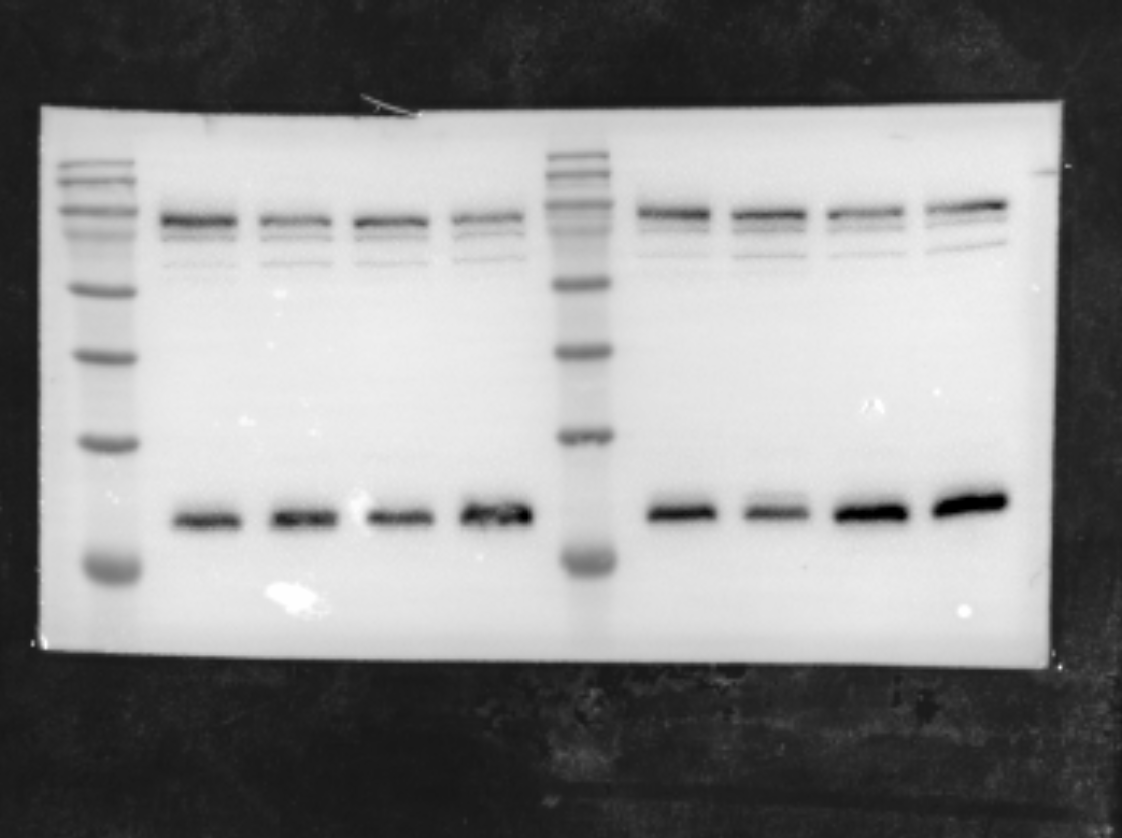

Supplement: Supplementary file 1 — Additional file 1: Figure S1. Verification of deletion mutants by PCR and quantitative real-time PCR. (A) Rice blast fungus knockout model. (B) PCR detection was performed on the null mutants, and the recombinant fragment (LF) was cloned from the positive strain but not from the WT strain. The 500-bp characteristic fragment (SF) of the target gene was cloned in the WT strain but not in the mutants. TUBULIN was used as a positive control. (C) The copy number of the resistance gene HPH in the deletion mutants was verified by quantitative real-time PCR. Figure S2. The targeted gene destruction of CSN subunits decreased the growth, sporulation, and pathogenicity of M. oryzae. (A) Colony morphology of 70-15, ΔMocsn1, Mocsn1-C, ΔMocsn2, Mocsn2-C, ΔMocsn3, Mocsn3-C, ΔMocsn4, Mocsn4-C, ΔMocsn7a, and Mocsn7a-C. The strains were grown on CM and MM plates for 9 days. (B) Statistical analysis of the colony growth diameter. The data were analyzed using GraphPad Prism 8.0 software. The error bars represent the standard deviations. ***P < 0.001. (C) Conidiophores of 70-15, ΔMocsn1, Mocsn1-C, ΔMocsn2, Mocsn2-C, ΔMocsn3, Mocsn3-C, ΔMocsn4, Mocsn4-C, ΔMocsn7a, and Mocsn7a-C. The strains were cultivated in an incubator at 25 ℃ for 9 days and observed under an optical microscope. (D) Disease spots of detached barley leaves inoculated with mycelial plugs from 70-15, ΔMocsn1, Mocsn1-C, ΔMocsn2, Mocsn2-C, ΔMocsn3, Mocsn3-C, ΔMocsn4, Mocsn4-C, ΔMocsn7a, and Mocsn7a-C. Leaves were cultured at 25 ℃ for 4 days after inoculation. Figure S3. CSN subunits are involved in the regulation of autophagy in M. oryzae. (A) Yeast two-hybrid assays were used to detect the interactions between CSN subunits and Atg-related proteins. Pairs of pGBKT7-53 and PGADT7-T were used as positive controls. Yeast transformants carrying the indicated constructs were plated onto selective plates supplemented without Leu/Trp or Leu/Trp/His/Ade for growth assays. (B) Autophagic flux analysis of GFP-MoAtg8 in 70-15 and ΔMo [file 12964_2024_1598_MOESM1_ESM.zip › Supplementary files/Figure6F-Lysate-A6GFP(left).tif]

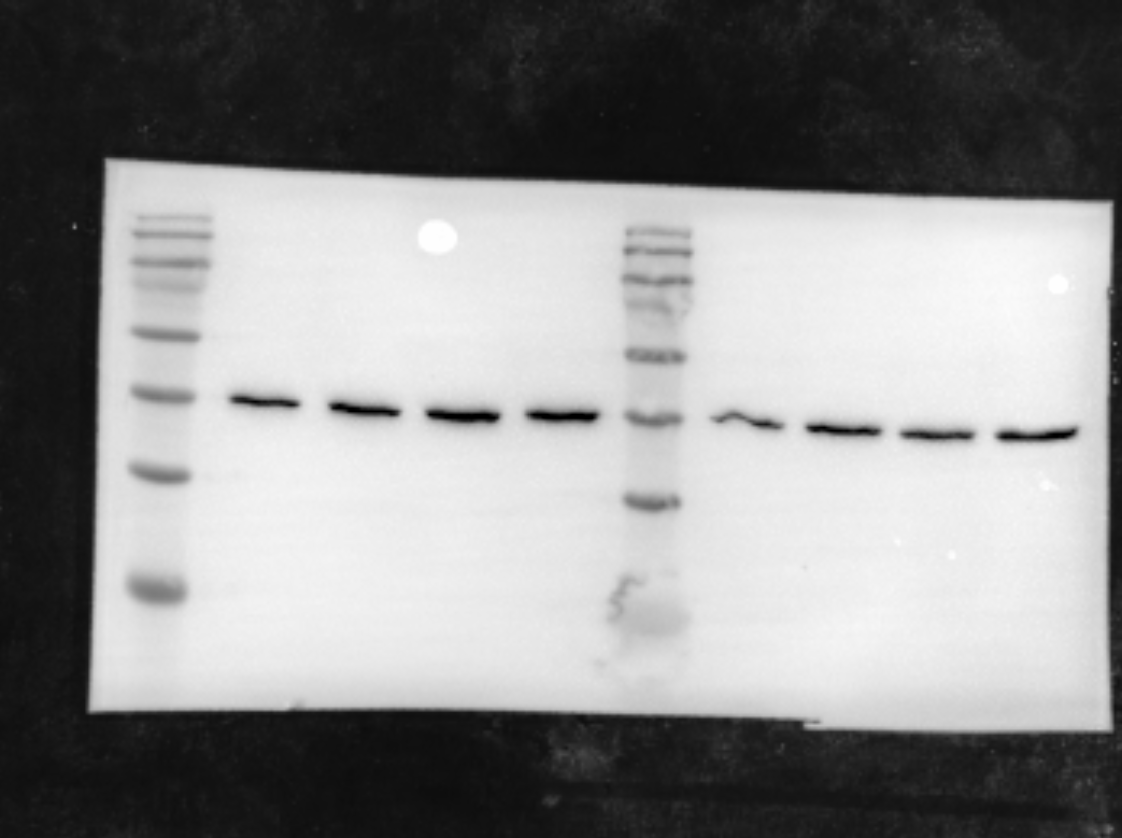

Supplement: Supplementary file 1 — Additional file 1: Figure S1. Verification of deletion mutants by PCR and quantitative real-time PCR. (A) Rice blast fungus knockout model. (B) PCR detection was performed on the null mutants, and the recombinant fragment (LF) was cloned from the positive strain but not from the WT strain. The 500-bp characteristic fragment (SF) of the target gene was cloned in the WT strain but not in the mutants. TUBULIN was used as a positive control. (C) The copy number of the resistance gene HPH in the deletion mutants was verified by quantitative real-time PCR. Figure S2. The targeted gene destruction of CSN subunits decreased the growth, sporulation, and pathogenicity of M. oryzae. (A) Colony morphology of 70-15, ΔMocsn1, Mocsn1-C, ΔMocsn2, Mocsn2-C, ΔMocsn3, Mocsn3-C, ΔMocsn4, Mocsn4-C, ΔMocsn7a, and Mocsn7a-C. The strains were grown on CM and MM plates for 9 days. (B) Statistical analysis of the colony growth diameter. The data were analyzed using GraphPad Prism 8.0 software. The error bars represent the standard deviations. ***P < 0.001. (C) Conidiophores of 70-15, ΔMocsn1, Mocsn1-C, ΔMocsn2, Mocsn2-C, ΔMocsn3, Mocsn3-C, ΔMocsn4, Mocsn4-C, ΔMocsn7a, and Mocsn7a-C. The strains were cultivated in an incubator at 25 ℃ for 9 days and observed under an optical microscope. (D) Disease spots of detached barley leaves inoculated with mycelial plugs from 70-15, ΔMocsn1, Mocsn1-C, ΔMocsn2, Mocsn2-C, ΔMocsn3, Mocsn3-C, ΔMocsn4, Mocsn4-C, ΔMocsn7a, and Mocsn7a-C. Leaves were cultured at 25 ℃ for 4 days after inoculation. Figure S3. CSN subunits are involved in the regulation of autophagy in M. oryzae. (A) Yeast two-hybrid assays were used to detect the interactions between CSN subunits and Atg-related proteins. Pairs of pGBKT7-53 and PGADT7-T were used as positive controls. Yeast transformants carrying the indicated constructs were plated onto selective plates supplemented without Leu/Trp or Leu/Trp/His/Ade for growth assays. (B) Autophagic flux analysis of GFP-MoAtg8 in 70-15 and ΔMo [file 12964_2024_1598_MOESM1_ESM.zip › Supplementary files/Figure6F-Lysate-Actin(left).tif]

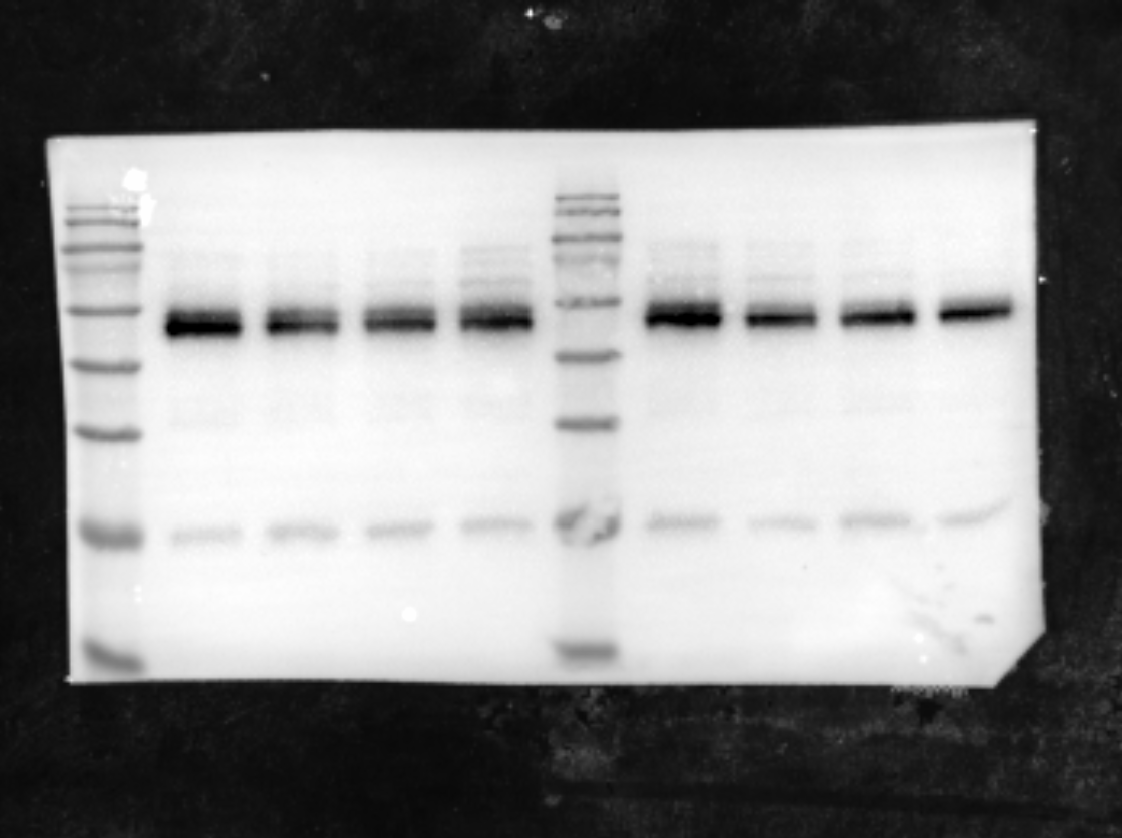

Supplement: Supplementary file 1 — Additional file 1: Figure S1. Verification of deletion mutants by PCR and quantitative real-time PCR. (A) Rice blast fungus knockout model. (B) PCR detection was performed on the null mutants, and the recombinant fragment (LF) was cloned from the positive strain but not from the WT strain. The 500-bp characteristic fragment (SF) of the target gene was cloned in the WT strain but not in the mutants. TUBULIN was used as a positive control. (C) The copy number of the resistance gene HPH in the deletion mutants was verified by quantitative real-time PCR. Figure S2. The targeted gene destruction of CSN subunits decreased the growth, sporulation, and pathogenicity of M. oryzae. (A) Colony morphology of 70-15, ΔMocsn1, Mocsn1-C, ΔMocsn2, Mocsn2-C, ΔMocsn3, Mocsn3-C, ΔMocsn4, Mocsn4-C, ΔMocsn7a, and Mocsn7a-C. The strains were grown on CM and MM plates for 9 days. (B) Statistical analysis of the colony growth diameter. The data were analyzed using GraphPad Prism 8.0 software. The error bars represent the standard deviations. ***P < 0.001. (C) Conidiophores of 70-15, ΔMocsn1, Mocsn1-C, ΔMocsn2, Mocsn2-C, ΔMocsn3, Mocsn3-C, ΔMocsn4, Mocsn4-C, ΔMocsn7a, and Mocsn7a-C. The strains were cultivated in an incubator at 25 ℃ for 9 days and observed under an optical microscope. (D) Disease spots of detached barley leaves inoculated with mycelial plugs from 70-15, ΔMocsn1, Mocsn1-C, ΔMocsn2, Mocsn2-C, ΔMocsn3, Mocsn3-C, ΔMocsn4, Mocsn4-C, ΔMocsn7a, and Mocsn7a-C. Leaves were cultured at 25 ℃ for 4 days after inoculation. Figure S3. CSN subunits are involved in the regulation of autophagy in M. oryzae. (A) Yeast two-hybrid assays were used to detect the interactions between CSN subunits and Atg-related proteins. Pairs of pGBKT7-53 and PGADT7-T were used as positive controls. Yeast transformants carrying the indicated constructs were plated onto selective plates supplemented without Leu/Trp or Leu/Trp/His/Ade for growth assays. (B) Autophagic flux analysis of GFP-MoAtg8 in 70-15 and ΔMo [file 12964_2024_1598_MOESM1_ESM.zip › Supplementary files/Figure6G-IP-A14FLAG(left).tif]

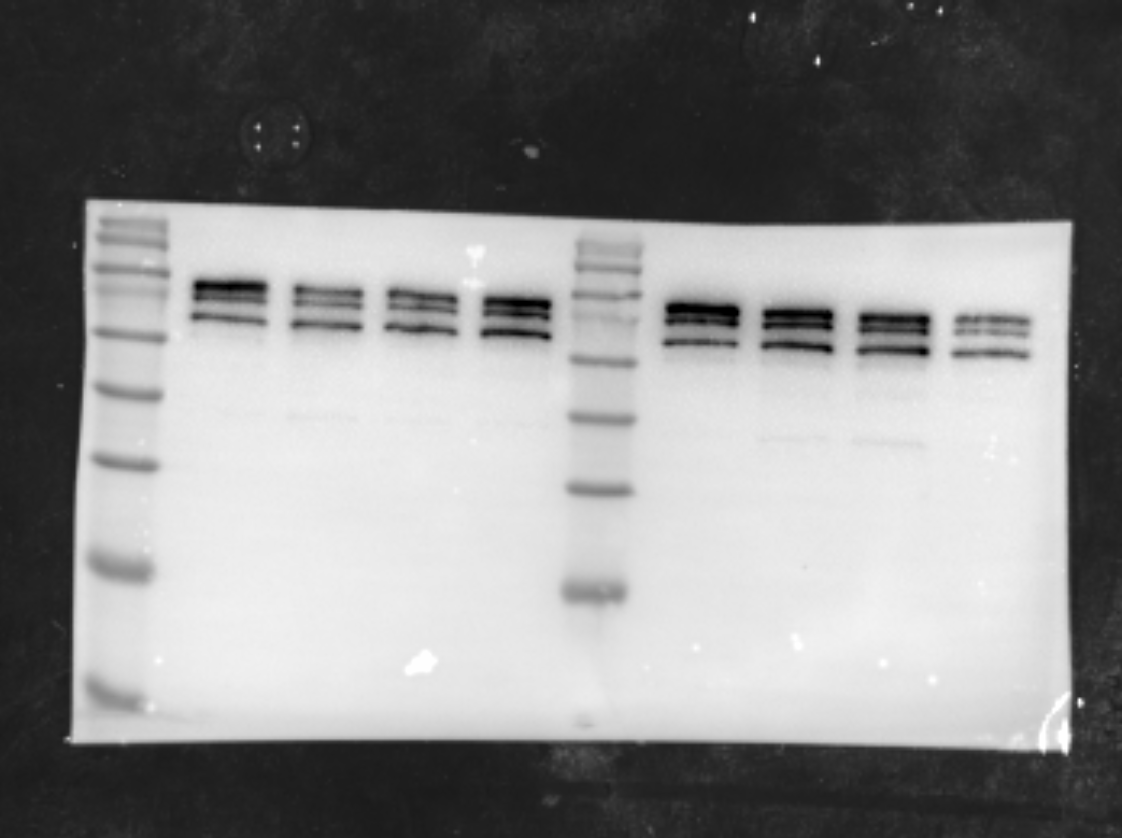

Supplement: Supplementary file 1 — Additional file 1: Figure S1. Verification of deletion mutants by PCR and quantitative real-time PCR. (A) Rice blast fungus knockout model. (B) PCR detection was performed on the null mutants, and the recombinant fragment (LF) was cloned from the positive strain but not from the WT strain. The 500-bp characteristic fragment (SF) of the target gene was cloned in the WT strain but not in the mutants. TUBULIN was used as a positive control. (C) The copy number of the resistance gene HPH in the deletion mutants was verified by quantitative real-time PCR. Figure S2. The targeted gene destruction of CSN subunits decreased the growth, sporulation, and pathogenicity of M. oryzae. (A) Colony morphology of 70-15, ΔMocsn1, Mocsn1-C, ΔMocsn2, Mocsn2-C, ΔMocsn3, Mocsn3-C, ΔMocsn4, Mocsn4-C, ΔMocsn7a, and Mocsn7a-C. The strains were grown on CM and MM plates for 9 days. (B) Statistical analysis of the colony growth diameter. The data were analyzed using GraphPad Prism 8.0 software. The error bars represent the standard deviations. ***P < 0.001. (C) Conidiophores of 70-15, ΔMocsn1, Mocsn1-C, ΔMocsn2, Mocsn2-C, ΔMocsn3, Mocsn3-C, ΔMocsn4, Mocsn4-C, ΔMocsn7a, and Mocsn7a-C. The strains were cultivated in an incubator at 25 ℃ for 9 days and observed under an optical microscope. (D) Disease spots of detached barley leaves inoculated with mycelial plugs from 70-15, ΔMocsn1, Mocsn1-C, ΔMocsn2, Mocsn2-C, ΔMocsn3, Mocsn3-C, ΔMocsn4, Mocsn4-C, ΔMocsn7a, and Mocsn7a-C. Leaves were cultured at 25 ℃ for 4 days after inoculation. Figure S3. CSN subunits are involved in the regulation of autophagy in M. oryzae. (A) Yeast two-hybrid assays were used to detect the interactions between CSN subunits and Atg-related proteins. Pairs of pGBKT7-53 and PGADT7-T were used as positive controls. Yeast transformants carrying the indicated constructs were plated onto selective plates supplemented without Leu/Trp or Leu/Trp/His/Ade for growth assays. (B) Autophagic flux analysis of GFP-MoAtg8 in 70-15 and ΔMo [file 12964_2024_1598_MOESM1_ESM.zip › Supplementary files/Figure6G-IP-A6GFP(left).tif]

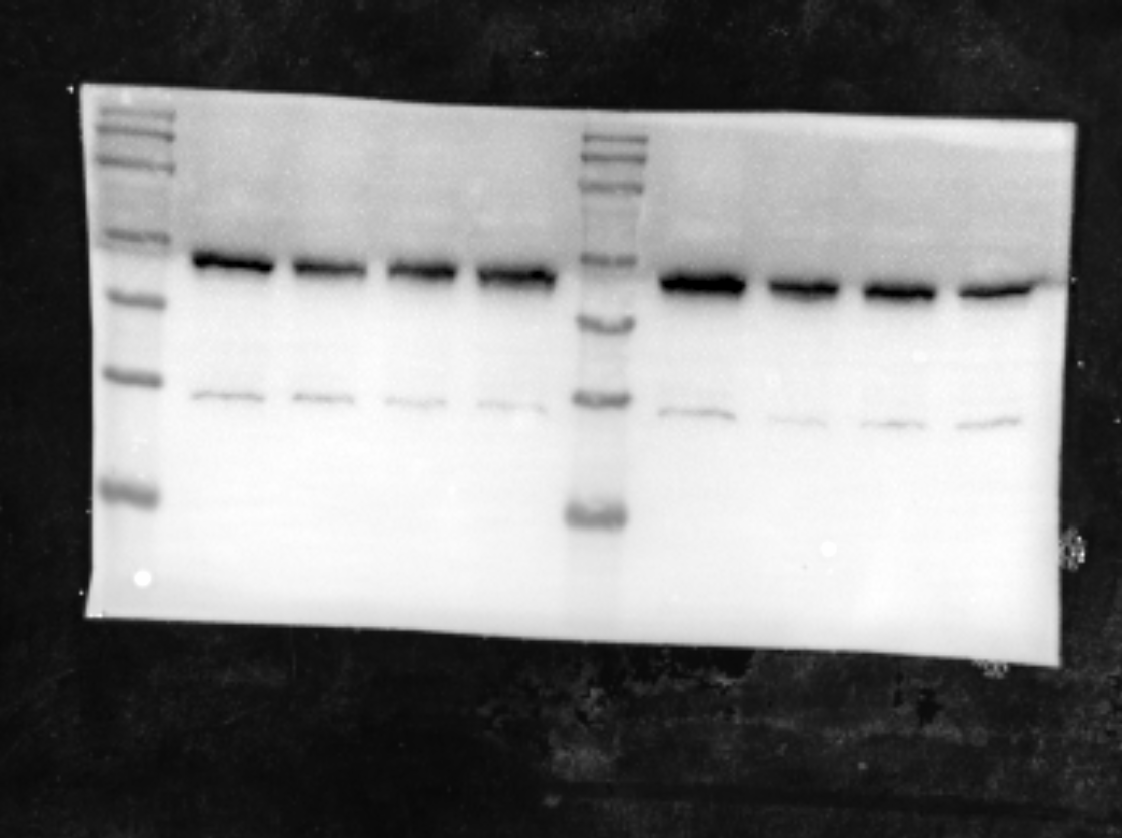

Supplement: Supplementary file 1 — Additional file 1: Figure S1. Verification of deletion mutants by PCR and quantitative real-time PCR. (A) Rice blast fungus knockout model. (B) PCR detection was performed on the null mutants, and the recombinant fragment (LF) was cloned from the positive strain but not from the WT strain. The 500-bp characteristic fragment (SF) of the target gene was cloned in the WT strain but not in the mutants. TUBULIN was used as a positive control. (C) The copy number of the resistance gene HPH in the deletion mutants was verified by quantitative real-time PCR. Figure S2. The targeted gene destruction of CSN subunits decreased the growth, sporulation, and pathogenicity of M. oryzae. (A) Colony morphology of 70-15, ΔMocsn1, Mocsn1-C, ΔMocsn2, Mocsn2-C, ΔMocsn3, Mocsn3-C, ΔMocsn4, Mocsn4-C, ΔMocsn7a, and Mocsn7a-C. The strains were grown on CM and MM plates for 9 days. (B) Statistical analysis of the colony growth diameter. The data were analyzed using GraphPad Prism 8.0 software. The error bars represent the standard deviations. ***P < 0.001. (C) Conidiophores of 70-15, ΔMocsn1, Mocsn1-C, ΔMocsn2, Mocsn2-C, ΔMocsn3, Mocsn3-C, ΔMocsn4, Mocsn4-C, ΔMocsn7a, and Mocsn7a-C. The strains were cultivated in an incubator at 25 ℃ for 9 days and observed under an optical microscope. (D) Disease spots of detached barley leaves inoculated with mycelial plugs from 70-15, ΔMocsn1, Mocsn1-C, ΔMocsn2, Mocsn2-C, ΔMocsn3, Mocsn3-C, ΔMocsn4, Mocsn4-C, ΔMocsn7a, and Mocsn7a-C. Leaves were cultured at 25 ℃ for 4 days after inoculation. Figure S3. CSN subunits are involved in the regulation of autophagy in M. oryzae. (A) Yeast two-hybrid assays were used to detect the interactions between CSN subunits and Atg-related proteins. Pairs of pGBKT7-53 and PGADT7-T were used as positive controls. Yeast transformants carrying the indicated constructs were plated onto selective plates supplemented without Leu/Trp or Leu/Trp/His/Ade for growth assays. (B) Autophagic flux analysis of GFP-MoAtg8 in 70-15 and ΔMo [file 12964_2024_1598_MOESM1_ESM.zip › Supplementary files/Figure6G-Lysate-A14Flag(left).tif]

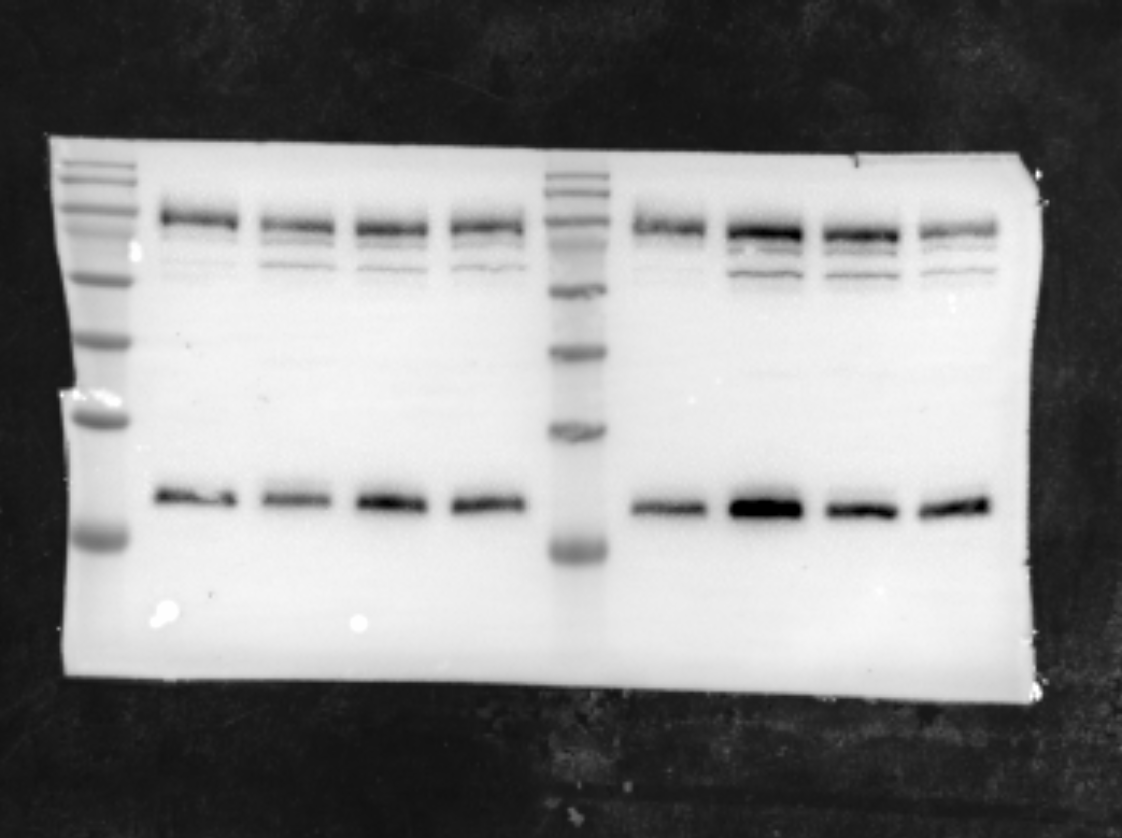

Supplement: Supplementary file 1 — Additional file 1: Figure S1. Verification of deletion mutants by PCR and quantitative real-time PCR. (A) Rice blast fungus knockout model. (B) PCR detection was performed on the null mutants, and the recombinant fragment (LF) was cloned from the positive strain but not from the WT strain. The 500-bp characteristic fragment (SF) of the target gene was cloned in the WT strain but not in the mutants. TUBULIN was used as a positive control. (C) The copy number of the resistance gene HPH in the deletion mutants was verified by quantitative real-time PCR. Figure S2. The targeted gene destruction of CSN subunits decreased the growth, sporulation, and pathogenicity of M. oryzae. (A) Colony morphology of 70-15, ΔMocsn1, Mocsn1-C, ΔMocsn2, Mocsn2-C, ΔMocsn3, Mocsn3-C, ΔMocsn4, Mocsn4-C, ΔMocsn7a, and Mocsn7a-C. The strains were grown on CM and MM plates for 9 days. (B) Statistical analysis of the colony growth diameter. The data were analyzed using GraphPad Prism 8.0 software. The error bars represent the standard deviations. ***P < 0.001. (C) Conidiophores of 70-15, ΔMocsn1, Mocsn1-C, ΔMocsn2, Mocsn2-C, ΔMocsn3, Mocsn3-C, ΔMocsn4, Mocsn4-C, ΔMocsn7a, and Mocsn7a-C. The strains were cultivated in an incubator at 25 ℃ for 9 days and observed under an optical microscope. (D) Disease spots of detached barley leaves inoculated with mycelial plugs from 70-15, ΔMocsn1, Mocsn1-C, ΔMocsn2, Mocsn2-C, ΔMocsn3, Mocsn3-C, ΔMocsn4, Mocsn4-C, ΔMocsn7a, and Mocsn7a-C. Leaves were cultured at 25 ℃ for 4 days after inoculation. Figure S3. CSN subunits are involved in the regulation of autophagy in M. oryzae. (A) Yeast two-hybrid assays were used to detect the interactions between CSN subunits and Atg-related proteins. Pairs of pGBKT7-53 and PGADT7-T were used as positive controls. Yeast transformants carrying the indicated constructs were plated onto selective plates supplemented without Leu/Trp or Leu/Trp/His/Ade for growth assays. (B) Autophagic flux analysis of GFP-MoAtg8 in 70-15 and ΔMo [file 12964_2024_1598_MOESM1_ESM.zip › Supplementary files/Figure6G-Lysate-A6GFP(left).tif]

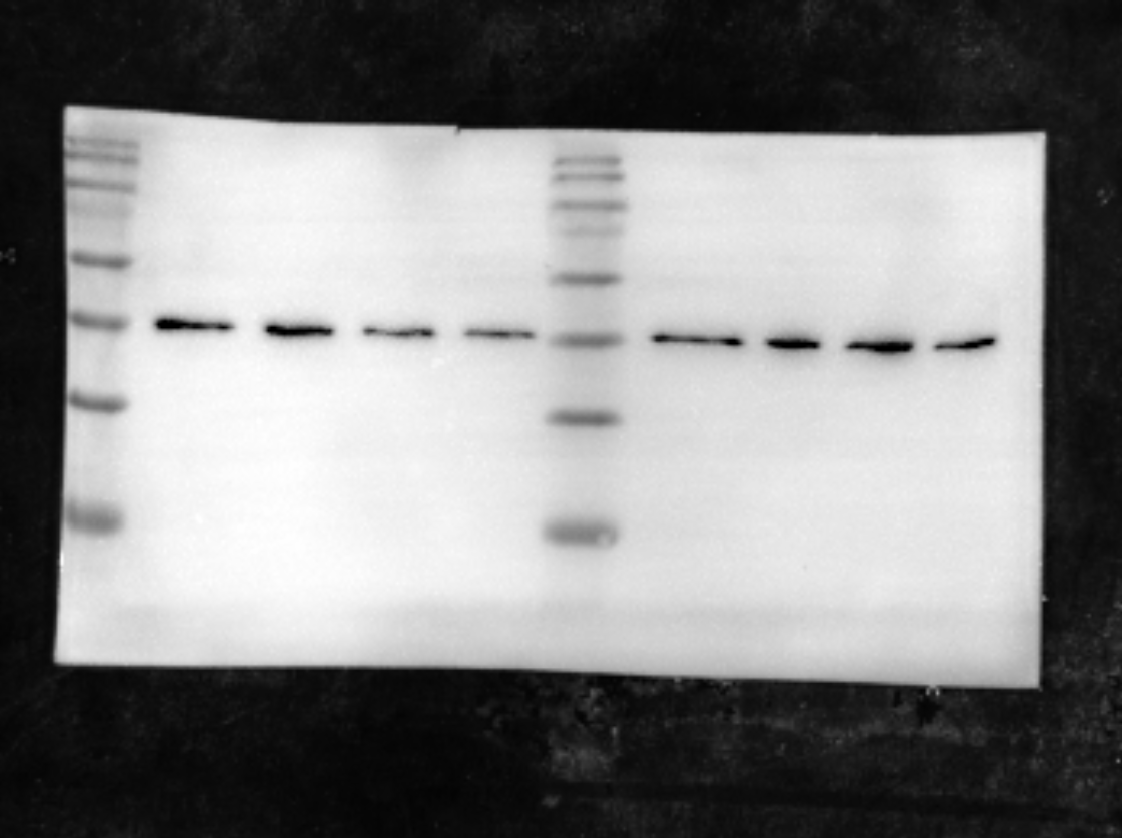

Supplement: Supplementary file 1 — Additional file 1: Figure S1. Verification of deletion mutants by PCR and quantitative real-time PCR. (A) Rice blast fungus knockout model. (B) PCR detection was performed on the null mutants, and the recombinant fragment (LF) was cloned from the positive strain but not from the WT strain. The 500-bp characteristic fragment (SF) of the target gene was cloned in the WT strain but not in the mutants. TUBULIN was used as a positive control. (C) The copy number of the resistance gene HPH in the deletion mutants was verified by quantitative real-time PCR. Figure S2. The targeted gene destruction of CSN subunits decreased the growth, sporulation, and pathogenicity of M. oryzae. (A) Colony morphology of 70-15, ΔMocsn1, Mocsn1-C, ΔMocsn2, Mocsn2-C, ΔMocsn3, Mocsn3-C, ΔMocsn4, Mocsn4-C, ΔMocsn7a, and Mocsn7a-C. The strains were grown on CM and MM plates for 9 days. (B) Statistical analysis of the colony growth diameter. The data were analyzed using GraphPad Prism 8.0 software. The error bars represent the standard deviations. ***P < 0.001. (C) Conidiophores of 70-15, ΔMocsn1, Mocsn1-C, ΔMocsn2, Mocsn2-C, ΔMocsn3, Mocsn3-C, ΔMocsn4, Mocsn4-C, ΔMocsn7a, and Mocsn7a-C. The strains were cultivated in an incubator at 25 ℃ for 9 days and observed under an optical microscope. (D) Disease spots of detached barley leaves inoculated with mycelial plugs from 70-15, ΔMocsn1, Mocsn1-C, ΔMocsn2, Mocsn2-C, ΔMocsn3, Mocsn3-C, ΔMocsn4, Mocsn4-C, ΔMocsn7a, and Mocsn7a-C. Leaves were cultured at 25 ℃ for 4 days after inoculation. Figure S3. CSN subunits are involved in the regulation of autophagy in M. oryzae. (A) Yeast two-hybrid assays were used to detect the interactions between CSN subunits and Atg-related proteins. Pairs of pGBKT7-53 and PGADT7-T were used as positive controls. Yeast transformants carrying the indicated constructs were plated onto selective plates supplemented without Leu/Trp or Leu/Trp/His/Ade for growth assays. (B) Autophagic flux analysis of GFP-MoAtg8 in 70-15 and ΔMo [file 12964_2024_1598_MOESM1_ESM.zip › Supplementary files/Figure6G-Lysate-Actin(left).tif]

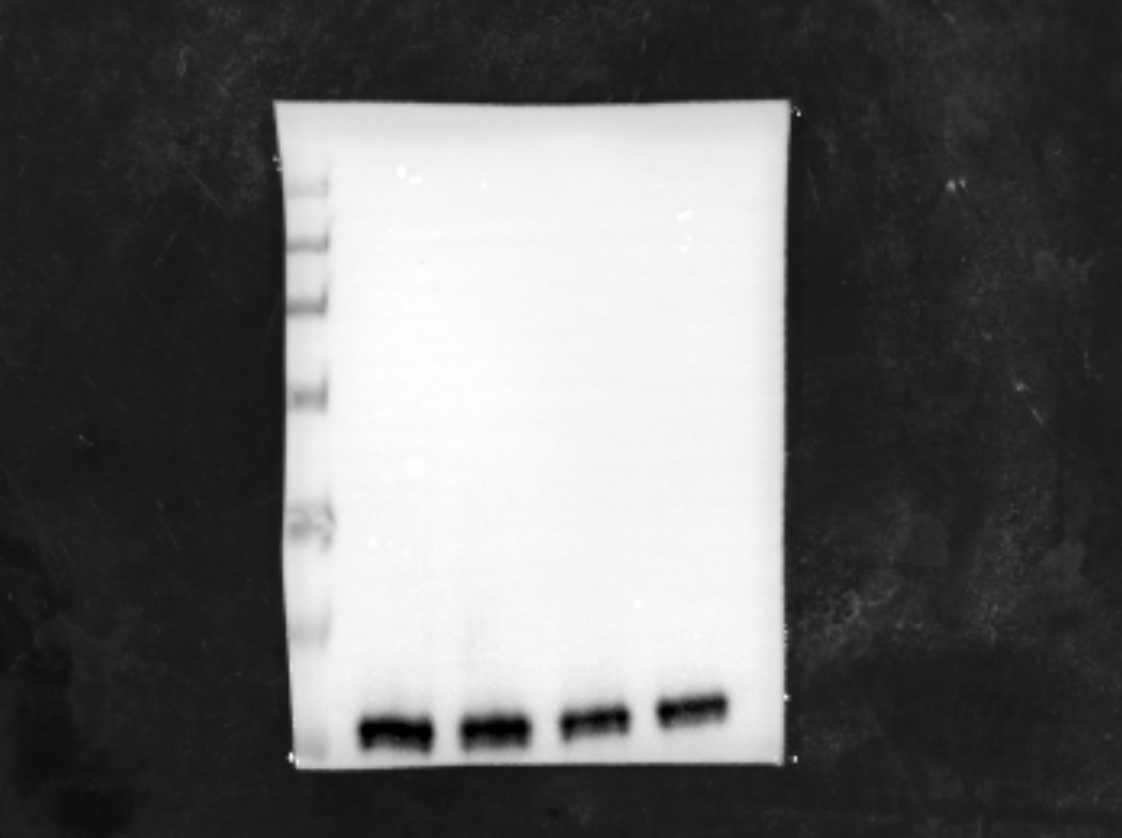

Supplement: Supplementary file 1 — Additional file 1: Figure S1. Verification of deletion mutants by PCR and quantitative real-time PCR. (A) Rice blast fungus knockout model. (B) PCR detection was performed on the null mutants, and the recombinant fragment (LF) was cloned from the positive strain but not from the WT strain. The 500-bp characteristic fragment (SF) of the target gene was cloned in the WT strain but not in the mutants. TUBULIN was used as a positive control. (C) The copy number of the resistance gene HPH in the deletion mutants was verified by quantitative real-time PCR. Figure S2. The targeted gene destruction of CSN subunits decreased the growth, sporulation, and pathogenicity of M. oryzae. (A) Colony morphology of 70-15, ΔMocsn1, Mocsn1-C, ΔMocsn2, Mocsn2-C, ΔMocsn3, Mocsn3-C, ΔMocsn4, Mocsn4-C, ΔMocsn7a, and Mocsn7a-C. The strains were grown on CM and MM plates for 9 days. (B) Statistical analysis of the colony growth diameter. The data were analyzed using GraphPad Prism 8.0 software. The error bars represent the standard deviations. ***P < 0.001. (C) Conidiophores of 70-15, ΔMocsn1, Mocsn1-C, ΔMocsn2, Mocsn2-C, ΔMocsn3, Mocsn3-C, ΔMocsn4, Mocsn4-C, ΔMocsn7a, and Mocsn7a-C. The strains were cultivated in an incubator at 25 ℃ for 9 days and observed under an optical microscope. (D) Disease spots of detached barley leaves inoculated with mycelial plugs from 70-15, ΔMocsn1, Mocsn1-C, ΔMocsn2, Mocsn2-C, ΔMocsn3, Mocsn3-C, ΔMocsn4, Mocsn4-C, ΔMocsn7a, and Mocsn7a-C. Leaves were cultured at 25 ℃ for 4 days after inoculation. Figure S3. CSN subunits are involved in the regulation of autophagy in M. oryzae. (A) Yeast two-hybrid assays were used to detect the interactions between CSN subunits and Atg-related proteins. Pairs of pGBKT7-53 and PGADT7-T were used as positive controls. Yeast transformants carrying the indicated constructs were plated onto selective plates supplemented without Leu/Trp or Leu/Trp/His/Ade for growth assays. (B) Autophagic flux analysis of GFP-MoAtg8 in 70-15 and ΔMo [file 12964_2024_1598_MOESM1_ESM.zip › Supplementary files/Figure6H-Flag.tif]

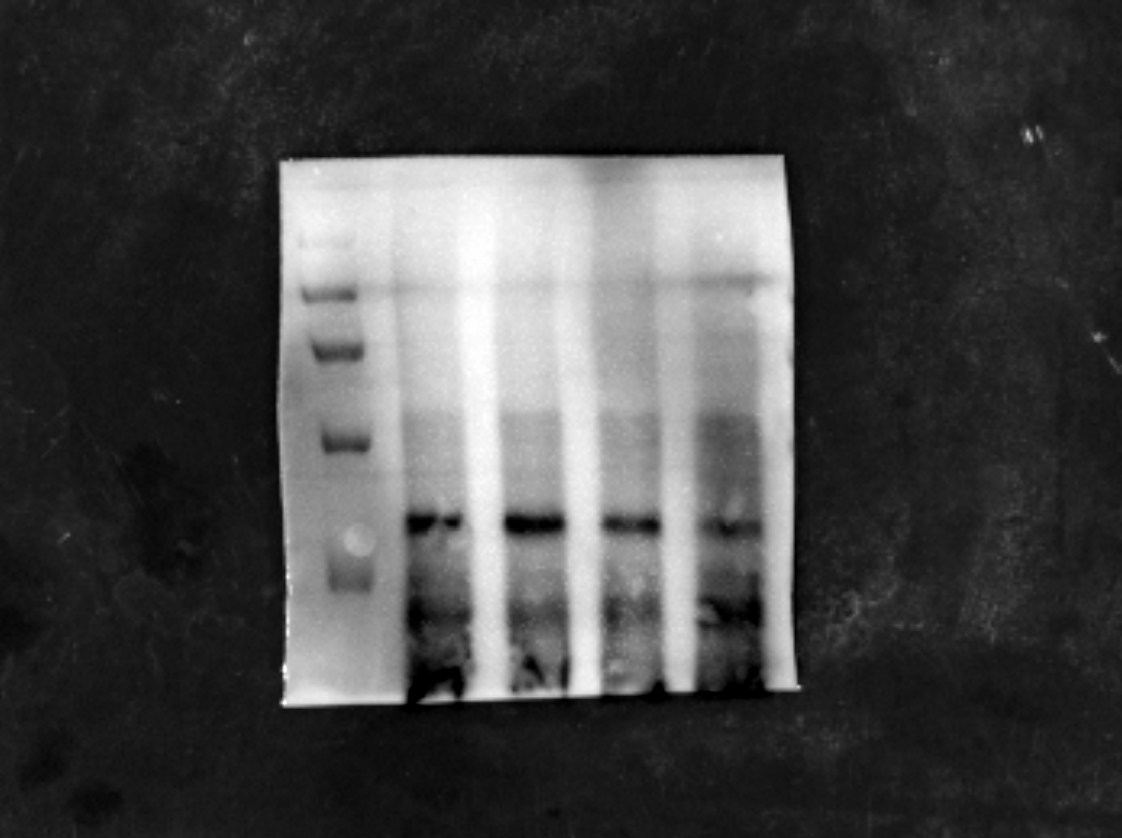

Supplement: Supplementary file 1 — Additional file 1: Figure S1. Verification of deletion mutants by PCR and quantitative real-time PCR. (A) Rice blast fungus knockout model. (B) PCR detection was performed on the null mutants, and the recombinant fragment (LF) was cloned from the positive strain but not from the WT strain. The 500-bp characteristic fragment (SF) of the target gene was cloned in the WT strain but not in the mutants. TUBULIN was used as a positive control. (C) The copy number of the resistance gene HPH in the deletion mutants was verified by quantitative real-time PCR. Figure S2. The targeted gene destruction of CSN subunits decreased the growth, sporulation, and pathogenicity of M. oryzae. (A) Colony morphology of 70-15, ΔMocsn1, Mocsn1-C, ΔMocsn2, Mocsn2-C, ΔMocsn3, Mocsn3-C, ΔMocsn4, Mocsn4-C, ΔMocsn7a, and Mocsn7a-C. The strains were grown on CM and MM plates for 9 days. (B) Statistical analysis of the colony growth diameter. The data were analyzed using GraphPad Prism 8.0 software. The error bars represent the standard deviations. ***P < 0.001. (C) Conidiophores of 70-15, ΔMocsn1, Mocsn1-C, ΔMocsn2, Mocsn2-C, ΔMocsn3, Mocsn3-C, ΔMocsn4, Mocsn4-C, ΔMocsn7a, and Mocsn7a-C. The strains were cultivated in an incubator at 25 ℃ for 9 days and observed under an optical microscope. (D) Disease spots of detached barley leaves inoculated with mycelial plugs from 70-15, ΔMocsn1, Mocsn1-C, ΔMocsn2, Mocsn2-C, ΔMocsn3, Mocsn3-C, ΔMocsn4, Mocsn4-C, ΔMocsn7a, and Mocsn7a-C. Leaves were cultured at 25 ℃ for 4 days after inoculation. Figure S3. CSN subunits are involved in the regulation of autophagy in M. oryzae. (A) Yeast two-hybrid assays were used to detect the interactions between CSN subunits and Atg-related proteins. Pairs of pGBKT7-53 and PGADT7-T were used as positive controls. Yeast transformants carrying the indicated constructs were plated onto selective plates supplemented without Leu/Trp or Leu/Trp/His/Ade for growth assays. (B) Autophagic flux analysis of GFP-MoAtg8 in 70-15 and ΔMo [file 12964_2024_1598_MOESM1_ESM.zip › Supplementary files/Figure6H-Ubi.tif]

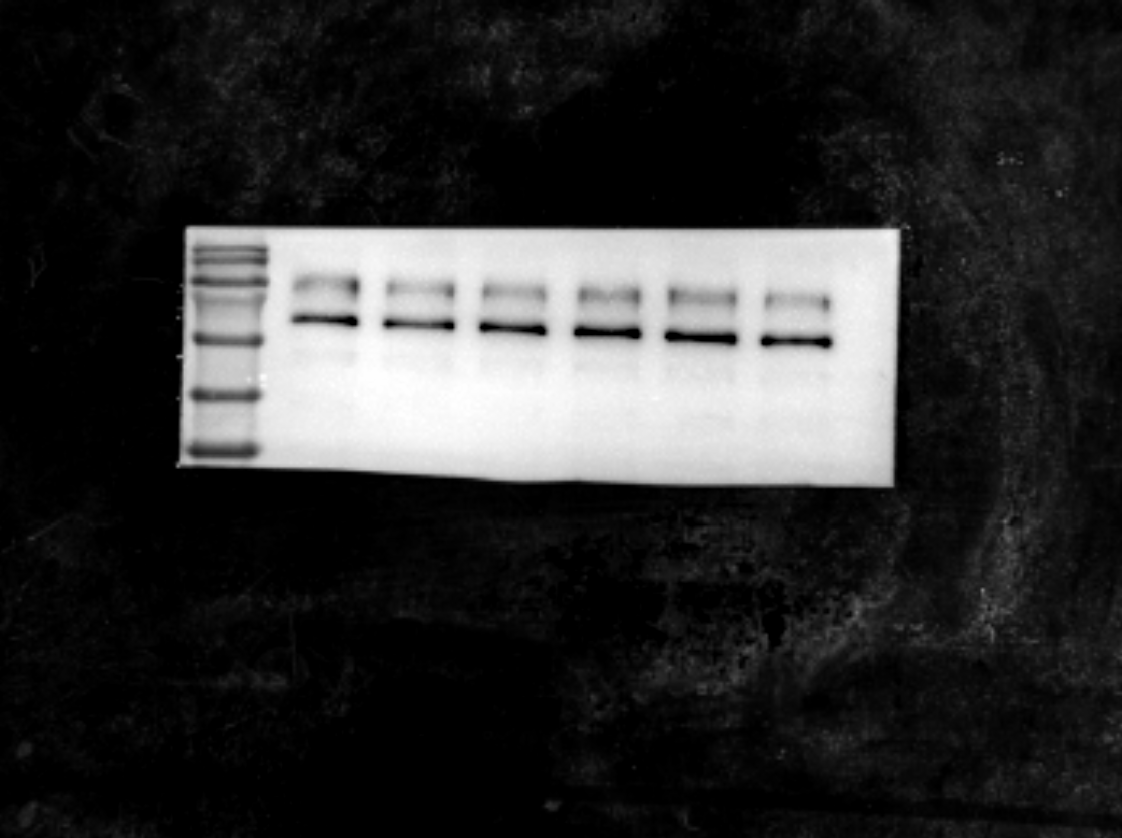

Supplement: Supplementary file 1 — Additional file 1: Figure S1. Verification of deletion mutants by PCR and quantitative real-time PCR. (A) Rice blast fungus knockout model. (B) PCR detection was performed on the null mutants, and the recombinant fragment (LF) was cloned from the positive strain but not from the WT strain. The 500-bp characteristic fragment (SF) of the target gene was cloned in the WT strain but not in the mutants. TUBULIN was used as a positive control. (C) The copy number of the resistance gene HPH in the deletion mutants was verified by quantitative real-time PCR. Figure S2. The targeted gene destruction of CSN subunits decreased the growth, sporulation, and pathogenicity of M. oryzae. (A) Colony morphology of 70-15, ΔMocsn1, Mocsn1-C, ΔMocsn2, Mocsn2-C, ΔMocsn3, Mocsn3-C, ΔMocsn4, Mocsn4-C, ΔMocsn7a, and Mocsn7a-C. The strains were grown on CM and MM plates for 9 days. (B) Statistical analysis of the colony growth diameter. The data were analyzed using GraphPad Prism 8.0 software. The error bars represent the standard deviations. ***P < 0.001. (C) Conidiophores of 70-15, ΔMocsn1, Mocsn1-C, ΔMocsn2, Mocsn2-C, ΔMocsn3, Mocsn3-C, ΔMocsn4, Mocsn4-C, ΔMocsn7a, and Mocsn7a-C. The strains were cultivated in an incubator at 25 ℃ for 9 days and observed under an optical microscope. (D) Disease spots of detached barley leaves inoculated with mycelial plugs from 70-15, ΔMocsn1, Mocsn1-C, ΔMocsn2, Mocsn2-C, ΔMocsn3, Mocsn3-C, ΔMocsn4, Mocsn4-C, ΔMocsn7a, and Mocsn7a-C. Leaves were cultured at 25 ℃ for 4 days after inoculation. Figure S3. CSN subunits are involved in the regulation of autophagy in M. oryzae. (A) Yeast two-hybrid assays were used to detect the interactions between CSN subunits and Atg-related proteins. Pairs of pGBKT7-53 and PGADT7-T were used as positive controls. Yeast transformants carrying the indicated constructs were plated onto selective plates supplemented without Leu/Trp or Leu/Trp/His/Ade for growth assays. (B) Autophagic flux analysis of GFP-MoAtg8 in 70-15 and ΔMo [file 12964_2024_1598_MOESM1_ESM.zip › Supplementary files/Figure6I-A6GFP.tif]

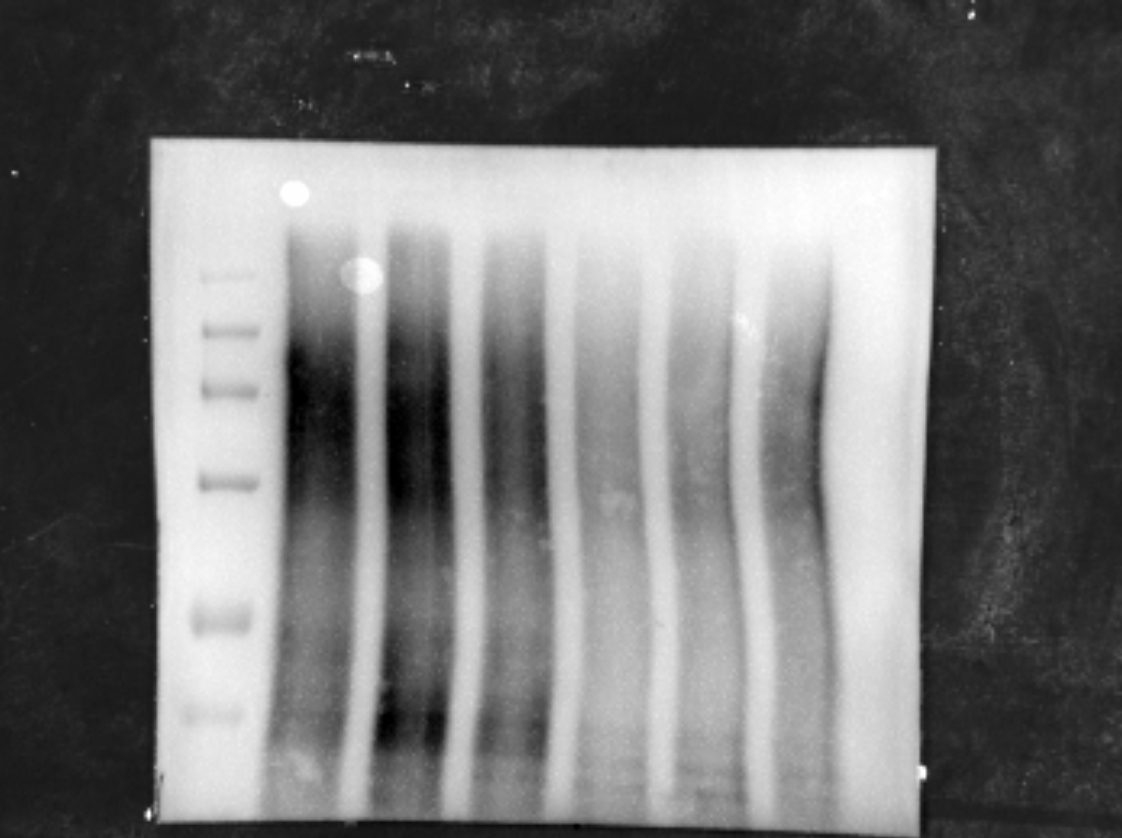

Supplement: Supplementary file 1 — Additional file 1: Figure S1. Verification of deletion mutants by PCR and quantitative real-time PCR. (A) Rice blast fungus knockout model. (B) PCR detection was performed on the null mutants, and the recombinant fragment (LF) was cloned from the positive strain but not from the WT strain. The 500-bp characteristic fragment (SF) of the target gene was cloned in the WT strain but not in the mutants. TUBULIN was used as a positive control. (C) The copy number of the resistance gene HPH in the deletion mutants was verified by quantitative real-time PCR. Figure S2. The targeted gene destruction of CSN subunits decreased the growth, sporulation, and pathogenicity of M. oryzae. (A) Colony morphology of 70-15, ΔMocsn1, Mocsn1-C, ΔMocsn2, Mocsn2-C, ΔMocsn3, Mocsn3-C, ΔMocsn4, Mocsn4-C, ΔMocsn7a, and Mocsn7a-C. The strains were grown on CM and MM plates for 9 days. (B) Statistical analysis of the colony growth diameter. The data were analyzed using GraphPad Prism 8.0 software. The error bars represent the standard deviations. ***P < 0.001. (C) Conidiophores of 70-15, ΔMocsn1, Mocsn1-C, ΔMocsn2, Mocsn2-C, ΔMocsn3, Mocsn3-C, ΔMocsn4, Mocsn4-C, ΔMocsn7a, and Mocsn7a-C. The strains were cultivated in an incubator at 25 ℃ for 9 days and observed under an optical microscope. (D) Disease spots of detached barley leaves inoculated with mycelial plugs from 70-15, ΔMocsn1, Mocsn1-C, ΔMocsn2, Mocsn2-C, ΔMocsn3, Mocsn3-C, ΔMocsn4, Mocsn4-C, ΔMocsn7a, and Mocsn7a-C. Leaves were cultured at 25 ℃ for 4 days after inoculation. Figure S3. CSN subunits are involved in the regulation of autophagy in M. oryzae. (A) Yeast two-hybrid assays were used to detect the interactions between CSN subunits and Atg-related proteins. Pairs of pGBKT7-53 and PGADT7-T were used as positive controls. Yeast transformants carrying the indicated constructs were plated onto selective plates supplemented without Leu/Trp or Leu/Trp/His/Ade for growth assays. (B) Autophagic flux analysis of GFP-MoAtg8 in 70-15 and ΔMo [file 12964_2024_1598_MOESM1_ESM.zip › Supplementary files/Figure6I-K48.tif]

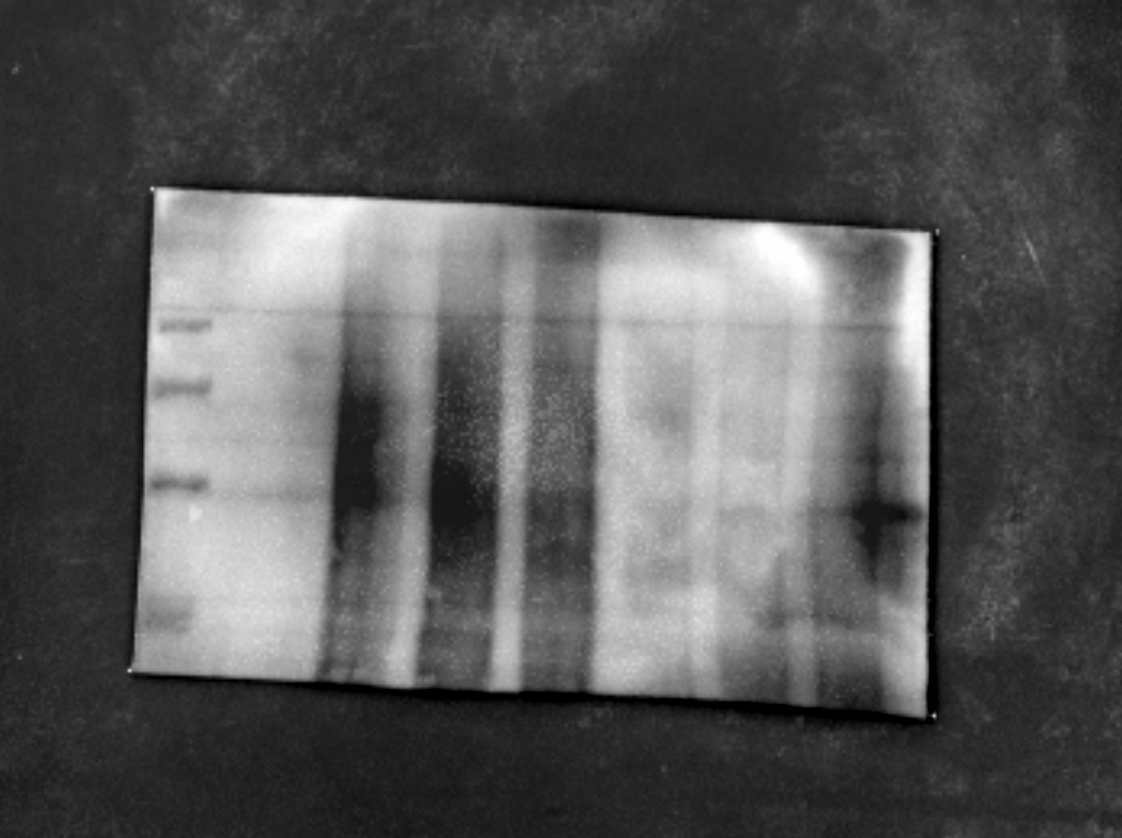

Supplement: Supplementary file 1 — Additional file 1: Figure S1. Verification of deletion mutants by PCR and quantitative real-time PCR. (A) Rice blast fungus knockout model. (B) PCR detection was performed on the null mutants, and the recombinant fragment (LF) was cloned from the positive strain but not from the WT strain. The 500-bp characteristic fragment (SF) of the target gene was cloned in the WT strain but not in the mutants. TUBULIN was used as a positive control. (C) The copy number of the resistance gene HPH in the deletion mutants was verified by quantitative real-time PCR. Figure S2. The targeted gene destruction of CSN subunits decreased the growth, sporulation, and pathogenicity of M. oryzae. (A) Colony morphology of 70-15, ΔMocsn1, Mocsn1-C, ΔMocsn2, Mocsn2-C, ΔMocsn3, Mocsn3-C, ΔMocsn4, Mocsn4-C, ΔMocsn7a, and Mocsn7a-C. The strains were grown on CM and MM plates for 9 days. (B) Statistical analysis of the colony growth diameter. The data were analyzed using GraphPad Prism 8.0 software. The error bars represent the standard deviations. ***P < 0.001. (C) Conidiophores of 70-15, ΔMocsn1, Mocsn1-C, ΔMocsn2, Mocsn2-C, ΔMocsn3, Mocsn3-C, ΔMocsn4, Mocsn4-C, ΔMocsn7a, and Mocsn7a-C. The strains were cultivated in an incubator at 25 ℃ for 9 days and observed under an optical microscope. (D) Disease spots of detached barley leaves inoculated with mycelial plugs from 70-15, ΔMocsn1, Mocsn1-C, ΔMocsn2, Mocsn2-C, ΔMocsn3, Mocsn3-C, ΔMocsn4, Mocsn4-C, ΔMocsn7a, and Mocsn7a-C. Leaves were cultured at 25 ℃ for 4 days after inoculation. Figure S3. CSN subunits are involved in the regulation of autophagy in M. oryzae. (A) Yeast two-hybrid assays were used to detect the interactions between CSN subunits and Atg-related proteins. Pairs of pGBKT7-53 and PGADT7-T were used as positive controls. Yeast transformants carrying the indicated constructs were plated onto selective plates supplemented without Leu/Trp or Leu/Trp/His/Ade for growth assays. (B) Autophagic flux analysis of GFP-MoAtg8 in 70-15 and ΔMo [file 12964_2024_1598_MOESM1_ESM.zip › Supplementary files/Figure6I-UBI.tif]

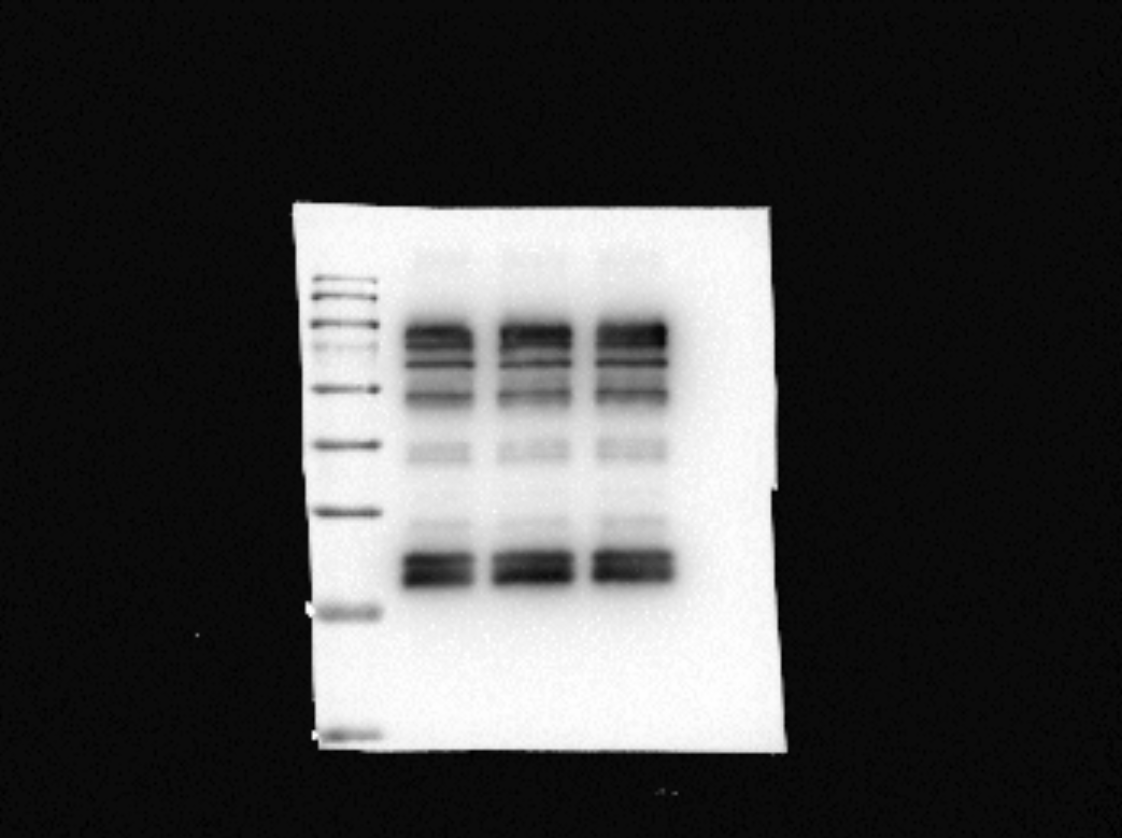

Supplement: Supplementary file 1 — Additional file 1: Figure S1. Verification of deletion mutants by PCR and quantitative real-time PCR. (A) Rice blast fungus knockout model. (B) PCR detection was performed on the null mutants, and the recombinant fragment (LF) was cloned from the positive strain but not from the WT strain. The 500-bp characteristic fragment (SF) of the target gene was cloned in the WT strain but not in the mutants. TUBULIN was used as a positive control. (C) The copy number of the resistance gene HPH in the deletion mutants was verified by quantitative real-time PCR. Figure S2. The targeted gene destruction of CSN subunits decreased the growth, sporulation, and pathogenicity of M. oryzae. (A) Colony morphology of 70-15, ΔMocsn1, Mocsn1-C, ΔMocsn2, Mocsn2-C, ΔMocsn3, Mocsn3-C, ΔMocsn4, Mocsn4-C, ΔMocsn7a, and Mocsn7a-C. The strains were grown on CM and MM plates for 9 days. (B) Statistical analysis of the colony growth diameter. The data were analyzed using GraphPad Prism 8.0 software. The error bars represent the standard deviations. ***P < 0.001. (C) Conidiophores of 70-15, ΔMocsn1, Mocsn1-C, ΔMocsn2, Mocsn2-C, ΔMocsn3, Mocsn3-C, ΔMocsn4, Mocsn4-C, ΔMocsn7a, and Mocsn7a-C. The strains were cultivated in an incubator at 25 ℃ for 9 days and observed under an optical microscope. (D) Disease spots of detached barley leaves inoculated with mycelial plugs from 70-15, ΔMocsn1, Mocsn1-C, ΔMocsn2, Mocsn2-C, ΔMocsn3, Mocsn3-C, ΔMocsn4, Mocsn4-C, ΔMocsn7a, and Mocsn7a-C. Leaves were cultured at 25 ℃ for 4 days after inoculation. Figure S3. CSN subunits are involved in the regulation of autophagy in M. oryzae. (A) Yeast two-hybrid assays were used to detect the interactions between CSN subunits and Atg-related proteins. Pairs of pGBKT7-53 and PGADT7-T were used as positive controls. Yeast transformants carrying the indicated constructs were plated onto selective plates supplemented without Leu/Trp or Leu/Trp/His/Ade for growth assays. (B) Autophagic flux analysis of GFP-MoAtg8 in 70-15 and ΔMo [file 12964_2024_1598_MOESM1_ESM.zip › Supplementary files/Figure6J-A6GFP.tif]

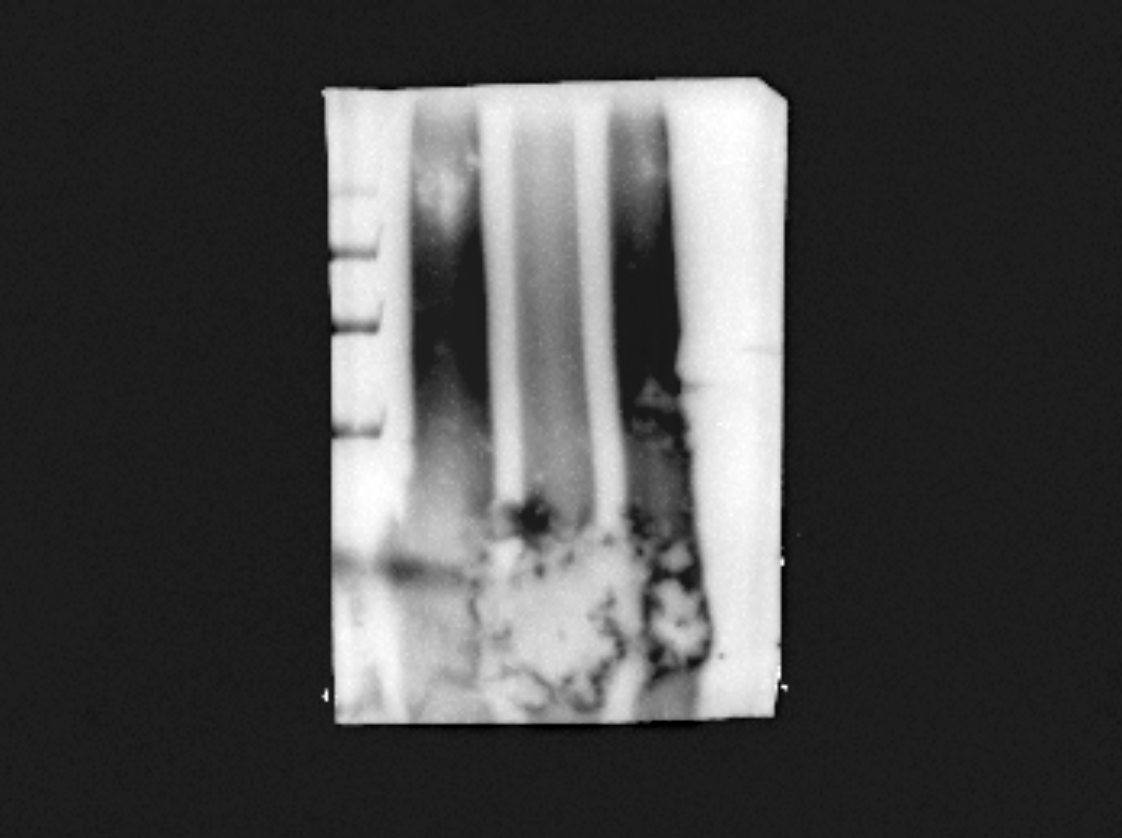

Supplement: Supplementary file 1 — Additional file 1: Figure S1. Verification of deletion mutants by PCR and quantitative real-time PCR. (A) Rice blast fungus knockout model. (B) PCR detection was performed on the null mutants, and the recombinant fragment (LF) was cloned from the positive strain but not from the WT strain. The 500-bp characteristic fragment (SF) of the target gene was cloned in the WT strain but not in the mutants. TUBULIN was used as a positive control. (C) The copy number of the resistance gene HPH in the deletion mutants was verified by quantitative real-time PCR. Figure S2. The targeted gene destruction of CSN subunits decreased the growth, sporulation, and pathogenicity of M. oryzae. (A) Colony morphology of 70-15, ΔMocsn1, Mocsn1-C, ΔMocsn2, Mocsn2-C, ΔMocsn3, Mocsn3-C, ΔMocsn4, Mocsn4-C, ΔMocsn7a, and Mocsn7a-C. The strains were grown on CM and MM plates for 9 days. (B) Statistical analysis of the colony growth diameter. The data were analyzed using GraphPad Prism 8.0 software. The error bars represent the standard deviations. ***P < 0.001. (C) Conidiophores of 70-15, ΔMocsn1, Mocsn1-C, ΔMocsn2, Mocsn2-C, ΔMocsn3, Mocsn3-C, ΔMocsn4, Mocsn4-C, ΔMocsn7a, and Mocsn7a-C. The strains were cultivated in an incubator at 25 ℃ for 9 days and observed under an optical microscope. (D) Disease spots of detached barley leaves inoculated with mycelial plugs from 70-15, ΔMocsn1, Mocsn1-C, ΔMocsn2, Mocsn2-C, ΔMocsn3, Mocsn3-C, ΔMocsn4, Mocsn4-C, ΔMocsn7a, and Mocsn7a-C. Leaves were cultured at 25 ℃ for 4 days after inoculation. Figure S3. CSN subunits are involved in the regulation of autophagy in M. oryzae. (A) Yeast two-hybrid assays were used to detect the interactions between CSN subunits and Atg-related proteins. Pairs of pGBKT7-53 and PGADT7-T were used as positive controls. Yeast transformants carrying the indicated constructs were plated onto selective plates supplemented without Leu/Trp or Leu/Trp/His/Ade for growth assays. (B) Autophagic flux analysis of GFP-MoAtg8 in 70-15 and ΔMo [file 12964_2024_1598_MOESM1_ESM.zip › Supplementary files/Figure6J-K48.tif]

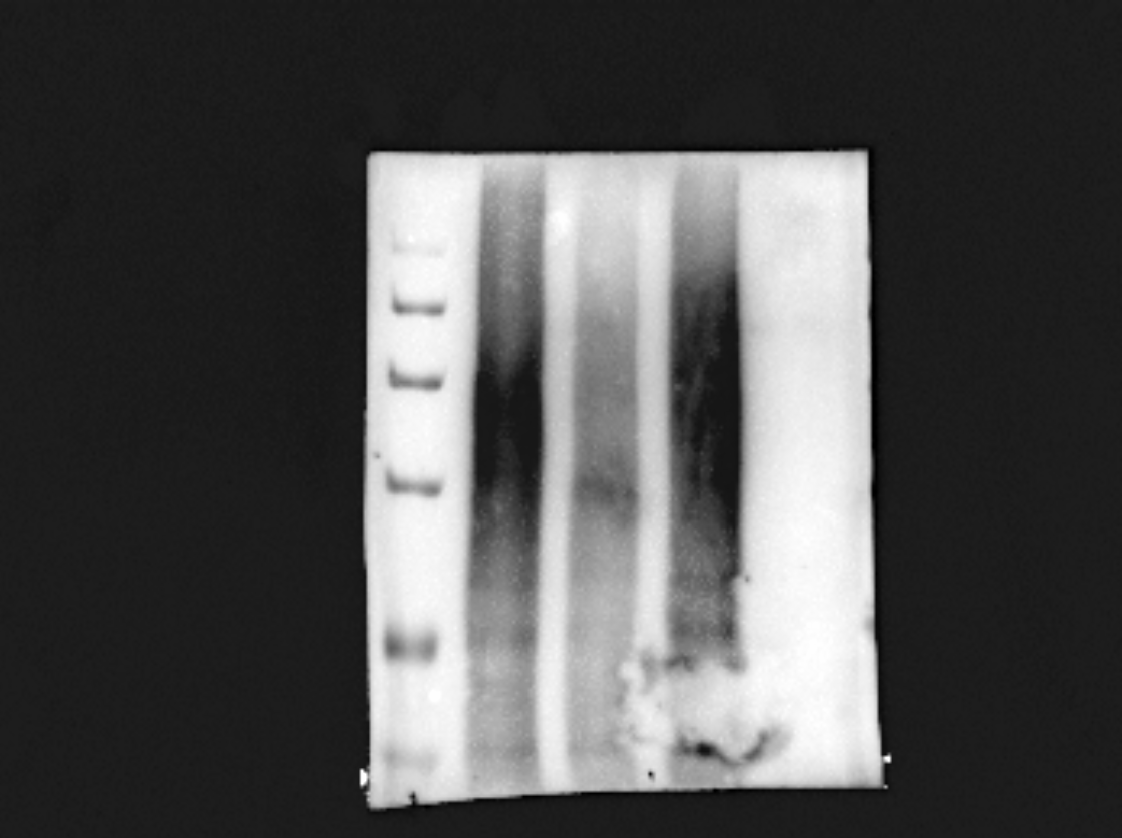

Supplement: Supplementary file 1 — Additional file 1: Figure S1. Verification of deletion mutants by PCR and quantitative real-time PCR. (A) Rice blast fungus knockout model. (B) PCR detection was performed on the null mutants, and the recombinant fragment (LF) was cloned from the positive strain but not from the WT strain. The 500-bp characteristic fragment (SF) of the target gene was cloned in the WT strain but not in the mutants. TUBULIN was used as a positive control. (C) The copy number of the resistance gene HPH in the deletion mutants was verified by quantitative real-time PCR. Figure S2. The targeted gene destruction of CSN subunits decreased the growth, sporulation, and pathogenicity of M. oryzae. (A) Colony morphology of 70-15, ΔMocsn1, Mocsn1-C, ΔMocsn2, Mocsn2-C, ΔMocsn3, Mocsn3-C, ΔMocsn4, Mocsn4-C, ΔMocsn7a, and Mocsn7a-C. The strains were grown on CM and MM plates for 9 days. (B) Statistical analysis of the colony growth diameter. The data were analyzed using GraphPad Prism 8.0 software. The error bars represent the standard deviations. ***P < 0.001. (C) Conidiophores of 70-15, ΔMocsn1, Mocsn1-C, ΔMocsn2, Mocsn2-C, ΔMocsn3, Mocsn3-C, ΔMocsn4, Mocsn4-C, ΔMocsn7a, and Mocsn7a-C. The strains were cultivated in an incubator at 25 ℃ for 9 days and observed under an optical microscope. (D) Disease spots of detached barley leaves inoculated with mycelial plugs from 70-15, ΔMocsn1, Mocsn1-C, ΔMocsn2, Mocsn2-C, ΔMocsn3, Mocsn3-C, ΔMocsn4, Mocsn4-C, ΔMocsn7a, and Mocsn7a-C. Leaves were cultured at 25 ℃ for 4 days after inoculation. Figure S3. CSN subunits are involved in the regulation of autophagy in M. oryzae. (A) Yeast two-hybrid assays were used to detect the interactions between CSN subunits and Atg-related proteins. Pairs of pGBKT7-53 and PGADT7-T were used as positive controls. Yeast transformants carrying the indicated constructs were plated onto selective plates supplemented without Leu/Trp or Leu/Trp/His/Ade for growth assays. (B) Autophagic flux analysis of GFP-MoAtg8 in 70-15 and ΔMo [file 12964_2024_1598_MOESM1_ESM.zip › Supplementary files/Figure6J-UBI.tif]

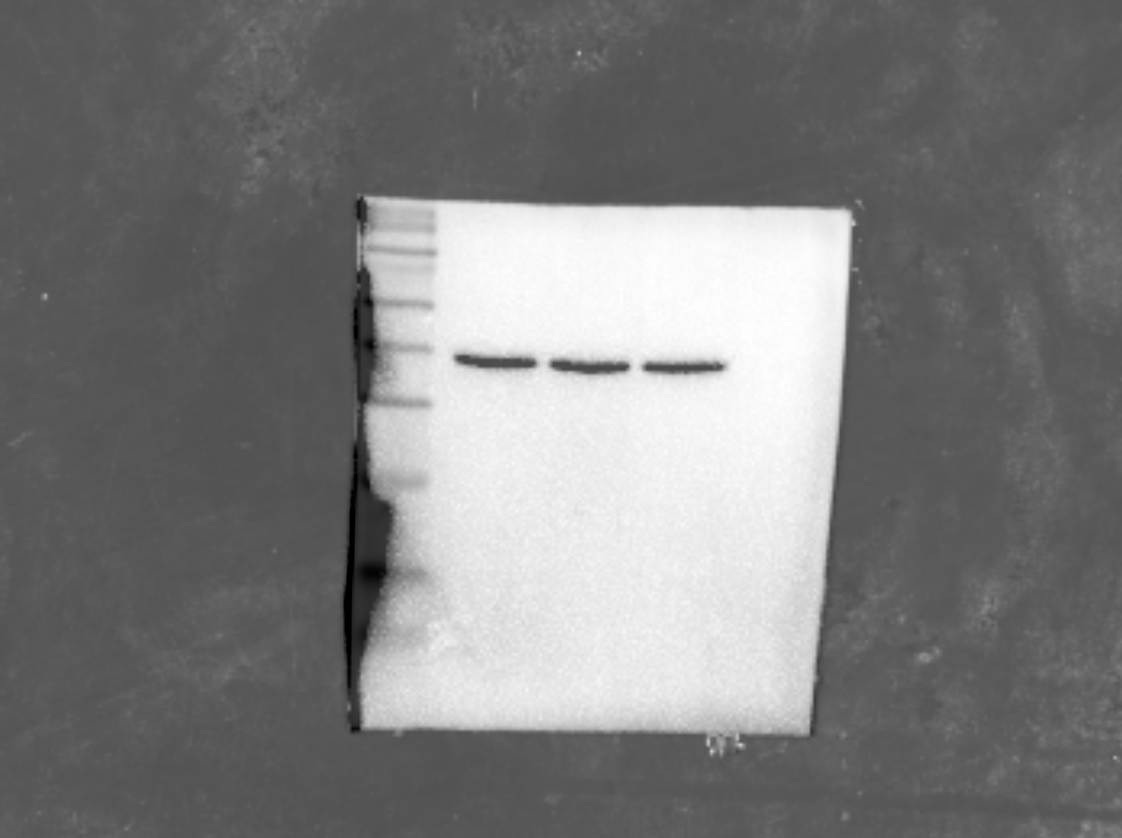

Supplement: Supplementary file 1 — Additional file 1: Figure S1. Verification of deletion mutants by PCR and quantitative real-time PCR. (A) Rice blast fungus knockout model. (B) PCR detection was performed on the null mutants, and the recombinant fragment (LF) was cloned from the positive strain but not from the WT strain. The 500-bp characteristic fragment (SF) of the target gene was cloned in the WT strain but not in the mutants. TUBULIN was used as a positive control. (C) The copy number of the resistance gene HPH in the deletion mutants was verified by quantitative real-time PCR. Figure S2. The targeted gene destruction of CSN subunits decreased the growth, sporulation, and pathogenicity of M. oryzae. (A) Colony morphology of 70-15, ΔMocsn1, Mocsn1-C, ΔMocsn2, Mocsn2-C, ΔMocsn3, Mocsn3-C, ΔMocsn4, Mocsn4-C, ΔMocsn7a, and Mocsn7a-C. The strains were grown on CM and MM plates for 9 days. (B) Statistical analysis of the colony growth diameter. The data were analyzed using GraphPad Prism 8.0 software. The error bars represent the standard deviations. ***P < 0.001. (C) Conidiophores of 70-15, ΔMocsn1, Mocsn1-C, ΔMocsn2, Mocsn2-C, ΔMocsn3, Mocsn3-C, ΔMocsn4, Mocsn4-C, ΔMocsn7a, and Mocsn7a-C. The strains were cultivated in an incubator at 25 ℃ for 9 days and observed under an optical microscope. (D) Disease spots of detached barley leaves inoculated with mycelial plugs from 70-15, ΔMocsn1, Mocsn1-C, ΔMocsn2, Mocsn2-C, ΔMocsn3, Mocsn3-C, ΔMocsn4, Mocsn4-C, ΔMocsn7a, and Mocsn7a-C. Leaves were cultured at 25 ℃ for 4 days after inoculation. Figure S3. CSN subunits are involved in the regulation of autophagy in M. oryzae. (A) Yeast two-hybrid assays were used to detect the interactions between CSN subunits and Atg-related proteins. Pairs of pGBKT7-53 and PGADT7-T were used as positive controls. Yeast transformants carrying the indicated constructs were plated onto selective plates supplemented without Leu/Trp or Leu/Trp/His/Ade for growth assays. (B) Autophagic flux analysis of GFP-MoAtg8 in 70-15 and ΔMo [file 12964_2024_1598_MOESM1_ESM.zip › Supplementary files/Figure6K-Actin.tif]

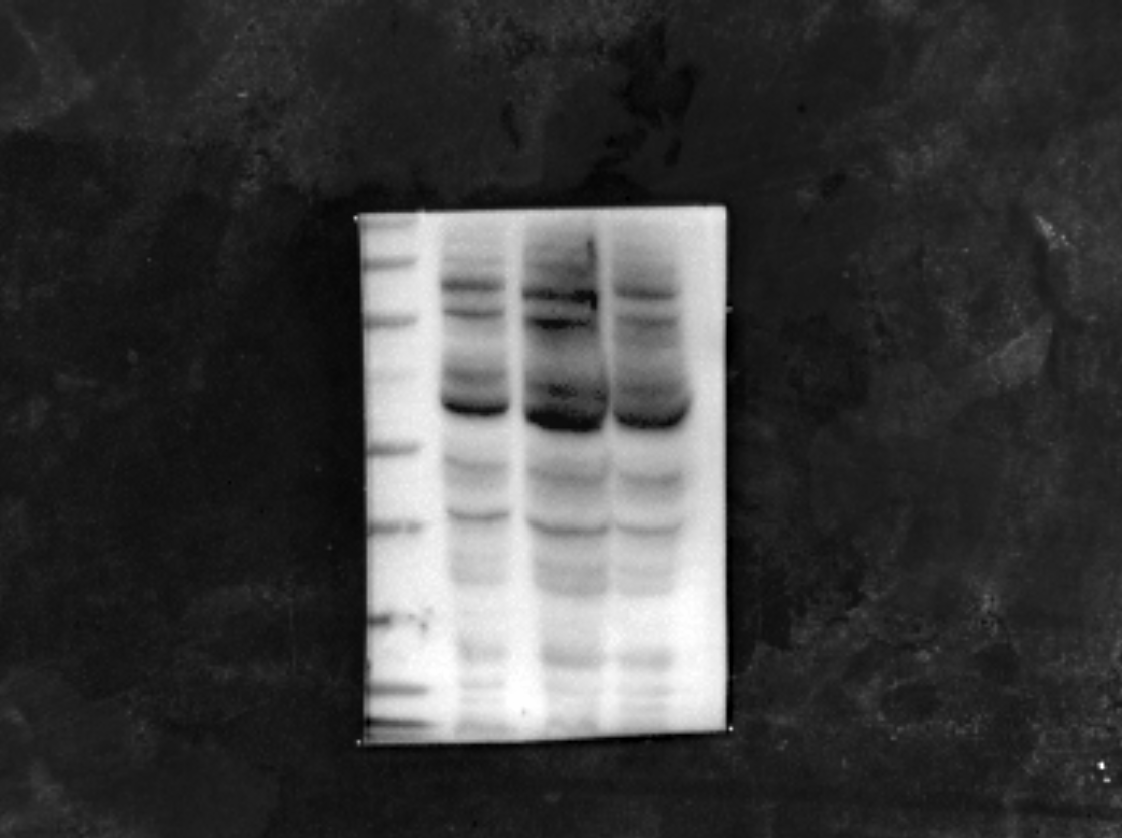

Supplement: Supplementary file 1 — Additional file 1: Figure S1. Verification of deletion mutants by PCR and quantitative real-time PCR. (A) Rice blast fungus knockout model. (B) PCR detection was performed on the null mutants, and the recombinant fragment (LF) was cloned from the positive strain but not from the WT strain. The 500-bp characteristic fragment (SF) of the target gene was cloned in the WT strain but not in the mutants. TUBULIN was used as a positive control. (C) The copy number of the resistance gene HPH in the deletion mutants was verified by quantitative real-time PCR. Figure S2. The targeted gene destruction of CSN subunits decreased the growth, sporulation, and pathogenicity of M. oryzae. (A) Colony morphology of 70-15, ΔMocsn1, Mocsn1-C, ΔMocsn2, Mocsn2-C, ΔMocsn3, Mocsn3-C, ΔMocsn4, Mocsn4-C, ΔMocsn7a, and Mocsn7a-C. The strains were grown on CM and MM plates for 9 days. (B) Statistical analysis of the colony growth diameter. The data were analyzed using GraphPad Prism 8.0 software. The error bars represent the standard deviations. ***P < 0.001. (C) Conidiophores of 70-15, ΔMocsn1, Mocsn1-C, ΔMocsn2, Mocsn2-C, ΔMocsn3, Mocsn3-C, ΔMocsn4, Mocsn4-C, ΔMocsn7a, and Mocsn7a-C. The strains were cultivated in an incubator at 25 ℃ for 9 days and observed under an optical microscope. (D) Disease spots of detached barley leaves inoculated with mycelial plugs from 70-15, ΔMocsn1, Mocsn1-C, ΔMocsn2, Mocsn2-C, ΔMocsn3, Mocsn3-C, ΔMocsn4, Mocsn4-C, ΔMocsn7a, and Mocsn7a-C. Leaves were cultured at 25 ℃ for 4 days after inoculation. Figure S3. CSN subunits are involved in the regulation of autophagy in M. oryzae. (A) Yeast two-hybrid assays were used to detect the interactions between CSN subunits and Atg-related proteins. Pairs of pGBKT7-53 and PGADT7-T were used as positive controls. Yeast transformants carrying the indicated constructs were plated onto selective plates supplemented without Leu/Trp or Leu/Trp/His/Ade for growth assays. (B) Autophagic flux analysis of GFP-MoAtg8 in 70-15 and ΔMo [file 12964_2024_1598_MOESM1_ESM.zip › Supplementary files/Figure6K-Beclin1.tif]

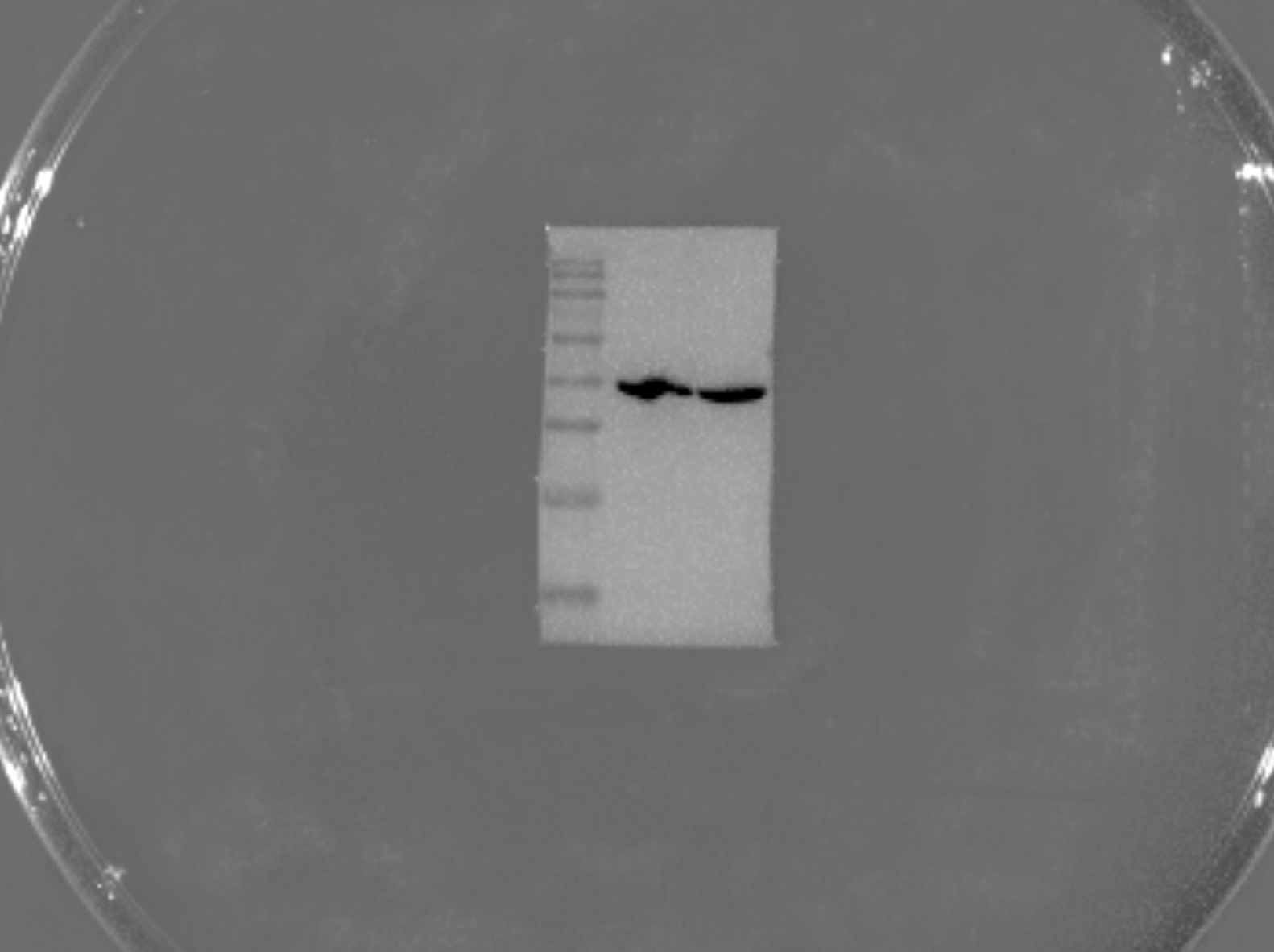

Supplement: Supplementary file 1 — Additional file 1: Figure S1. Verification of deletion mutants by PCR and quantitative real-time PCR. (A) Rice blast fungus knockout model. (B) PCR detection was performed on the null mutants, and the recombinant fragment (LF) was cloned from the positive strain but not from the WT strain. The 500-bp characteristic fragment (SF) of the target gene was cloned in the WT strain but not in the mutants. TUBULIN was used as a positive control. (C) The copy number of the resistance gene HPH in the deletion mutants was verified by quantitative real-time PCR. Figure S2. The targeted gene destruction of CSN subunits decreased the growth, sporulation, and pathogenicity of M. oryzae. (A) Colony morphology of 70-15, ΔMocsn1, Mocsn1-C, ΔMocsn2, Mocsn2-C, ΔMocsn3, Mocsn3-C, ΔMocsn4, Mocsn4-C, ΔMocsn7a, and Mocsn7a-C. The strains were grown on CM and MM plates for 9 days. (B) Statistical analysis of the colony growth diameter. The data were analyzed using GraphPad Prism 8.0 software. The error bars represent the standard deviations. ***P < 0.001. (C) Conidiophores of 70-15, ΔMocsn1, Mocsn1-C, ΔMocsn2, Mocsn2-C, ΔMocsn3, Mocsn3-C, ΔMocsn4, Mocsn4-C, ΔMocsn7a, and Mocsn7a-C. The strains were cultivated in an incubator at 25 ℃ for 9 days and observed under an optical microscope. (D) Disease spots of detached barley leaves inoculated with mycelial plugs from 70-15, ΔMocsn1, Mocsn1-C, ΔMocsn2, Mocsn2-C, ΔMocsn3, Mocsn3-C, ΔMocsn4, Mocsn4-C, ΔMocsn7a, and Mocsn7a-C. Leaves were cultured at 25 ℃ for 4 days after inoculation. Figure S3. CSN subunits are involved in the regulation of autophagy in M. oryzae. (A) Yeast two-hybrid assays were used to detect the interactions between CSN subunits and Atg-related proteins. Pairs of pGBKT7-53 and PGADT7-T were used as positive controls. Yeast transformants carrying the indicated constructs were plated onto selective plates supplemented without Leu/Trp or Leu/Trp/His/Ade for growth assays. (B) Autophagic flux analysis of GFP-MoAtg8 in 70-15 and ΔMo [file 12964_2024_1598_MOESM1_ESM.zip › Supplementary files/Figure7C-Actin.tif]

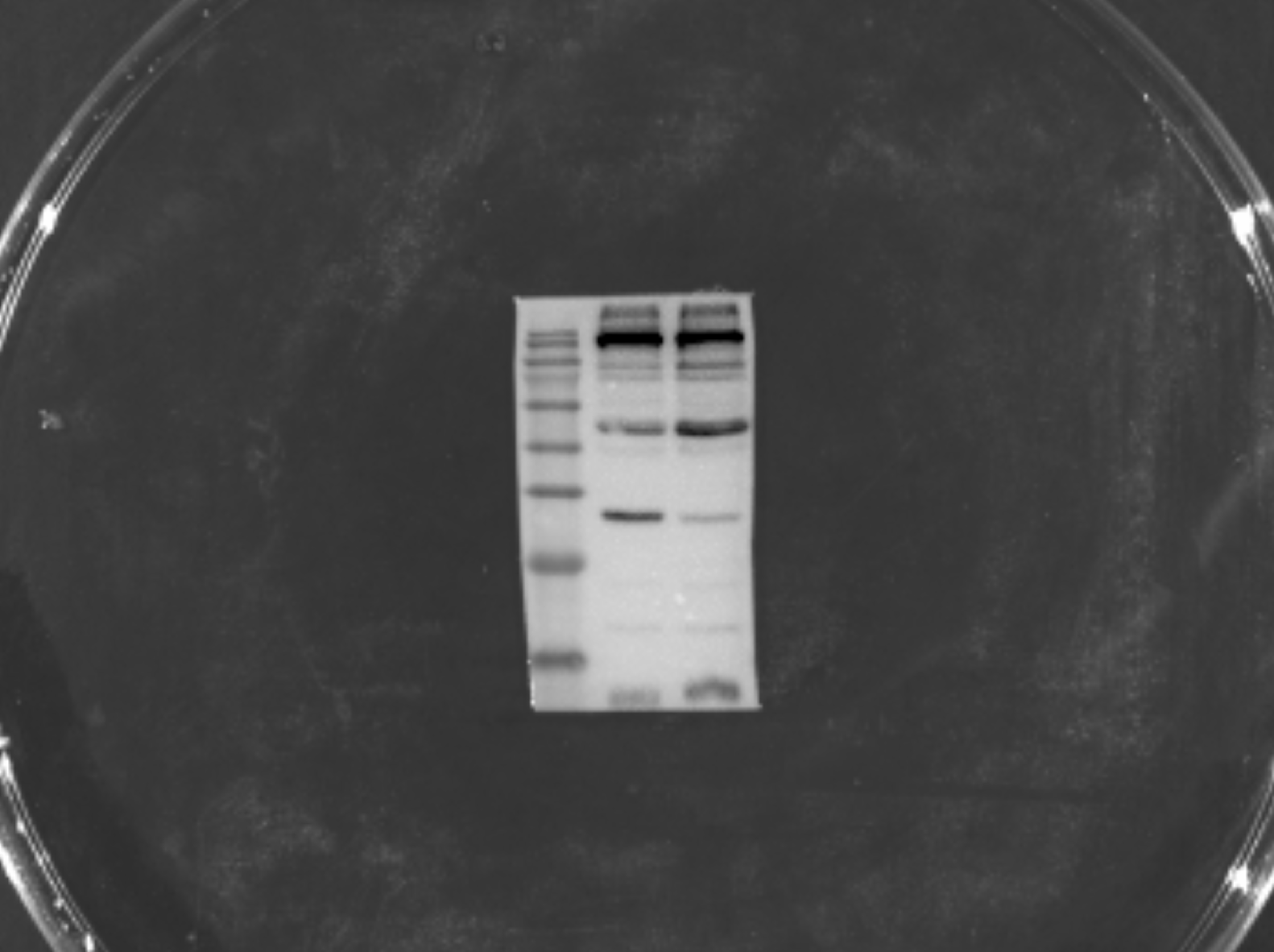

Supplement: Supplementary file 1 — Additional file 1: Figure S1. Verification of deletion mutants by PCR and quantitative real-time PCR. (A) Rice blast fungus knockout model. (B) PCR detection was performed on the null mutants, and the recombinant fragment (LF) was cloned from the positive strain but not from the WT strain. The 500-bp characteristic fragment (SF) of the target gene was cloned in the WT strain but not in the mutants. TUBULIN was used as a positive control. (C) The copy number of the resistance gene HPH in the deletion mutants was verified by quantitative real-time PCR. Figure S2. The targeted gene destruction of CSN subunits decreased the growth, sporulation, and pathogenicity of M. oryzae. (A) Colony morphology of 70-15, ΔMocsn1, Mocsn1-C, ΔMocsn2, Mocsn2-C, ΔMocsn3, Mocsn3-C, ΔMocsn4, Mocsn4-C, ΔMocsn7a, and Mocsn7a-C. The strains were grown on CM and MM plates for 9 days. (B) Statistical analysis of the colony growth diameter. The data were analyzed using GraphPad Prism 8.0 software. The error bars represent the standard deviations. ***P < 0.001. (C) Conidiophores of 70-15, ΔMocsn1, Mocsn1-C, ΔMocsn2, Mocsn2-C, ΔMocsn3, Mocsn3-C, ΔMocsn4, Mocsn4-C, ΔMocsn7a, and Mocsn7a-C. The strains were cultivated in an incubator at 25 ℃ for 9 days and observed under an optical microscope. (D) Disease spots of detached barley leaves inoculated with mycelial plugs from 70-15, ΔMocsn1, Mocsn1-C, ΔMocsn2, Mocsn2-C, ΔMocsn3, Mocsn3-C, ΔMocsn4, Mocsn4-C, ΔMocsn7a, and Mocsn7a-C. Leaves were cultured at 25 ℃ for 4 days after inoculation. Figure S3. CSN subunits are involved in the regulation of autophagy in M. oryzae. (A) Yeast two-hybrid assays were used to detect the interactions between CSN subunits and Atg-related proteins. Pairs of pGBKT7-53 and PGADT7-T were used as positive controls. Yeast transformants carrying the indicated constructs were plated onto selective plates supplemented without Leu/Trp or Leu/Trp/His/Ade for growth assays. (B) Autophagic flux analysis of GFP-MoAtg8 in 70-15 and ΔMo [file 12964_2024_1598_MOESM1_ESM.zip › Supplementary files/Figure7C-p-Rps6.tif]

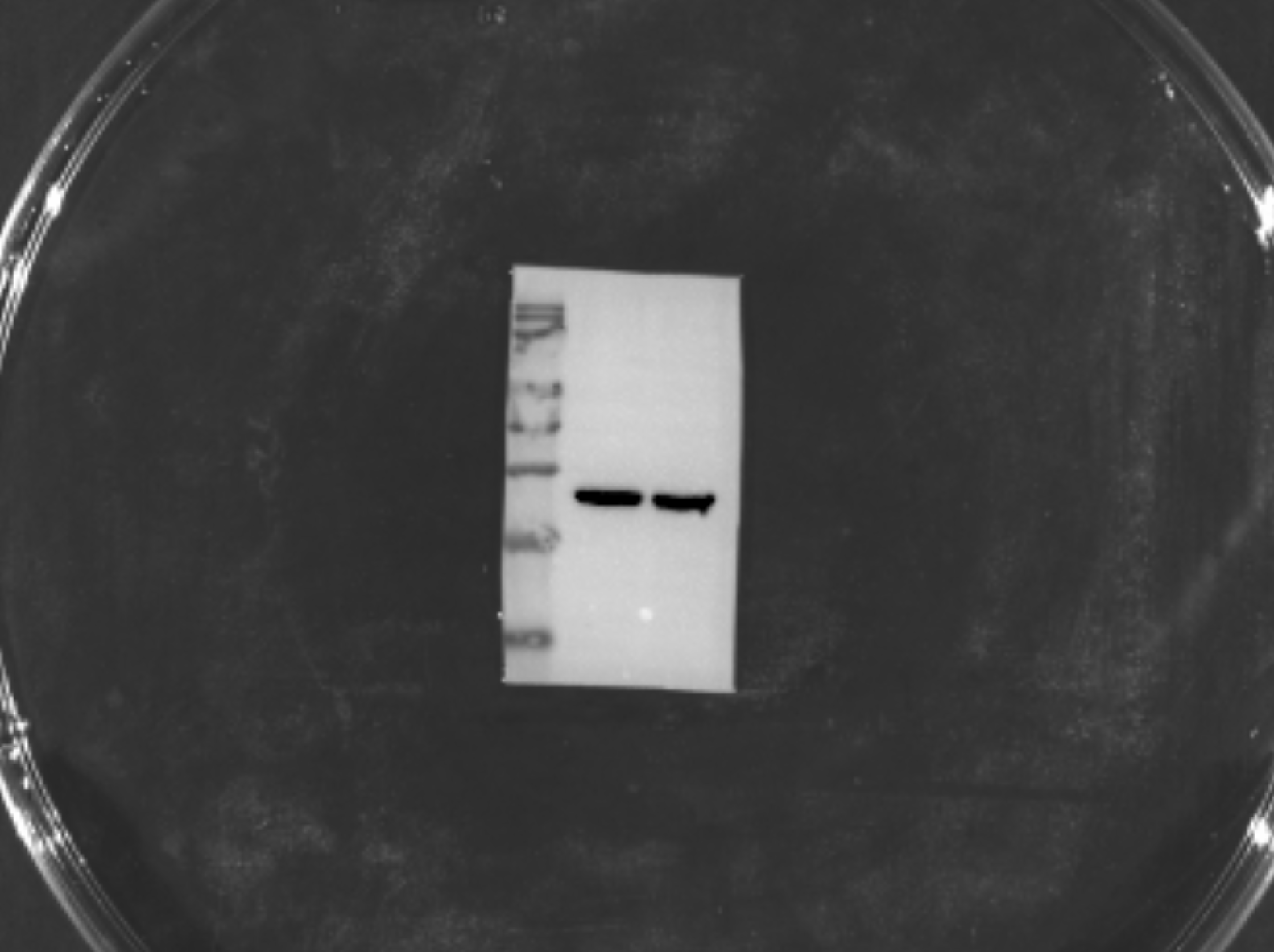

Supplement: Supplementary file 1 — Additional file 1: Figure S1. Verification of deletion mutants by PCR and quantitative real-time PCR. (A) Rice blast fungus knockout model. (B) PCR detection was performed on the null mutants, and the recombinant fragment (LF) was cloned from the positive strain but not from the WT strain. The 500-bp characteristic fragment (SF) of the target gene was cloned in the WT strain but not in the mutants. TUBULIN was used as a positive control. (C) The copy number of the resistance gene HPH in the deletion mutants was verified by quantitative real-time PCR. Figure S2. The targeted gene destruction of CSN subunits decreased the growth, sporulation, and pathogenicity of M. oryzae. (A) Colony morphology of 70-15, ΔMocsn1, Mocsn1-C, ΔMocsn2, Mocsn2-C, ΔMocsn3, Mocsn3-C, ΔMocsn4, Mocsn4-C, ΔMocsn7a, and Mocsn7a-C. The strains were grown on CM and MM plates for 9 days. (B) Statistical analysis of the colony growth diameter. The data were analyzed using GraphPad Prism 8.0 software. The error bars represent the standard deviations. ***P < 0.001. (C) Conidiophores of 70-15, ΔMocsn1, Mocsn1-C, ΔMocsn2, Mocsn2-C, ΔMocsn3, Mocsn3-C, ΔMocsn4, Mocsn4-C, ΔMocsn7a, and Mocsn7a-C. The strains were cultivated in an incubator at 25 ℃ for 9 days and observed under an optical microscope. (D) Disease spots of detached barley leaves inoculated with mycelial plugs from 70-15, ΔMocsn1, Mocsn1-C, ΔMocsn2, Mocsn2-C, ΔMocsn3, Mocsn3-C, ΔMocsn4, Mocsn4-C, ΔMocsn7a, and Mocsn7a-C. Leaves were cultured at 25 ℃ for 4 days after inoculation. Figure S3. CSN subunits are involved in the regulation of autophagy in M. oryzae. (A) Yeast two-hybrid assays were used to detect the interactions between CSN subunits and Atg-related proteins. Pairs of pGBKT7-53 and PGADT7-T were used as positive controls. Yeast transformants carrying the indicated constructs were plated onto selective plates supplemented without Leu/Trp or Leu/Trp/His/Ade for growth assays. (B) Autophagic flux analysis of GFP-MoAtg8 in 70-15 and ΔMo [file 12964_2024_1598_MOESM1_ESM.zip › Supplementary files/Figure7C-Rps6.tif]

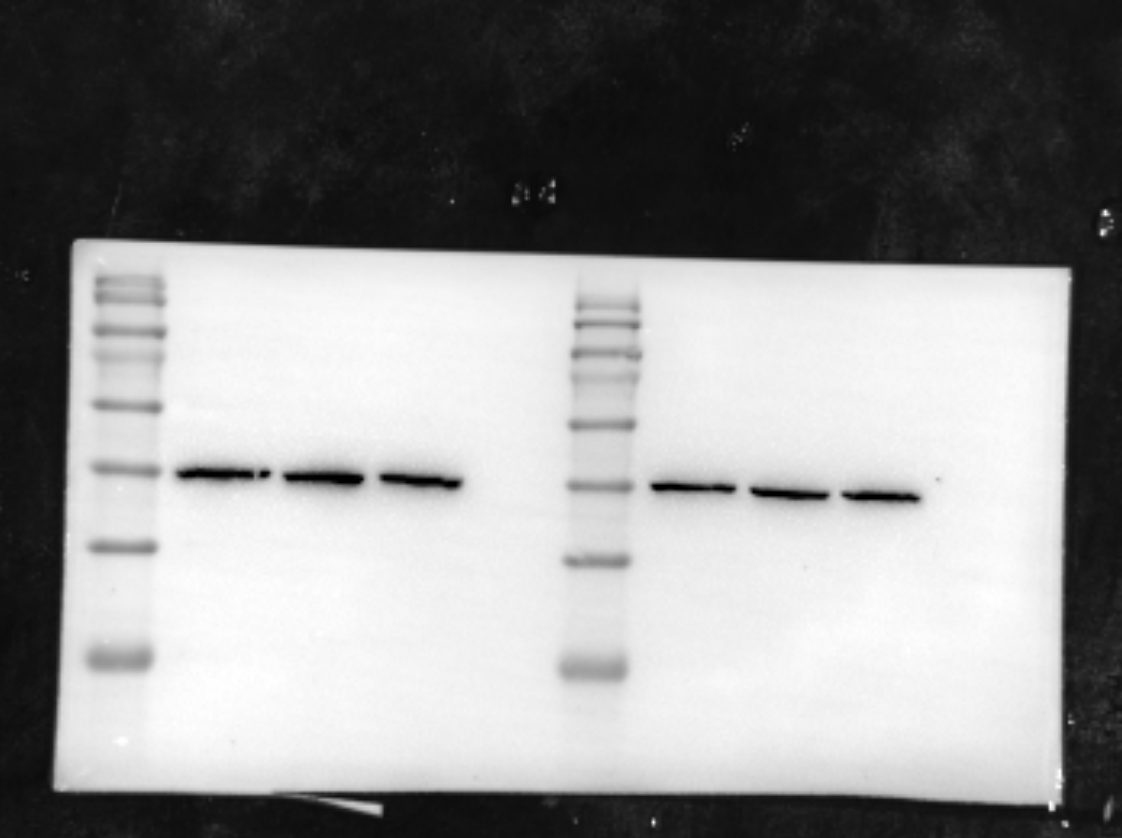

Supplement: Supplementary file 1 — Additional file 1: Figure S1. Verification of deletion mutants by PCR and quantitative real-time PCR. (A) Rice blast fungus knockout model. (B) PCR detection was performed on the null mutants, and the recombinant fragment (LF) was cloned from the positive strain but not from the WT strain. The 500-bp characteristic fragment (SF) of the target gene was cloned in the WT strain but not in the mutants. TUBULIN was used as a positive control. (C) The copy number of the resistance gene HPH in the deletion mutants was verified by quantitative real-time PCR. Figure S2. The targeted gene destruction of CSN subunits decreased the growth, sporulation, and pathogenicity of M. oryzae. (A) Colony morphology of 70-15, ΔMocsn1, Mocsn1-C, ΔMocsn2, Mocsn2-C, ΔMocsn3, Mocsn3-C, ΔMocsn4, Mocsn4-C, ΔMocsn7a, and Mocsn7a-C. The strains were grown on CM and MM plates for 9 days. (B) Statistical analysis of the colony growth diameter. The data were analyzed using GraphPad Prism 8.0 software. The error bars represent the standard deviations. ***P < 0.001. (C) Conidiophores of 70-15, ΔMocsn1, Mocsn1-C, ΔMocsn2, Mocsn2-C, ΔMocsn3, Mocsn3-C, ΔMocsn4, Mocsn4-C, ΔMocsn7a, and Mocsn7a-C. The strains were cultivated in an incubator at 25 ℃ for 9 days and observed under an optical microscope. (D) Disease spots of detached barley leaves inoculated with mycelial plugs from 70-15, ΔMocsn1, Mocsn1-C, ΔMocsn2, Mocsn2-C, ΔMocsn3, Mocsn3-C, ΔMocsn4, Mocsn4-C, ΔMocsn7a, and Mocsn7a-C. Leaves were cultured at 25 ℃ for 4 days after inoculation. Figure S3. CSN subunits are involved in the regulation of autophagy in M. oryzae. (A) Yeast two-hybrid assays were used to detect the interactions between CSN subunits and Atg-related proteins. Pairs of pGBKT7-53 and PGADT7-T were used as positive controls. Yeast transformants carrying the indicated constructs were plated onto selective plates supplemented without Leu/Trp or Leu/Trp/His/Ade for growth assays. (B) Autophagic flux analysis of GFP-MoAtg8 in 70-15 and ΔMo [file 12964_2024_1598_MOESM1_ESM.zip › Supplementary files/Figure7D-Actin(left).tif]

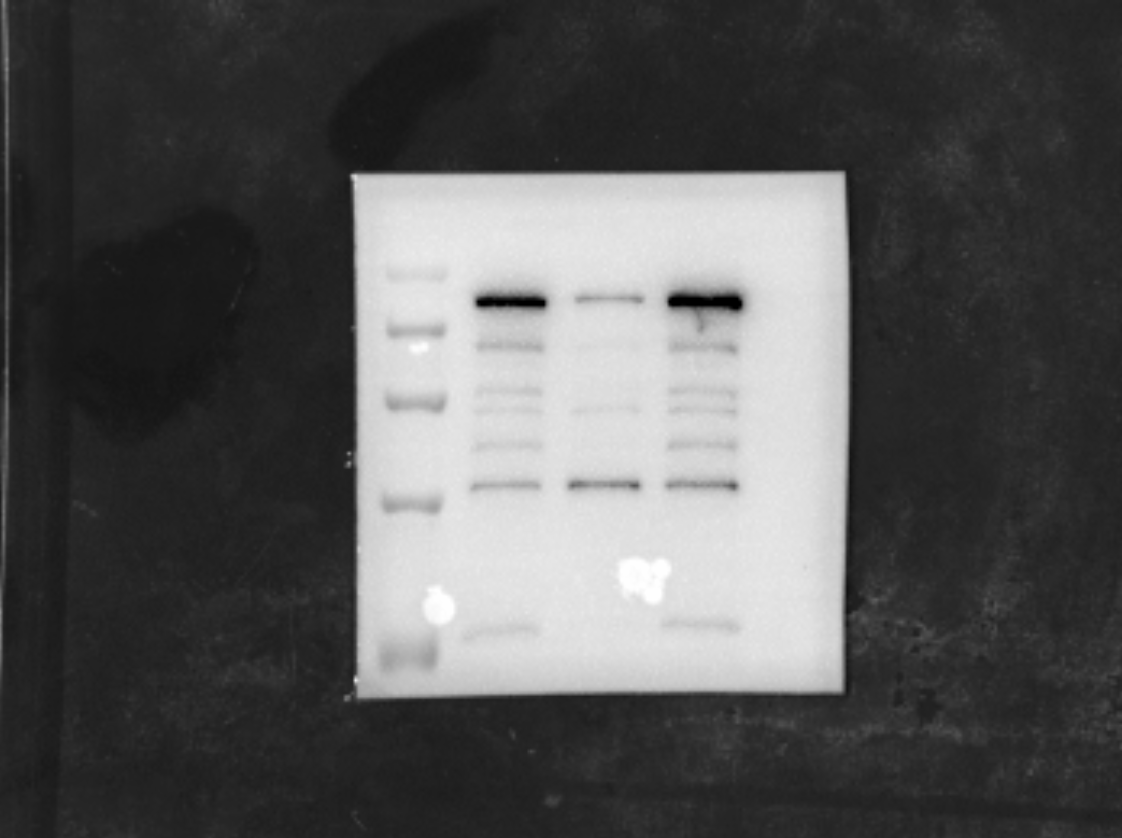

Supplement: Supplementary file 1 — Additional file 1: Figure S1. Verification of deletion mutants by PCR and quantitative real-time PCR. (A) Rice blast fungus knockout model. (B) PCR detection was performed on the null mutants, and the recombinant fragment (LF) was cloned from the positive strain but not from the WT strain. The 500-bp characteristic fragment (SF) of the target gene was cloned in the WT strain but not in the mutants. TUBULIN was used as a positive control. (C) The copy number of the resistance gene HPH in the deletion mutants was verified by quantitative real-time PCR. Figure S2. The targeted gene destruction of CSN subunits decreased the growth, sporulation, and pathogenicity of M. oryzae. (A) Colony morphology of 70-15, ΔMocsn1, Mocsn1-C, ΔMocsn2, Mocsn2-C, ΔMocsn3, Mocsn3-C, ΔMocsn4, Mocsn4-C, ΔMocsn7a, and Mocsn7a-C. The strains were grown on CM and MM plates for 9 days. (B) Statistical analysis of the colony growth diameter. The data were analyzed using GraphPad Prism 8.0 software. The error bars represent the standard deviations. ***P < 0.001. (C) Conidiophores of 70-15, ΔMocsn1, Mocsn1-C, ΔMocsn2, Mocsn2-C, ΔMocsn3, Mocsn3-C, ΔMocsn4, Mocsn4-C, ΔMocsn7a, and Mocsn7a-C. The strains were cultivated in an incubator at 25 ℃ for 9 days and observed under an optical microscope. (D) Disease spots of detached barley leaves inoculated with mycelial plugs from 70-15, ΔMocsn1, Mocsn1-C, ΔMocsn2, Mocsn2-C, ΔMocsn3, Mocsn3-C, ΔMocsn4, Mocsn4-C, ΔMocsn7a, and Mocsn7a-C. Leaves were cultured at 25 ℃ for 4 days after inoculation. Figure S3. CSN subunits are involved in the regulation of autophagy in M. oryzae. (A) Yeast two-hybrid assays were used to detect the interactions between CSN subunits and Atg-related proteins. Pairs of pGBKT7-53 and PGADT7-T were used as positive controls. Yeast transformants carrying the indicated constructs were plated onto selective plates supplemented without Leu/Trp or Leu/Trp/His/Ade for growth assays. (B) Autophagic flux analysis of GFP-MoAtg8 in 70-15 and ΔMo [file 12964_2024_1598_MOESM1_ESM.zip › Supplementary files/Figure7D-Flag.tif]

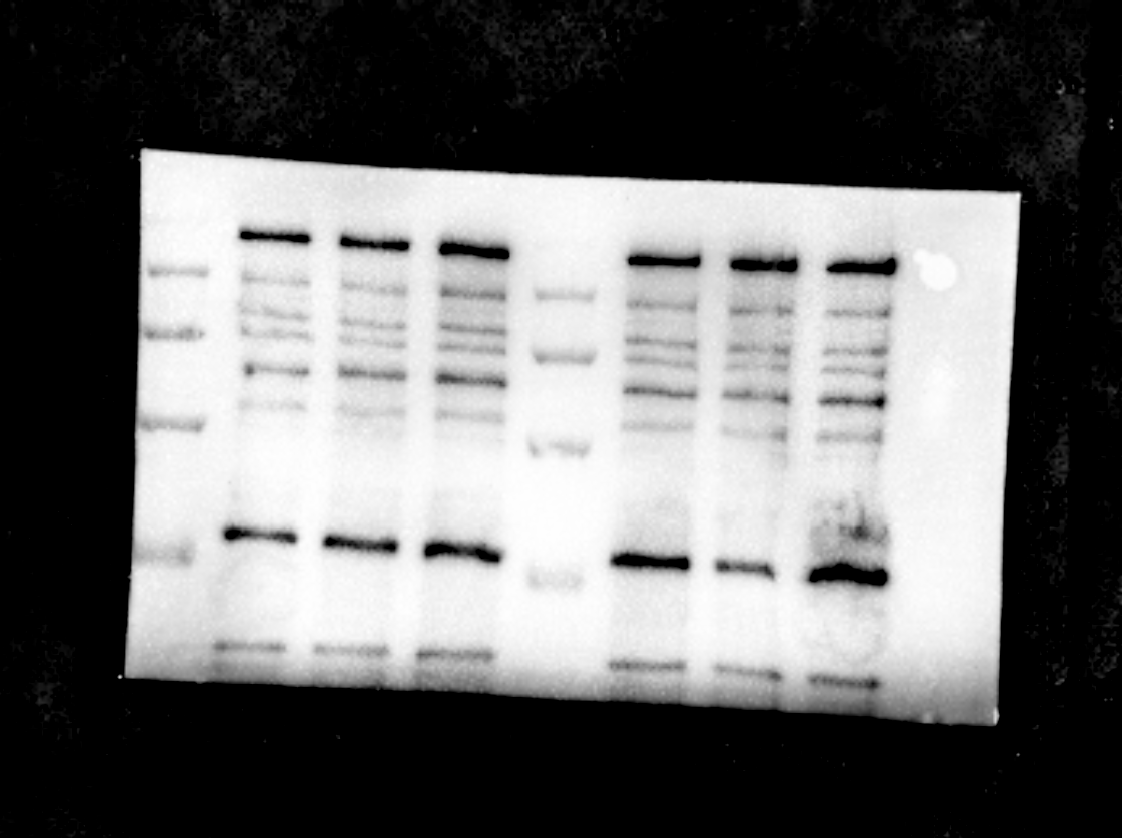

Supplement: Supplementary file 1 — Additional file 1: Figure S1. Verification of deletion mutants by PCR and quantitative real-time PCR. (A) Rice blast fungus knockout model. (B) PCR detection was performed on the null mutants, and the recombinant fragment (LF) was cloned from the positive strain but not from the WT strain. The 500-bp characteristic fragment (SF) of the target gene was cloned in the WT strain but not in the mutants. TUBULIN was used as a positive control. (C) The copy number of the resistance gene HPH in the deletion mutants was verified by quantitative real-time PCR. Figure S2. The targeted gene destruction of CSN subunits decreased the growth, sporulation, and pathogenicity of M. oryzae. (A) Colony morphology of 70-15, ΔMocsn1, Mocsn1-C, ΔMocsn2, Mocsn2-C, ΔMocsn3, Mocsn3-C, ΔMocsn4, Mocsn4-C, ΔMocsn7a, and Mocsn7a-C. The strains were grown on CM and MM plates for 9 days. (B) Statistical analysis of the colony growth diameter. The data were analyzed using GraphPad Prism 8.0 software. The error bars represent the standard deviations. ***P < 0.001. (C) Conidiophores of 70-15, ΔMocsn1, Mocsn1-C, ΔMocsn2, Mocsn2-C, ΔMocsn3, Mocsn3-C, ΔMocsn4, Mocsn4-C, ΔMocsn7a, and Mocsn7a-C. The strains were cultivated in an incubator at 25 ℃ for 9 days and observed under an optical microscope. (D) Disease spots of detached barley leaves inoculated with mycelial plugs from 70-15, ΔMocsn1, Mocsn1-C, ΔMocsn2, Mocsn2-C, ΔMocsn3, Mocsn3-C, ΔMocsn4, Mocsn4-C, ΔMocsn7a, and Mocsn7a-C. Leaves were cultured at 25 ℃ for 4 days after inoculation. Figure S3. CSN subunits are involved in the regulation of autophagy in M. oryzae. (A) Yeast two-hybrid assays were used to detect the interactions between CSN subunits and Atg-related proteins. Pairs of pGBKT7-53 and PGADT7-T were used as positive controls. Yeast transformants carrying the indicated constructs were plated onto selective plates supplemented without Leu/Trp or Leu/Trp/His/Ade for growth assays. (B) Autophagic flux analysis of GFP-MoAtg8 in 70-15 and ΔMo [file 12964_2024_1598_MOESM1_ESM.zip › Supplementary files/Figure7F+7G-Actin(left7Gright7F).tif]

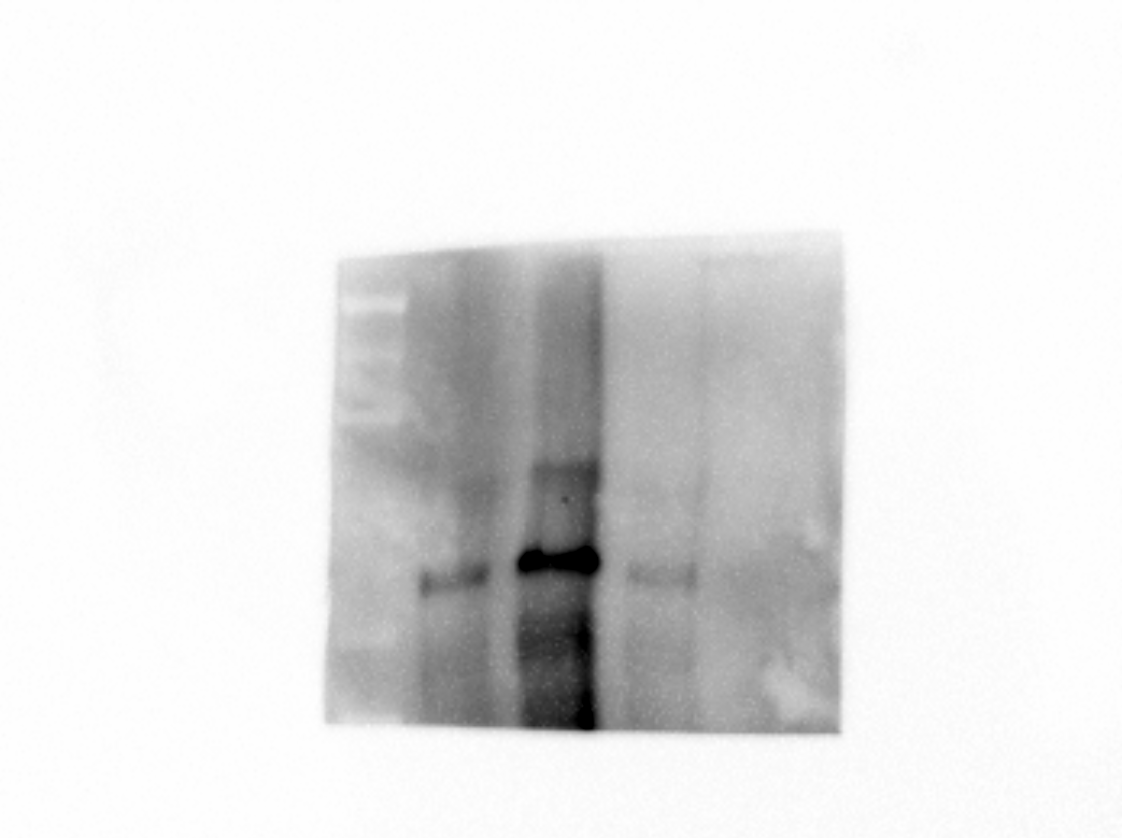

Supplement: Supplementary file 1 — Additional file 1: Figure S1. Verification of deletion mutants by PCR and quantitative real-time PCR. (A) Rice blast fungus knockout model. (B) PCR detection was performed on the null mutants, and the recombinant fragment (LF) was cloned from the positive strain but not from the WT strain. The 500-bp characteristic fragment (SF) of the target gene was cloned in the WT strain but not in the mutants. TUBULIN was used as a positive control. (C) The copy number of the resistance gene HPH in the deletion mutants was verified by quantitative real-time PCR. Figure S2. The targeted gene destruction of CSN subunits decreased the growth, sporulation, and pathogenicity of M. oryzae. (A) Colony morphology of 70-15, ΔMocsn1, Mocsn1-C, ΔMocsn2, Mocsn2-C, ΔMocsn3, Mocsn3-C, ΔMocsn4, Mocsn4-C, ΔMocsn7a, and Mocsn7a-C. The strains were grown on CM and MM plates for 9 days. (B) Statistical analysis of the colony growth diameter. The data were analyzed using GraphPad Prism 8.0 software. The error bars represent the standard deviations. ***P < 0.001. (C) Conidiophores of 70-15, ΔMocsn1, Mocsn1-C, ΔMocsn2, Mocsn2-C, ΔMocsn3, Mocsn3-C, ΔMocsn4, Mocsn4-C, ΔMocsn7a, and Mocsn7a-C. The strains were cultivated in an incubator at 25 ℃ for 9 days and observed under an optical microscope. (D) Disease spots of detached barley leaves inoculated with mycelial plugs from 70-15, ΔMocsn1, Mocsn1-C, ΔMocsn2, Mocsn2-C, ΔMocsn3, Mocsn3-C, ΔMocsn4, Mocsn4-C, ΔMocsn7a, and Mocsn7a-C. Leaves were cultured at 25 ℃ for 4 days after inoculation. Figure S3. CSN subunits are involved in the regulation of autophagy in M. oryzae. (A) Yeast two-hybrid assays were used to detect the interactions between CSN subunits and Atg-related proteins. Pairs of pGBKT7-53 and PGADT7-T were used as positive controls. Yeast transformants carrying the indicated constructs were plated onto selective plates supplemented without Leu/Trp or Leu/Trp/His/Ade for growth assays. (B) Autophagic flux analysis of GFP-MoAtg8 in 70-15 and ΔMo [file 12964_2024_1598_MOESM1_ESM.zip › Supplementary files/Figure7F-Ubi.tif]

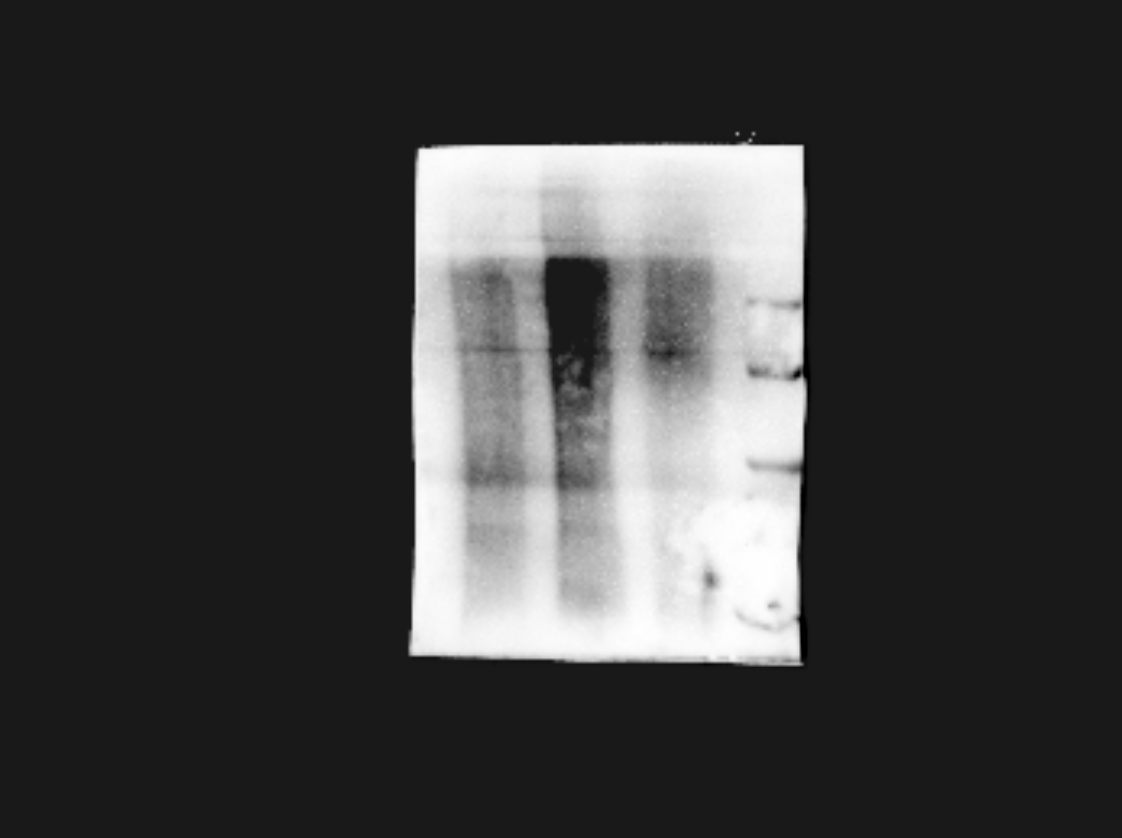

Supplement: Supplementary file 1 — Additional file 1: Figure S1. Verification of deletion mutants by PCR and quantitative real-time PCR. (A) Rice blast fungus knockout model. (B) PCR detection was performed on the null mutants, and the recombinant fragment (LF) was cloned from the positive strain but not from the WT strain. The 500-bp characteristic fragment (SF) of the target gene was cloned in the WT strain but not in the mutants. TUBULIN was used as a positive control. (C) The copy number of the resistance gene HPH in the deletion mutants was verified by quantitative real-time PCR. Figure S2. The targeted gene destruction of CSN subunits decreased the growth, sporulation, and pathogenicity of M. oryzae. (A) Colony morphology of 70-15, ΔMocsn1, Mocsn1-C, ΔMocsn2, Mocsn2-C, ΔMocsn3, Mocsn3-C, ΔMocsn4, Mocsn4-C, ΔMocsn7a, and Mocsn7a-C. The strains were grown on CM and MM plates for 9 days. (B) Statistical analysis of the colony growth diameter. The data were analyzed using GraphPad Prism 8.0 software. The error bars represent the standard deviations. ***P < 0.001. (C) Conidiophores of 70-15, ΔMocsn1, Mocsn1-C, ΔMocsn2, Mocsn2-C, ΔMocsn3, Mocsn3-C, ΔMocsn4, Mocsn4-C, ΔMocsn7a, and Mocsn7a-C. The strains were cultivated in an incubator at 25 ℃ for 9 days and observed under an optical microscope. (D) Disease spots of detached barley leaves inoculated with mycelial plugs from 70-15, ΔMocsn1, Mocsn1-C, ΔMocsn2, Mocsn2-C, ΔMocsn3, Mocsn3-C, ΔMocsn4, Mocsn4-C, ΔMocsn7a, and Mocsn7a-C. Leaves were cultured at 25 ℃ for 4 days after inoculation. Figure S3. CSN subunits are involved in the regulation of autophagy in M. oryzae. (A) Yeast two-hybrid assays were used to detect the interactions between CSN subunits and Atg-related proteins. Pairs of pGBKT7-53 and PGADT7-T were used as positive controls. Yeast transformants carrying the indicated constructs were plated onto selective plates supplemented without Leu/Trp or Leu/Trp/His/Ade for growth assays. (B) Autophagic flux analysis of GFP-MoAtg8 in 70-15 and ΔMo [file 12964_2024_1598_MOESM1_ESM.zip › Supplementary files/Figure7G-K48.tif]
