# Supplementary material for: Csn5 inhibits autophagy by regulating the ubiquitination of Atg6 and Tor to mediate the pathogenicity of Magnaporthe oryzae
Source: Cell Commun Signal. 2024 Apr 9;22:222. doi: 10.1186/s12964-024-01598-7 (PMC11003145; doi:10.1186/s12964-024-01598-7)
Supplement: Supplementary file 2 — Additional file 2: Table S1. Primers used in this study. [file 12964_2024_1598_MOESM2_ESM.docx]

**Table S1 Primers used in this study**

| **Primer name** | **Primer** |
| --- | --- |
| **Primers for gene knock out and complementation** | |
| CSN1-Up-F | AGGCTAACTGACACTCTAGACACTAGCTCTAGCCTAGACGT |
| CSN1-Up-R | TGTTGACCTCCACTAAGCTGACAGCGTTAATAAGGCT |
| CSN1-Down-F | GGAATAGAGTAGATGGTTAGGCTCGGACTTCTAGGT |
| CSN1-Down-R | CGACGGCCAGTGCCAAGCTTAAGGAGGCCGAGGAGGAAAG |
| CSN1-Long-F | CCTGATAATGCCAAGCCC |
| CSN1-Short-F | TGTCGCTCACTCATCATGTC |
| CSN1-Short-R | CTTCGTCTCTTCCCTGTTG |
| CSN1C-F | ATCACAATGGCCGGATCCATGGCGACTCCTCATGAGAAGC |
| CSN1C-R | CTTGCTCACCATCCCGGGAAATACGGATGCCATCGCTGAA |
| CSN2-Up-F | AGGCTAACTGACACTCTAGACCGTTCATCATCCCTTGG |
| CSN2-Up-R | TGTTGACCTCCACTACGTTGTGACTGGGTTTACT |
| CSN2-Down-F | GGAATAGAGTAGATGCGGCAGTTGGTAGCGCATTCAC |
| CSN2-Down-R | CGACGGCCAGTGCCAAGCTTGAAGAAAACGGAAAGTAGGGCG |
| CSN2-Long-F | GCTGAATGTTGTGTCGCC |
| CSN2-Short-F | GCCGTGGATATCGAGAACAAGT |
| CSN2-Short-R | TTCCTATCGAGCAGAAGC |
| CSN2C-F | ATCACAATGGCCGGATCCATGTCCGACGACGACTTCATGC |
| CSN2C-R | CTTGCTCACCATCCCGGGTGTAAAGGTCGAATGAACTGCC |
| CSN3-Up-F | AGGCTAACTGACACTCTAGACCTCTGCCATGGGACTAT |
| CSN3-Up-R | TGTTGACCTCCACTAGCTTTCGAGGTTTCTGTCGT |
| CSN3-Down-F | GGAATAGAGTAGATGGTGACATTGTGTGGACTGAACC |
| CSN3-Down-R | CGACGGCCAGTGCCAAGCTTCAAGGTTGGCTCACTTGC |
| CSN3-Long-F | TGGCTTCGGTGGACATGT |
| CSN3-Short-F | ATTCAAGGAGCGTAGCGCTGT |
| CSN3-Short-R | TTGATGTGAGTACGGAAC |
| CSN3C-F | ATCACAATGGCCGGATCCATGGATCACTGCGCGTCGGTCC |
| CSN3C-R | CTTGCTCACCATCCCGGGCTGCCCAGATACTATGCCGGAC |
| CSN4-Up-F | AGGCTAACTGACACTCTAGAAGGTTAACCGCGAAATGT |
| CSN4-Up-R | TGTTGACCTCCACTATCCGTATCGCGTCGAAAAGCGC |
| CSN4-Down-F | GGAATAGAGTAGATGTAGCTCGGCGTTTTTAGAGT |
| CSN4-Down-R | CGACGGCCAGTGCCAAGCTTATCGAGACTGAGAATCTGT |
| CSN4-Long-F | GAAATCACGCTCCCTGACAACC |
| CSN4-Short-F | TGGAGAAGCTGCTTGTTGACTT |
| CSN4-Short-R | GTAGGTTGAGATCGCGGT |
| CSN4C-F | ATCACAATGGCCGGATCCATGGCCTCCGACTCGATAAAGG |
| CSN4C-R | CTTGCTCACCATCCCGGGGACTACCAAGTTGGCCGCAACA |
| CSN5-Up-F | AGGCTAACTGACACTCTAGAACCGCCCAAAAAGCAAGGGAT |
| CSN5-Up-R | TGTTGACCTCCACTATCGTAAGCCTCTCAGTAAAG |
| CSN5-Down-F | GGAATAGAGTAGATGACTGGCCTTAGTTTCTCTT |
| CSN5-Down-R | CGACGGCCAGTGCCAAGCTTGAAGAAGGGAGCGCTGACTCT |
| CSN5-Long-F | TCAGGTTCTCTCTCGGATCT |
| CSN5-SF | CTCTGCCCCTACTCTTGAT |
| CSN5-SR | AACTGCGTGCTCACGTCGATGC |
| CSN5C-GFP-F | ATCACAATGGCCGGATCCATGGATGTTGCTATGAAGTCGT |
| CSN5C-GFP-R | CTTGCTCACCATCCCGGGCGACGCAGCCGACGGCTCAGCC |
| CSN5Cnative-GFP-F | ACAATCACTAGTGAATTCATGAGGACACCGGTTTCAGGAG |
| CSN5Cnative-GFP-R | CATCCCGGGGATGGATCCCGACGCAGCCGACGGCTCAGCC |
| CSN5C-Flag-F | ATCACAATGGCCGGATCCATGGATGTTGCTATGAAGTCGT |
| CSN5C-Flag-R | GTCCTTGTAGTCCCCGGGCGACGCAGCCGACGGCTCAGCC |
| CSN7a-Up-F | AGGCTAACTGACACTCTAGAACAAGGCCAAGCAGACTAGGT |
| CSN7a-Up-R | TGTTGACCTCCACTATGCGGGGTAGCATCCAATTC |
| CSN7a-Down-F | GGAATAGAGTAGATGCTAGGCTTCCGGATTAAT |
| CSN7a-Down-R | CGACGGCCAGTGCCAAGCTTGAGCGTGAAATCGTGCTTCC |
| CSN7a-Long-F | TCACTGACAGGTTACACAG |
| CSN7a-Short-F | ACCCGCAATGCTAACTTGTC |
| CSN7a-Short-R | TGATAAGGTCTGGAACAGTTC |
| CSN7aC-F | ATCACAATGGCCGGATCCATGGAGCAAGCAAAGGCTCTCA |
| CSN7aC-R | CTTGCTCACCATCCCGGGAAGCTTTCTTCGACTCGACCTC |
| Long-HPH-R | GTCGGAGACGCTGTCGAACTT |
| HPH-F | TAGTGGAGGTCAACAATGAATG |
| HPH-R | CATCTACTCTATTCCTTTGCCC |
| TUBULIN-F | CCATCCCGAGCTTGTTGATA |
| TUBULIN-R | GTAGTTCAGGTCACCGTATGAG |
| **Primer for yeast two hybrid** | |
| CSN1-AD-F | ATGGAGGCCAGTGAATTCATGGCGACTCCTCATGAGAAGC |
| CSN1-AD-R | CTCGAGCTCGATGGATCCAAATACGGATGCCATCGCTGAA |
| CSN3-AD-F | ATGGAGGCCAGTGAATTCATGGATCACTGCGCGTCGGTCC |
| CSN3-AD-R | CTCGAGCTCGATGGATCCCTGCCCAGATACTATGCCGGAC |
| CSN4-AD-F | ATGGAGGCCAGTGAATTCATGGCCTCCGACTCGATAAAGG |
| CSN4-AD-R | CTCGAGCTCGATGGATCCGACTACCAAGTTGGCCGCAACA |
| CSN5-BD-F | GCCATGGAGGCCGAATTCATGGATGTTGCTATGAAGTCGT |
| CSN5-BD-R | CTGCAGGTCGACGGATCCCGACGCAGCCGACGGCTCAGCC |
| CSN7a-BD-F | GCCATGGAGGCCGAATTCATGGAGCAAGCAAAGGCTCTCA |
| CSN7a-BD-R | CTGCAGGTCGACGGATCCAAGCTTTCTTCGACTCGACCTC |
| CULLIN3-AD-F | ATGGAGGCCAGTGAATTCATGCAGCGTCAGAACAAGATAC |
| CULLIN3-AD-R | CTCGAGCTCGATGGATCCTGCCATGTACTTGTAACCAGAA |
| ATG5-BD-F | GCCATGGAGGCCGAATTCATGGCTTCGCCGCGCCGATCAG |
| ATG5-BD-R | CTGCAGGTCGACGGATCCTAATGGCACGACGGTTAAACAA |
| ATG6-AD-F | ATGGAGGCCAGTGAATTCATGATGTTTTGCCAAAAATGCC |
| ATG6-AD-R | CTCGAGCTCGATGGATCCGGTCGAGCTTGAGCCCAAAACC |
| ATG6-BD-F | CATGGAGGCCGAATTCATGATGTTTTGCCAAAAATGCCG |
| ATG6-BD-R | GCAGGTCGACGGATCCGGTCGAGCTTGAGCCCAAAAC |
| ATG12-BD-F | GCCATGGAGGCCGAATTCATGTCGTCTCCACCGACACGTT |
| ATG12-BD-R | CTGCAGGTCGACGGATCC CCCAAAGGCAGGAGTCATGGAG |
| ATG14-AD-F | ATGGAGGCCAGTGAATTCATGTCATGCTATATCTGCGGAC |
| ATG14-AD-R | CTCGAGCTCGATGGATCCAGTCCAAGCATCATCCTCCAGC |
| ATG14-BD-F | GCCATGGAGGCCGAATTCATGTCATGCTATATCTGCGGAC |
| ATG14-BD-R | CTGCAGGTCGACGGATCC AGTCCAAGCATCATCCTCCAGC |
| ATG16-BD-F | GCCATGGAGGCCGAATTCATGTCTTCGCTGCCGGACTGGC |
| ATG16-BD-R | CTGCAGGTCGACGGATCCTCCTCGTTTGGCGAATTTCGGC |
| ATG17-BD-F | GCCATGGAGGCCGAATTCATGCCGTCTTCAAGTTCCGCCA |
| ATG17-BD-R | CTGCAGGTCGACGGATCC ACGTCCATGTACCCTCTCCCTC |
| ATG18-BD-F | GCCATGGAGGCCGAATTCATGGCGACTGCAACGCTAAACT |
| ATG18-BD-R | CTGCAGGTCGACGGATCC AGACTCATATGTCGAAGAAGAG |
| **Primer for Pulldown, Co-IP, and colocalization** | |
| ATG14-GST-F | CTGGTTCCGCGTGGATCCATGTCATGCTATATCTGCGGAC |
| ATG14-GST-R | GAGTCGACCCGGGAATTCAGTCCAAGCATCATCCTCCAGC |
| CSN5-His-F | CCACGCGGTTCTGGATCCATGGATGTTGCTATGAAGTCGT |
| CSN5-His-R | TCGACGGAGCTCGAATTCCGACGCAGCCGACGGCTCAGCC |
| ATG6-GFP-F | CAATCACAATGGCCGGATCC ATGATGTTTTGCCAAAAAT |
| ATG6-GFP-R | CCTTGCTCACCATCCCGGG GGTCGAGCTTGAGCCCAAAAC |
| ATG14-GFP-F | ATCACAATGGCCGGATCCATGTCATGCTATATCTGCGGAC |
| ATG14-GFP-R | CTTGCTCACCATCCCGGGAGTCCAAGCATCATCCTCCAGC |
| TOR-Flag-F | CAATCACAATGGCCGGATCC ATGTCTTCACCAAGCCCAGGC |
| TOR-Flag-R | TGGTCCTTGTAGTCCCCGGG CCAGAAGCTGCACCATCCAA |
| H2B-mCherry-F | ATGGTCGGATCCATCCCCGGGATGCCCCCCAAGGCCGCTGACAA |
| H_2_B-mCherry-R | TTACTGCAGGTCGACTCTAGAGCCGCCGGTGGAGTGGCGGCC |
| **Primer for quantitative real-time PCR** | |
| HPH-RT-F | ATGTCCTGCGGGTAAATAGC |
| HPH-RT-R | GATGCAATAGGTCAGGCTCTC |
| TUBULIN-RT-F | ACAACTTCGTCTTCGGTCAG |
| TUBULIN-RT-R | GTGATCTGGAAACCCTGGAG |
| qRT-ACTIN-F | ACAATGGTTCGGGTATGTGC |
| qRT-ACTIN-R | CGACAATGGACGGGAAGAC |
| qRT-TOR-F | AGTGTGATGGCTGTTCTCG |
| qRT-TOR-R | TGCTTGGCGTCTTGGTTAG |
| qRT-ATG6-F | GATCTCAACCCAGCCGC |
| qRT-ATG6-R | CCTTCCTGTTCTGATCGTGG |
| qRT-SKP1-F | TGGGATCAGAAGTTCATGCAG |
| qRT-SKP1-R | ATATCAAGGTAGTTGCTCGCC |
| qRT-CULLIN1-F | CAGGAAGAGGTGGAGACAAAC |
| qRT-CULLIN1-R | ACAAACCGTCCCTTGATCTG |
| qRT-CULLIN3-F | AGCGTCAGAACAAGATACGG |
| qRT-CULLIN3-R | TGGCTCGGTATAAATGCTCG |
| qRT-CULLIN4b-F | AGGTCATGGCGAAAGATAACG |
| qRT-CULLIN4b-R | GATGAATGTTCGGTCCAGGTAG |
